# Structural and conformational trends of silver(i) and iodine(i) complexes of 3-substituted pyridines

**DOI:** 10.1039/d6ra07510j

**Published:** 2026-10-20

**Authors:** Oskari Koivisto, Ari Lehtonen, Anssi Peuronen

**Affiliations:** a Department of Chemistry, University of Turku Turku Finland anssi.peuronen@utu.fi

## Abstract

To systematically explore the impact of *meta*-substituents on the halogen bond of [bis(pyridine)iodine(i)]^+^ complexes, five new iodine(i) complexes of 3-substituted pyridines 1–5 with substituents ranging from weakly electron-donating (–CH_3_) to strongly electron-withdrawing (–F) have been synthesized. The iodine(i) complexes were prepared from their silver(i) analogs by cation exchange. Crystals of both the silver(i) and iodine(i) complexes were grown by vapor diffusion and analyzed by single-crystal X-ray diffraction. Notably, in each case the silver(i) and iodine(i) complexes were found to crystallize in opposite *syn*–*anti* conformations with respect to the 3-substituent. However, DFT calculations indicate no preference for either conformation in any of the complexes, suggesting that the observed phenomenon is coincidental and the result of packing forces. The silver(i) complex of the 2,5-disubstituted pyridine 6 adopted different structures depending on the counterion: it existed as discrete [6–Ag–6]^+^ units in the PF_6_^−^ salt, formed dimers of [6–Ag–6]^+^ in the BF_4_^−^ salt and assembled into polymeric 6-Ag-PO_2_F_2_ chains. The latter two counterions resulted from the hydrolysis of PF_6_^−^. Crystallographic studies were augmented with ^1^H and ^1^H–^15^N HMBC NMR studies in solution. This revealed only small differences in ^15^N NMR shifts between the different pyridines, indicating that substituents in the 3-position have a diminished ability to influence the halogen bonds in [bis(pyridine)iodine(i)]^+^ complexes. This means that the 3-position can be employed in the design of supramolecular motifs without any concerns relating to the potential weakening of the N–I^+^–N bond.

## Abstract 2

Crystallographic and NMR studies of a series of *meta*-substituted pyridines reveal that the 3-position weakens the impact of substituents on the halogen bonds in [bis(pyridine)iodine(i)]^+^ complexes.

## Graphical abstract

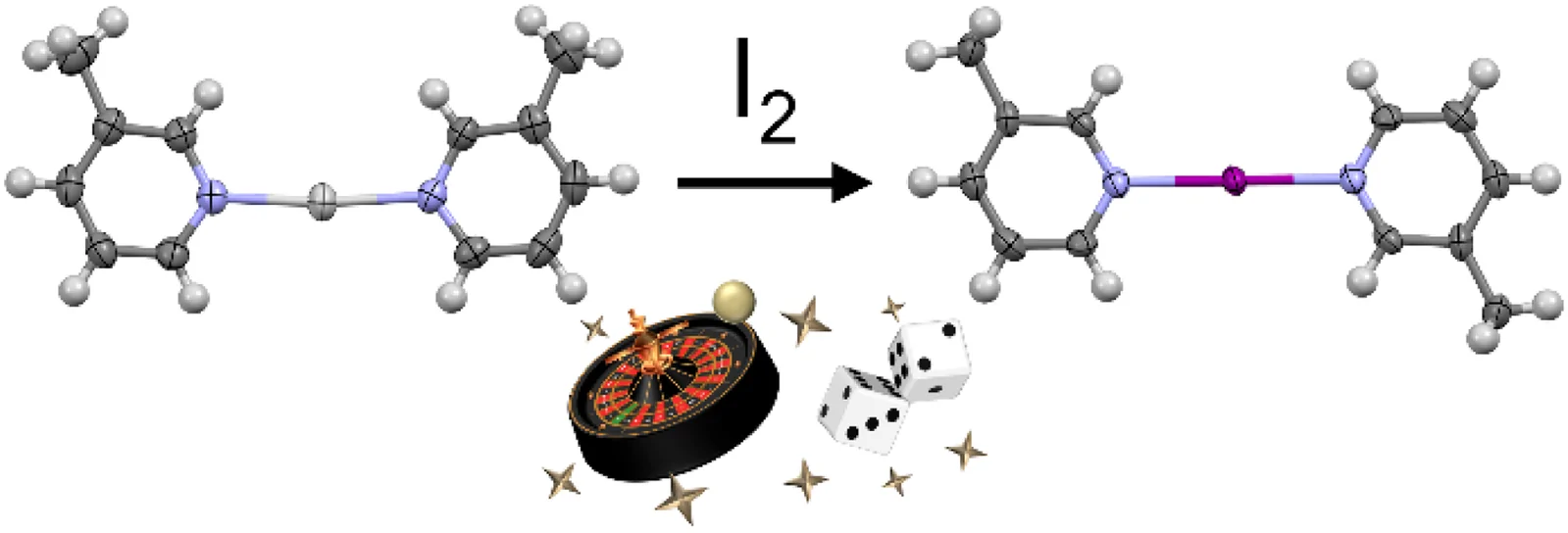

## Introduction

The halogen bond, defined as the attractive interaction between an electrophilic region in a polarized halogen atom and a Lewis base,^1^ has emerged as a core non-covalent interaction owing to its directionality, strength, and tunability.^2–6^ The strongest halogen bonds are found in the extreme case of polarization in which the halogen bond donor is a halogen(I) cation.^4,5,7^ Halogen(I) cations can be stabilized by an interaction with two Lewis bases, leading to the formation of a [L–X–L]^+^ complex, with iodine(i) complexes generally being the most stable.^5,8^ The archetypal example of these is the iodine(i) complex bis(pyridine)iodine(i) tetrafluoroborate or Barluenga's reagent, used in oxidation and iodination reactions since the 1980s.^9,10^

Iodine(i) complexes, and more specifically, Barluenga-type [N–I–N]^+^ complexes with pyridines as the Lewis bases have received by far the most attention in the literature and are characterized by symmetry, linearity, strength, and short I–N bonds.^7,11–14^ As the field has advanced and the focus shifted towards applications, examples of asymmetric heteroleptic complexes,^15,16^ complexes with alternative Lewis bases,^17–19^ and supramolecular capsules,^20–22^ helicates,^23,24^ and frameworks^25–30^ have been reported.

The ability to tune the strength of the [N–I–N]^+^ halogen bond in [bis(pyridine)iodine(i)]^+^ complexes using a variety of substituents in the *ortho*-^31^ and *para*-positions^14^ has already been explored. Slight differences in the pyridine influence reactivity, as the iodine(i) complex of pyridine and that of 2,4,6-trimethylpyridine lead to different products in the iodocyclization of α-propargylic glycine derivatives.^32^ On the other hand, examples of complexes with *meta*-substituted pyridines have only recently emerged, with the first example, [bis(3-acetaminopyridine)iodine(i)]PF_6_, exhibiting unexpected reactivity towards etheric solvents.^33,34^ The impact of different substituents in the *meta*-position has not yet been thoroughly investigated experimentally,^33,34^ and in addition to revealing new information on the nature of the [N–I–N]^+^ halogen bond, *meta*-substituted pyridines offer an opportunity to study the conformation of their complexes. Computational results by Velasquez, Echeverría, and Alvarez^35^ indicate that *meta*-substituents have a weaker effect on the strength of the halogen bond in [bis(pyridine)iodine(i)]^+^ complexes compared to *para*-substituents, while steric effects play a pivotal role for *ortho*-substituents. Herein, inspired by the recent work of Kumar *et al.*^31^ on the complexes of *ortho*-substituted pyridines, we report the synthesis and characterization of a series of silver(i) and iodine(i) complexes of six *meta*-substituted pyridines 1–6, with a focus on simple methyl and halogen substituents (Scheme 1). Crystal structures of the complexes of 1–6 are described, while density functional theory (DFT) is utilized in the assessment of an observed trend of opposite conformations upon crystallization. The crystallographic analysis is accompanied by solution-state ^1^H and ^1^H–^15^N HMBC NMR studies. Together, these approaches elucidate the influence of *meta*-substituents on the halogen bonds of [bis(pyridine)iodine(i)]^+^ complexes.

**Scheme 1:**
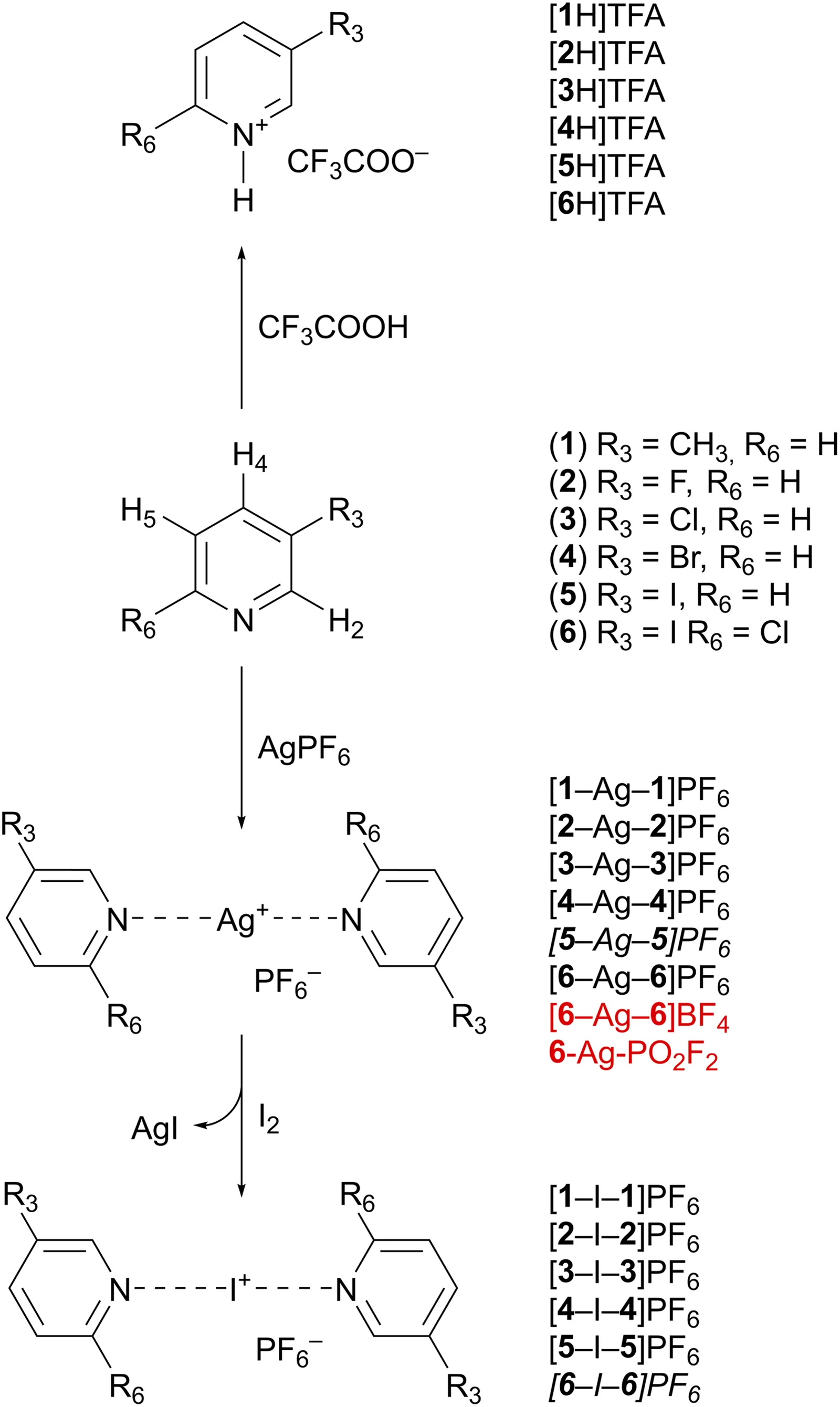
General synthetic routes to [LH]TFA, [L–Ag–L]PF_6_ and [L–I–L]PF_6_ complexes of 3-substituted pyridines 1–5 and 2,5-disubstituted pyridine 6. Complexes in italics did not give ^15^N NMR signals and could not be crystallized, whereas the tetrafluoroborate and difluorophosphate in red were unexpected products.

## Results and discussion

### Molecular structure

Single-crystal structures of the obtained complexes confirm the successful synthesis of [bis(pyridine)silver(i)]^+^ complexes and the subsequent formation of the respective iodine(i) species following the reaction with I_2_. The iodine(i) complexes of 1–5 show a characteristic preference for a linear geometry with N–I–N bond angles of 174.6(2)–180° (see bond parameters in Table 1). A similar trend is observed for the respective silver(i) complexes of 1–5 and the PF_6_^−^ salt of 6, in which the N–Ag–N bond angles fall within the range of 173.6(2)–180°. On the other hand, [6–Ag–6]BF_4_ shows significant bending of the N–Ag–N angle [161.1(2)°] due to stronger Ag⋯anion interactions compared to the PF_6_^−^ salts and the existence of a seemingly short Ag⋯I–C contact (see more detailed discussion below). These interactions are also reflected in the elongated Ag–N bond lengths [2.236(3)–2.237(3)°] observed in the [6–Ag–6]BF_4_ complex which significantly deviate from the respective values in the PF_6_^−^ salts [2.11(2)–2.175(3)Å]. The I–N bond lengths occupy a narrow range, 2.251(5)–2.279(5) Å, typical of [bis(pyridine)iodine(i)]^+^ complexes.^4,35^ Differences in bond lengths between the complexes of 2–5 and their *ortho*- and *para*-isomers are minimal (<1%).^31,36^ The dihedral angle *φ* between the two planes defined by the pyridine rings varies between 0–18.1(2)° for the iodine(i) complexes and 0–25.3(2)° for the silver(i) PF_6_^−^ complexes. [6–Ag–6]BF_4_ is again an exception as *φ* is significantly larger [52.3(2)°] than any of the other iodine(i) or silver(i) PF_6_^−^ complexes.

**Table 1:** Bond lengths *d* (X = silver(i) or iodine(i)), bond angles ∠ and dihedral angles *φ* in the crystal structures of silver(i) and iodine(i) complexes of 1–6

Complex	*d*(X–N) (Å)	∠N–X–N; *φ* (°)
[1–Ag–1]PF_6_	2.122(3), 2.126(3)	173.6(2); 2.7(2)
[1–I–1]PF_6_	2.263(8)	180; 0
[2–Ag–2]PF_6_	2.142(2)	179.1(2); 0.1(2)
[2–I–2]PF_6_	2.259(2)	180; 0
[3–Ag–3]PF_6_	2.136(2), 2.137(2); 2.138(3)	175.0(2), 180; 25.3(2), 0
[3–I–3]PF_6_	2.267(2)	176.77(9); 17.1(2)
[4–Ag–4]PF_6_	2.11(2), 2.11(2); 2.14(2), 2.15(2)	177.2(7), 177.2(6); 2.6(8), 4.6(7)
[4–I–4]PF_6_	2.268(3); 2.269(4)	176.8(2), 178.3(2); 18.1(2), 16.0(2)
[5–Ag–5]PF_6_	–a	–a
[5–I–5]PF_6_	2.251(5), 2.279(5)	174.6(2); 4.2(2)
[6–Ag–6]PF_6_	2.175(3)	180; 0
[6–Ag–6]BF_4_	2.236(3), 2.237(3)	161.1(2); 52.3(2)
[6–I–6]PF_6_	–a	–a

a An X-ray crystal structure could not be obtained.

Close comparison of the crystal structures of silver(i) and iodine(i) complexes of 1–5 reveals that the silver(i) and iodine(i) complexes adopt opposite conformations upon crystallization (Table 2). This means that, in these complexes, an opposing orientation of the 3-substituted pyridine rings is observed in each of the crystal structures. For example, in [1–Ag–1]PF_6_ the methyl groups are *syn* to one another and *anti* in [1–I–1]PF_6_ (Fig. 1). Similar flips are observed in previously reported complexes of both *meta*-^33,34^ and *ortho*-substituted pyridines.^31^ However, counterexamples exist in which there is no change in conformation or in which crystals obtained under different conditions exhibited different behavior. For instance, the iodine(i) complex of 3-acetaminopyridine crystallized in two polymorphs depending on temperature, one in the *syn* and the other in the *anti* conformation.^33^ In square planar copper(ii) complexes with two *meta*-substituted pyridines *trans* to each other, the *anti* conformation is clearly dominant even with simple substituents.^37^ The two additional ligands play a key role: although the DFT-optimized *syn* conformers of Cu(3)_2_Cl_2_ and Cu(3)_2_Br_2_ are slightly more stable, both complexes exist in the *anti* conformation in the solid state possibly due to intermolecular Cu⋯Cl and Cu⋯Br bonds, respectively, in addition to intramolecular C–Cl⋯Cu and C–Cl⋯H–C bonds.^38,39^ The similar *trans* square planar palladium(ii) and platinum(ii) complexes Pd(L)_2_Cl_2_ and Pt(L)_2_Cl_2_ of 1–5 all adopt the *anti* conformation in the solid state, with the halopyridines 2–5 forming C−X⋯H–C interactions, C–H⋯Cl−M hydrogen bonds, and 3–5 forming M–Cl⋯X–C halogen bonds.^40–42^

**Table 2:** Observed conformations in the crystal structures of the complexes of 1–6

L	[L–Ag–L]^+^	[L–I–L]^+^
1	*syn*	*anti*
2	*syn*	*anti*
3	*anti*	*syn*
4	*syn*	*anti*
5	*syn* a	*anti*
6	*anti* b	–c

a Preliminary X-ray crystal structure.

b PF_6_^−^ salt.

c An X-ray crystal structure could not be obtained.

**Fig. 1:**
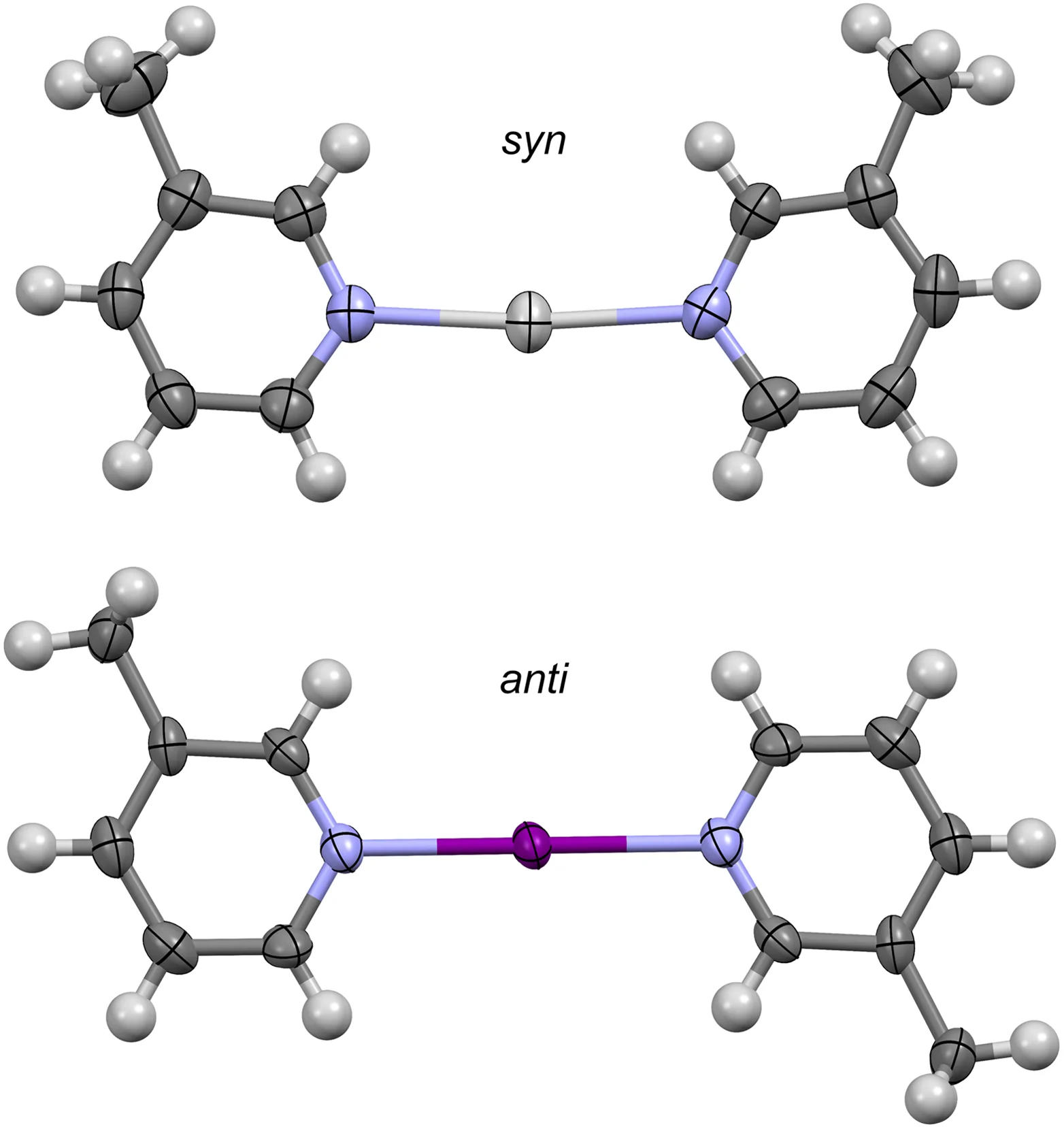
Crystal structures of [1–Ag–1]PF_6_ (top) and [1–I–1]PF_6_ (bottom) illustrating the opposite conformations. Thermal ellipsoids are drawn at 50% probability, and the PF_6_^−^ counterions have been omitted for clarity.

To further study the observed trend in conformations, the geometries of the silver(i) and iodine(i) complexes of 1–5 were DFT-optimized in both the *syn* and *anti* planar conformations at the M06-2X/def2-TZVP level of theory. In each case, the two respective conformers were found to be isoenergetic. The largest difference in energy was 1.88 kJ mol^−1^ for [1–Ag–1]PF_6_, with all others being 0.06 kJ mol^−1^ or below (Table S3). It is noteworthy that in this case, the optimized geometry of the *syn* conformer is non-planar and a fully planar minimum could not be located. These results suggest that, although for these complexes a trend of opposite conformations is observed, this is the result of packing forces in the crystals and not something inherent to the complexes themselves.

### Intermolecular interactions

The iodine(i) complexes of 1–5 exist as discrete units, with the shortest contacts C–H⋯F of approximately 2.3–2.4 Å being between the PF_6_^−^ counterion and a hydrogen atom in one of the pyridine rings (distances measured using calculated positions of the hydrogen atoms). No position is preferred: the relevant hydrogen atom is located in the methyl substituent for [1–I–1]PF_6_, in the *ortho*-position for [2–I–2]PF_6_, in the *meta*-position for [4–I–4]PF_6_ and [5–I–5]PF_6_, and in the *para*-position for [3–I–3]PF_6_. [2–I–2]PF_6_, [3–I–3]PF_6_, and [5–I–5]PF_6_ also exhibit F⋯N contacts between the counterion and the pyridinic nitrogen with lengths just below or above the sum of van der Waals (vdW) radii (ca. 2.9–3.1 Å).^43^ The belt of negative potential surrounding the iodine(i) cation hinders close contacts with the PF_6_^−^ anion. Only in the crystal structure of [5–I–5]PF_6_ is the shortest contact distance below the sum of vdW radii at 3.372(5) Å, while otherwise these range from 3.494(2) Å for [3–I–3]PF_6_ to 3.716(1) Å for [1–I–1]PF_6_.

[5–I–5]PF_6_ shows C–I⋯F interactions between the iodo-substituent and a fluorine atom in the PF_6_^−^ anion at 3.090(5) Å (I3⋯F5) and 3.171(5) Å (I2⋯F3), below the sum of vdW radii. An analogous interaction was previously observed with the iodine(i) complex of 2-iodopyridine.^31^ [4–I–4]PF_6_ exhibits similar C–Br⋯F interactions at 3.098(3) Å (Br2⋯F4) and 3.222(3) Å (Br1⋯F3). These interactions are close to linear and can thus be described as conventional halogen bonds between a neutral halogen and a Lewis base, with C–I–F angles of 167.0(2)° and 154.8(2)°, and C–Br–F angles of 163.4(2)° and 176.9(2)° (Fig. 2). Although the interaction distances are shorter than the sum of vdW radii, only the stronger C–I⋯F interactions in [5–I–5]PF_6_ are reflected in the packing of the complexes (Fig. 3). The iodine(i) complexes of 1–4 form layers allowing for offset π-stacking interactions between pyridine rings, with the shortest centroid–centroid distances ranging from 3.652(4) Å for [4–I–4]PF_6_ to 3.962(2) Å for [2–I–2]PF_6_. For [5–I–5]PF_6_, the shortest centroid–centroid distance is slightly longer at 4.062(4) Å.

**Fig. 2:**
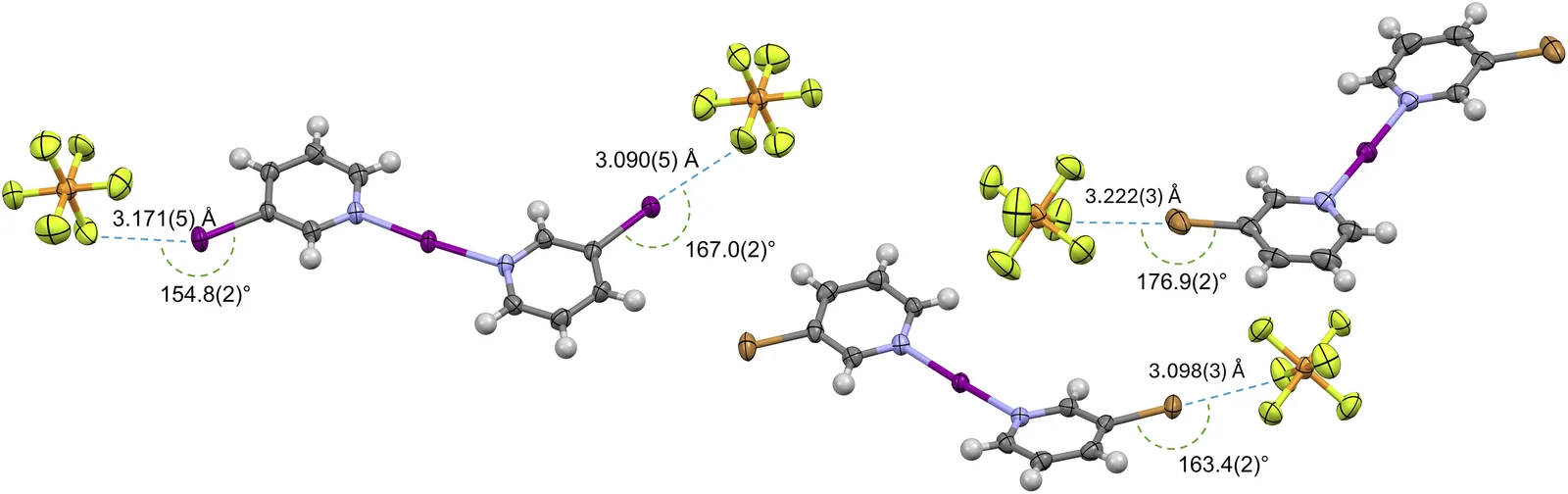
C–I⋯F interactions in the crystal structure of [5–I–5]PF_6_ (left) and C–Br⋯F interactions in the crystal structure of [4–I–4]PF_6_ (right). The iodine(i) of both [4–I–4]^+^ complexes is on a two-fold axis, leading to two identical C–Br⋯F interactions. Thermal ellipsoids are drawn at 50% probability.

**Fig. 3:**
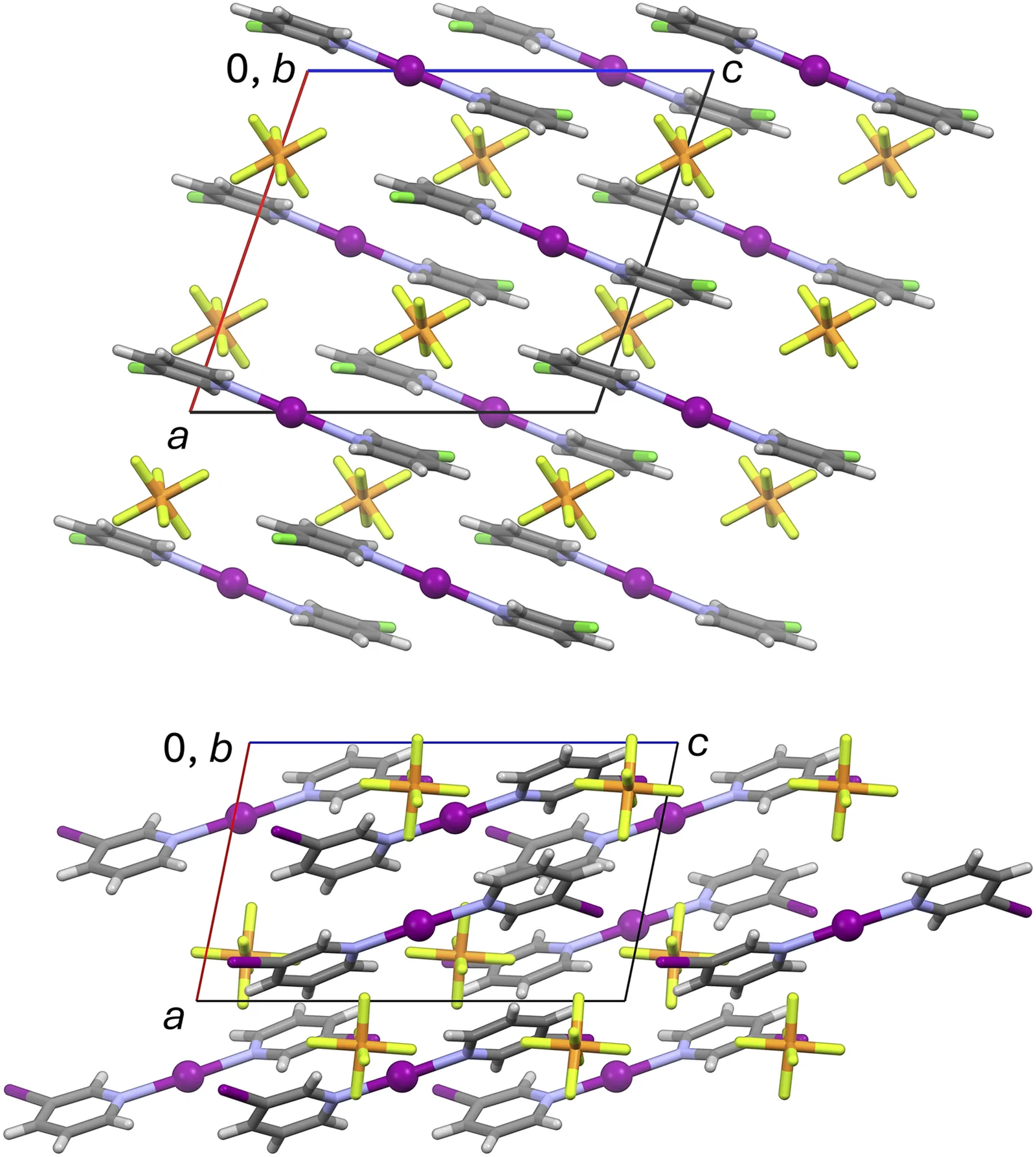
[3–I–3]^+^ units form a layered structure (top). The formation of a similar structure in [5–I–5]PF_6_ is somewhat disrupted by C–I⋯F interactions (bottom). Both crystal structures are viewed along their respective crystallographic *b* axes. Central atoms are presented as spheres.

Unlike the iodine(i) complexes which exist as discrete units, the silver(i) complexes of 1–4 are drawn together to form dimers by argentophilic interactions. Similar interactions are seen in the complexes of *para*-substituted pyridines but not in those of *ortho*-pyridines,^37^ likely due to steric effects. The closest Ag⋯Ag distance ranges from 3.355(3) Å for one of the crystallographically independent [4–Ag–4]^+^ units to 3.616(1) Å for [2–Ag–2]^+^ (Fig. 4). Both the PF_6_^−^ and BF_4_^−^ salts of [6–Ag–6]^+^ show Ag⋯I interactions instead and do not form argentophilic dimers. Additionally, cation–π interactions between the silver(i) cation and a pyridine ring of adjacent argentophilic dimers are observed in the crystal structure of [1–Ag–1]PF_6_, with a silver(i)–centroid distance of 3.394(2) Å. This is observed only for ligand 1 while none of the 3-halopyridines show similar behavior. Offset π-stacking interactions between the pyridine rings of neighboring argentophilic dimers are present in the silver(i) complexes of 2–4, and the shortest centroid–centroid distances range from 3.544(2) Å for [4–Ag–4]PF_6_ to 3.785(2) Å for [3–Ag–3]PF_6_. These interactions are also present in the structure of [6–Ag–6]BF_4_, although in this case the dimers are held together by Ag⋯I bonds instead (see below).

**Fig. 4:**
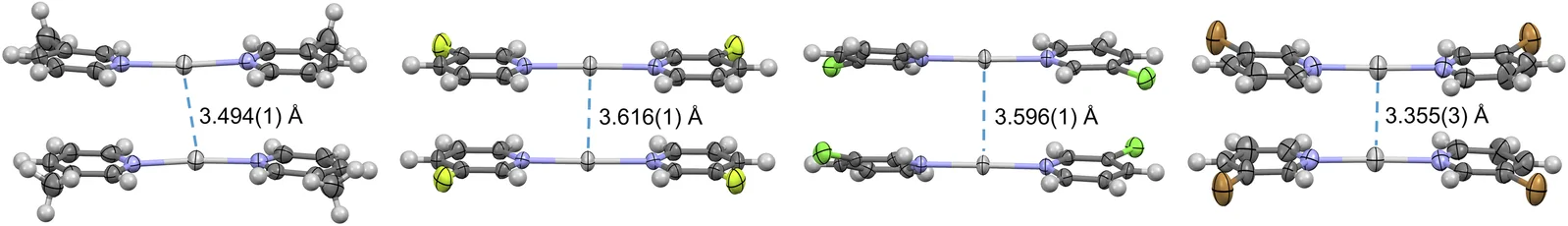
Closest Ag⋯Ag distances in the crystal structures of the silver(i) complexes of 1–4. Thermal ellipsoids are drawn at 50% probability.

A key feature highlighting the differences between silver(i) and iodine(i) complexes is the location of the counterion. Unlike the iodine(i) cation, silver(i) allows for additional coordination from one or more of the PF_6_^−^ anions. Furthermore, there is considerable charge delocalization from the iodine(i) cation to the pyridine rings, leading to a lower charge on the iodine.^7,8^ These are evident in the packing of the PF_6_^−^ counterions, resulting in short distances between the PF_6_^−^ anion and the silver(i) center: the shortest contact distance ranges from 2.65(4) Å for [4–Ag–4]PF_6_ to 2.975(3) Å for [6–Ag–6]PF_6_, all clearly below the sum of vdW radii of 3.19 Å (Fig. 5).

**Fig. 5:**
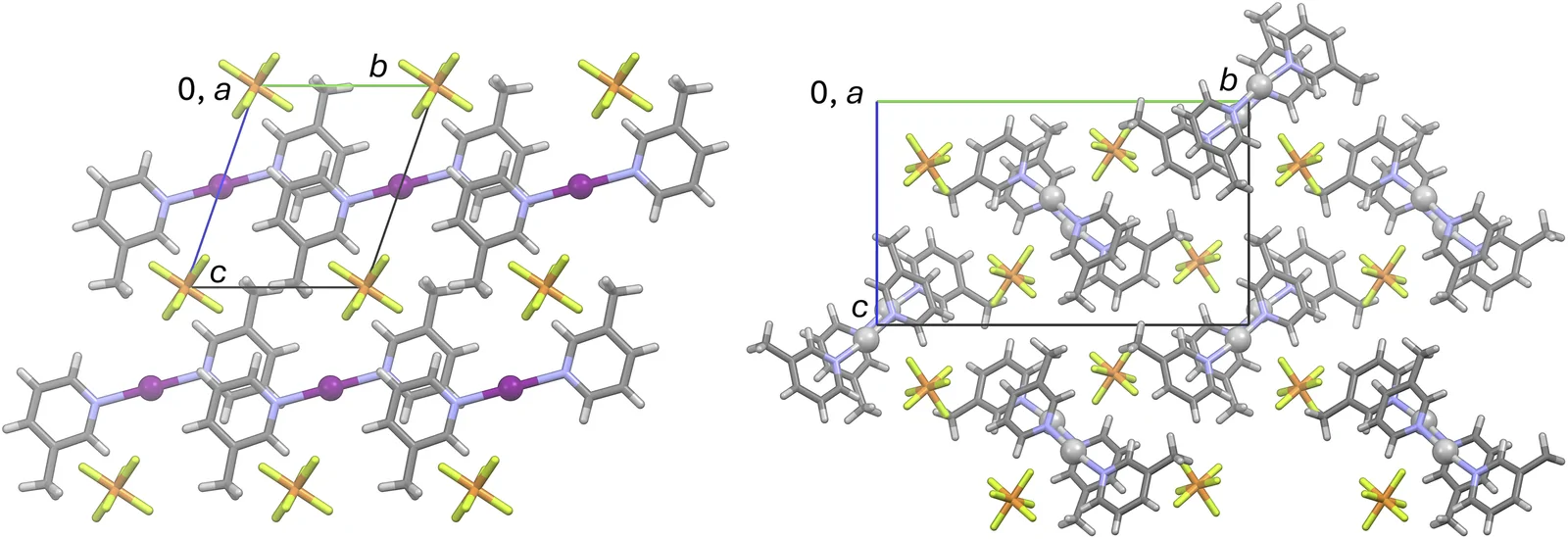
PF_6_^−^ counterions position themselves closer to the pyridines in the crystal structure of [1–I–1]PF_6_ (left), while they are close to the silver(i) cations in the crystal structure of [1–Ag–1]PF_6_ (right). Both crystal structures are viewed along their respective crystallographic *a* axes. Central atoms are presented as spheres.

Another interaction seemingly driving the intermolecular packing of the silver(i) complexes is the Ag⋯3-halo interaction which becomes more prominent with the heavier halogens. The crystal structure of [2–Ag–2]PF_6_ with 3-fluoropyridine does not exhibit this interaction, but in [3–Ag–3]PF_6_ one of the three crystallographically distinct Cl atoms (Cl2) displays a vdW contact with the neighboring silver(i) cation (Ag2), forming a Cl⋯Ag⋯Cl motif between three distinct complex cations with a Ag⋯Cl distance of 3.342(1) Å. The structure of [4–Ag–4]PF_6_ contains two crystallographically independent [4–Ag–4]^+^ units, one of which forms argentophilic dimers as described above (Ag1⋯Ag1), while the other interacts with other [4–Ag–4]^+^ units through Ag⋯Br interactions, with distances of 3.876(3) Å (Ag2⋯Br3) and 4.153(3) Å (Ag1⋯Br4) (Fig. 6), also allowing for π-stacking. Although attempts to crystallize [5–Ag–5]PF_6_ failed, packing in the crystal structures of [6–Ag–6]PF_6_ and [6–Ag–6]BF_4_, 6 having an iodo-substituent in the 3-position, is also dominated by Ag⋯3-halo interactions (see below). A detailed analysis of packing interactions using Hirshfeld surfaces^44^ is provided in the SI (Fig. S68–S71).

**Fig. 6:**
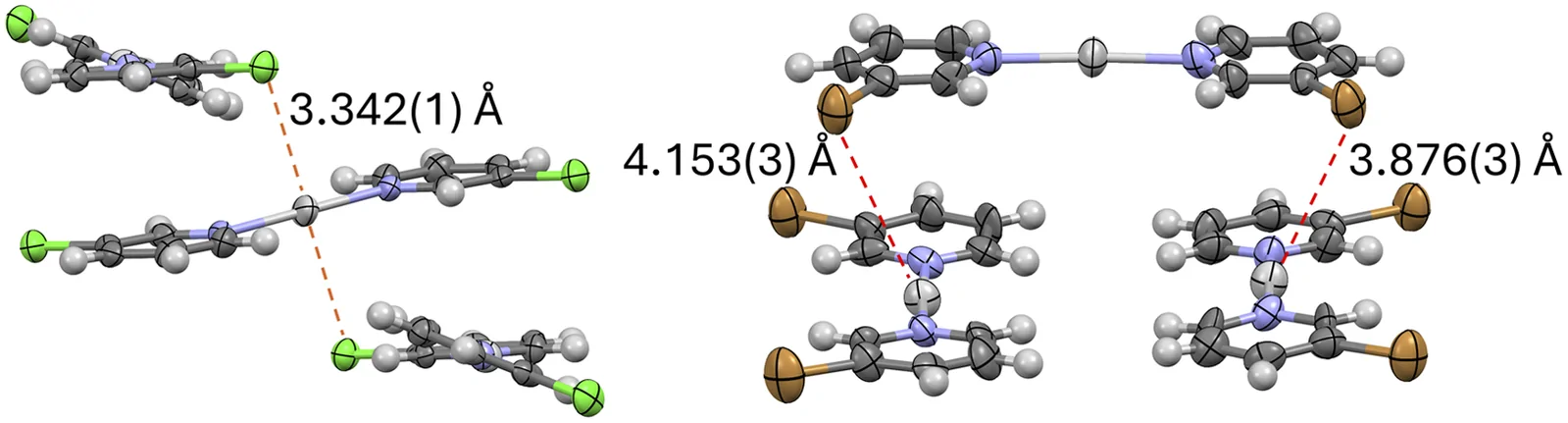
Ag⋯3-halo interactions in the crystal structures of [3–Ag–3]PF_6_ (left) and [4–Ag–4]PF_6_ (right). Only one Ag⋯Cl distance in [3–Ag–3]PF_6_ is labeled; the other is equivalent. Thermal ellipsoids are drawn at 50% probability, and the PF_6_^−^ counterions have been omitted for clarity.

The crystal structures of [2–Ag–2]PF_6_ and [3–Ag–3]PF_6_, obtained as dichloromethane solvates, show coordination from chlorine atoms in the solvent molecules to silver(i) centers, a possible driving factor for solvate formation (cf. iodine(i) complexes, none of which crystallized as solvates). [2–Ag–2]PF_6_ crystallized with one molecule of dichloromethane disordered around an inversion center for every two formula units of the complex, with the shorter Ag⋯Cl distance being 3.10(2) Å. The structure of [3–Ag–3]PF_6_ contains one molecule of dichloromethane located on a two-fold axis for every three formula units of the complex, with a Ag⋯Cl distance of 3.400(2) Å (Fig. 7).

**Fig. 7:**
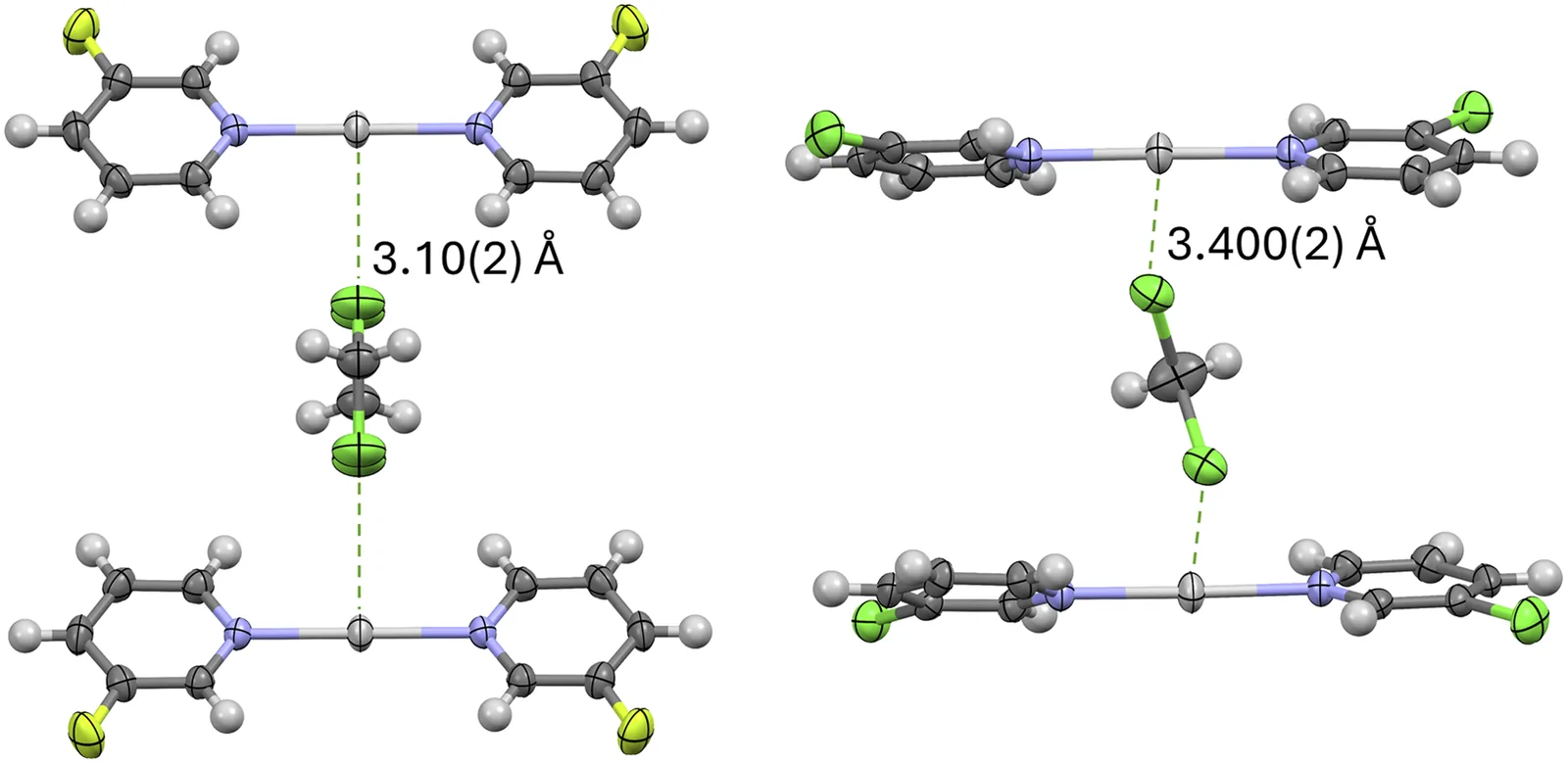
Coordination by dichloromethane molecules to silver(i) cations in the crystal structures of [2–Ag–2]PF_6_ (left) and [3–Ag–3]PF_6_ (right). Only one Ag⋯Cl distance in either structure is labeled; the other is equivalent. Thermal ellipsoids are drawn at 50% probability, and the PF_6_^−^ counterions have been omitted for clarity.

The unexpected [6–Ag–6]BF_4_ differs considerably from all the other silver(i) complexes in bond length, bond angle and dihedral angle. [6–Ag–6]BF_4_ forms dimers in which the two silver(i) complexes are joined by an interaction between the nucleophilic region of the iodo-substituent in the *meta*-position and the silver(i) cation with a C–I–Ag angle of 96.8(2)°, bending the complex away from planarity. The Ag⋯I distance is only 3.037(1) Å, significantly below the sum of vdW radii of 3.70 Å and leads to a three-coordinate T-shaped geometry around the silver(i) cation. In itself, this is not an unusual phenomenon as dimers with Ag⋯I interactions do exist in literature,^45–47^ with the PF_6_^−^ salt of the complex also showing signs of this interaction. However, this behavior seems to be dependent on the counterion: [6–Ag–6]PF_6_ does not form dimers in this way and is perfectly planar, forming step-like chains of [6–Ag–6]^+^ complexes, each connected to two other units *via* weaker Ag⋯I interactions. The BF_4_^−^ anion, due to its smaller size and higher localized negative charge, is located close to the silver(i) center of the complex: the shortest Ag⋯F distance is 2.797(3) Å, well below the sum of vdW radii. Comparable interactions between BF_4_^−^ counterions and silver(i) centers leading to bent geometries have been reported in the literature, for example with the complexes of 4-styrylpyridine^48^ and pyrazolylmethylpyridine^49^ derivatives in which the Ag⋯F interaction facilitated the formation of argentophilic interactions. In the PF_6_^−^ salt, the shortest Ag⋯F contact is somewhat longer, as noted earlier (Fig. 8). C–I⋯F interactions are present in both crystal structures, with a distance of 3.195(4) Å for the PF_6_^−^ and 3.154(4) Å for the BF_4_^−^ salt.

**Fig. 8:**
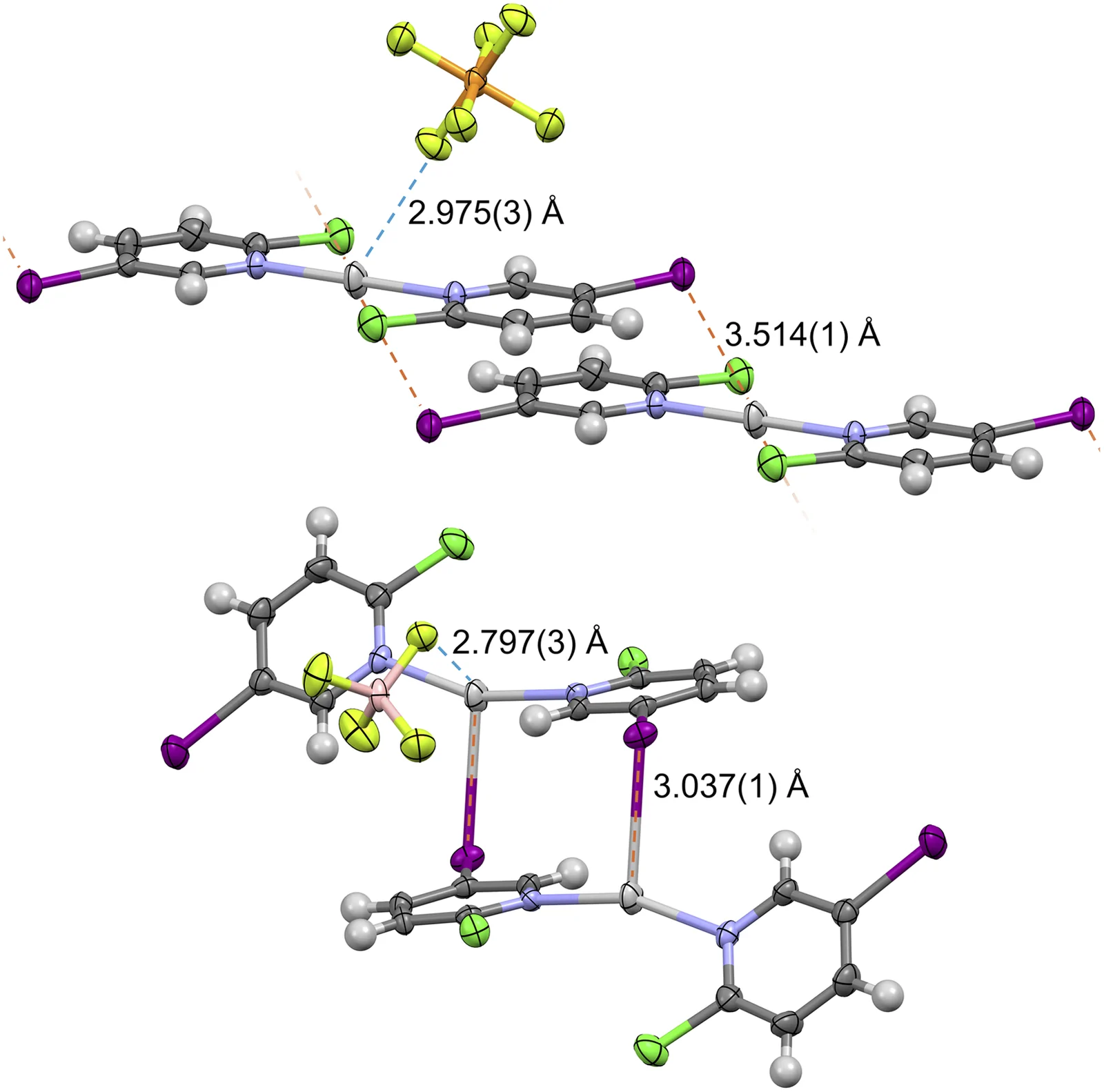
Crystal structures of [6–Ag–6]PF_6_ and [6–Ag–6]BF_4_ with Ag⋯I and Ag⋯F distances. Only one Ag⋯I distance is labeled; the other is equivalent. Thermal ellipsoids are drawn at 50% probability.

Remarkably, there was never a deliberate attempt made to synthesize the BF_4_^−^ salt of [6–Ag–6]^+^, and its formation was instead observed serendipitously when using non-commercial silver hexafluorophosphate, prepared from potassium hexafluorophosphate and silver nitrate in acetonitrile, stored in a glass vial. Initial attempts to grow crystals of [6–Ag–6]PF_6_ by vapor diffusion in a glass vial instead resulted in good-quality crystals of [6–Ag–6]BF_4_. Crystals of [6–Ag–6]PF_6_ only formed after the crystallization was repeated in a plastic microcentrifuge tube. Silver hexafluorophosphate was the silver-containing reagent in all crystallizations, suggesting that the BF_4_^−^ anions originated from etching of the borosilicate glass vials, possibly due to HF created *in situ* by the hydrolysis of the PF_6_^−^ anion.

A detailed investigation lies beyond the scope of the present work, but to study the phenomenon further, we repeated the crystallizations using commercial silver hexafluorophosphate and measured the ^19^F and ^31^P NMR spectra of both silver salts and the crystals obtained (Fig. S49–S54). Crystallizations in plastic microcentrifuge tubes yielded [6–Ag–6]PF_6_, whereas those in glass vials afforded good-quality single crystals of a new silver(i) complex of 6 in which the counterion was assigned as PO_2_F_2_^−^ (Fig. 9). The difluorophosphate anions link 6-Ag^+^ units *via* their oxygen atoms, forming a chain-like coordination polymer. The silver(i) ions adopt a distorted tetrahedral coordination geometry with three Ag–O bonds and one Ag–N bond, possibly explaining the longer Ag⋯I contacts [3.624(1) Å] and leading instead to C–I⋯O halogen bonds between neighboring chains. This observation indicates that the two unexpected anions were the result of the hydrolysis of PF_6_^−^.^50–52^

**Fig. 9:**
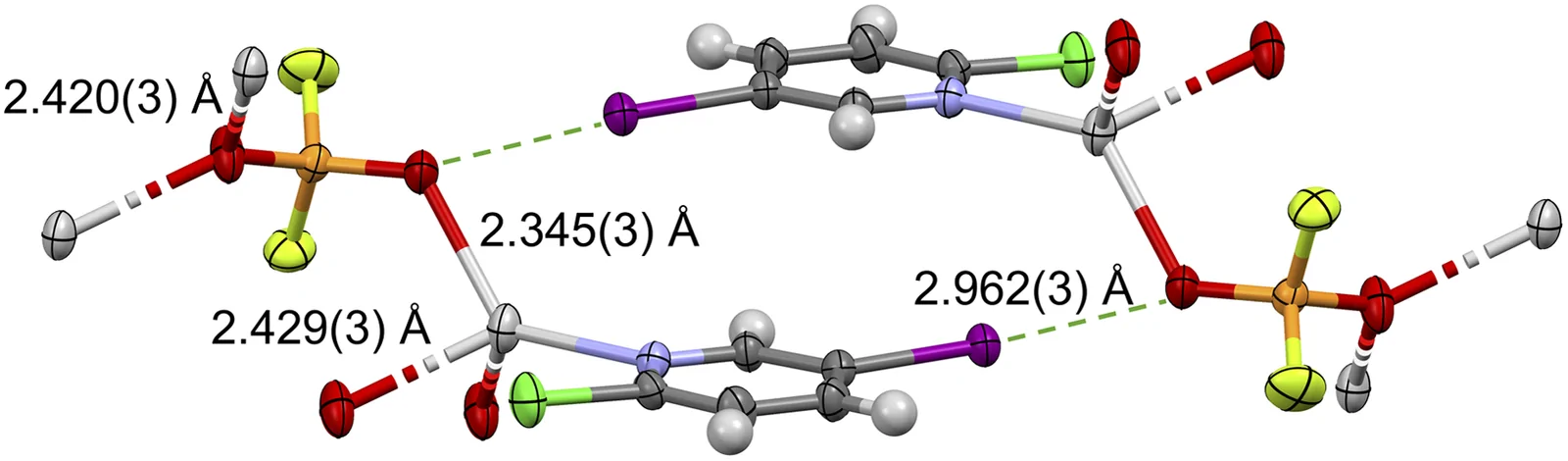
Crystal structure of 6-Ag-PO_2_F_2_ with Ag–O and C–I⋯O distances. Only one I⋯O distance is labeled; the other is equivalent. Thermal ellipsoids are drawn at 50% probability.

The ^19^F and ^31^P spectra of the non-commercial silver hexafluorophosphate both show signals corresponding to multiple species. In the ^19^F NMR spectrum, the strongest signals are a doublet from PF_6_^−^ at −73.8 (*J*_FP_ = 705.8 Hz) ppm, a doublet from PO_2_F_2_^−^ at −83.7 (*J*_FP_ = 950.9 Hz) ppm and two singlets from BF_4_^−^, with the ^11^B singlet at −152.7 ppm. Two weaker signals are present, a doublet likely from PO_3_F^2−^ at −76.1 (*J*_FP_ = 904.8 Hz) ppm, and a singlet at −139.2 ppm possibly from a silicon species, presumably SiF_4_, SiF_5_^−^, or SiF_6_^2−^. The ^31^P NMR spectrum shows three multiplets: a doublet from PO_3_F^2−^ at −6.1 (*J*_PF_ = 905.6 Hz) ppm, a triplet from PO_2_F_2_^−^ at −15.8 (*J*_PF_ = 950.6) ppm, and a heptet from PF_6_^−^ at −145.5 (*J*_PF_ = 706.2) ppm. In contrast, the ^19^F and ^31^P NMR spectra of the commercial silver hexafluorophosphate show much weaker yet identifiable signals from PO_2_F_2_^−^ in addition to those from PF_6_^−^. No signals from silicon species or BF_4_^−^ are present as the salt was stored in a plastic container. The ^19^F and ^31^P NMR spectra of crystals prepared using the commercial silver hexafluorophosphate dissolved in CD_3_CN show near-complete hydrolysis of PF_6_^−^. In the ^19^F NMR spectrum, signals corresponding to PF_6_^−^, PO_2_F_2_^−^, a silicon species, and BF_4_^−^ are observed. However, in the ^31^P NMR spectrum no heptet from PF_6_^−^ is present, likely owing to its low concentration (ca. 1% of PO_2_F_2_^−^). Instead, in addition to a triplet from PO_2_F_2_^−^, a weak singlet at 0.2 ppm is observed, which possibly arises from the total hydrolysis of PF_6_^−^.

Although only observed for 6, these findings are a reminder that PF_6_^−^ may undergo significant hydrolysis under benchtop conditions and demonstrate that a mixture of crystallographically similar phosphorus fluoride and oxyfluoride species can be present.^53–55^ Consequently, based on the crystallographic and spectroscopic evidence presented here, the three crystal structures deposited in the CSD reported to contain tetrahedral PF_4_^−^ anions (SAGROL,^56^ VADSOM,^57^ and XOVLEA^58^) merit further investigation and may possibly be re-evaluated as PO_2_F_2_^−^ salts.

### Powder X-ray diffraction

To complement the structural analysis, PXRD was used to examine powdered samples and assess their structural consistency with the corresponding single-crystal structures. These samples were prepared by rapid precipitation upon addition of an antisolvent (pentane or diethyl ether) to dichloromethane solutions of the silver(i) and iodine(i) complexes. The measured diffraction patterns were compared against patterns simulated based on the X-ray crystal structures, and Pawley fitting was done to patterns with apparent resemblance (Fig. S72–S82 and Table S2 for refined lattice parameters). While most bulk materials show patterns that match the respective simulated data, Pawley fitting was not performed to [1–I–1]PF_6_ or [2–Ag–2]PF_6_ since their experimental and simulated patterns differed significantly. The PXRD pattern of [1–I–1]PF_6_ provides strong evidence for the formation of a different polymorph compared to the obtained crystal structure, although the degradation of the iodine(i) complex is also possible. The sample containing [6–Ag–6]^+^ consisted mostly of [6–Ag–6]PF_6_ and 6-Ag-PO_2_F_2_, as peaks corresponding to the simulated pattern of [6–Ag–6]BF_4_ were not observed in the PXRD pattern. The crystal structures of [2–Ag–2]PF_6_ and [3–Ag–3]PF_6_ were obtained as dichloromethane solvates, and clear changes over the four scans of the measurement were observed (Fig. S83 and S84). This is likely a result of desolvation since in both cases the first scan shows the closest match to the simulated pattern. [3–Ag–3]PF_6_ was fitted despite a secondary pattern being clearly observable. Overall, the crystal structures show a good match to the bulk material which suggests that the complexes do not exist as mixtures of polymorphs. Consequently, the opposite conformations do not appear to exist in the bulk even under rapid crystallization which is expected to increase the possibility of crystallization of metastable polymorphs.

### NMR spectroscopy

Complexation was confirmed by ^1^H and ^1^H–^15^N HMBC NMR spectroscopy, with spectra acquired in CD_2_Cl_2_ (Fig. S1–S48 and Table S1). All complexes exhibit considerable changes in ^15^N NMR chemical shifts *δ*(^15^N) relative to the pure pyridines (Table 3), consistent with previous reports.^31,34,59,60^ The chemical shifts of the halopyridines 2–5 are close to identical, differing by less than 3 ppm, and show no dependence on the electronegativity of the halo-substituent. This contrasts with observations of *ortho*-substituted pyridines in acetone-*d*_6_ in which the *δ*(^15^N_lig_) values shifted upfield as the electronegativity of the halogen increased.^31^ The ^15^N NMR chemical shifts of pyridines are known to vary slightly depending on the solvent: differences of at most 2 ppm in the *δ*(^15^N) of some *meta*-substituted pyridines in CD_2_Cl_2_ and CD_3_CN have been reported,^34^ whereas differences of around 5–7 ppm with *para-*substituted pyridines were found in CDCl_3_ and CD_3_CN.^61^ The larger separation between the *meta*-substituents and the pyridinic nitrogen atom should nevertheless be expected to reduce the impact on chemical shifts.

**Table 3:** ^15^N NMR chemical shifts (*δ* in ppm) of and Δ*δ*(^15^N) of their silver(i) and iodine(i) complexes, and protonated pyridines [LH]^+^ in CD_2_Cl_2_

L	*δ*(^15^N_lig_)	Δ*δ*(^15^N_Ag_)	Δ*δ*(^15^N_I_)	Δ*δ*(^15^N_H_)
1	−67.2	−55.2	−107.1	−84.1
2	−58.3	−62.0	−115.2	−50.0
3	−61.1	−60.1	−111.8	−49.0
4	−60.3	−52.4	−111.7	−44.4
5	−59.5	–a	−111.6	−55.9
6	−67.8	−32.4	–a	−24.7

a No signals were observed at 298 K.

Coordination shifts for both the silver(i) and iodine(i) complexes are in line with previously reported Δ*δ*(^15^N) values in CD_2_Cl_2_.^15,34,59,60,62^ No ^15^N NMR signals were observed for [5–Ag–5]^+^ as the complex is virtually insoluble in CD_2_Cl_2_. The measurement was not repeated in a different solvent to maintain consistency with the rest of the chemical shifts. Differences in Δ*δ*(^15^N_Ag_) and Δ*δ*(^15^N_I_) between the pyridines remain relatively small, except for [6–Ag–6]^+^, with Δ*δ*(^15^N_Ag_) otherwise ranging from −55.2 ppm for [1–Ag–1]^+^ to −62.0 ppm for [2–Ag–2]^+^, and Δ*δ*(^15^N_I_) from −107.1 ppm for [1–I–1]^+^ to −115.2 ppm for [2–I–2]^+^. The *δ*(^15^N_Ag_) values and thus the coordination shifts Δ*δ*(^15^N_Ag_) are sensitive to the choice of solvent because the silver(i) ion allows for coordination by solvent molecules. This effect is more pronounced in strongly coordinating solvents such as CD_3_CN where there is a stronger dependence of *δ*(^15^N_Ag_) and thus Δ*δ*(^15^N_Ag_) on the electron density in the pyridine.^33,56,61^ This may be a factor in the narrow range observed for Δ*δ*(^15^N_Ag_), as CD_2_Cl_2_ is not expected to strongly interact with the silver(i) ions. *δ*(^15^N_I_), on the other hand, are not influenced by solvent,^12,56^ with Δ*δ*(^15^N_I_) values of roughly −110 ppm being characteristic of [bis(pyridine)iodine(i)]^+^ complexes.^4^ Additionally, as noted by Rissanen and Ward,^34^ resonance limits the impact of substituents in the *meta*-position.

The Δ*δ*(^15^N_H_) of the protonated pyridines differ from both Δ*δ*(^15^N_Ag_) and Δ*δ*(^15^N_I_), confirming the existence of the silver(i) and iodine(i) complexes. The four halopyridines 2–5 show Δ*δ*(^15^N_H_) of between −44.4 ppm for [4H]^+^ and −55.9 ppm for [5H]^+^. 6 shows the smallest change with a Δ*δ*(^15^N_H_) of −24.7 ppm. In comparison, the Δ*δ*(^15^N_H_) values of their *ortho*-substituted counterparts were reported to all be close to 0 ppm in acetone-*d*_6_.^31^ The Δ*δ*(^15^N_H_) of the methylpyridine 1 is −84.1 ppm, closer to its *ortho*-isomer with a Δ*δ*(^15^N_H_) value of −100.6 ppm.^31^ Measurements on the *para*-isomer and its complexes in CD_2_Cl_2_ showed similar behavior, with a Δ*δ*(^15^N_H_) value of −98.6 ppm.^59^1 having the smallest change in *δ*(^15^N_H_) of its isomers supports the interpretation that resonance diminishes the effect of substituents in the *meta*-position. This can also be seen using 6 as an *ortho*-substituted benchmark: while it has an iodo-substituent in the *meta*-position like 5, its Δ*δ*(^15^N_H_) is dominated by the chloro-substituent in the *ortho*-position. This decreases the nucleophilicity of the pyridinic nitrogen, presumably explaining why no ^15^N NMR signals were observed for the complex [6–I–6]^+^.

## Conclusions

The resonance-disfavored *meta*-position diminishes the impact of substituents on the Lewis basicity of pyridines and thus the halogen bonds of [bis(pyridine)iodine(i)]^+^ complexes, with little difference in ^15^N shifts or I–N bond lengths, which occupy a markedly narrow range of 0.028 Å. The silver(i) and iodine(i) complexes of *meta*-substituted pyridines 1–5 consistently crystallized in opposite conformations. Albeit an interesting observation, there appears to be no evidence for this trend to be anything more than a coincidence and a result of crystal packing interactions. Further investigation with various linear complexes under different crystallization conditions could help determine the origins of this phenomenon. These findings suggest that substituents in the *meta*-position can be exchanged with little effect on the strength of the halogen bond in [bis(pyridine)iodine(i)]^+^ complexes, enabling independent fine-tuning of solubility and intermolecular interactions, for example by harnessing hydrogen bonding *via* substituents in hierarchical architectures. Insights gained from this work are anticipated to aid in the design of motifs for supramolecular architectures, such as XOFs.

## Experimental section

### Materials and methods

Dichloromethane and acetonitrile were dried and stored over 3 Å molecular sieves under a nitrogen atmosphere prior to use. Silver hexafluorophosphate was prepared according to a published procedure and used in crystallizations,^63^ whereas a commercial salt was utilized in NMR studies. All other reagents and solvents were purchased from commercial suppliers and used as received.

Single-crystal X-ray diffraction data were collected on a Bruker–Nonius Kappa APEX II diffractometer using Mo K_α_ radiation (*λ* = 0.71073 Å) at 170 K (200 K for [1–Ag–1]PF_6_ and 6-Ag-PO_2_F_2_) using COLLECT,^64^ DENZO, and SCALEPACK,^65^ while the absorption correction was applied using SADABS.^66^ Crystal structures were solved and refined using the SHELXT,^67^ SHELXS,^68^ and SHELXL^69^ programs in Olex2.^70^ Crystallographic data are presented in Table 4.

Selected crystallographic parameters and structure refinement data for the silver(i) complexes of 1–4 and 6 and iodine(i) complexes of 1–5

**Table:** 

Complex	[1–Ag–1]PF_6_	[2–Ag–2]PF_6_	[3–Ag–3]PF_6_	[4–Ag–4]PF_6_	[6–Ag–6]PF_6_	[6–Ag–6]BF_4_
Empirical formula	C_12_H_14_AgF_6_N_2_P	C_10.5_H_9_AgClF_8_N_2_P	C_10.3_H_8.7_AgCl_2.7_F_6_N_2_P	C_10_H_8_AgBr_2_F_6_N_2_P	C_10_H_6_N_2_Cl_2_I_2_AgPF_6_	C_10_H_6_AgBCl_2_F_4_I_2_N_2_
Formula weight/g mol^−1^	439.09	489.49	508.23	568.84	731.71	673.55
Temperature/K	170(1)	170(1)	170(1)	170(1)	170(1)	170(1)
Crystal system	Monoclinic	Monoclinic	Monoclinic	Tetragonal	Triclinic	Triclinic
Space group	*P*2_1_/*n*	*C*2/*m*	*C*2/*c*	*P*4_3_2_1_2	*P*1̄	P1̄
*a*/Å	8.2694(2)	13.9337(5)	10.7468(2)	21.3063(4)	7.7679(2)	7.27770(10)
*b*/Å	17.7293(3)	16.3478(5)	28.3299(4)	21.3063(4)	7.9821(2)	8.2872(2)
*c*/Å	10.9625(2)	7.9912(2)	16.4019(2)	13.9878(4)	8.5646(2)	14.3961(3)
*α*/°	90	90	90	90	78.703(2)	73.9460(10)
*β*/°	103.6310(10)	121.808(2)	105.3710(10)	90	65.161(2)	84.8700(10)
*γ*/°	90	90	90	90	66.999(2)	82.5010(10)
V Å^−3^	1561.95(5)	1546.91(9)	4815.03(13)	6349.9(3)	443.28(2)	825.96(3)
Z	4	4	12	16	1	2
µ mm^−1^	1.451	1.662	1.857	6.460	5.065	5.314
F(000)	864.0	948.0	2952.0	4288.0	336.0	616.0
Crystal size/mm^3^	0.2 × 0.2 × 0.2	0.1 × 0.1 × 0.1	0.2 × 0.2 × 0.2	0.2 × 0.2 × 0.2	0.3 × 0.1 × 0.05	0.2 × 0.1 × 0.1
2*θ* range/°	5.566 to 55.038	5.682 to 55.044	4.186 to 54.986	5.172 to 52.05	5.244 to 54.986	5.146 to 55.064
Reflns collected	30 028	8513	22 016	32 818	5672	12 468
Independent reflns	3592	1829	5503	6203	2021	3789
Data/restraints/params	3592/0/201	1829/2/118	5503/0/317	6203/175/435	2021/0/112	3789/0/199
Goodness-of-fit^a^ on *F*^2^	1.155	1.111	1.104	1.100	1.080	1.136
*R*_int_	0.0457	0.0264	0.0324	0.0902	0.0285	0.0295
Final *R* indices^b^ [I>2σ(*I*)]; *R*_1_, w*R*_2_	0.0446, 0.0699	0.0359, 0.0729	0.0409, 0.0693	0.0765, 0.1647	0.0301, 0.0555	0.0329, 0.0567
Final *R* indices [all data]; *R*_1_, w*R*_2_	0.0617, 0.0762	0.0404, 0.0757	0.0521, 0.0734	0.1373, 0.1977	0.0351, 0.0578	0.0387, 0.0591
Largest diff. peak/hole/e Å^−3^	0.92/–0.63	0.72/–1.02	0.57/–0.43	2.79/–0.69	0.87/–0.86	0.66/–0.88
						

a
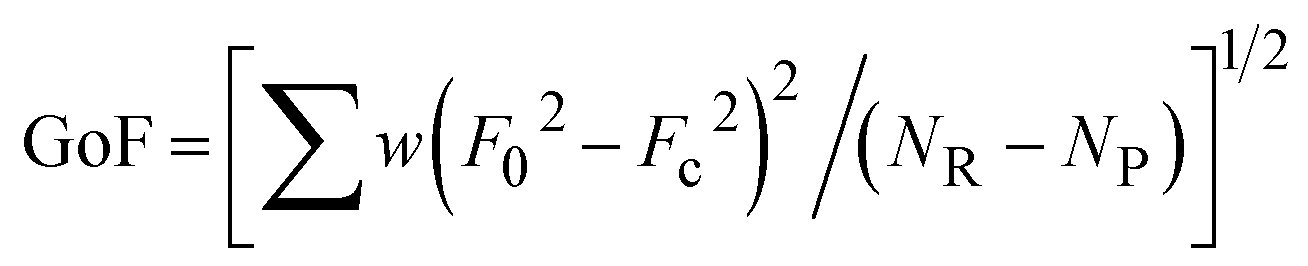
.

b
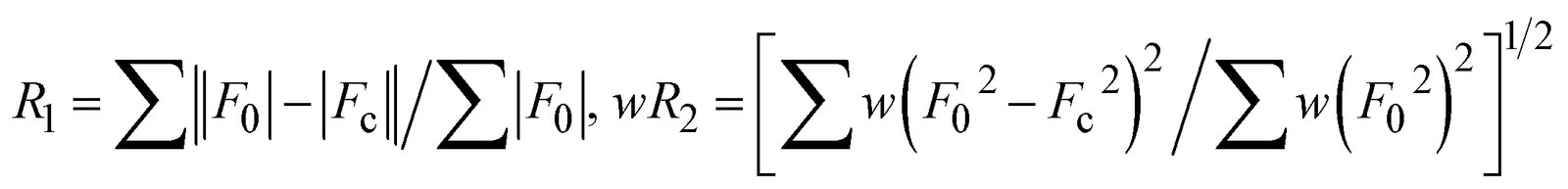
.

c *R*_int_ prior to twin refinement.

**Table:** 

Complex	[1–I–1]PF_6_	[2–I–2]PF_6_	[3–I–3]PF_6_	[4–I–4]PF_6_	[5–I–5]PF_6_	6-Ag-PO_2_F_2_
Empirical formula	C_12_H_14_F_6_IN_2_P	C_10_H_8_F_8_IN_2_P	C_10_H_8_Cl_2_F_6_IN_2_P	C_10_H_8_Br_2_F_6_IN_2_P	C_10_H_8_F_6_I_3_N_2_P	C_5_H_3_AgClF_2_INO_2_P
Formula weight/g mol^−1^	458.12	466.05	498.95	587.87	681.85	448.27
Temperature/K	170(1)	170(1)	170(1)	170(1)	170(1)	200(1)
Crystal system	Triclinic	Monoclinic	Monoclinic	Orthorhombic	Monoclinic	Triclinic
Space group	*P*1̄	*C*2/*c*	*C*2/*c*	*Ibca*	*P*2_1_/*c*	*P*1̄
*a*/Å	7.0041(4)	11.8378(2)	11.6914(2)	15.9398(2)	8.34210(10)	5.08620(10)
*b*/Å	7.8666(4)	8.64570(10)	10.6389(2)	16.0917(2)	15.2705(3)	10.0050(2)
*c*/Å	8.5083(5)	14.7323(2)	13.1149(3)	25.4136(4)	13.5415(2)	10.7362(3)
*α*/°	104.859(3)	90	90	90	90	102.1000(10)
*β*/°	95.412(3)	102.8030(10)	109.0260(10)	90	101.5861(9)	99.0110(10)
*γ*/°	114.912(3)	90	90	90	90	98.5990(10)
V Å^−3^	399.86(4)	1470.31(4)	1542.16(5)	6518.55(15)	1689.88(5)	518.12(2)
Z	1	4	4	16	4	2
µ mm^−1^	2.159	2.370	2.584	7.022	5.697	5.337
*F*(000)	222.0	888.0	952.0	4384.0	1240.0	412.0
Crystal size/mm^3^	0.2 × 0.2 × 0.2	0.3 × 0.2 × 0.2	0.2 × 0.2 × 0.2	0.2 × 0.1 × 0.1	0.2 × 0.2 × 0.1	0.14 × 0.08 × 0.06
2*θ* range/°	5.09 to 50.196	5.672 to 55.024	5.314 to 54.964	3.596 to 53.998	4.068 to 54.984	5.062 to 57.334
Reflns collected	1366	13 328	8160	53 352	30 595	8563
Independent reflns	1366	1695	1763	3570	3877	2203
Data/restraints/params	1366/0/105	1695/15/131	1763/0/103	3570/0/202	3877/0/199	2203/0/128
Goodness-of-fit on *F*^2^	1.087	1.086	1.120	1.028	1.108	1.119
*R*_int_	0.0569^c^	0.0311	0.0285	0.0806	0.0488	0.0403
Final *R* indexes [I>2σ(I)]; *R*_1_, w*R*_2_	0.0701, 0.1818	0.0195, 0.0364	0.0212, 0.0432	0.0339, 0.0563	0.0449, 0.0790	0.0292, 0.0547
Final *R* indexes [all data]; *R*_1_, w*R*_2_	0.0735, 0.1870	0.0234, 0.0379	0.0228, 0.0439	0.0537, 0.0617	0.0538, 0.0830	0.0359, 0.0572
Largest diff. peak/hole/e Å^−3^	3.04/−1.59	0.30/−0.29	0.30/−0.34	0.57/–0.45	1.29/–0.99	0.75/–1.09

[1–I–1]PF_6_ was refined as a two component non-merohedral rotational twin (twin law [1.005 0.010 0.158/−1.003–1.005 –0.079/0.000 0.000–1.000]) with refined BASF 0.465 using HKLF5 refinement. The corresponding *R*_int_ value is reported prior to the twin refinement. This improved the refinement, but significant maxima of positive residual electron density still exist at a close proximity (ca. 1.1 Å) to the iodine atom. A similar issue was observed in the refinement of [4–Ag–4]PF_6_. In this case the most significant positive residual electron density is located between the pyridines of two [4–Ag–4]^+^ units with a short distance (ca. 1.7 Å) to the C–H hydrogen atoms, suggesting that this does not correspond to an unlocated atom in the refinement. Additionally, [4–Ag–4]PF_6_ was refined in the non-centrosymmetric space group *P*4_3_2_1_2. The refined Flack parameter deviated significantly from zero [0.42(4)], and therefore the structure was refined as a partial inversion twin with BASF 0.418. One of the pyridine rings was treated with rigid bond restraint (RIGU) due to inconsistent anisotropic displacement parameters (ADPs) along the C–C bonds. One of the two PF_6_^−^ anions was modelled as disordered and the ADPs of the disordered fluorine atoms were restrained.

Powder X-ray diffraction (PXRD) data were collected with a Panalytical Aeris diffractometer using Cu K_α1,α2_ radiation (*λ*_α1_ = 1.5406, *λ*_α2_ = 1.5444 Å) using the Bragg–Brentano geometry with a fixed incident beam slit of 1/8° and 13 mm mask. Powder samples were prepared onto a zero-background silicon disc, and data collection was done in the 2*θ* range of 2.5–60°. Pawley fitting of the PXRD data was carried out using the Malvern Panalytical HighScore Plus v4.9 software.^71^

NMR spectra were recorded on Bruker Avance III 600 and Avance III 500 spectrometers in CD_2_Cl_2_ (^1^H and ^1^H–^15^N HMBC) or CD_3_CN (^19^F and ^31^P) at 298 K. Residual solvent signals were used as the internal standard for the ^1^H spectra. For the ^1^H–^15^N HMBC spectra, an external reference in the form of a sealed capillary filled with a mixture of CD_2_Cl_2_ and nitromethane was used. Chemical shifts in ^15^N were corrected to correspond to a pure nitromethane standard (*δ*_N_ 0.0 ppm) by a ^1^H–^15^N HMBC measurement of nitromethane with the internal standard (*δ*_N_ −4.2 ppm). For the ^19^F and ^31^P NMR spectra, absolute referencing *via* the Ξ ratio was used.

### Synthetic procedures

Iodine(i) complexes were obtained by the conventional two-step synthetic method involving the analogous silver(i) complexes. [Bis(pyridine)silver(i)]^+^ complexes [L–Ag–L]^+^ were prepared by reacting silver hexafluorophosphate and substituted pyridines 1–6 in a 1 : 2 ratio in dichloromethane, with yields of 80–95%. These could be converted into [bis(pyridine)iodine(i)]^+^ complexes [L–I–L]^+^ by the addition of a small excess of iodine into the solution, with yields of 60–83%. The addition of a small excess of iodine into a solution of a [bis(pyridine)silver(i)]^+^ complex colored the solution purple and resulted in the formation of a silver iodide precipitate, which was removed by centrifugation. Spectroscopic samples of the complexes were prepared by this general method in CD_2_Cl_2_. For the trifluoroacetate salts [LH]TFA, a small excess of trifluoroacetic acid was added to the solution of L in CD_2_Cl_2_. Full details are given in the (SI).

#### 3-Methylpyridine (1)

^1^H NMR (500 MHz, CD_2_Cl_2_) *δ* 8.42 (s, 1H, 2), 8.37 (d, *J* = 4.8 Hz, 1H, 6), 7.49 (d, *J* = 7.7 Hz, 1H, 4), 7.17 (dd, *J* = 7.7, 4.8 Hz, 1H, 5), 2.32 (s, 2H, –CH3); ^15^N NMR (51 MHz, CD_2_Cl_2_) *δ* −67.2.

#### [Bis(3-methylpyridine)silver(i)]^+^ ([1–Ag–1]^+^)

^1^H NMR (600 MHz, CD_2_Cl_2_) *δ* 8.47 (d, *J* = 2.2 Hz, 2H, 2), 8.42 (d, *J* = 5.3 Hz, 2H, 6), 7.78 (dd, *J* = 8.0, 2.2 Hz, 2H, 4), 7.45 (dd, *J* = 7.9, 5.2 Hz, 2H, 5), 2.40 (s, 6H, –CH3); ^15^N NMR (61 MHz, CD_2_Cl_2_) *δ* −122.4.

#### [Bis(3-methylpyridine)iodine(i)]^+^ ([1–I–1]^+^)

^1^H NMR (600 MHz, CD_2_Cl_2_) *δ* 8.57 (s, 2H, 2), 8.55 (d, *J* = 5.5 Hz, 2H, 6), 8.00 (d, *J* = 7.9 Hz, 2H, 4), 7.49 (dd, *J* = 7.9, 5.5 Hz, 2H, 5), 2.46 (s, 6H, –CH3); ^15^N NMR (61 MHz, CD_2_Cl_2_) *δ* −174.3.

#### 3-Methylpyridin-1-ium ([1H]^+^)

^1^H NMR (500 MHz, CD_2_Cl_2_) *δ* 8.64 (s, 1H, 2), 8.63 (d, *J* = 5.5 Hz, 1H, 6), 8.12 (d, *J* = 8.0 Hz, 1H, 4), 7.73 (dd, *J* = 8.0, 5.5 Hz, 1H, 5), 2.52 (s, 1H, –CH3); ^15^N NMR (51 MHz, CD_2_Cl_2_) *δ* −151.3.

#### 3-Fluoropyridine (2)

^1^H NMR (600 MHz, CD_2_Cl_2_) *δ* 8.46 (d, *J* = 2.9 Hz, 1H, 2), 8.41 (dt, *J* = 4.7, 1.6 Hz, 1H, 6), 7.42 (tdd, *J* = 8.6, 2.9, 1.3 Hz, 1H, 4), 7.32 (dtd, *J* = 8.5, 4.8, 0.7 Hz, 1H, 5); ^15^N NMR (61 MHz, CD_2_Cl_2_) *δ* −58.3.

#### [Bis(3-fluoropyridine)silver(i)]^+^ ([2–Ag–2]^+^)

^1^H NMR (600 MHz, CD_2_Cl_2_) *δ* 8.60 (t, *J* = 2.4 Hz, 2H, 2), 8.54 (dt, *J* = 5.2, 1.0 Hz, 2H, 6), 7.78 (tdd, *J* = 8.4, 2.9, 1.2 Hz, 2H, 4), 7.66 (dt, *J* = 8.7, 5.2 Hz, 2H, 5); ^15^N NMR (61 MHz, CD_2_Cl_2_) *δ* −120.3.

#### [Bis(3-fluoropyridine)iodine(i)]^+^ ([2–I–2]^+^)

^1^H NMR (600 MHz, CD_2_Cl_2_) *δ* 8.71 (d, *J* = 2.7 Hz, 2H, 2), 8.70 (d, *J* = 4.5 Hz, 2H, 6), 8.00 (td, *J* = 7.9, 2.1 Hz, 2H, 4), 7.69 (dt, *J* = 8.6, 5.2 Hz, 2H, 5); ^15^N NMR (61 MHz, CD_2_Cl_2_) *δ* −173.5.

#### 3-Fluoropyridin-1-ium ([2H]^+^)

^1^H NMR (600 MHz, CD_2_Cl_2_) *δ* 8.67 (s, 1H, 2), 8.60 (d, *J* = 4.3 Hz, 1H, 6), 7.85 (t, *J* = 8.6 Hz, 1H, 4), 7.70 (dt, *J* = 8.8, 4.6 Hz, 1H, 5); ^15^N NMR (61 MHz, CD_2_Cl_2_) *δ* −108.3.

#### 3-Chloropyridine (3)

^1^H NMR (500 MHz, CD_2_Cl_2_) *δ* 8.56 (dd, *J* = 2.5, 0.8 Hz, 1H, 2), 8.47 (dd, *J* = 4.7, 1.5 Hz, 1H, 6), 7.69 (ddd, *J* = 8.2, 2.5, 1.4 Hz, 1H, 4), 7.26 (ddd, *J* = 8.2, 4.8, 0.8 Hz, 1H, 5); ^15^N NMR (51 MHz, CD_2_Cl_2_) *δ* −61.1.

#### [Bis(3-chloropyridine)silver(i)]^+^ ([3–Ag–3]^+^)

^1^H NMR (600 MHz, CD_2_Cl_2_) *δ* 8.67 (d, *J* = 2.3 Hz, 2H, 2), 8.60 (dd, *J* = 5.3, 1.4 Hz, 2H, 6), 8.00 (ddd, *J* = 8.4, 2.4, 1.3 Hz, 2H, 4), 7.59 (dd, *J* = 8.4, 5.3 Hz, 2H, 5); ^15^N NMR (61 MHz, CD_2_Cl_2_) *δ* −121.2.

#### [Bis(3-chloropyridine)iodine(i)]^+^ ([3–I–3]^+^)

^1^H NMR (600 MHz, CD_2_Cl_2_) *δ* 8.76 (d, *J* = 2.2 Hz, 2H, 2), 8.72 (dd, *J* = 5.5, 1.2 Hz, 2H, 6), 8.21 (ddd, *J* = 8.4, 2.2, 1.2 Hz, 2H, 4), 7.63 (dd, *J* = 8.4, 5.5 Hz, 2H, 5); ^15^N NMR (61 MHz, CD_2_Cl_2_) *δ* −172.9.

#### 3-Chloropyridin-1-ium ([3H]^+^)

^1^H NMR (600 MHz, CD_2_Cl_2_) *δ* 8.72 (dd, *J* = 2.4, 0.7 Hz, 1H, 2), 8.62 (dd, *J* = 5.2, 1.4 Hz, 1H, 6), 8.06 (ddd, *J* = 8.3, 2.4, 1.4 Hz, 1H, 4), 7.60 (ddd, *J* = 8.3, 5.2, 0.8 Hz, 1H, 5); ^15^N NMR (61 MHz, CD_2_Cl_2_) *δ* −110.1.

#### 3-Bromopyridine (4)

^1^H NMR (500 MHz, CD_2_Cl_2_) *δ* 8.67 (d, *J* = 2.4 Hz, 1H, 2), 8.51 (dd, *J* = 4.7, 1.5 Hz, 1H, 6), 7.84 (ddd, *J* = 8.2, 2.4, 1.5 Hz, 1H, 4), 7.21 (dd, *J* = 8.1, 4.7 Hz, 1H, 5); ^15^N NMR (51 MHz, CD_2_Cl_2_) *δ* −60.3.

#### [Bis(3-bromopyridine)silver(i)]^+^ ([4–Ag–4]^+^)

^1^H NMR (600 MHz, CD_2_Cl_2_) *δ* 8.76 (dd, *J* = 2.3, 0.7 Hz, 2H, 2), 8.63 (dd, *J* = 5.2, 1.4 Hz, 2H, 6), 8.11 (ddd, *J* = 8.3, 2.3, 1.4 Hz, 2H, 4), 7.49 (ddd, *J* = 8.3, 5.2, 0.7 Hz, 2H, 5); ^15^N NMR (61 MHz, CD_2_Cl_2_) *δ* −112.7.

#### [Bis(3-bromopyridine)iodine(i)]^+^ ([4–I–4]^+^)

^1^H NMR (600 MHz, CD_2_Cl_2_) *δ* 8.85 (d, *J* = 2.1 Hz, 2H, 2), 8.75 (dd, *J* = 5.5, 1.2 Hz, 2H, 6), 8.35 (ddd, *J* = 8.3, 2.1, 1.2 Hz, 2H, 4), 7.57 (dd, *J* = 8.3, 5.5 Hz, 2H, 5); ^15^N NMR (61 MHz, CD_2_Cl_2_) *δ* −172.0.

#### 3-Bromopyridin-1-ium ([4H]^+^)

^1^H NMR (600 MHz, CD_2_Cl_2_) *δ* 8.80 (d, *J* = 2.2 Hz, 1H, 2), 8.64 (dd, *J* = 5.2, 1.3 Hz, 1H, 6), 8.17 (ddd, *J* = 8.2, 2.1, 1.5 Hz, 1H, 4), 7.51 (dd, *J* = 8.2, 5.1 Hz, 1H, 5); ^15^N NMR (61 MHz, CD_2_Cl_2_) *δ* −104.7.

#### 3-Iodopyridine (5)

^1^H NMR (600 MHz, CD_2_Cl_2_) *δ* 8.82 (s, 1H, 2), 8.53 (d, *J* = 3.8 Hz, 1H, 6), 8.02 (d, *J* = 8.0 Hz, 1H, 4), 7.11 (dd, *J* = 8.2, 3.9 Hz, 1H, 5); ^15^N NMR (61 MHz, CD_2_Cl_2_) *δ* −59.5.

#### [Bis(3-iodopyridine)silver(i)]^+^ ([5–Ag–5]^+^)

^1^H NMR (600 MHz, CD_2_Cl_2_) *δ* 8.88 (d, *J* = 1.7 Hz, 2H, 2), 8.64 (dd, *J* = 5.3, 1.4 Hz, 2H, 6), 8.36 (d, *J* = 5.3 Hz, 2H, 4), 7.44 (t, *J* = 5.4 Hz, 2H, 5). No ^15^N NMR signals were observed at 298 K due to poor solubility.

#### [Bis(3-iodopyridine)iodine(i)]^+^ ([5–I–5]^+^)

^1^H NMR (600 MHz, CD_2_Cl_2_) *δ* 8.96 (d, *J* = 1.6 Hz, 2H, 2), 8.75 (d, *J* = 5.5 Hz, 2H, 6), 8.51 (dt, *J* = 8.3, 1.5 Hz, 2H, 4), 7.44 (dd, *J* = 8.2, 5.5 Hz, 2H, 5); ^15^N NMR (61 MHz, CD_2_Cl_2_) *δ* −171.1.

#### 3-Iodopyridin-1-ium ([5H]^+^)

^1^H NMR (600 MHz, CD_2_Cl_2_) *δ* 8.96 (d, *J* = 1.7 Hz, 1H, 2), 8.70 (dd, *J* = 5.2, 1.2 Hz, 1H, 6), 8.42 (dt, *J* = 8.1, 1.8 Hz, 1H, 4), 7.45 (dd, *J* = 8.1, 5.3 Hz, 1H, 5); ^15^N NMR (61 MHz, CD_2_Cl_2_) *δ* −115.4.

#### 2-Chloro-5-iodopyridine (6)

^1^H NMR (500 MHz, CD_2_Cl_2_) *δ* 8.59 (d, *J* = 2.4 Hz, 1H, 2), 7.95 (dd, *J* = 8.4, 2.4 Hz, 1H, 4), 7.15 (d, *J* = 8.4 Hz, 1H, 5); ^15^N NMR (51 MHz, CD_2_Cl_2_) *δ* −67.8.

#### [Bis(2-chloro-5-iodopyridine)silver(i)]^+^ ([6–Ag–6]^+^)

^1^H NMR (600 MHz, CD_2_Cl_2_) *δ* 8.86 (d, *J* = 2.2 Hz, 2H, 2), 8.26 (dd, *J* = 8.5, 2.3 Hz, 2H, 4), 7.44 (d, *J* = 8.4 Hz, 2H, 5); ^15^N NMR (61 MHz, CD_2_Cl_2_) *δ* −100.2.

#### [Bis(2-chloro-5-iodopyridine)iodine(i)]^+^ ([6–I–6]^+^)

^1^H NMR (600 MHz, CD_2_Cl_2_) *δ* 9.10 (s, 2H, 2), 8.44 (d, *J* = 7.6 Hz, 2H, 4), 7.61 (d, *J* = 7.6 Hz, 2H, 5). No ^15^N NMR signals were observed at 298 K.

#### 2-Chloro-5-iodopyridin-1-ium ([6H]^+^)

^1^H NMR (600 MHz, CD_2_Cl_2_) *δ* 8.71 (dd, *J* = 2.3, 0.7 Hz, 1H, 2), 8.14 (dd, *J* = 8.4, 2.3 Hz, 1H, 4), 7.29 (dd, *J* = 8.4, 0.7 Hz, 1H, 5); ^15^N NMR (61 MHz, CD_2_Cl_2_) *δ* −92.5.

## Crystallization

Crystals suitable for X-ray diffraction were obtained by vapor diffusion with either pentane or diethyl ether ([4–Ag–4]PF_6_). Despite repeated attempts to crystallize [5–Ag–5]PF_6_, only poor-quality crystals were obtained by vapor diffusion with diethyl ether from a dichloromethane solution into which acetonitrile was added dropwise to dissolve the complex, and only a preliminary crystal structure could be determined (Fig. S59). Attempts to crystallize [6–I–6]PF_6_ only yielded crystals of pyridine 6. The fact that crystals of [6–I–6]PF_6_ were not obtained was not unexpected given that the crystal structure of the iodine(i) complex of 2-chloropyridine could not previously be determined either.^31^

## Author contributions

O. K.: investigation, methodology, visualization, writing – original draft, writing – review and editing. A. L.: conceptualization, supervision, methodology, writing – review and editing. A. P.: conceptualization, supervision, formal analysis, investigation, methodology, writing – review and editing.

## Conflicts of interest

There are no conflicts to declare.

## Supplementary Material

RA-OLF-D6RA07510J-s001

## Data Availability

CCDC 2467903–2467912, 2481496, and 2593238 contain the supplementary crystallographic data for this paper.^72*a*–*l*^ The data supporting this article have been included as part of the supplementary information (SI) Supplementary information: NMR spectra, additional X-ray crystallographic data, Hirshfeld surface analysis, PXRD patterns and fitting data, and DFT computational data. See DOI: https://doi.org/10.1039/d6ra07510j.
