# Supplementary material for: Structural and conformational trends of silver(i) and iodine(i) complexes of 3-substituted pyridines
Source: RSC Adv. 2026 Oct 20;16(48):64782–93. doi: 10.1039/d6ra07510j (PMC13682528; doi:10.1039/d6ra07510j)
Supplement: Supplementary file 1 [file RA-016-D6RA07510J-s001.pdf]

Supplementary Information (SI) for RSC Advances.
This journal is © The Royal Society of Chemistry

Supplementary Information for

Structural and conformational trends of silver(I) and iodine(I) complexes of 3-substituted pyridines

Oskari Koivisto, Ari Lehtonen and Anssi Peuronen*

Department of Chemistry, University of Turku, Henrikinkatu 2, 20500 Turku, Finland.

*Corresponding author. Email: anssi.peuronen@utu.fi.

Table of contents

1. Syntheses and characterization 2
2. NMR spectra 4
   2.1 ^{1}H spectra 4
   2.2 ^{1}H–^{15}N HMBC spectra 28
   2.3 ^{19}F spectra 52
   2.4 ^{31}P spectra 55
3. X-ray crystallography 58
   3.1 Silver(I) complexes 58
   3.2 Iodine(I) complexes 65
4. Hirshfeld surface analysis 71
5. Powder diffraction and Pawley fitting 74
6. DFT studies 87
References 94

1. Syntheses and characterization

The silver(I) and iodine(I) complexes of ligands **1**–**6** were prepared using slightly modified literature methods according to Scheme S1.[1,2] The silver(I) complexes could be prepared in high yields (> 80 %) by adding 2 equivalents of ligand **L** (**1**–**6**; 0.2 mmol) to a solution of 1 equivalent of $AgPF_6$ (0.1 mmol) in CH_2Cl_2 (5 ml). The iodine(I) complexes could be prepared in somewhat lower yields than corresponding silver(I) complexes (> 60 %) by adding 1 equivalent of elemental iodine (0.1 mmol) to the solution of a silver complex [**L**–Ag–**L**]$^+$ prepared as described above. The pale yellow AgI precipitate was separated by centrifugation (4400 rpm, 5 min) to give a purple solution containing the iodine(I) complex. Spectroscopic samples were prepared by this general method in CD_2Cl_2 (700 μl).

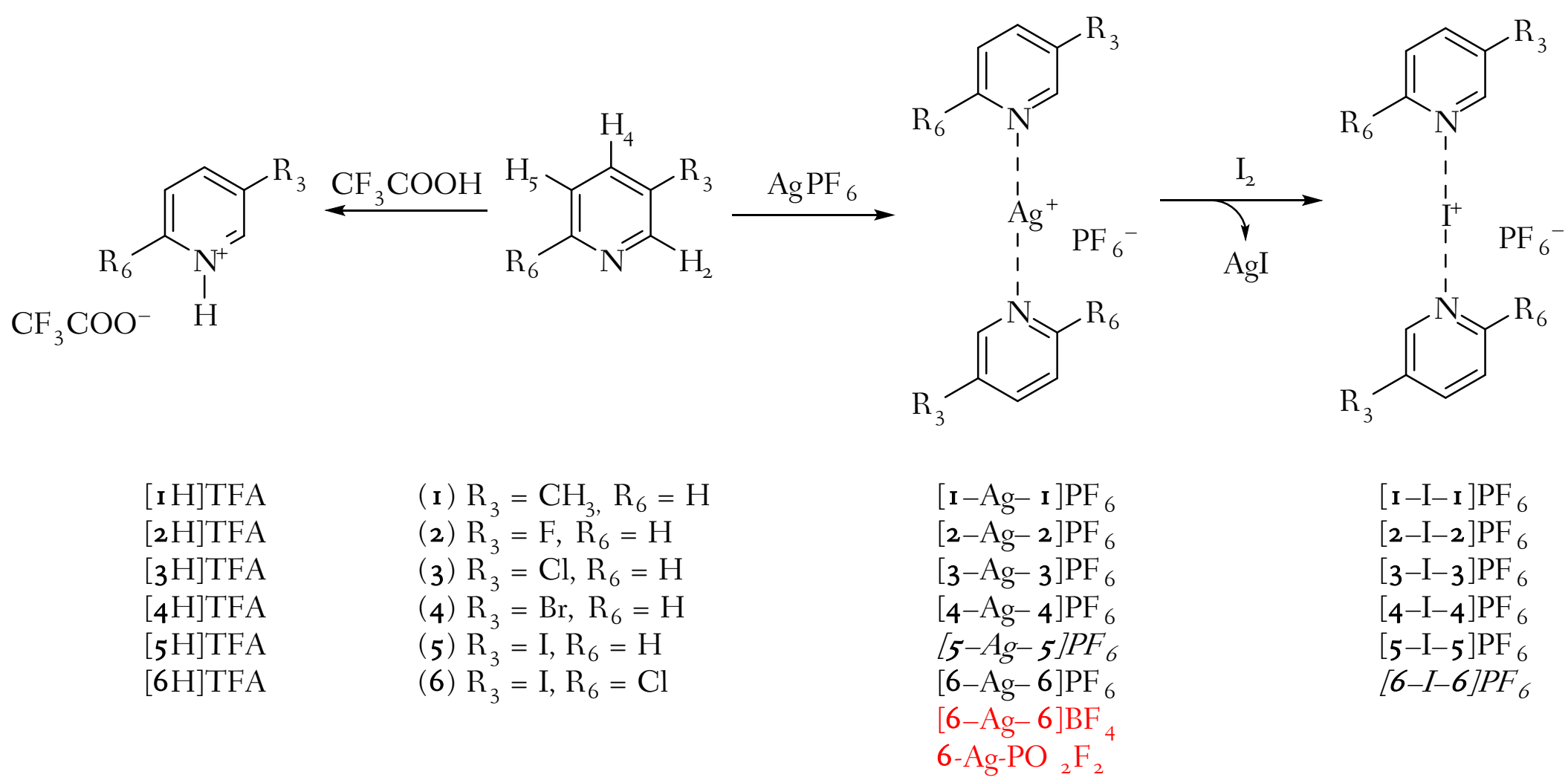

Scheme S1. Structural formulas and the synthetic routes to [**L**H]TFA, [**L**–Ag–**L**]PF_6 ([**6**–Ag–**6**]BF_4 and **6**-Ag-PO_2F_2) and [**L**–I–**L**]PF_6.

Chemical shifts are reported in δ (ppm) using residual solvent signals as the internal standard for the ^{1}H spectra. For the ^{1}H–^{15}N HMBC spectra, an internal reference in the form of a sealed capillary filled with a mixture of CD_2Cl_2 and nitromethane was used. Chemical shifts in ^{15}N were corrected to correspond to a pure nitromethane standard (δ$_N$ 0.0 ppm) by a ^{1}H–^{15}N HMBC measurement of nitromethane with the internal standard (δ$_N$ –4.2 ppm). The spectral widths were 10.8 ppm (^{1}H) and 399 ppm (^{15}N) with 128 increments for ^{15}N. For the ^{19}F and ^{31}P spectra, absolute referencing via the Ξratio[3] was used, with spectral widths of 241.5 ppm (^{19}F) and 402.8 (^{31}P). All spectra were processed with MestReNova 14.2.2.[4] In the ^{1}H NMR spectra, each resonance was assigned according to the following conventions: chemical shift (δ) in ppm, observed multiplicity, observed coupling constant (J in Hz), and number of hydrogens. The changes in ^{15}N chemical shifts are expressed as coordination shifts Δδ(^{15}N) obtained by subtracting the shift of the pure pyridine from that of the compound in question. Trifluoroacetate (TFA) salts of the protonated pyridines were prepared to confirm whether [**L**H]$^+$ are present in the solutions of silver(I) or iodine(I) complexes. The TFA salts of protonated ligands were prepared by adding 1 equivalent of trifluoroacetic acid (0.2 mmol) to the solution of 1 equivalent of ligand **L** (**1**–**6**; 0.2 mmol) in CD_2Cl_2 (700 μl). The ^{15}N

NMR chemical shifts of the pyridine nitrogen atoms in ligands **L**, complexes [**L**–Ag–**L**]$^+$ and [**L**–I–**L**]$^+$, and in [**L**H]$^+$ are presented in Table S1.

Table S1. The ^{15}N NMR chemical shifts δ$_N$ (in ppm) of the pyridine nitrogen atoms in pure ligands **L**, in complexes [**L**–Ag–**L**]$^+$ and [**L**–I–**L**]$^+$, and in protonated ligands [**L**H]$^+$.

**L**	Pure **L**	[**L**–Ag–**L**]$^+$	[**L**–I–**L**]$^+$	[**L**H]$^+$
**1**	–67.2	–122.4	–174.3	–151.3
**2**	–58.3	–120.3	–173.5	–108.3
**3**	–61.1	–121.2	–172.9	–110.1
**4**	–60.3	–112.7	–172.0	–104.7
**5**	–59.5	–	–171.1	–115.4
**6**	–67.8	–100.2	–	–92.5

2. NMR spectra

2.1 ^{1}H spectra

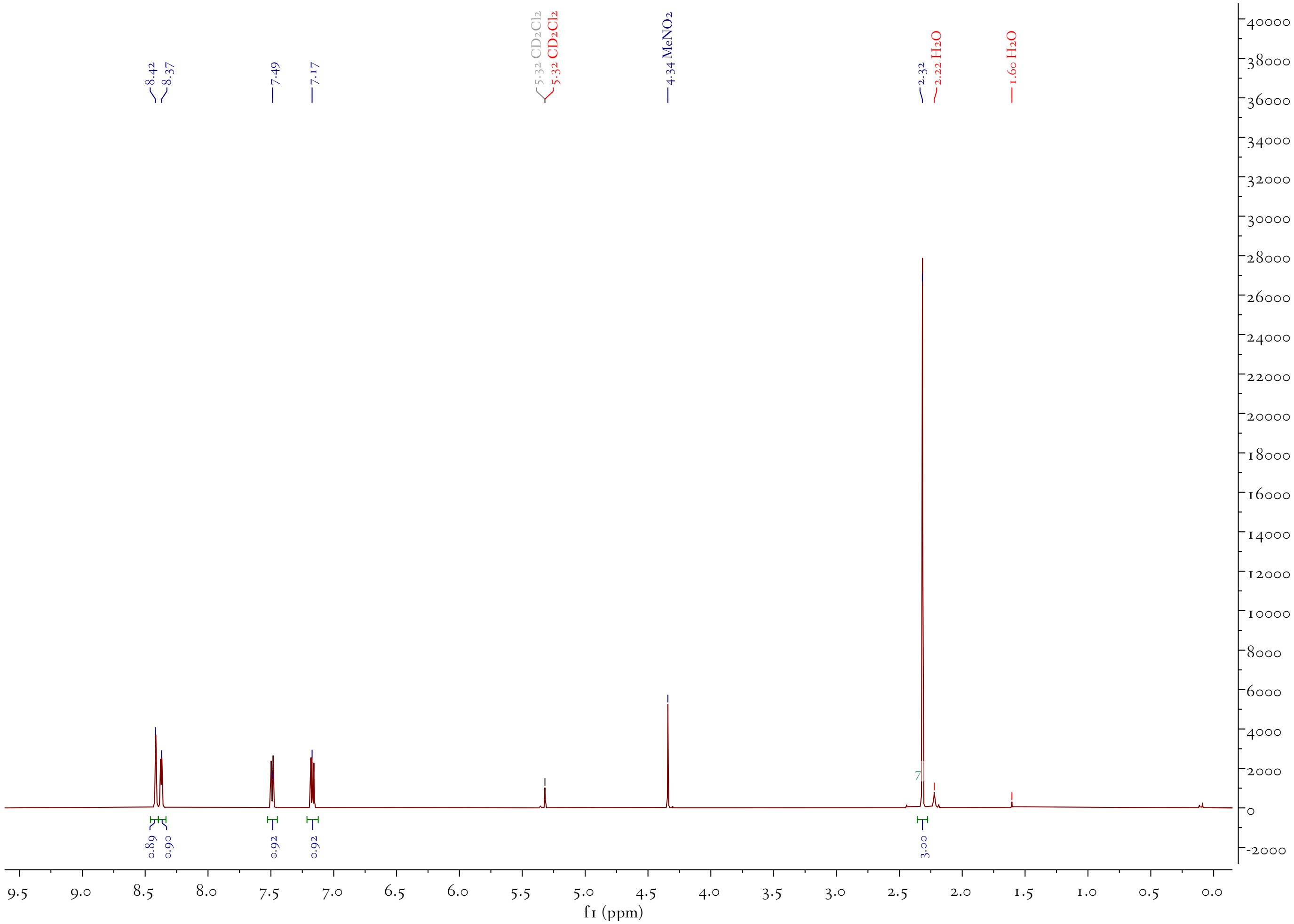

Figure S1. The ^{1}H spectrum of **1** in CD_2Cl_2.

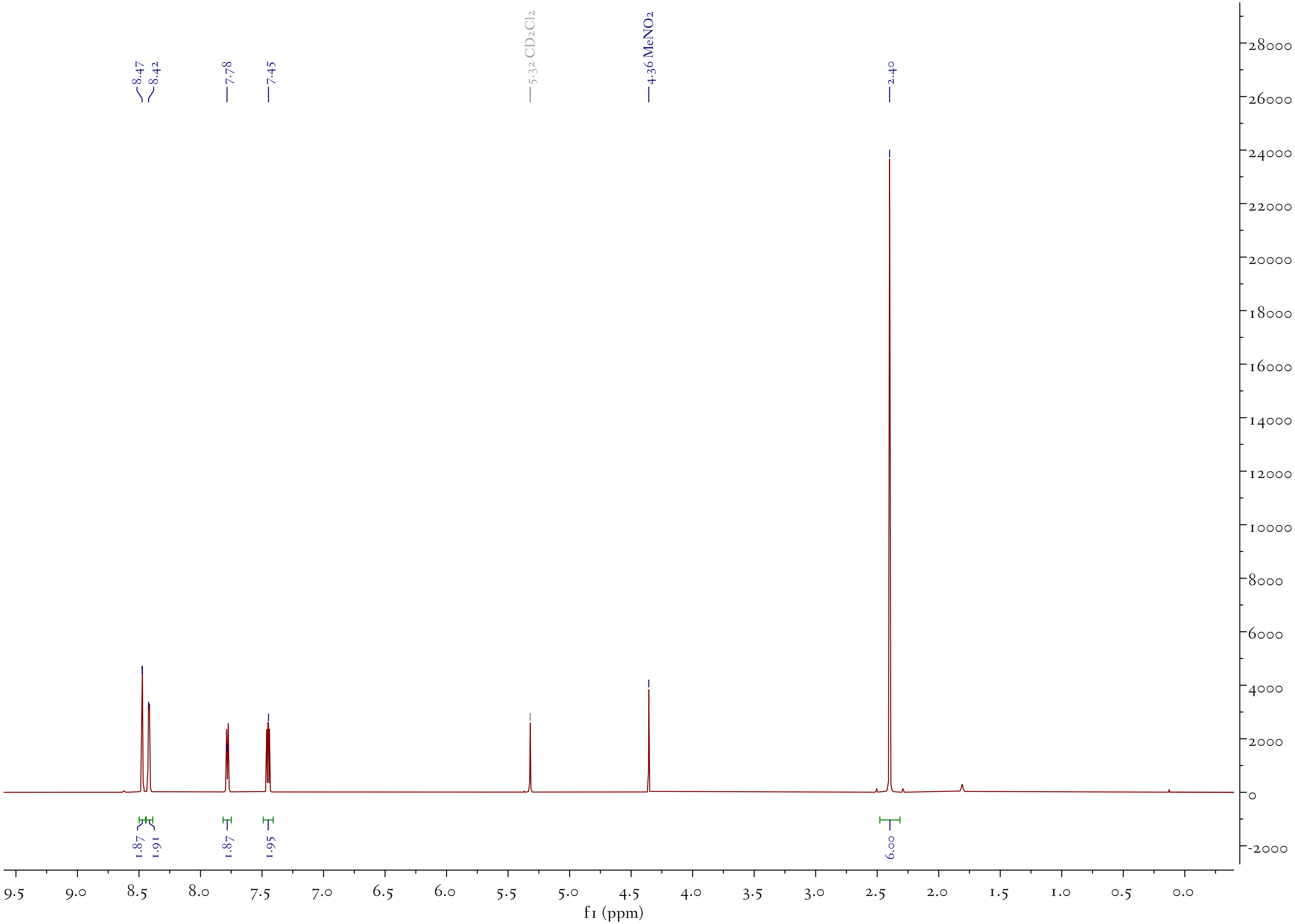

8.47
8.42
7.78
7.45
5.32 CD2Cl2
4.36 MeNO2
2.40
1.87
1.91
1.87
1.95
6.00
f1 (ppm)

Figure S2. The ^{1}H spectrum of [**1**–Ag–**1**]$^{+}$ in CD_2Cl_2.

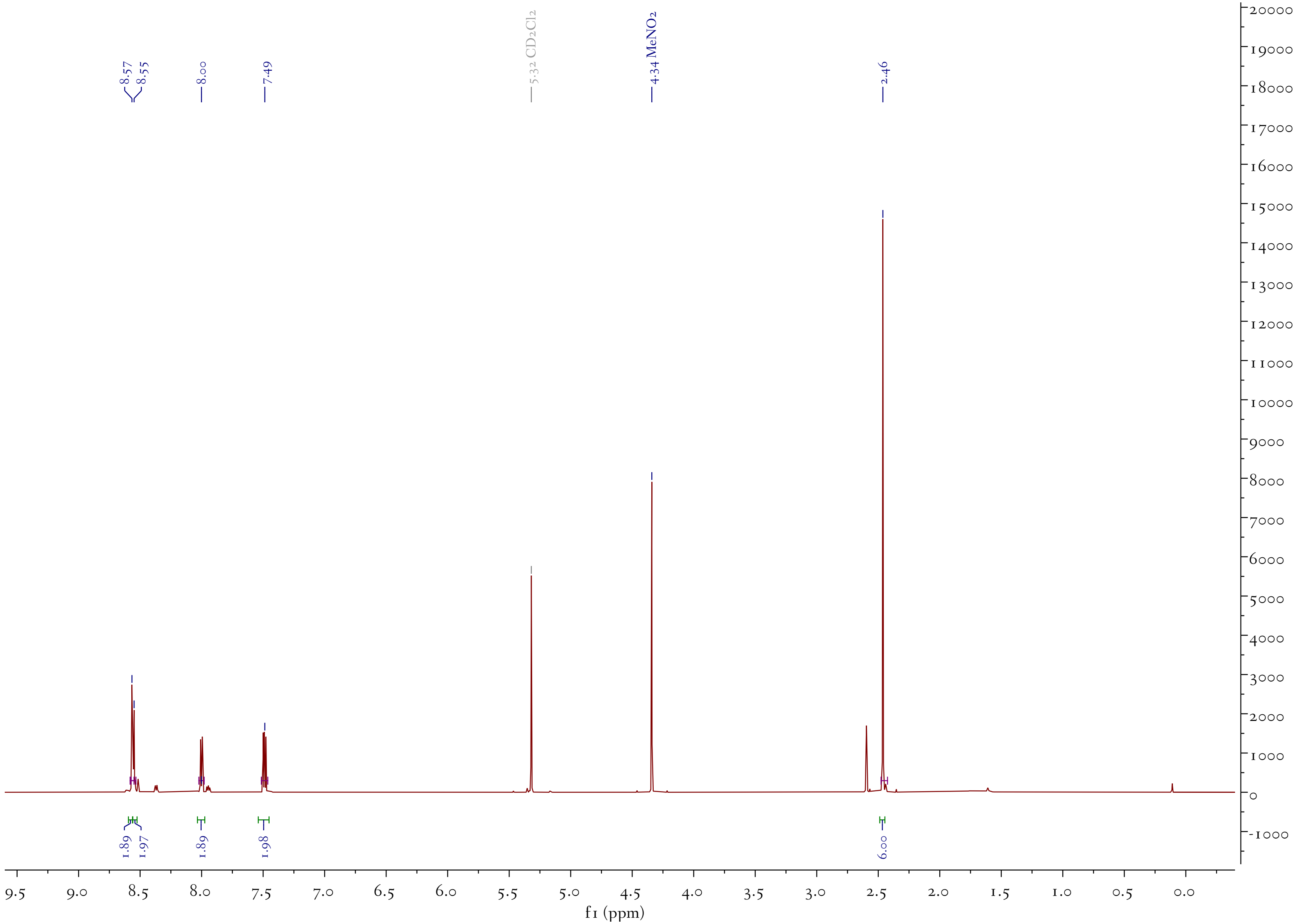

8.57
8.55
8.00
7.49
5.32 CD2Cl2
4.34 MeNO2
2.46
1.89
1.97
1.89
1.98
6.00
f1 (ppm)

Figure S3. The ^{1}H spectrum of [**1**–I–**1**]$^{+}$ in CD_2Cl_2.

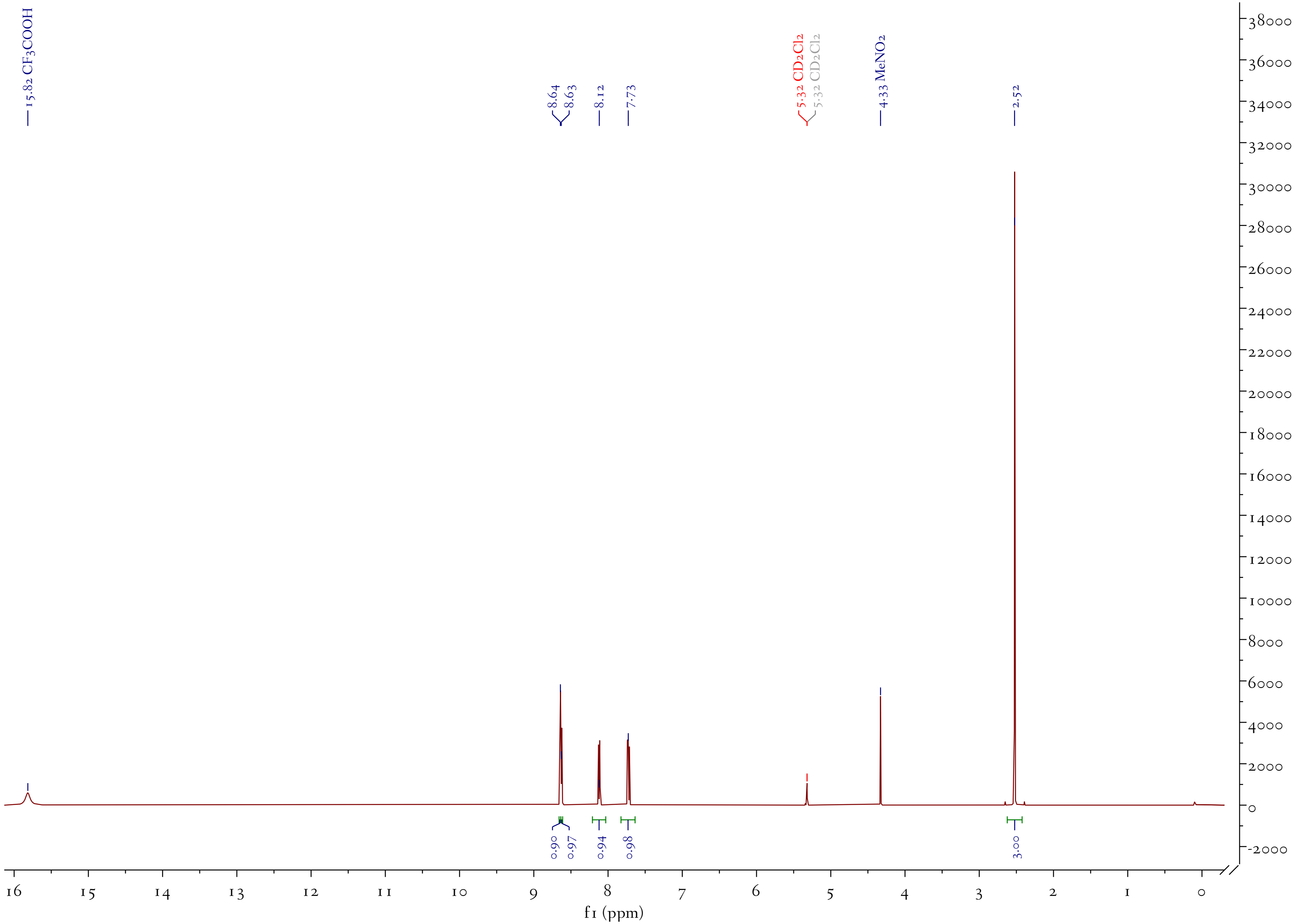
15.82 CF3COOH
8.64
8.63
8.12
7.73
5.32 CD2Cl2
5.32 CD2Cl2
4.33 MeNO2
2.52
0.90
0.97
0.94
0.98
3.00
f1 (ppm)

Figure S4. The ^{1}H spectrum of $[\mathbf{1}H]^+$ in CD_2Cl_2.

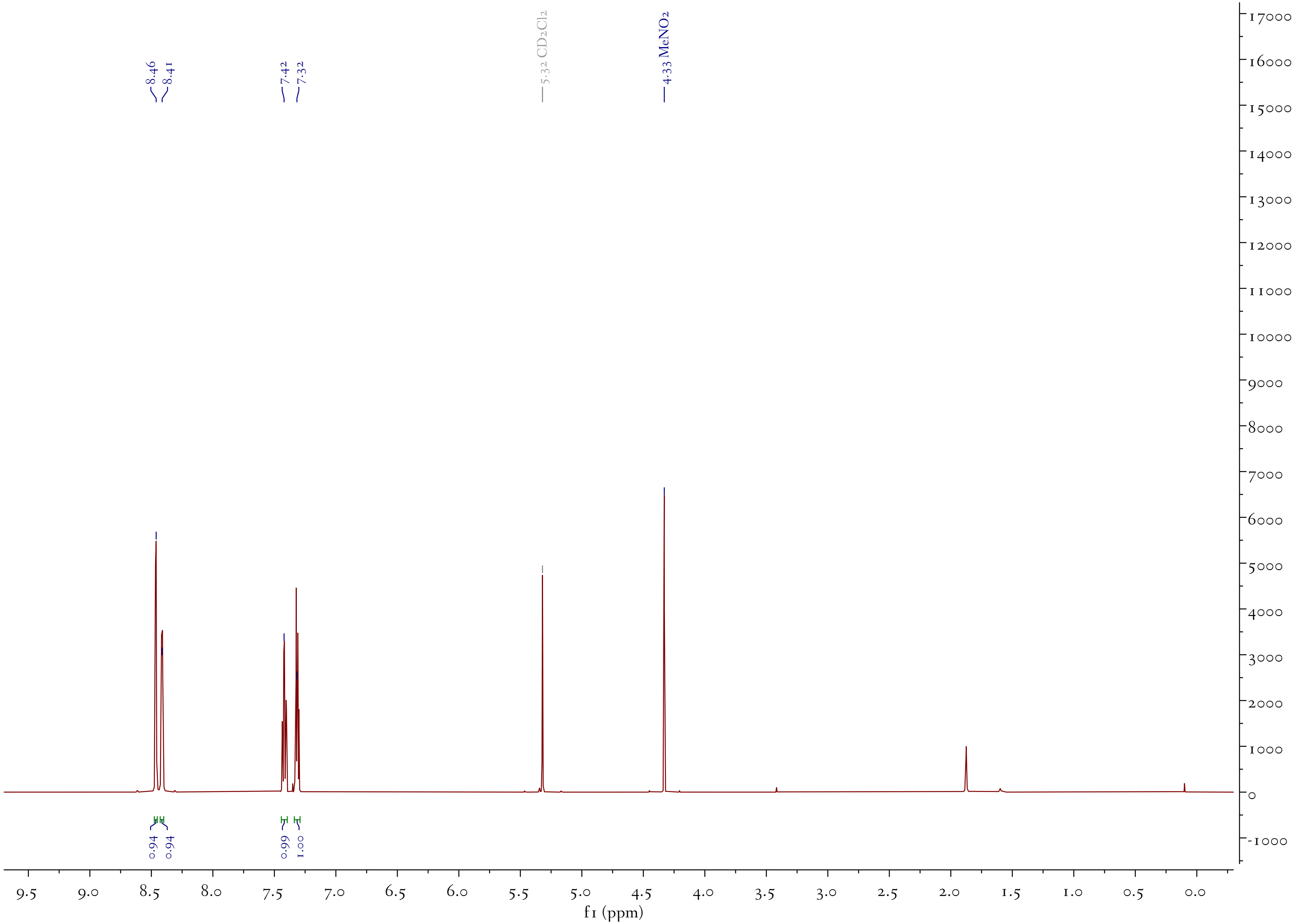

8.46
8.41
7.42
7.32
5.32 CD2Cl2
4.33 MeNO2
0.94
0.94
0.99
1.00
f1 (ppm)

Figure S5. The ^{1}H spectrum of **2** in CD_2Cl_2.

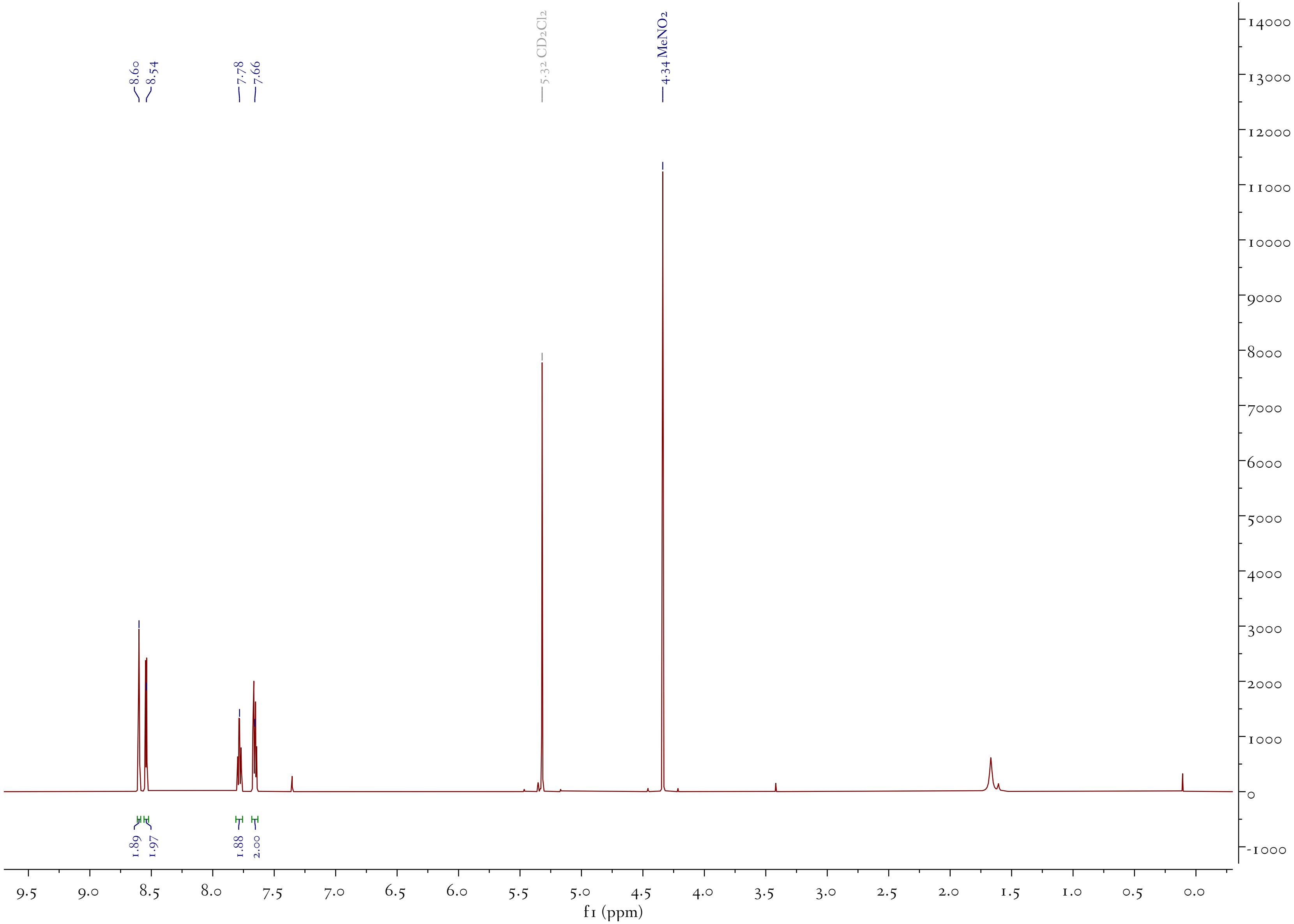
8.60
8.54
7.78
7.66
5.32 CD2Cl2
4.34 MeNO2
1.89
1.97
1.88
2.00
f1 (ppm)

Figure S6. The ^{1}H spectrum of [**2**–Ag–**2**]$^{+}$ in CD_2Cl_2.

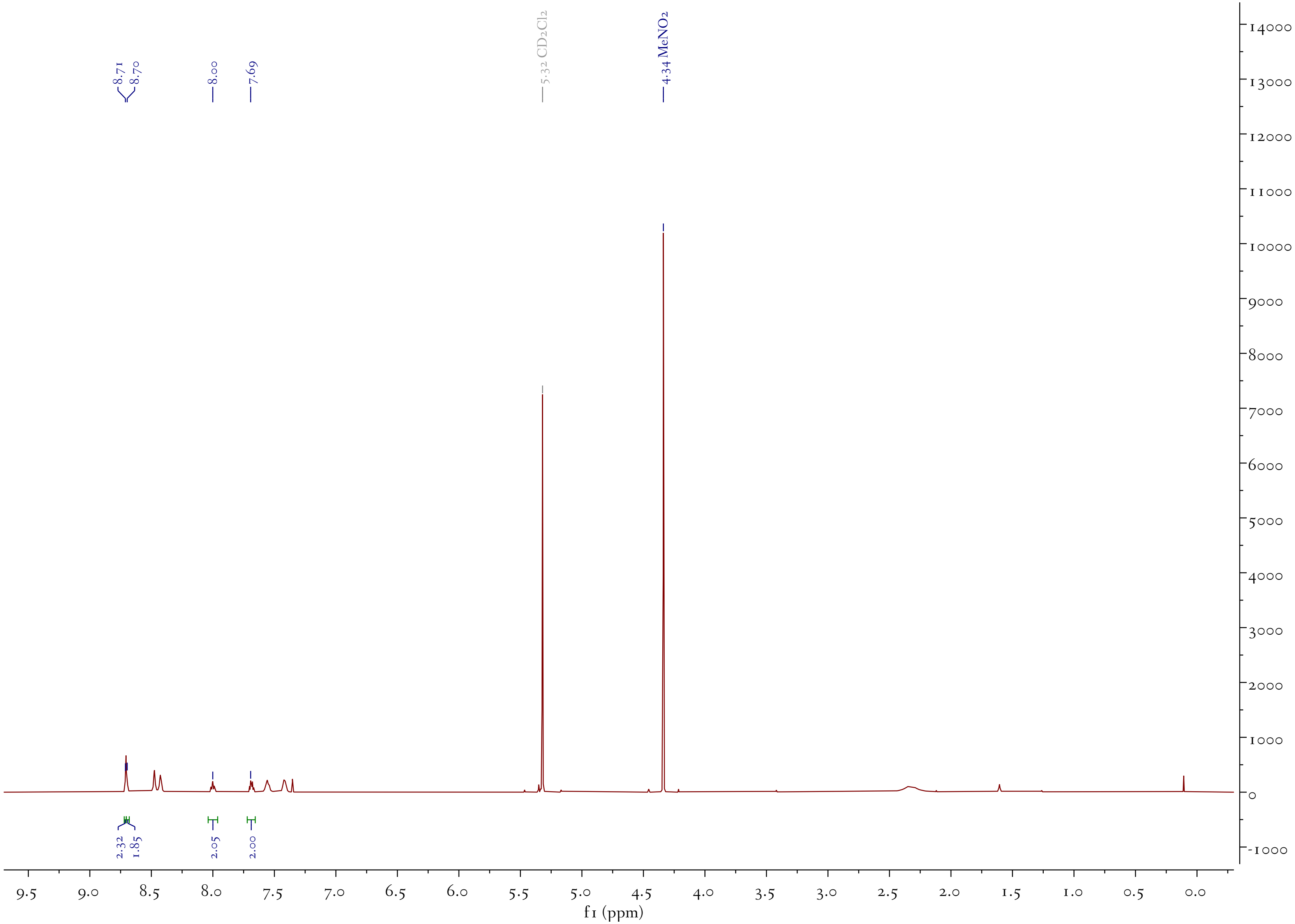

8.71
8.70
8.00
7.69
5.32 CD2Cl2
4.34 MeNO2
2.32
1.85
2.05
2.00
f1 (ppm)

Figure S7. The ^{1}H spectrum of [**2**–I–**2**]$^{+}$ in CD_2Cl_2.

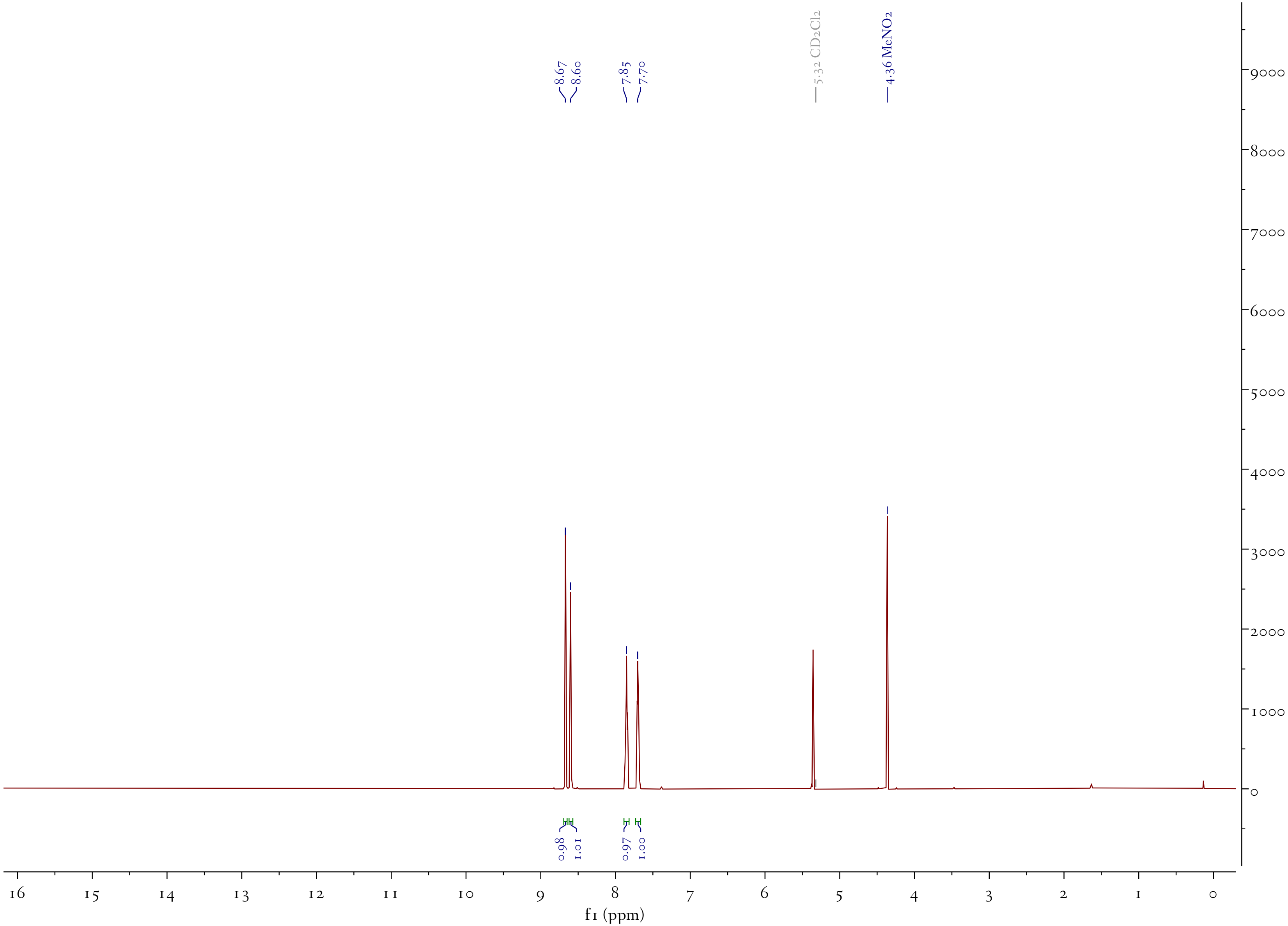
8.67
8.60
7.85
7.70
5.32 CD2Cl2
4.36 MeNO2
0.98
1.01
0.97
1.00
9000
8000
7000
6000
5000
4000
3000
2000
1000
0
16 15 14 13 12 11 10 9 8 7 6 5 4 3 2 1 0
f1 (ppm)

Figure S8. The ^{1}H spectrum of [**2**H]$^+$ in CD_2Cl_2.

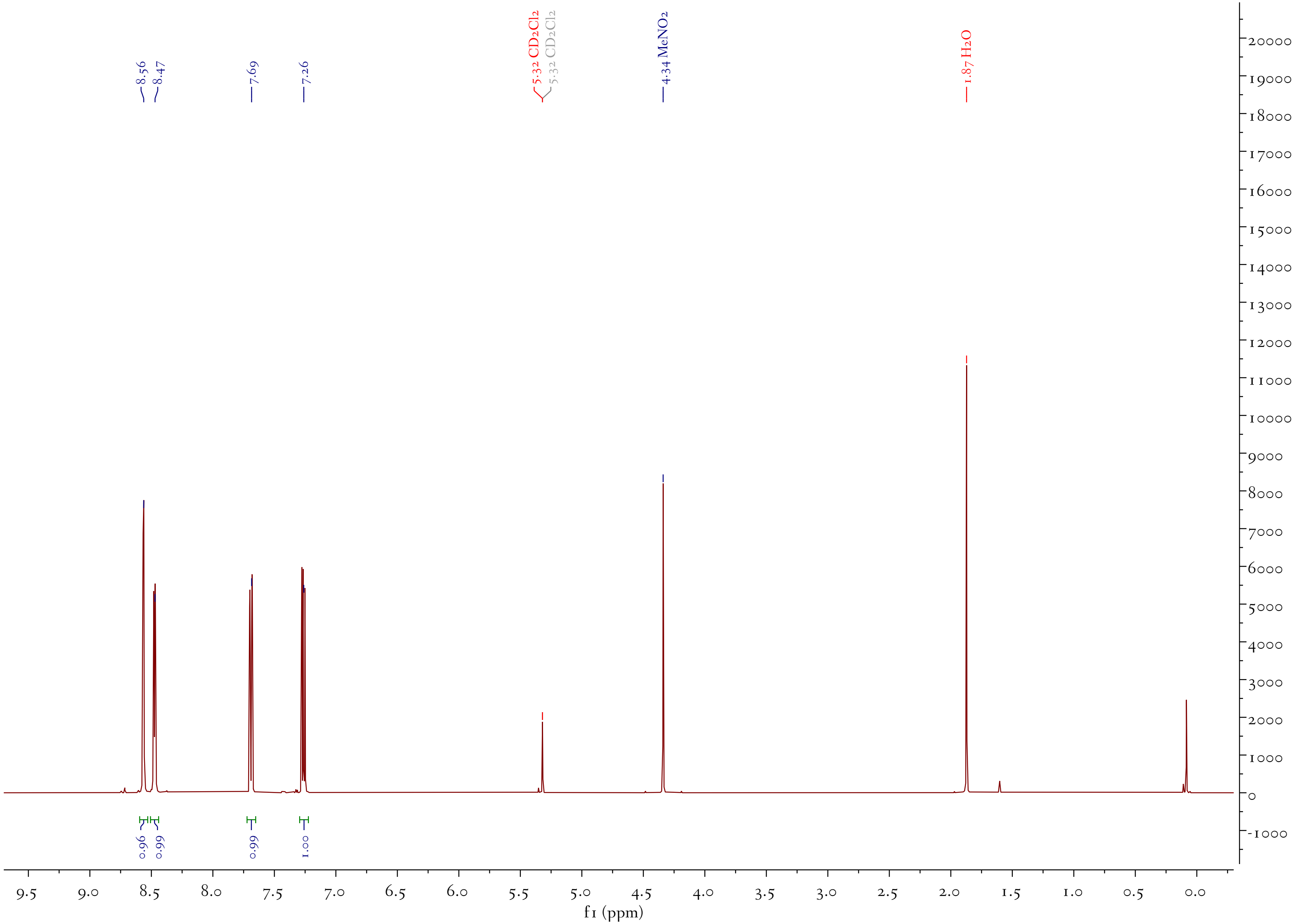
8.56
8.47
7.69
7.26
5.32 CD2Cl2
5.32 CD2Cl2
4.34 MeNO2
1.87 H2O
0.96
0.99
0.99
1.00
f1 (ppm)

Figure S9. The ^{1}H spectrum of **3** in CD_2Cl_2.

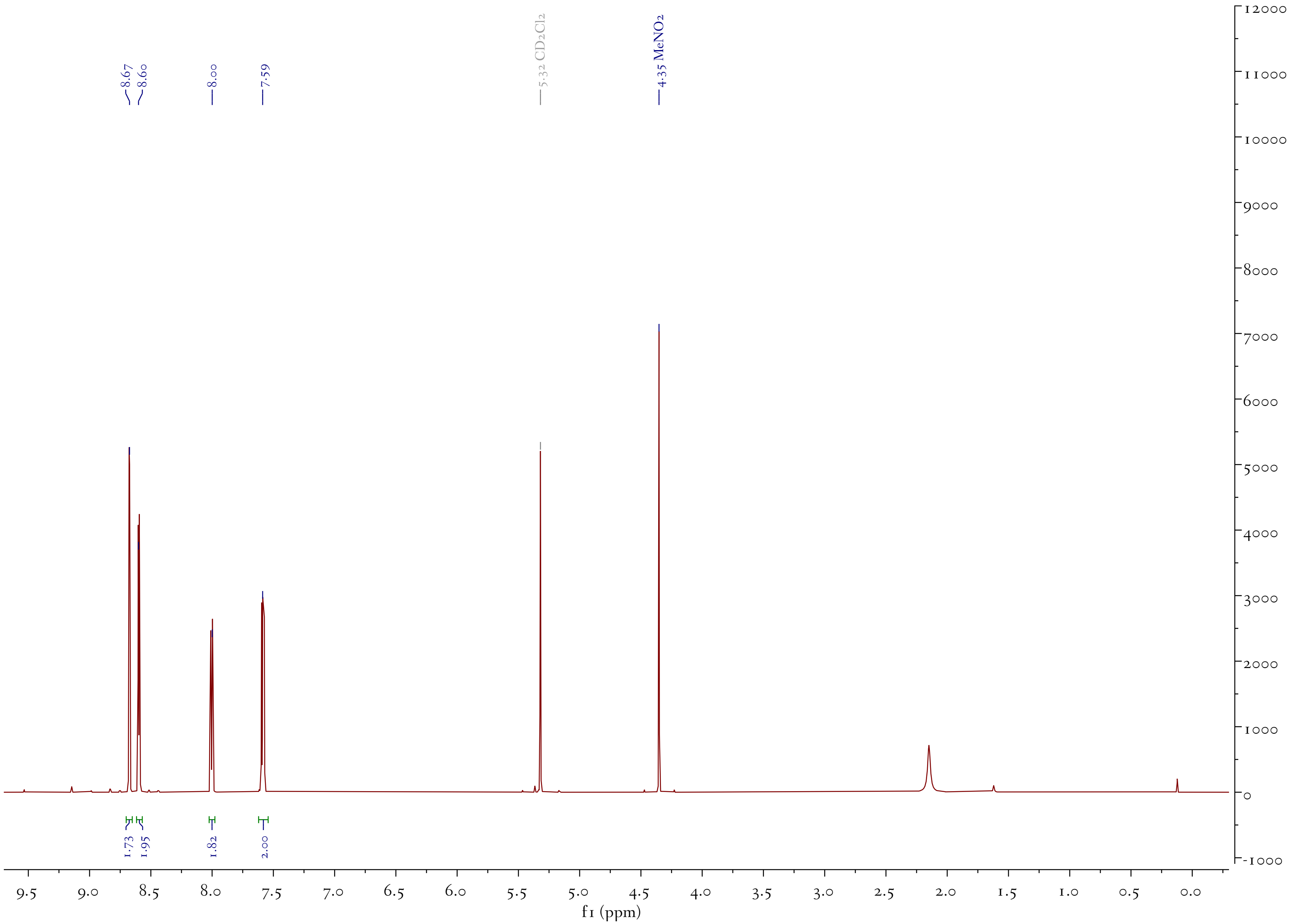

8.67
8.60
8.00
7.59
5.32 CD2Cl2
4.35 MeNO2
1.73
1.95
1.82
2.00
f1 (ppm)

Figure S10. The ^{1}H spectrum of [**3**–Ag–**3**]$^{+}$ in CD_2Cl_2.

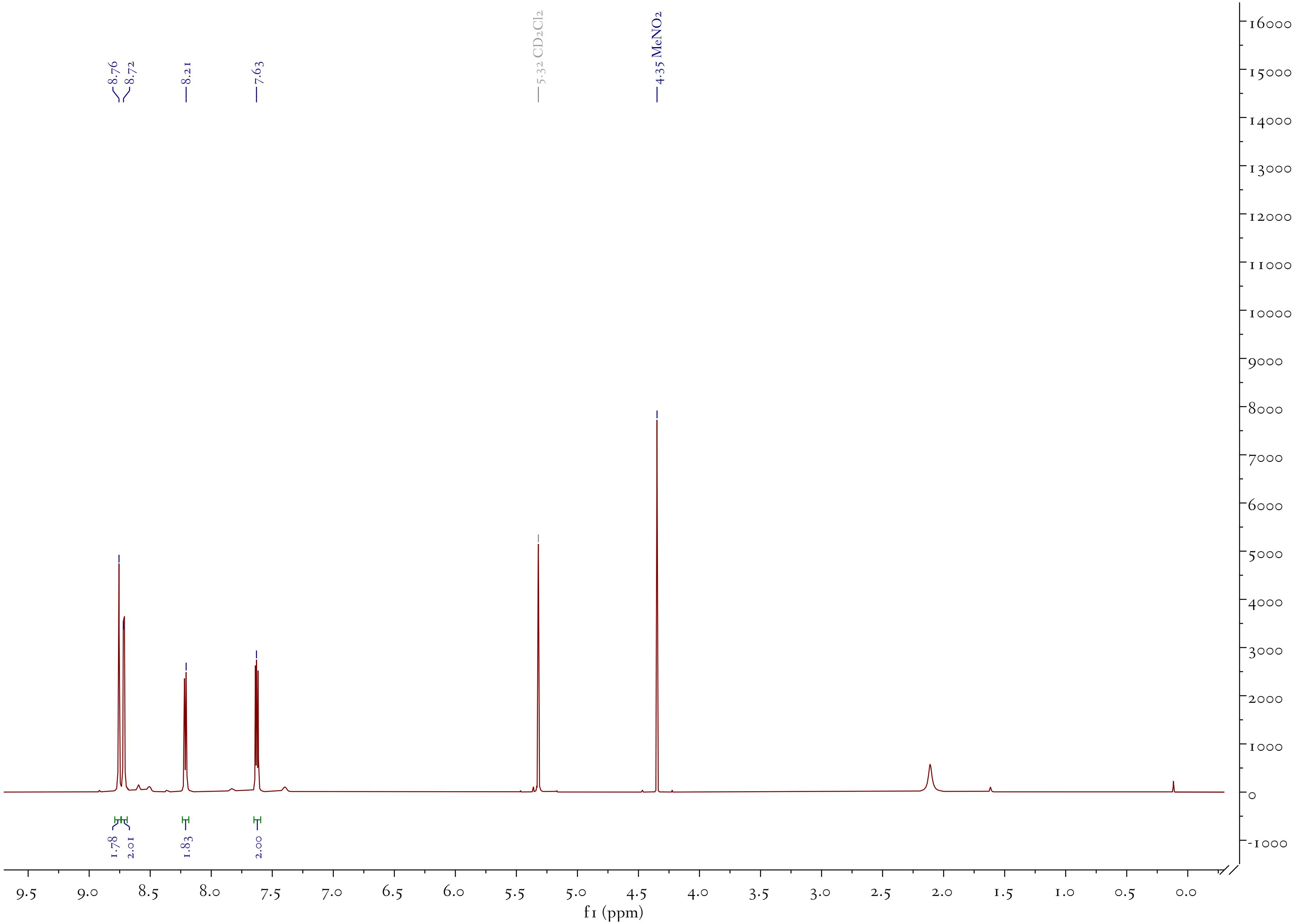
8.76
8.72
8.21
7.63
5.32 CD2Cl2
4.35 MeNO2
1.78
2.01
1.83
2.00
16000
15000
14000
13000
12000
11000
10000
9000
8000
7000
6000
5000
4000
3000
2000
1000
0
-1000
9.5
9.0
8.5
8.0
7.5
7.0
6.5
6.0
5.5
5.0
4.5
4.0
3.5
3.0
2.5
2.0
1.5
1.0
0.5
0.0
f1 (ppm)

Figure S11. The ^{1}H spectrum of [**3**–I–**3**]$^+$ in CD_2Cl_2.

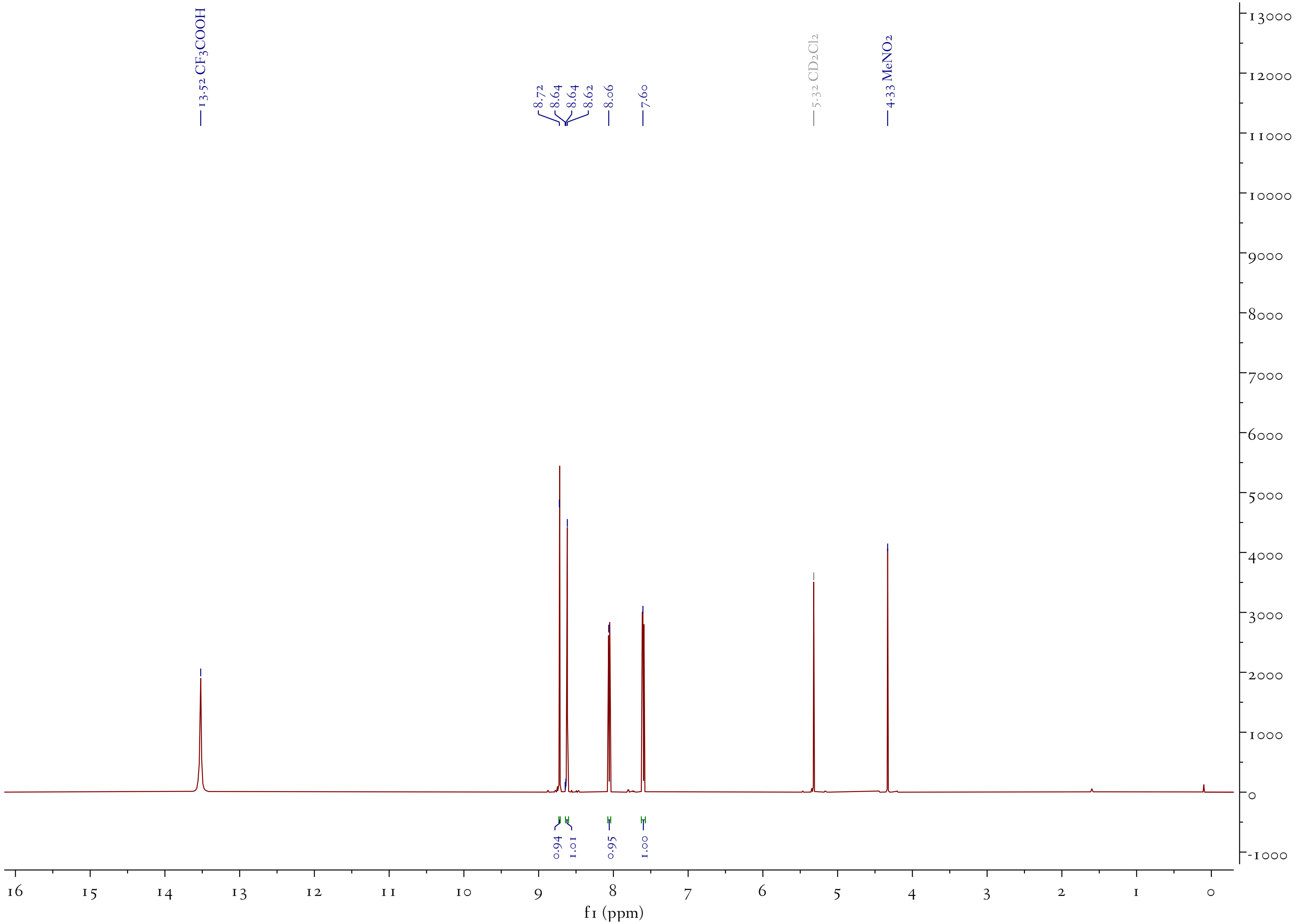

13.52 CF3COOH
8.72
8.64
8.64
8.62
8.06
7.60
5.32 CD2Cl2
4.33 MeNO2
0.94
1.01
0.95
1.00
13000
12000
11000
10000
9000
8000
7000
6000
5000
4000
3000
2000
1000
0
-1000
16
15
14
13
12
11
10
9
8
7
6
5
4
3
2
1
0
f1 (ppm)

Figure S12. The ^{1}H spectrum of [**3**H]$^{+}$ in CD_2Cl_2.

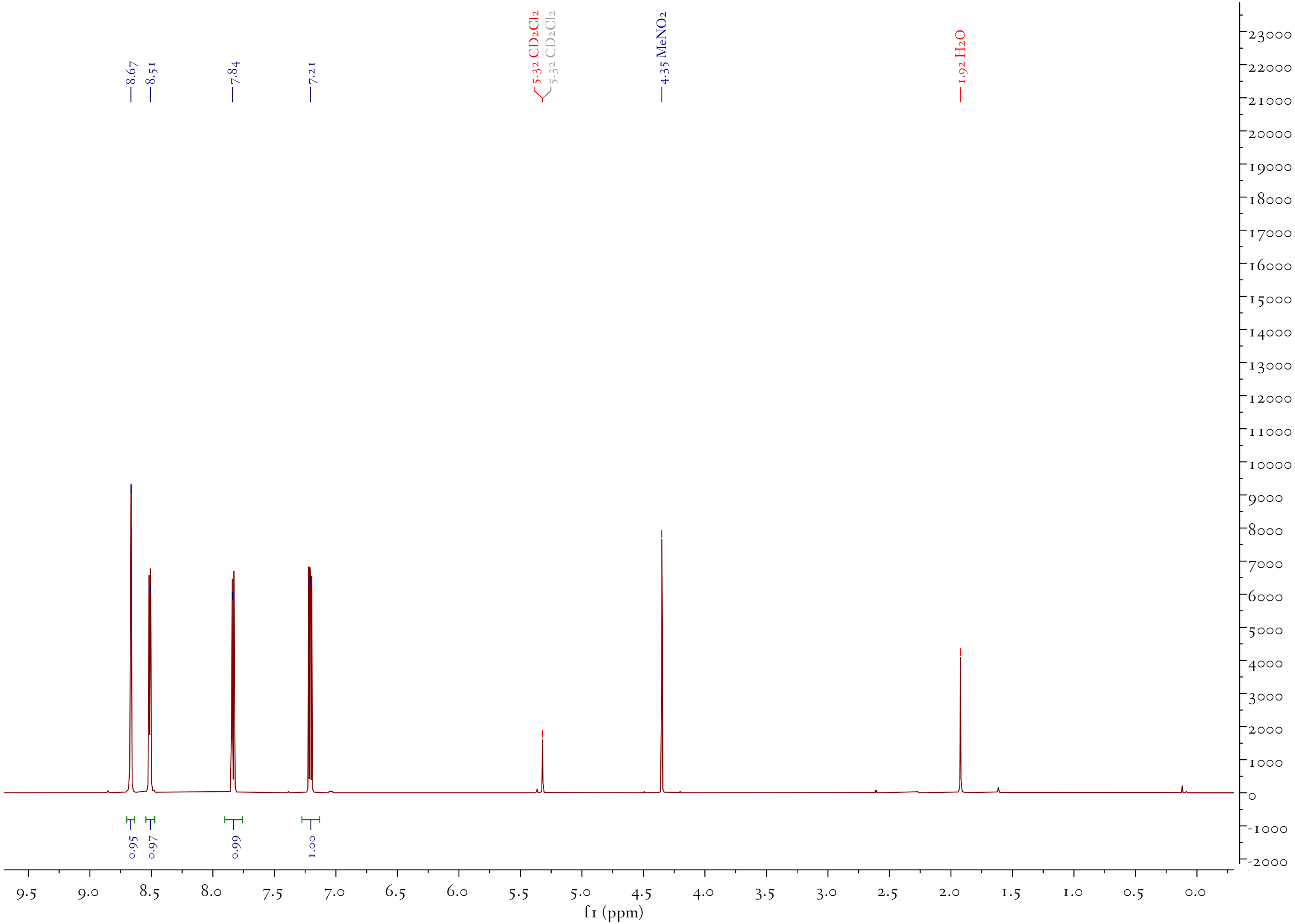

8.67
8.51
7.84
7.21
5.32 CD2Cl2
5.32 CD2Cl2
4.35 MeNO2
1.92 H2O
0.95
0.97
0.99
1.00
f1 (ppm)

Figure S13. The ^1H spectrum of **4** in CD_2Cl_2.

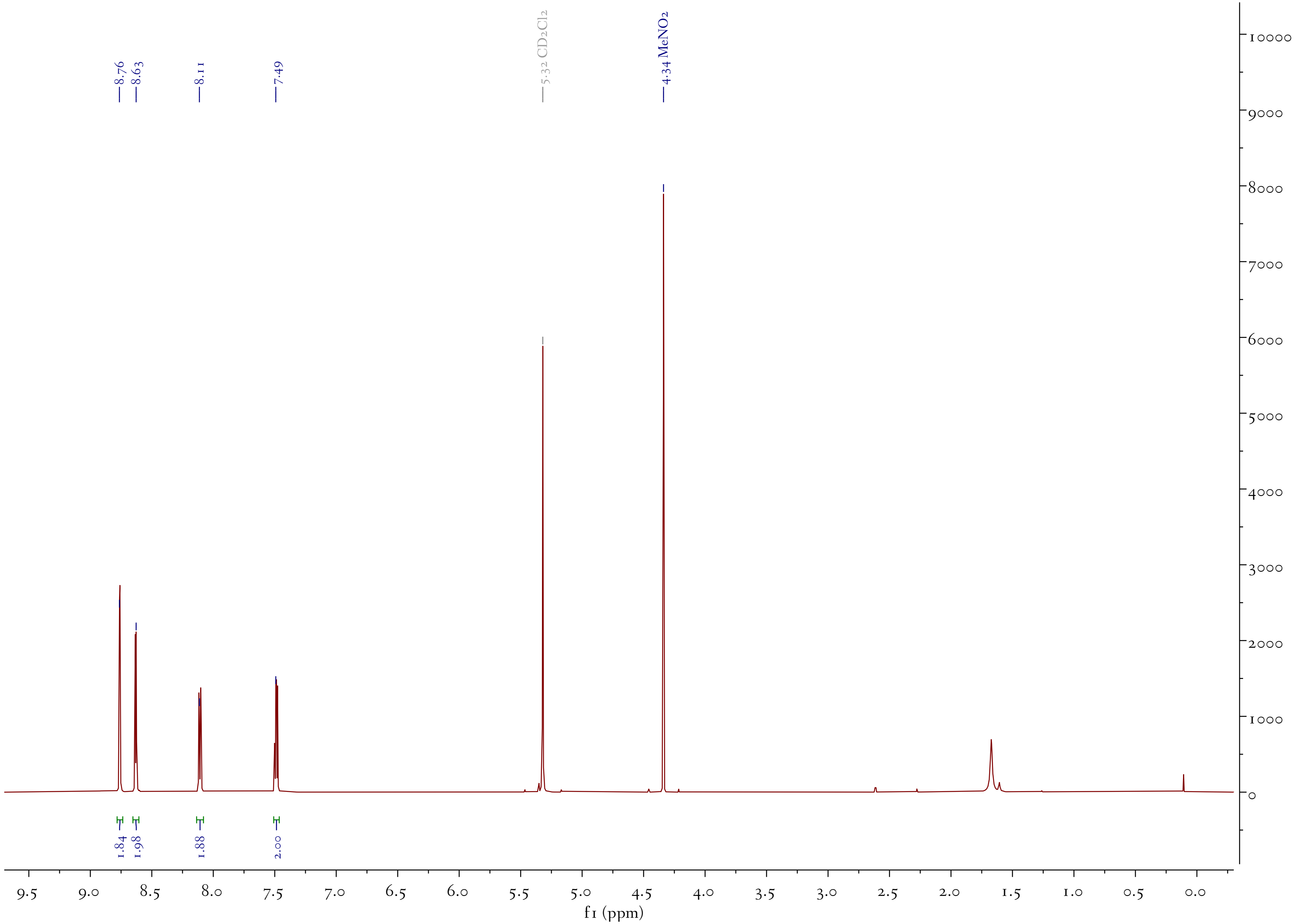
8.76
8.63
8.11
7.49
5.32 CD2Cl2
4.34 MeNO2
1.84
1.98
1.88
2.00
10000
9000
8000
7000
6000
5000
4000
3000
2000
1000
0
9.5
9.0
8.5
8.0
7.5
7.0
6.5
6.0
5.5
5.0
4.5
4.0
3.5
3.0
2.5
2.0
1.5
1.0
0.5
0.0
f1 (ppm)

Figure S14. The ^{1}H spectrum of [**4**–Ag–**4**]$^{+}$ in CD_2Cl_2.

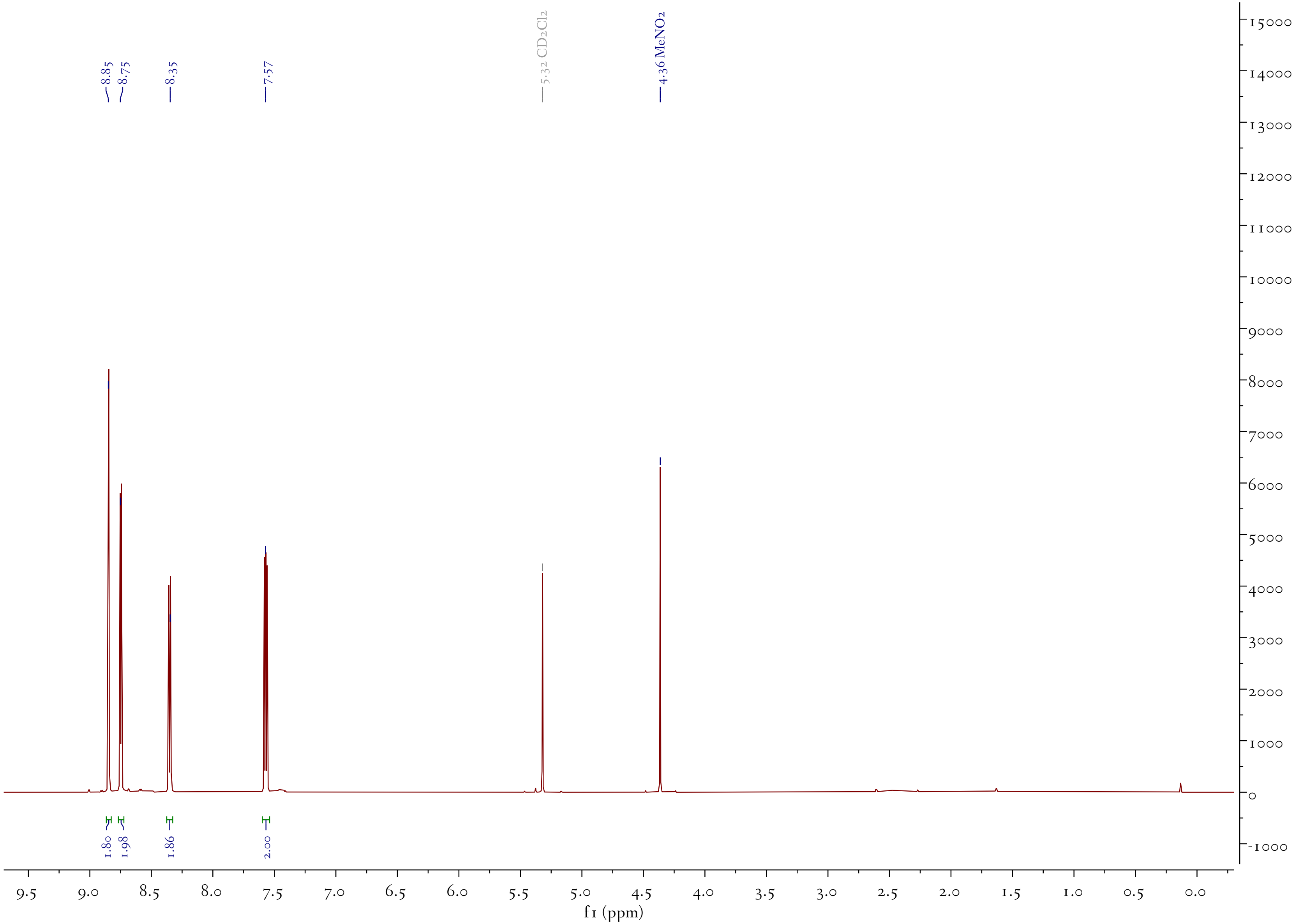

8.85
8.75
8.35
7.57
5.32 CD2Cl2
4.36 MeNO2
1.80
1.98
1.86
2.00
f1 (ppm)

Figure S15. The ^{1}H spectrum of [**4**–I–**4**]$^{+}$ in CD_2Cl_2.

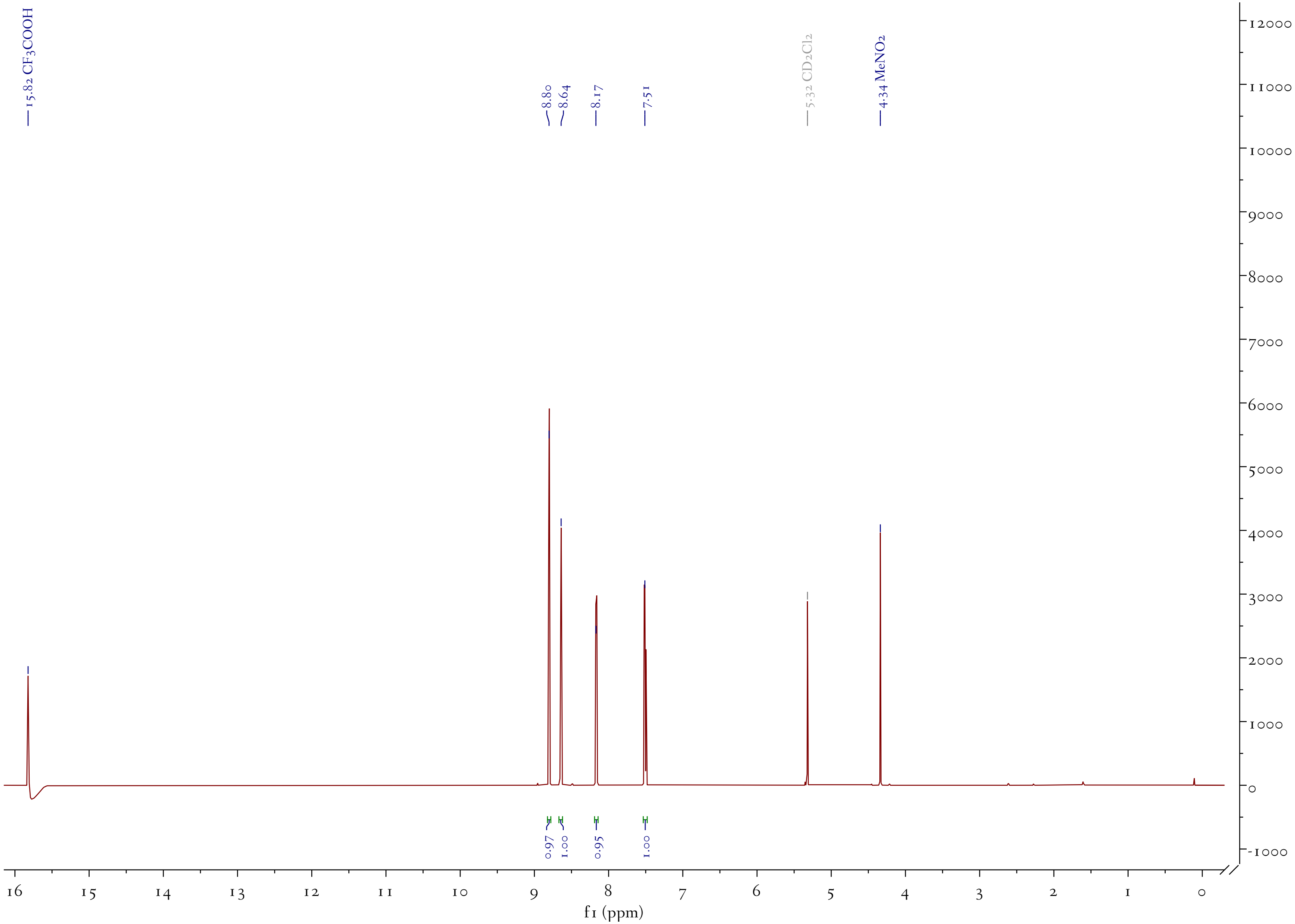
15.82 CF3COOH
8.80
8.64
8.17
7.51
5.32 CD2Cl2
4.34 MeNO2
0.97
1.00
0.95
1.00
12000
11000
10000
9000
8000
7000
6000
5000
4000
3000
2000
1000
0
-1000
16
15
14
13
12
11
10
9
8
7
6
5
4
3
2
1
0
f1 (ppm)

Figure S16. The ^{1}H spectrum of [**4**H]$^+$ in CD_2Cl_2.

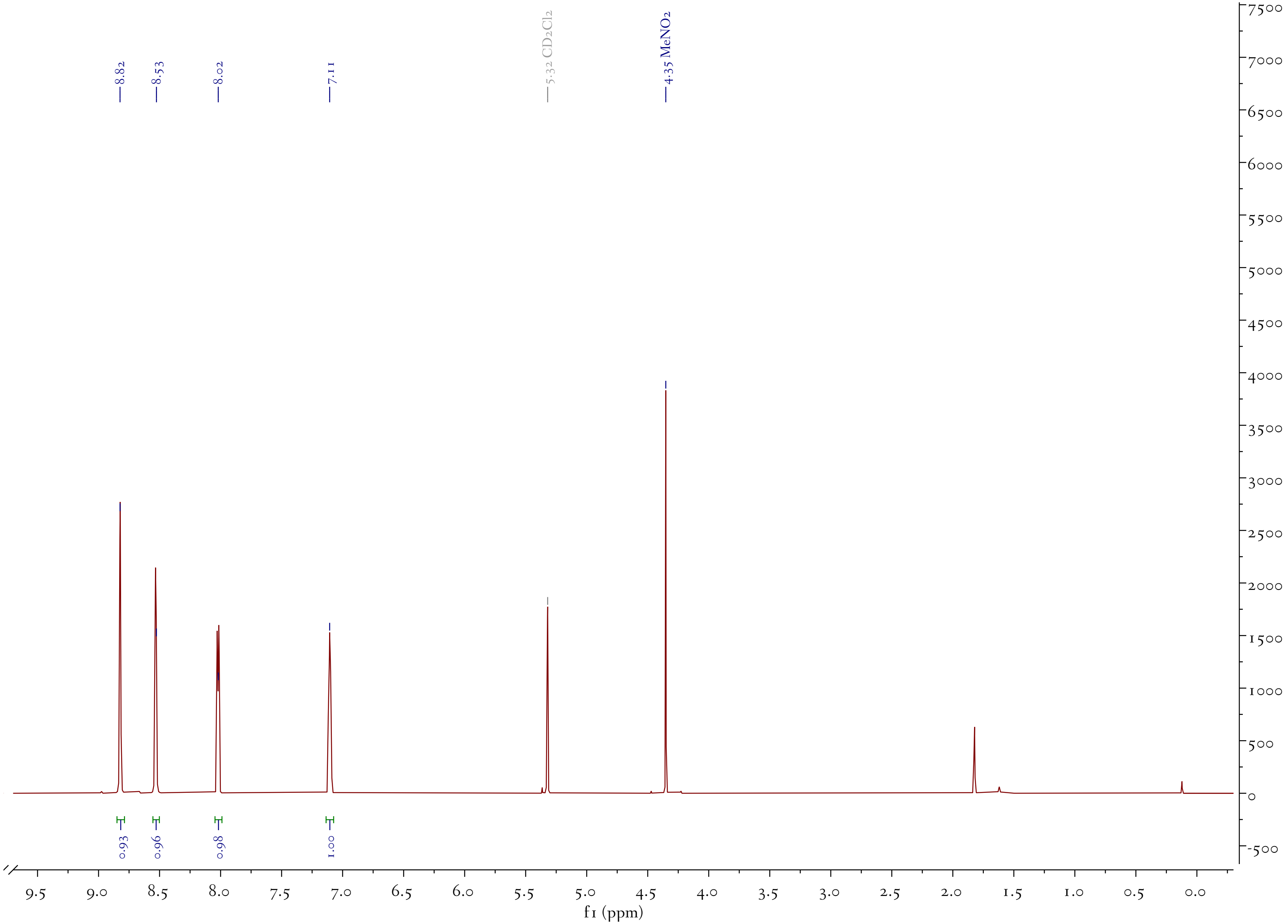

8.82
8.53
8.02
7.11
5.32 CD2Cl2
4.35 MeNO2
0.93
0.96
0.98
1.00
f1 (ppm)

Figure S17. The ^{1}H spectrum of **5** in CD_2Cl_2.

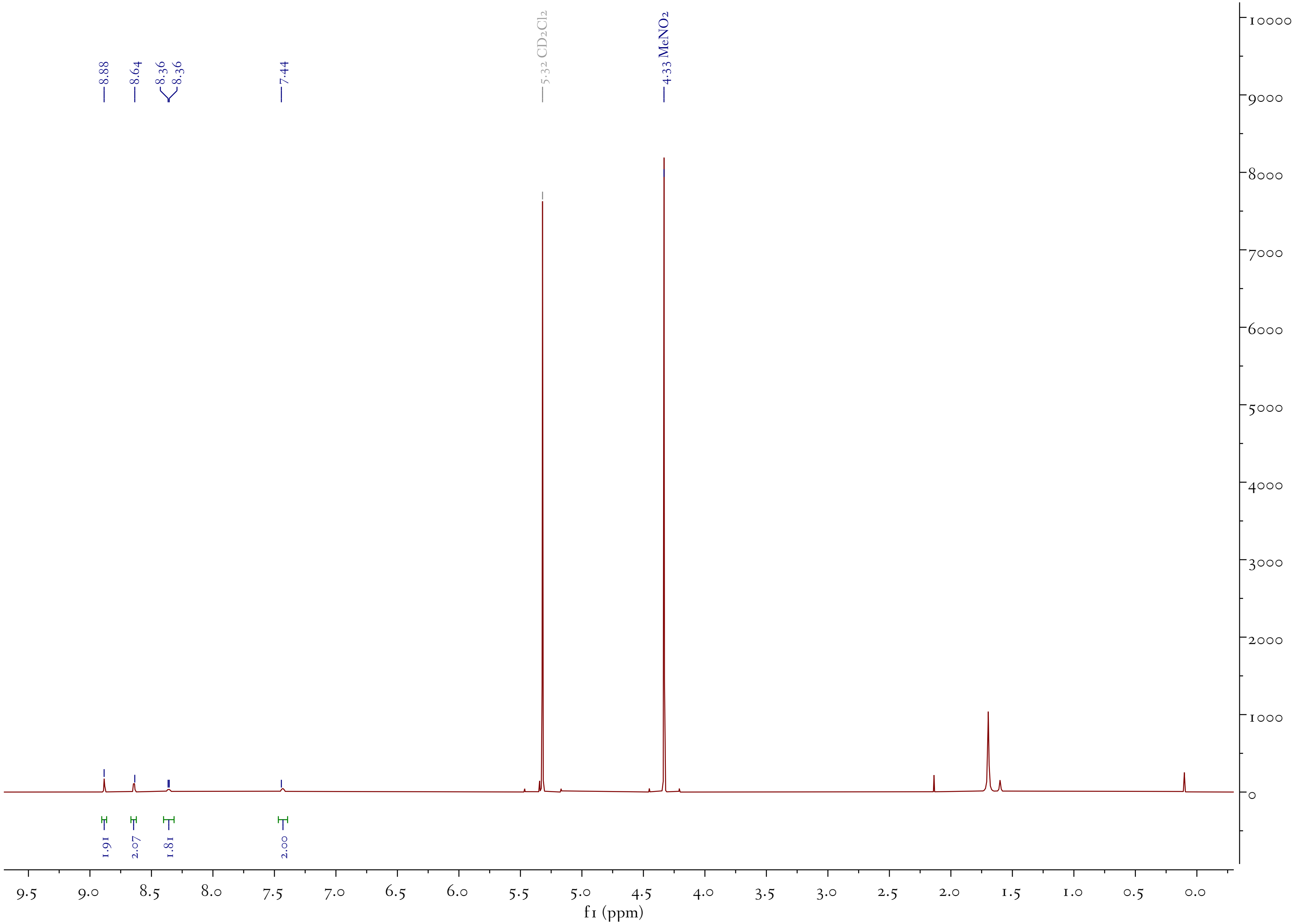
8.88
8.64
8.36
8.36
7.44
5.32 CD2Cl2
4.33 MeNO2
1.91
2.07
1.81
2.00
f1 (ppm)

Figure S18. The ^{1}H spectrum of [**5**–Ag–**5**]$^{+}$ in CD_2Cl_2.

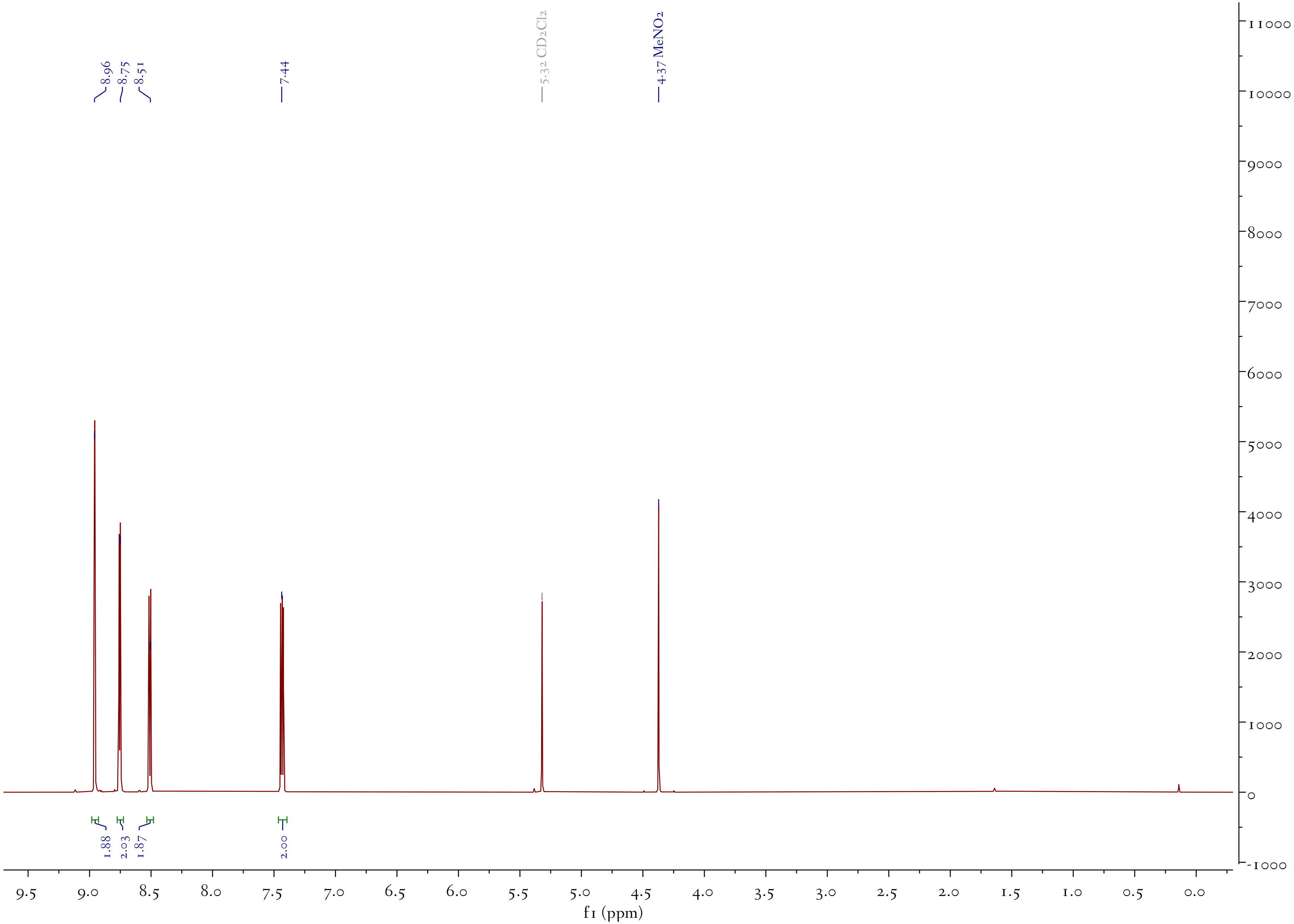
8.96
8.75
8.51
7.44
5.32 CD2Cl2
4.37 MeNO2
1.88
2.03
1.87
2.00
11000
10000
9000
8000
7000
6000
5000
4000
3000
2000
1000
0
-1000
9.5
9.0
8.5
8.0
7.5
7.0
6.5
6.0
5.5
5.0
4.5
4.0
3.5
3.0
2.5
2.0
1.5
1.0
0.5
0.0
f1 (ppm)

Figure S19. The ^{1}H spectrum of $[\mathbf{5}–I–\mathbf{5}]^+$ in CD_2Cl_2.

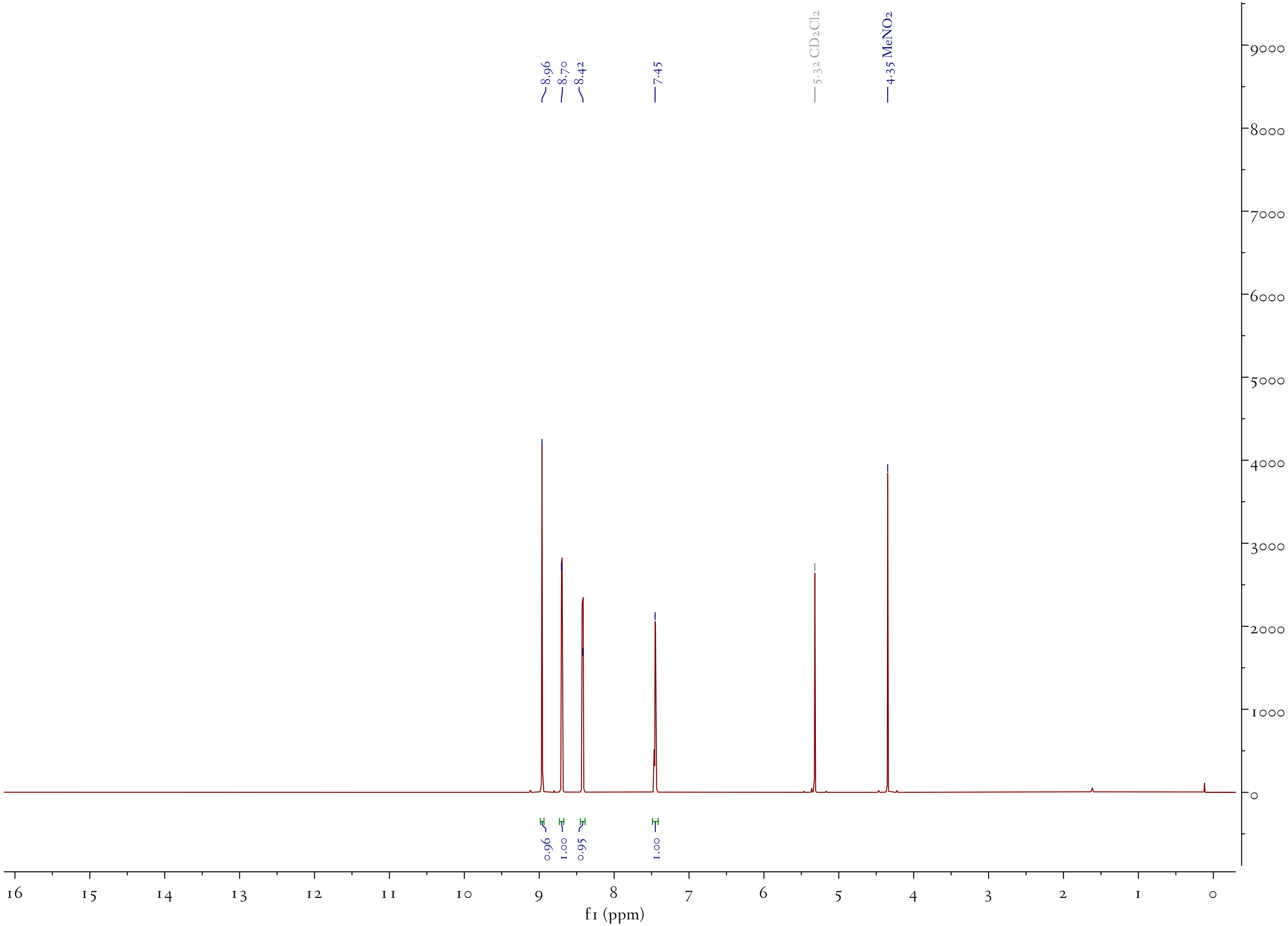
8.96
8.70
8.42
7.45
5.32 CD2Cl2
4.35 MeNO2
0.96
1.00
0.95
1.00
f1 (ppm)

Figure S20. The ^{1}H spectrum of [**5**H]$^+$ in CD_2Cl_2

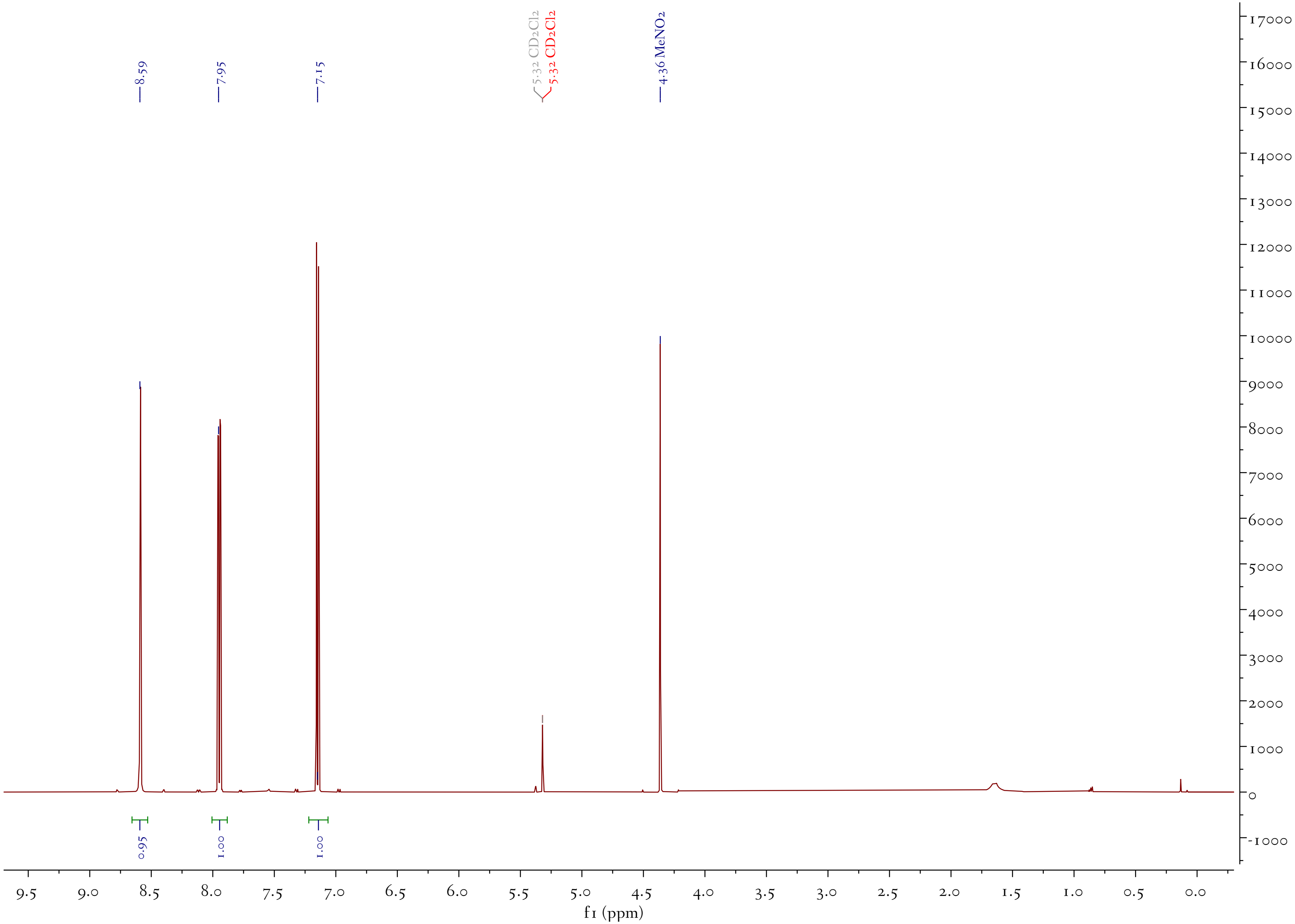

8.59
7.95
7.15
5.32 CD2Cl2
5.32 CD2Cl2
4.36 MeNO2
0.95
1.00
1.00
f1 (ppm)

Figure S21. The ^{1}H spectrum of **6** in CD_2Cl_2.

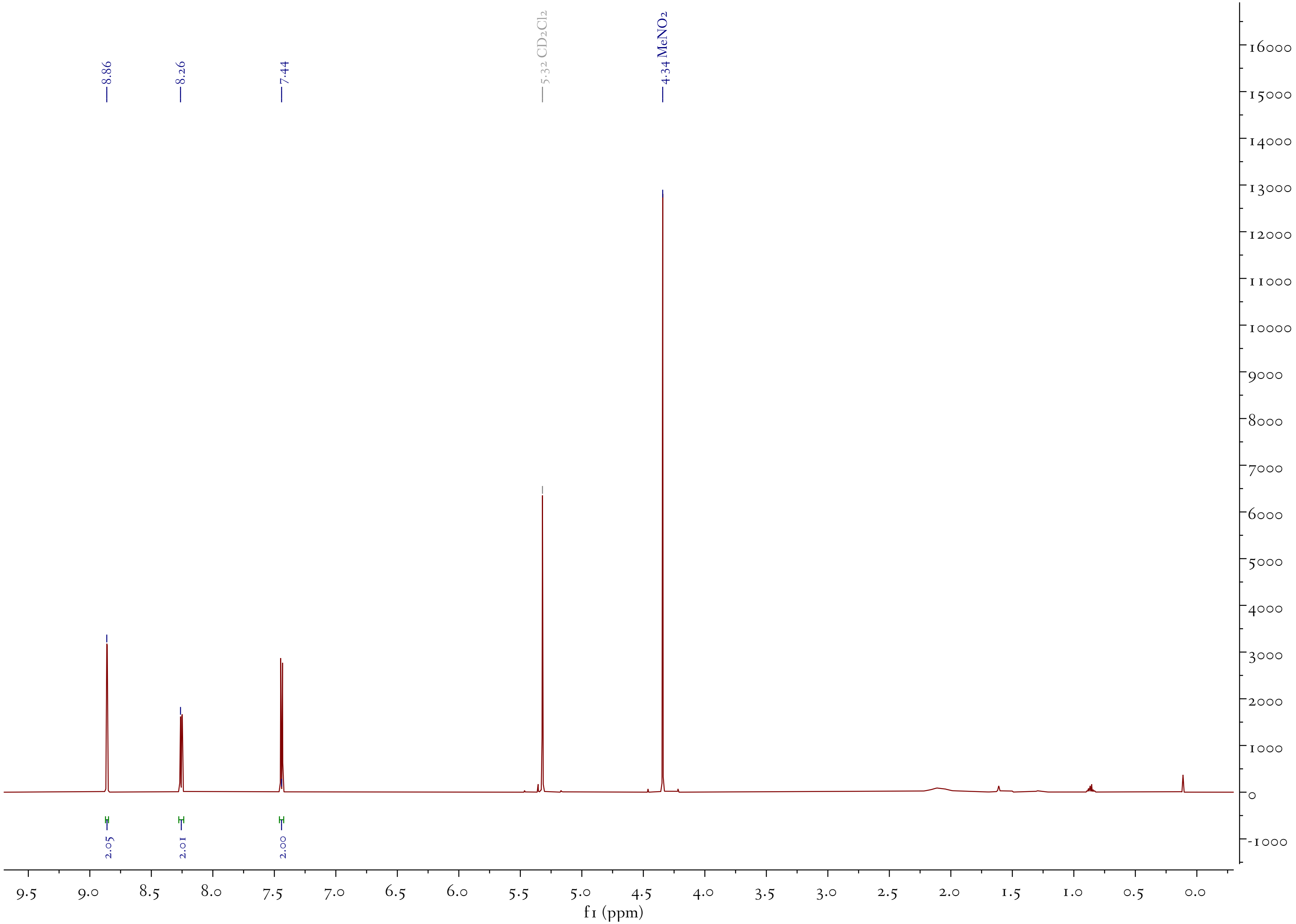

8.86
8.26
7.44
5.32 CD2Cl2
4.34 MeNO2
2.05
2.01
2.00
16000
15000
14000
13000
12000
11000
10000
9000
8000
7000
6000
5000
4000
3000
2000
1000
0
-1000
9.5
9.0
8.5
8.0
7.5
7.0
6.5
6.0
5.5
5.0
4.5
4.0
3.5
3.0
2.5
2.0
1.5
1.0
0.5
0.0
f1 (ppm)

Figure S22. The ^{1}H spectrum of [**6**–Ag–**6**]$^+$ in CD_2Cl_2.

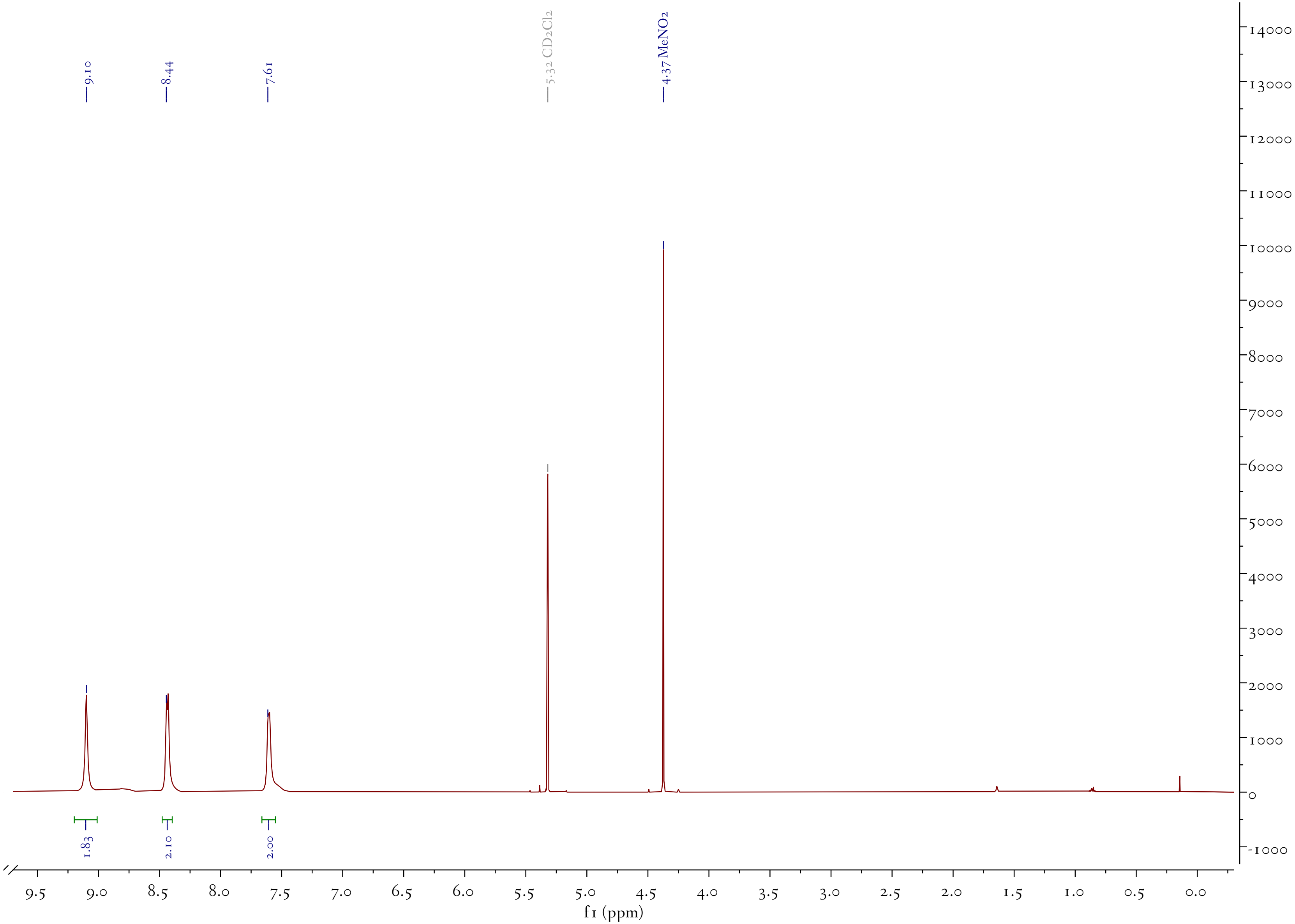

9.10
8.44
7.61
5.32 CD2Cl2
4.37 MeNO2
1.83
2.10
2.00
14000
13000
12000
11000
10000
9000
8000
7000
6000
5000
4000
3000
2000
1000
0
-1000
9.5
9.0
8.5
8.0
7.5
7.0
6.5
6.0
5.5
5.0
4.5
4.0
3.5
3.0
2.5
2.0
1.5
1.0
0.5
0.0
f1 (ppm)

Figure S23. The ^{1}H spectrum of [**6**–I–**6**]$^{+}$ in CD_2Cl_2.

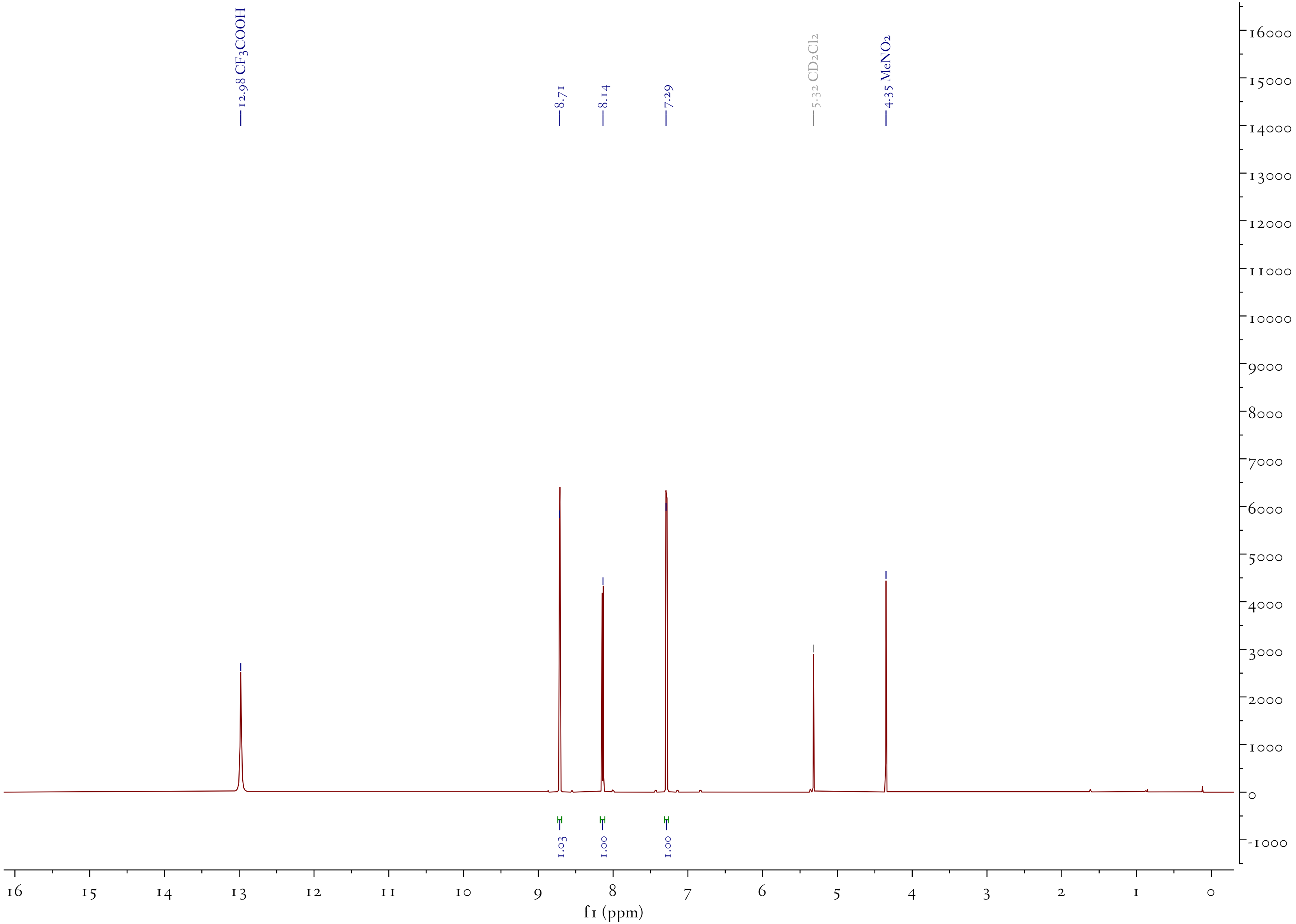
12.98 CF3COOH
8.71
8.14
7.29
5.32 CD2Cl2
4.35 MeNO2
1.03
1.00
1.00
f1 (ppm)
16000
15000
14000
13000
12000
11000
10000
9000
8000
7000
6000
5000
4000
3000
2000
1000
0
-1000
16
15
14
13
12
11
10
9
8
7
6
5
4
3
2
1
0

Figure S24. The ^{1}H spectrum of [**6**H]$^{+}$ in CD_2Cl_2

2.2 ^{1}H–^{15}N HMBC spectra

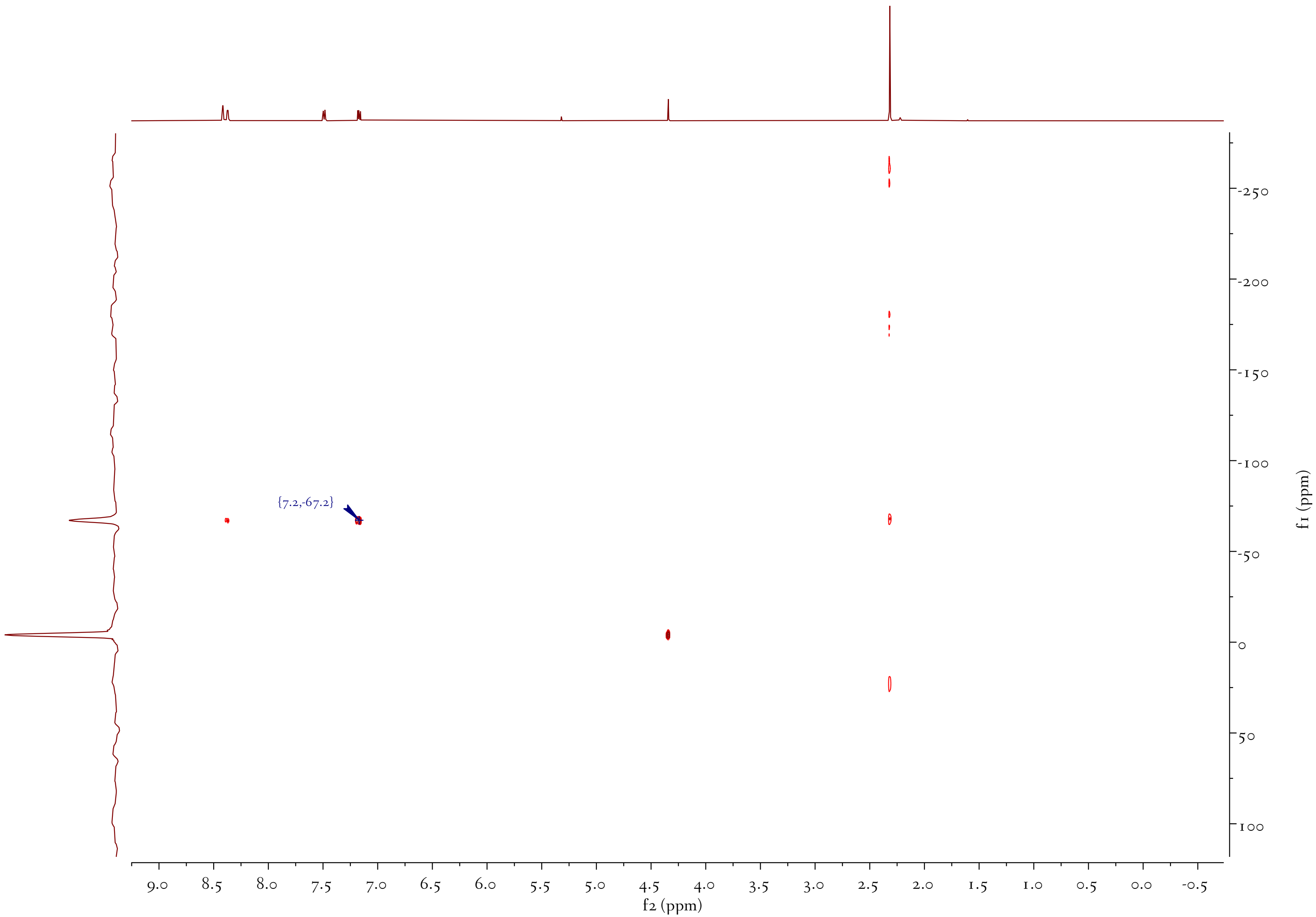

Figure S25. The ¹H–¹⁵N HMBC spectrum of **1** in CD_2Cl_2.

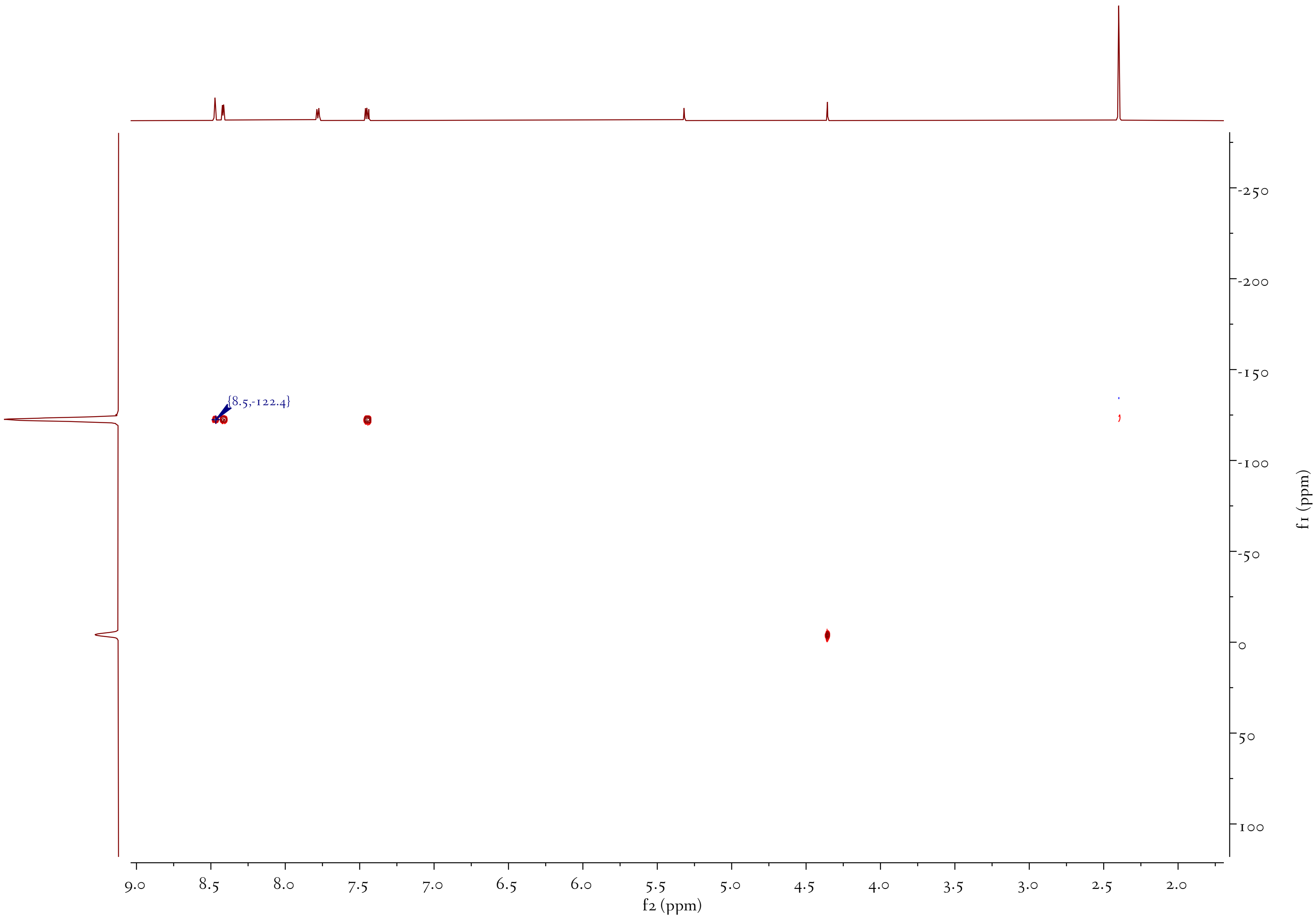
{8.5,-122.4}
-250
-200
-150
-100
-50
0
50
100
f1 (ppm)
9.0
8.5
8.0
7.5
7.0
6.5
6.0
5.5
5.0
4.5
4.0
3.5
3.0
2.5
2.0
f2 (ppm)

Figure S26. The ^{1}H–^{15}N HMBC spectrum of [**1**–Ag–**1**]$^{+}$ in CD_2Cl_2.

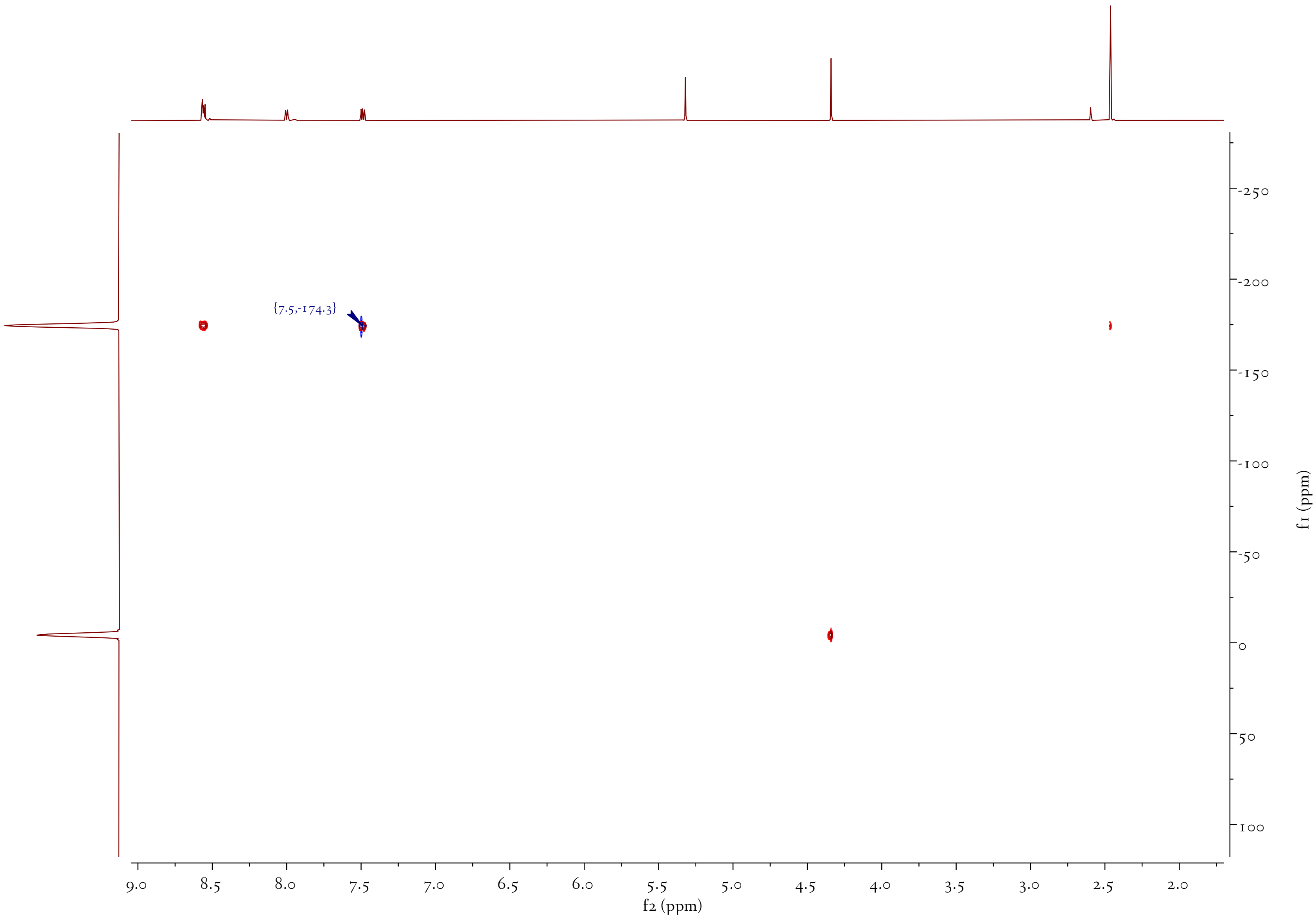
{7.5,-174.3}
f2 (ppm)
f1 (ppm)
9.0
8.5
8.0
7.5
7.0
6.5
6.0
5.5
5.0
4.5
4.0
3.5
3.0
2.5
2.0
-250
-200
-150
-100
-50
0
50
100

Figure S27. The ^1H–^{15}N HMBC spectrum of [**1**–I–**1**]$^+$ in CD_2Cl_2.

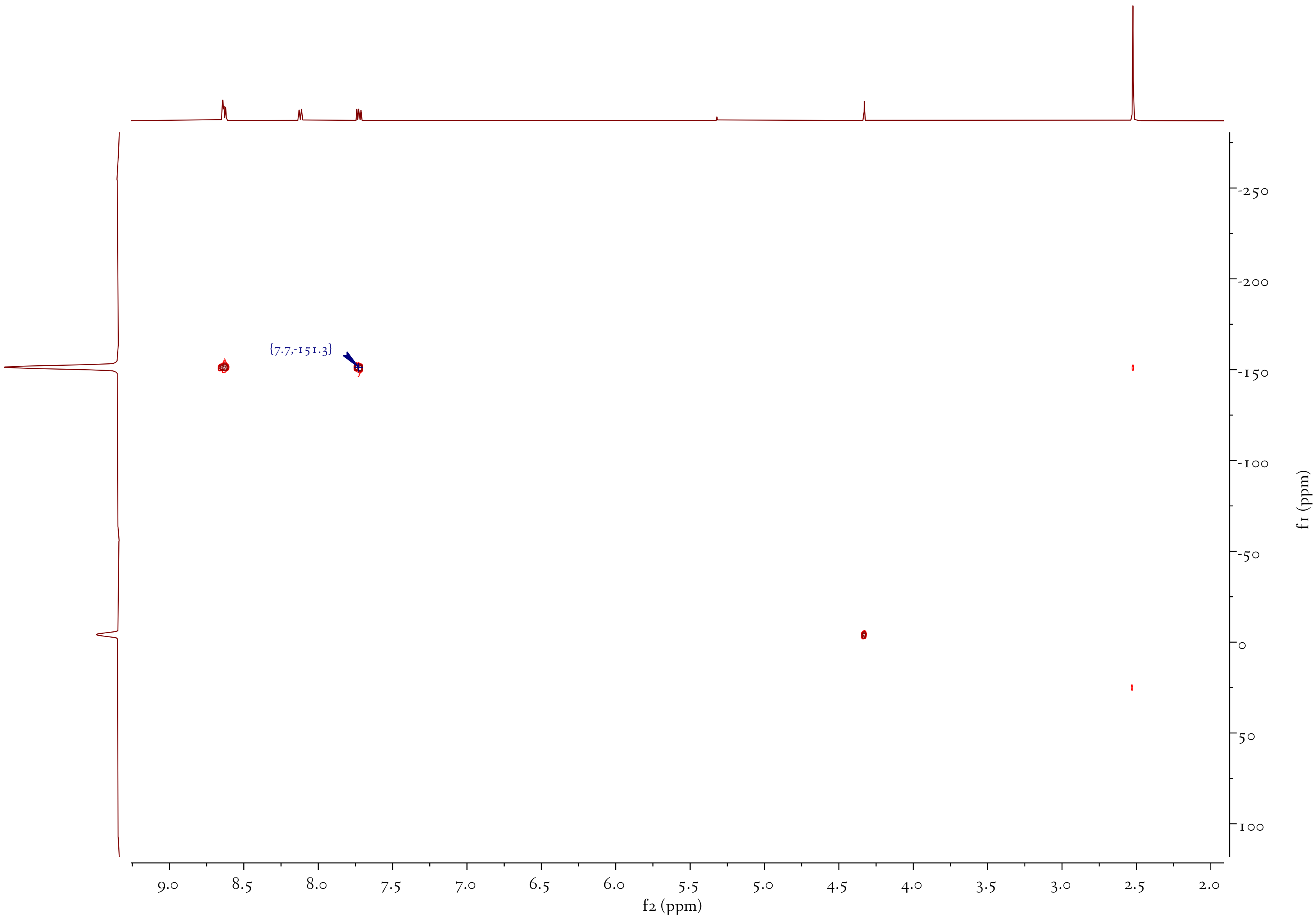
{7.7,-151.3}
-250
-200
-150
-100
-50
0
50
100
f1 (ppm)
9.0
8.5
8.0
7.5
7.0
6.5
6.0
5.5
5.0
4.5
4.0
3.5
3.0
2.5
2.0
f2 (ppm)

Figure S28. The ^{1}H–^{15}N HMBC spectrum of [**1**H]$^{+}$ in CD_2Cl_2.

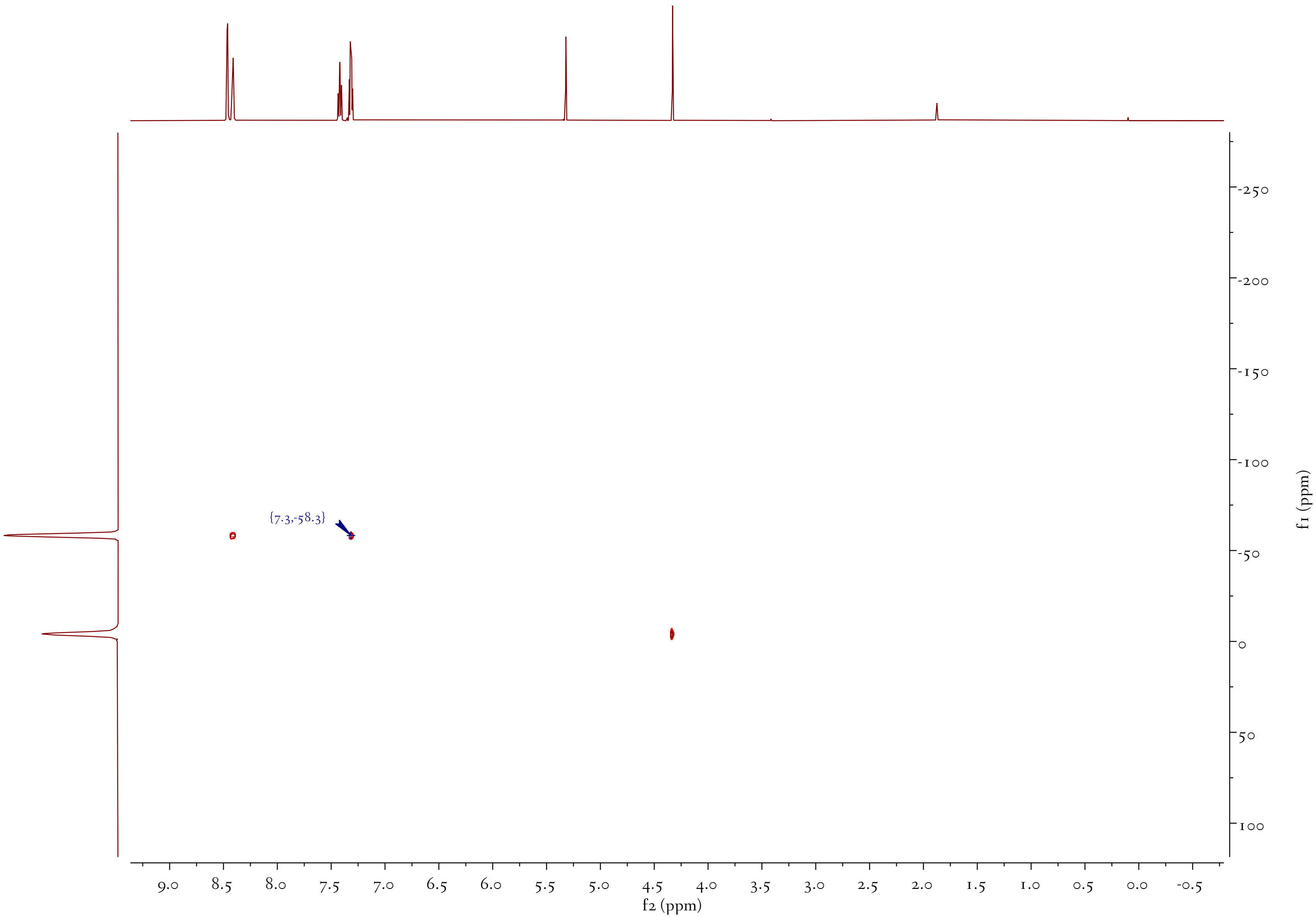
{7.3,-58.3}
f2 (ppm)
f1 (ppm)

Figure S29. The ^1H–^{15}N HMBC spectrum of **2** in CD_2Cl_2.

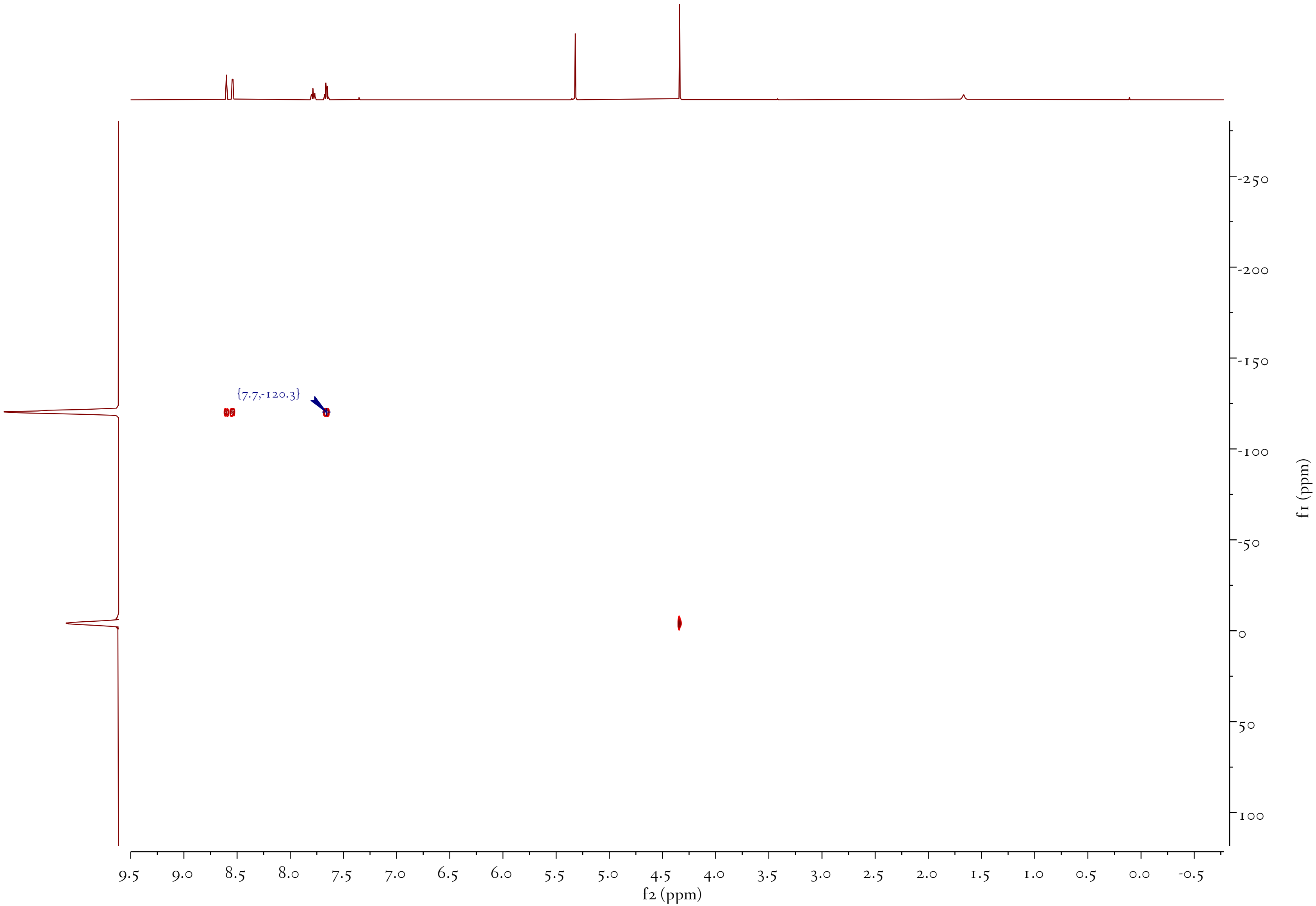

{7.7,-120.3}
f2 (ppm)
f1 (ppm)

Figure S30. The ^{1}H–^{15}N HMBC spectrum of [**2**–Ag–**2**]$^{+}$ in CD_2Cl_2.

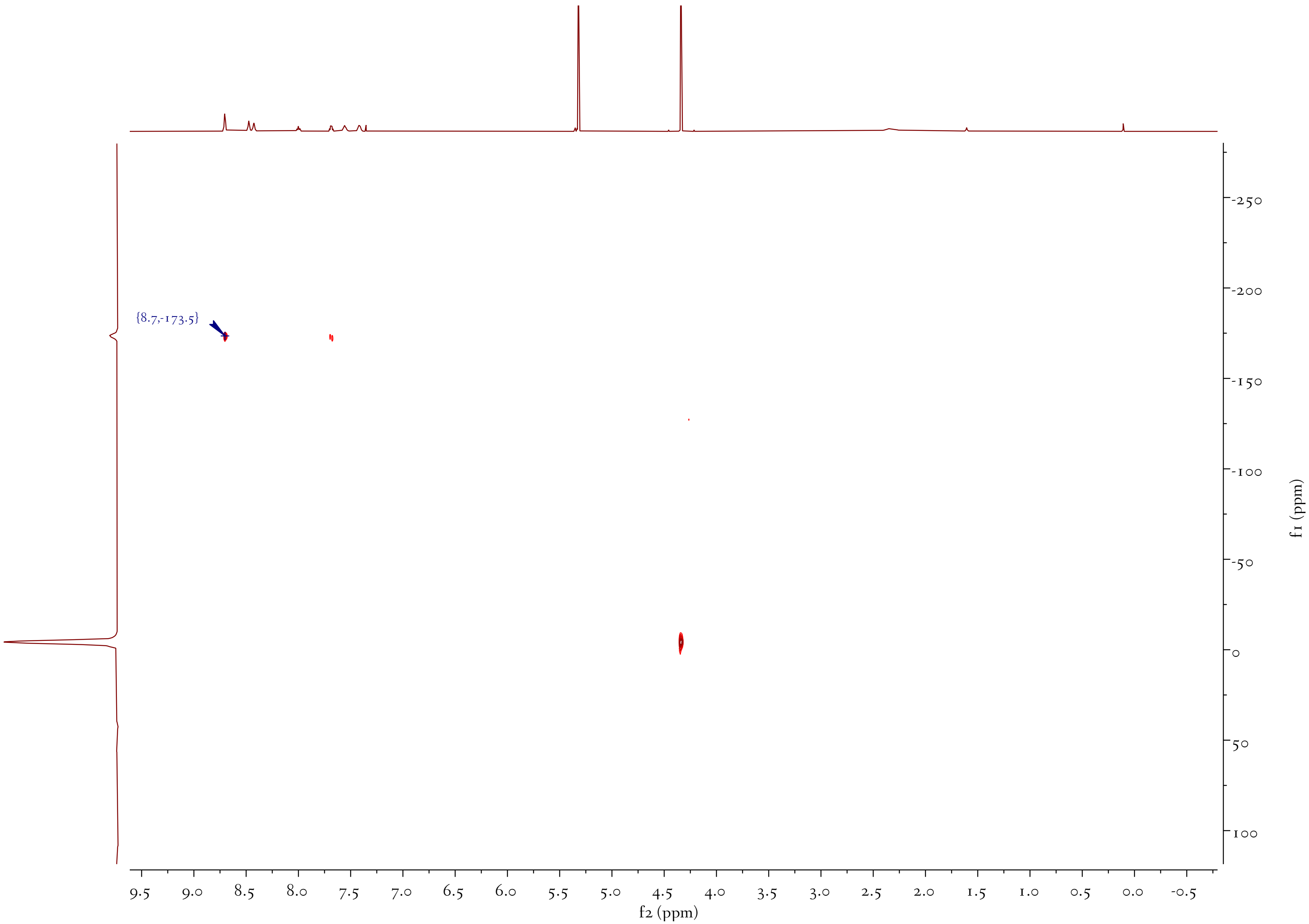
{8.7,-173.5}
-250
-200
-150
-100
-50
0
50
100
f1 (ppm)
9.5
9.0
8.5
8.0
7.5
7.0
6.5
6.0
5.5
5.0
4.5
4.0
3.5
3.0
2.5
2.0
1.5
1.0
0.5
0.0
-0.5
f2 (ppm)

Figure S31. The ^{1}H–^{15}N HMBC spectrum of [**2**–I–**2**]$^{+}$ in CD_2Cl_2.

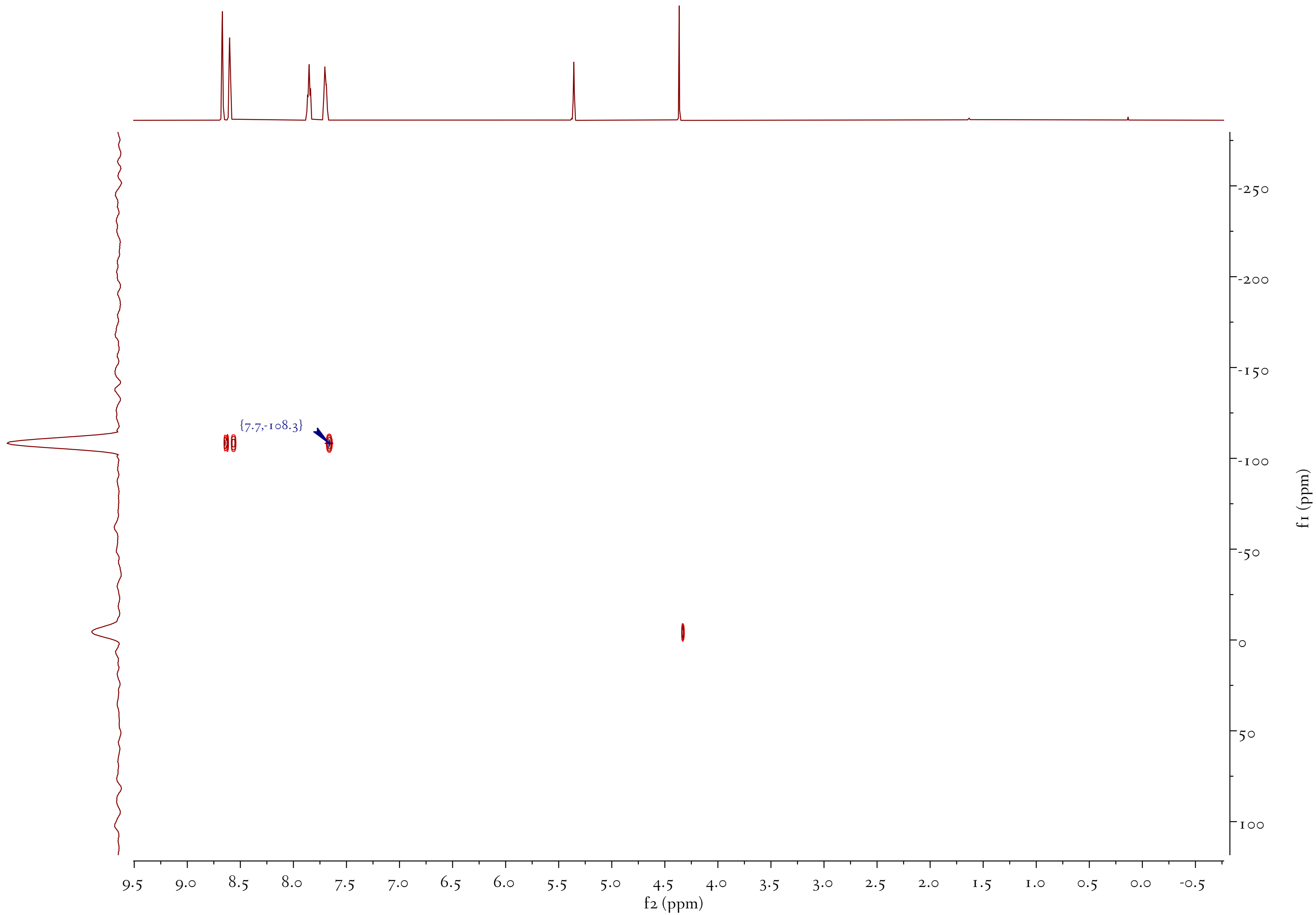

{7.7,-108.3}
-250
-200
-150
-100
-50
0
50
100
f1 (ppm)
9.5 9.0 8.5 8.0 7.5 7.0 6.5 6.0 5.5 5.0 4.5 4.0 3.5 3.0 2.5 2.0 1.5 1.0 0.5 0.0 -0.5
f2 (ppm)

Figure S32. The ^{1}H–^{15}N HMBC spectrum of [**2**H]$^{+}$ in CD_2Cl_2.

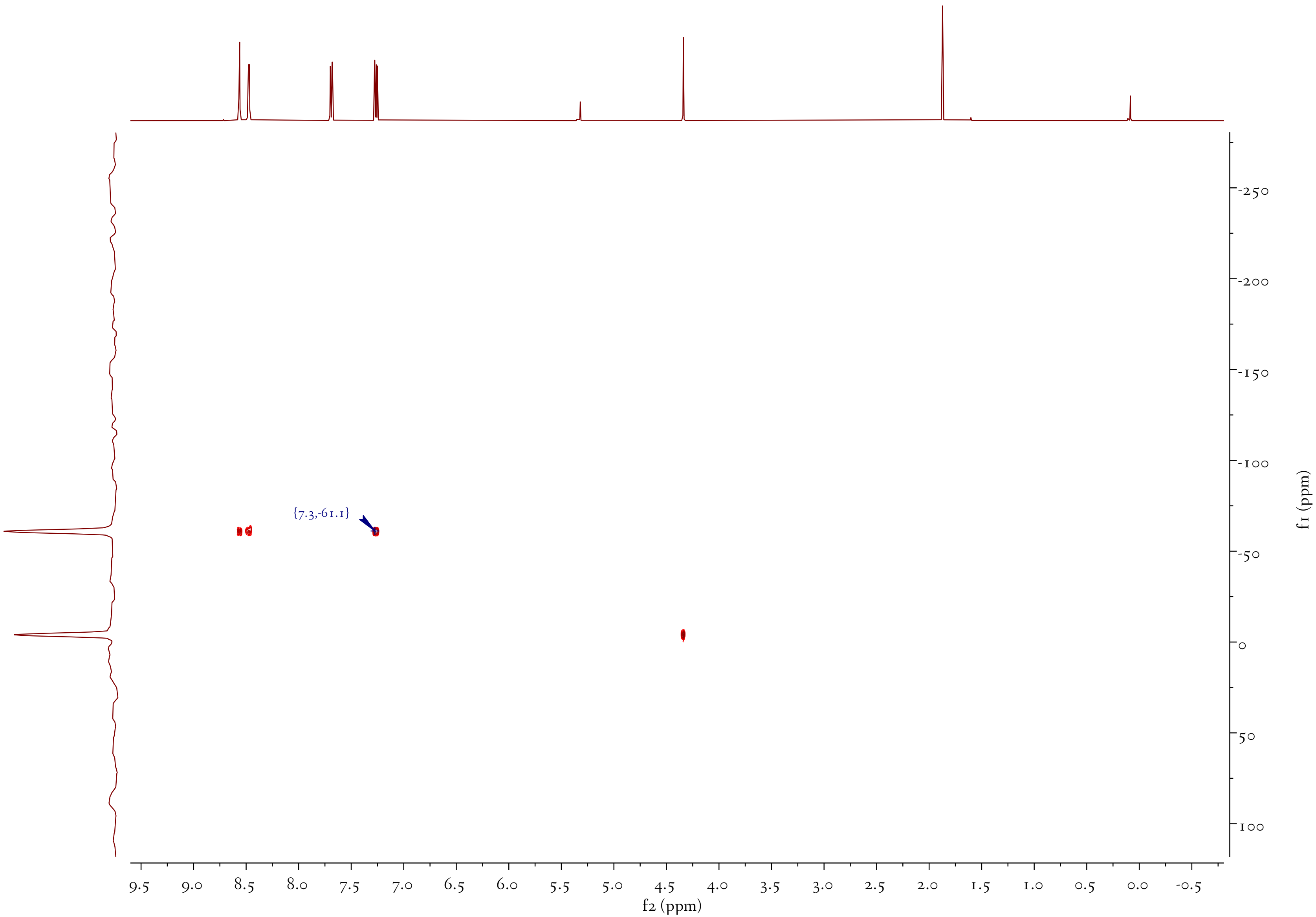
{7.3,-61.1}
-250
-200
-150
-100
-50
0
50
100
f1 (ppm)
9.5 9.0 8.5 8.0 7.5 7.0 6.5 6.0 5.5 5.0 4.5 4.0 3.5 3.0 2.5 2.0 1.5 1.0 0.5 0.0 -0.5
f2 (ppm)

Figure S33. The ^{1}H–^{15}N HMBC spectrum of **3** in CD_2Cl_2.

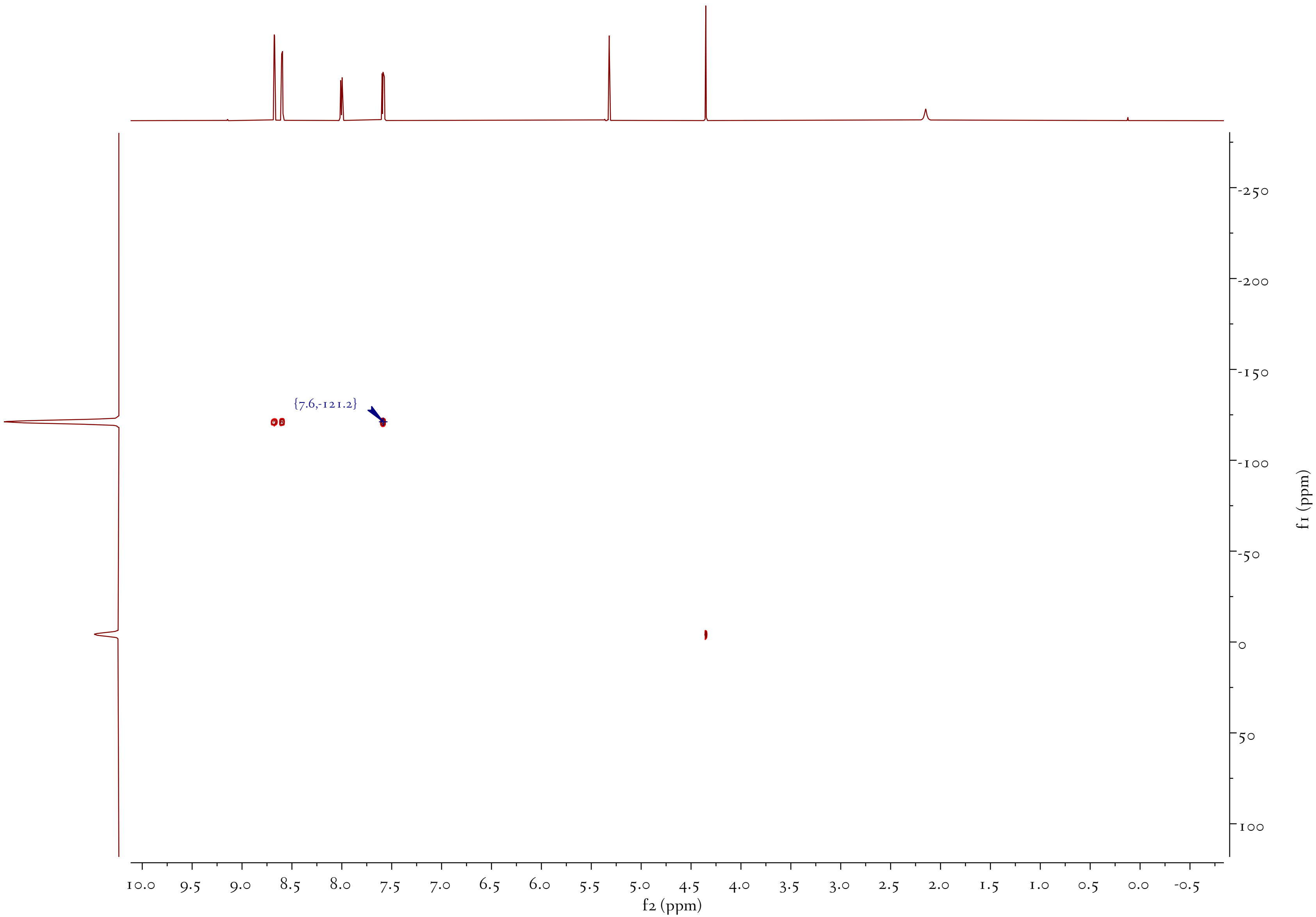
{7.6,-121.2}
f2 (ppm)
f1 (ppm)

Figure S34. The ^{1}H–^{15}N HMBC spectrum of complex [**3**–Ag–**3**]$^{+}$ in CD_2Cl_2.

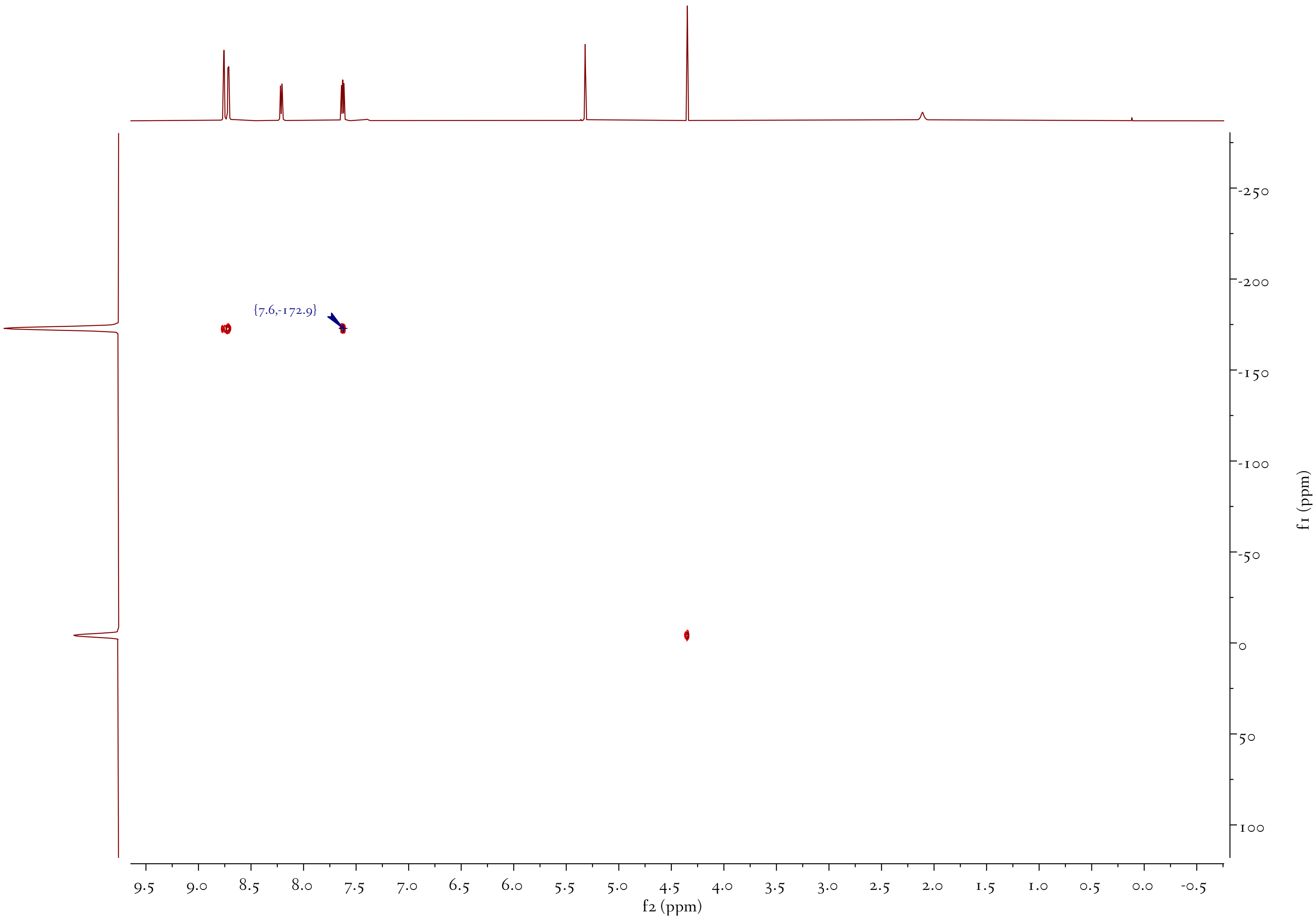
{7.6,-172.9}
-250
-200
-150
-100
-50
0
50
100
f1 (ppm)
9.5 9.0 8.5 8.0 7.5 7.0 6.5 6.0 5.5 5.0 4.5 4.0 3.5 3.0 2.5 2.0 1.5 1.0 0.5 0.0 -0.5
f2 (ppm)

Figure S35. The ^{1}H–^{15}N HMBC spectrum of complex [**3**–I–**3**]$^{+}$ in CD_2Cl_2.

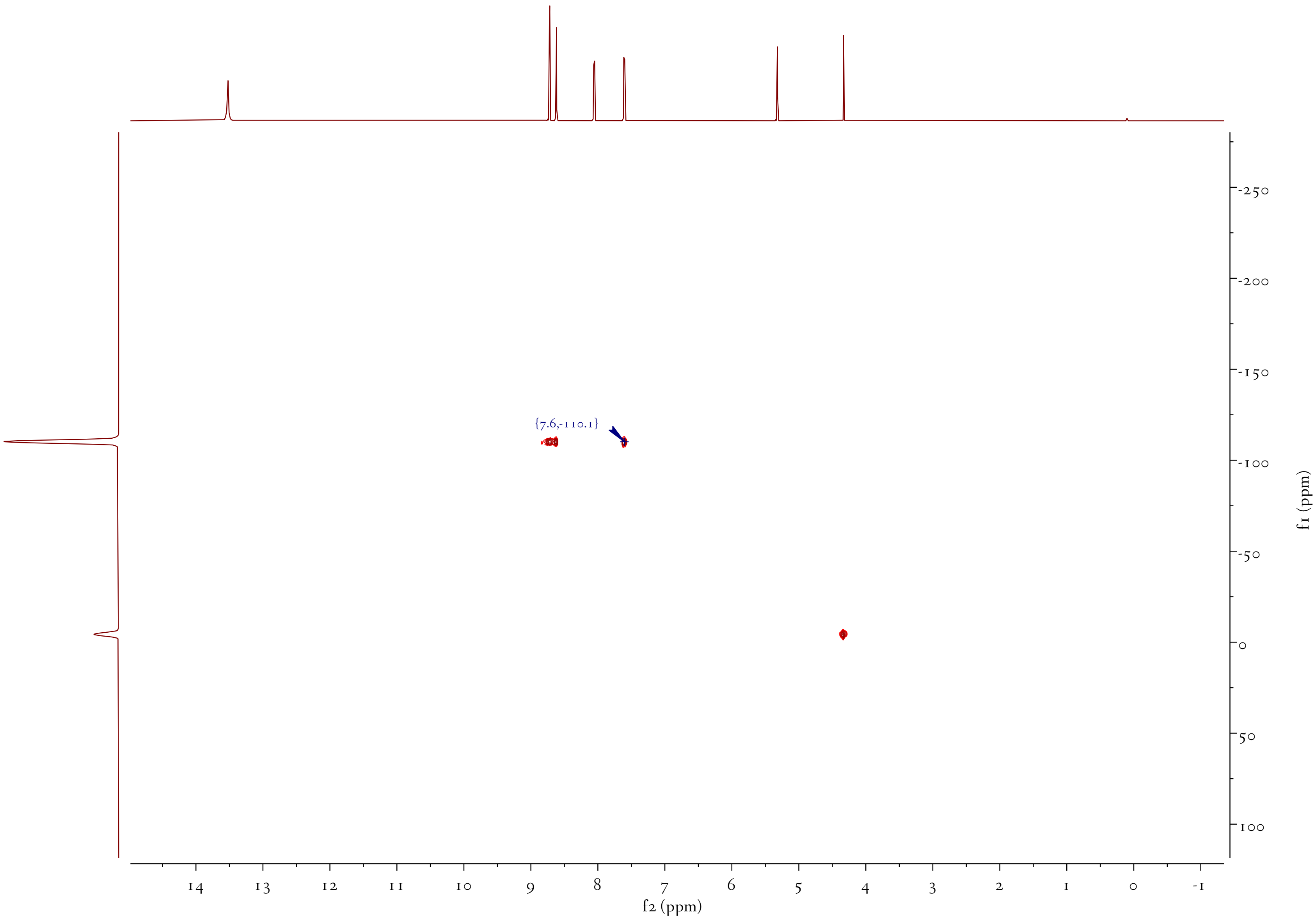
{7.6,-110.1}
-250
-200
-150
-100
-50
0
50
100
f1 (ppm)
14
13
12
11
10
9
8
7
6
5
4
3
2
1
0
-1
f2 (ppm)

Figure S36. The ^{1}H–^{15}N HMBC spectrum of [**3**H]$^{+}$ in CD_2Cl_2.

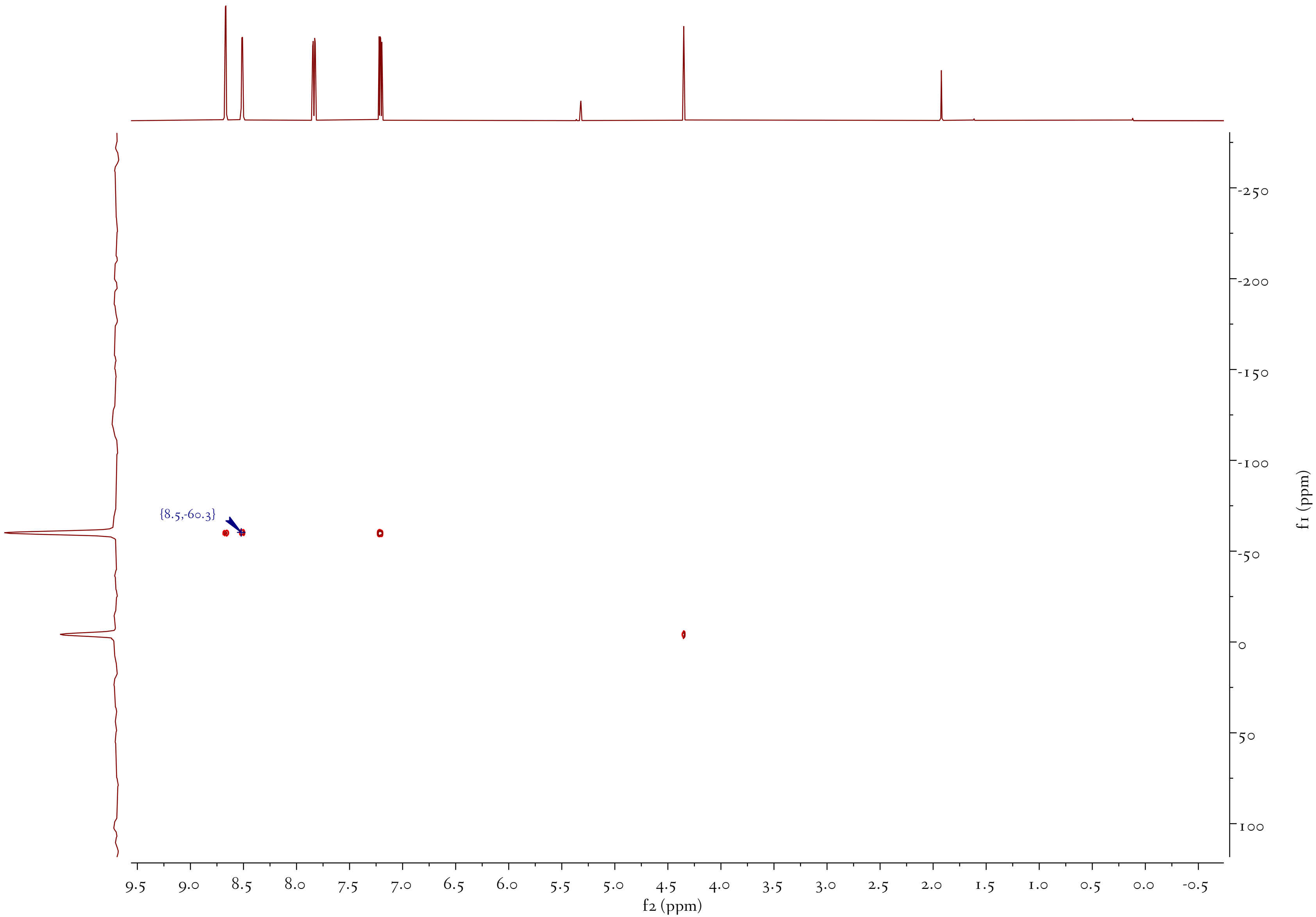
{8.5,-60.3}
-250
-200
-150
-100
-50
0
50
100
f1 (ppm)
9.5
9.0
8.5
8.0
7.5
7.0
6.5
6.0
5.5
5.0
4.5
4.0
3.5
3.0
2.5
2.0
1.5
1.0
0.5
0.0
-0.5
f2 (ppm)

Figure S37. The ^{1}H–^{15}N HMBC spectrum of **4** in CD_2Cl_2.

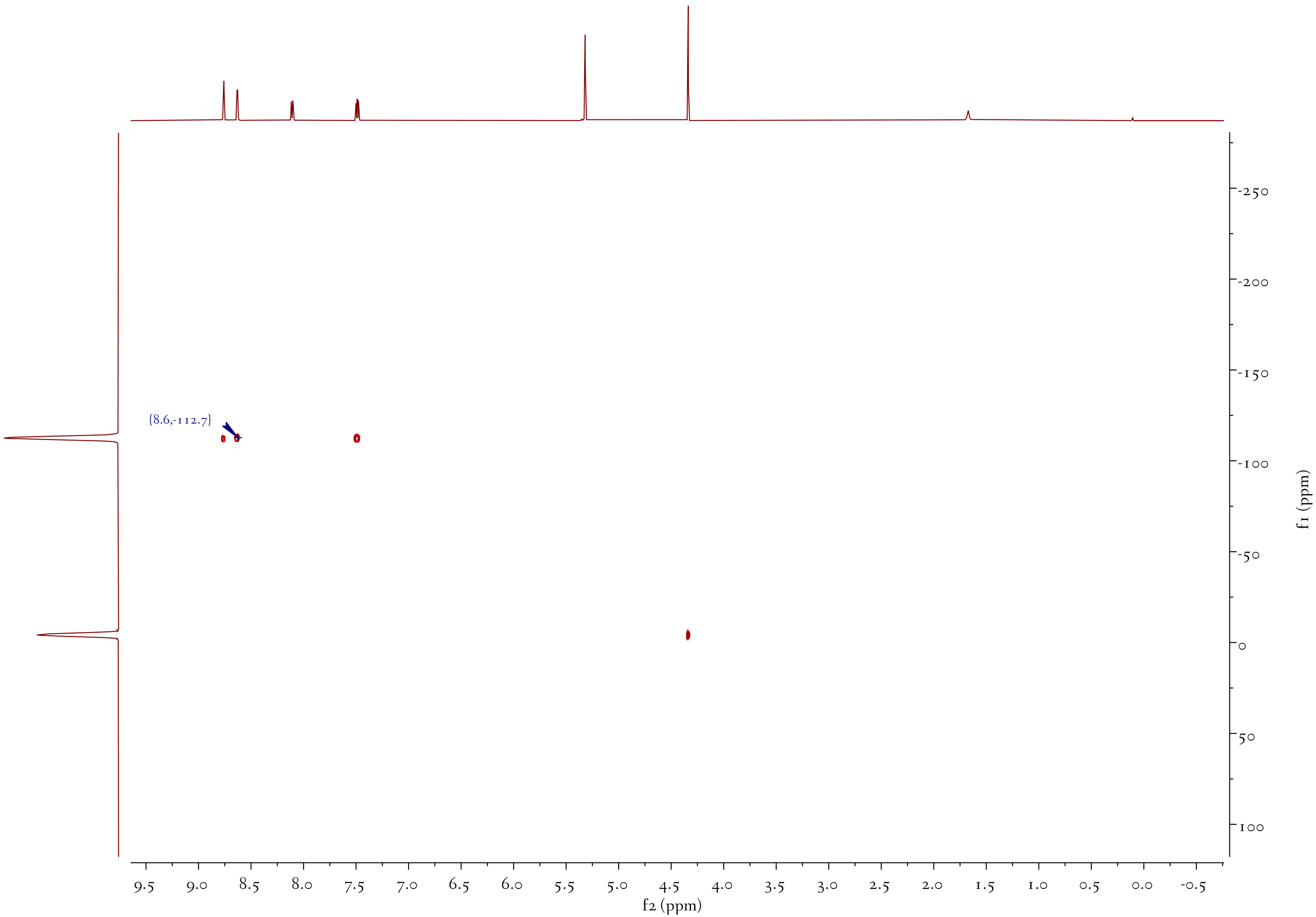
{8.6,-112.7}
-250
-200
-150
-100
-50
0
50
100
f1 (ppm)
9.5 9.0 8.5 8.0 7.5 7.0 6.5 6.0 5.5 5.0 4.5 4.0 3.5 3.0 2.5 2.0 1.5 1.0 0.5 0.0 -0.5
f2 (ppm)

Figure S38. The ^{1}H–^{15}N HMBC spectrum of [**4**–Ag–**4**]$^{+}$ in CD_2Cl_2.

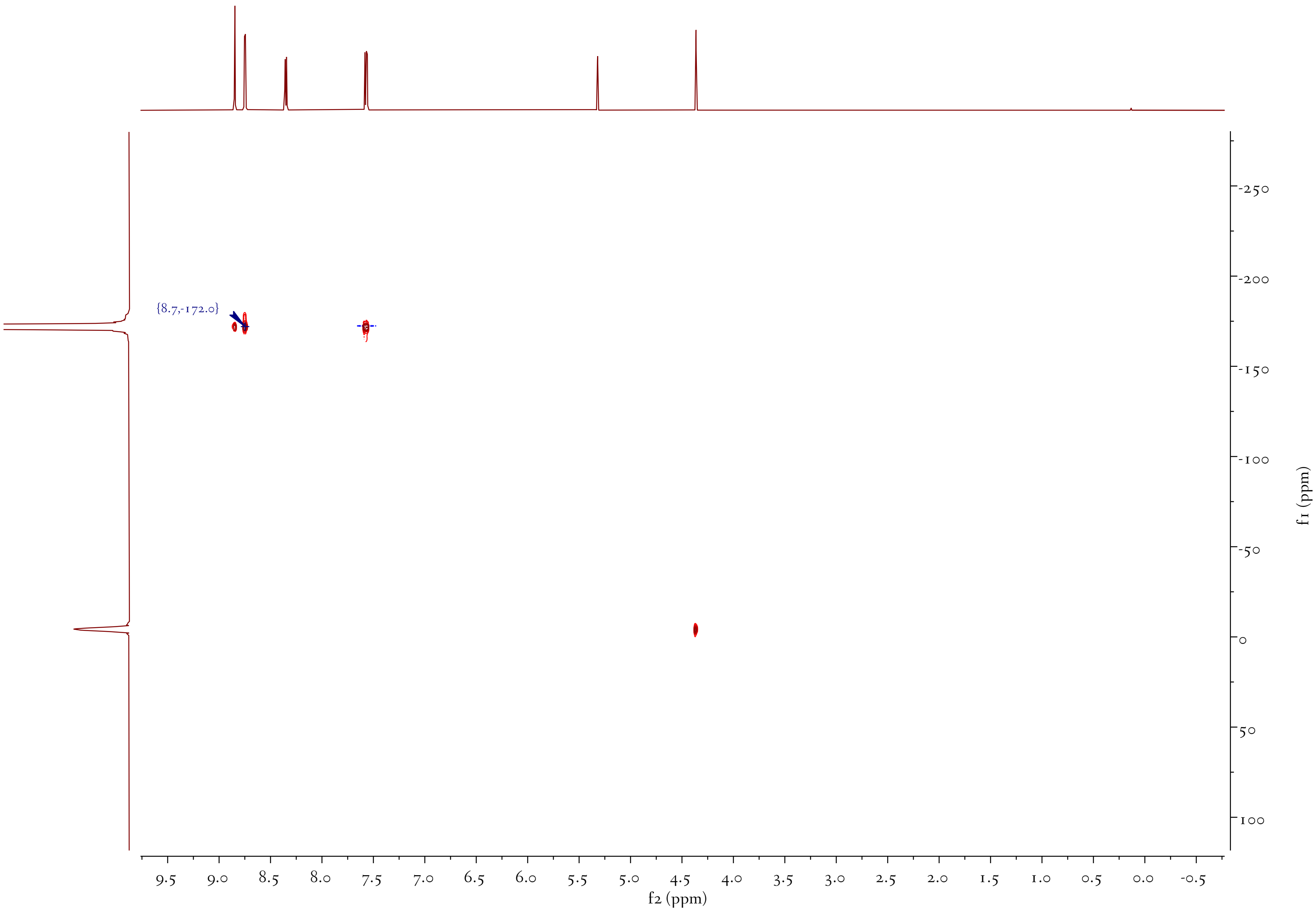
{8.7,-172.0}
-250
-200
-150
-100
-50
0
50
100
f1 (ppm)
9.5 9.0 8.5 8.0 7.5 7.0 6.5 6.0 5.5 5.0 4.5 4.0 3.5 3.0 2.5 2.0 1.5 1.0 0.5 0.0 -0.5
f2 (ppm)

Figure S39. The ^{1}H–^{15}N HMBC spectrum of [**4**–I–**4**]$^{+}$ in CD_2Cl_2.

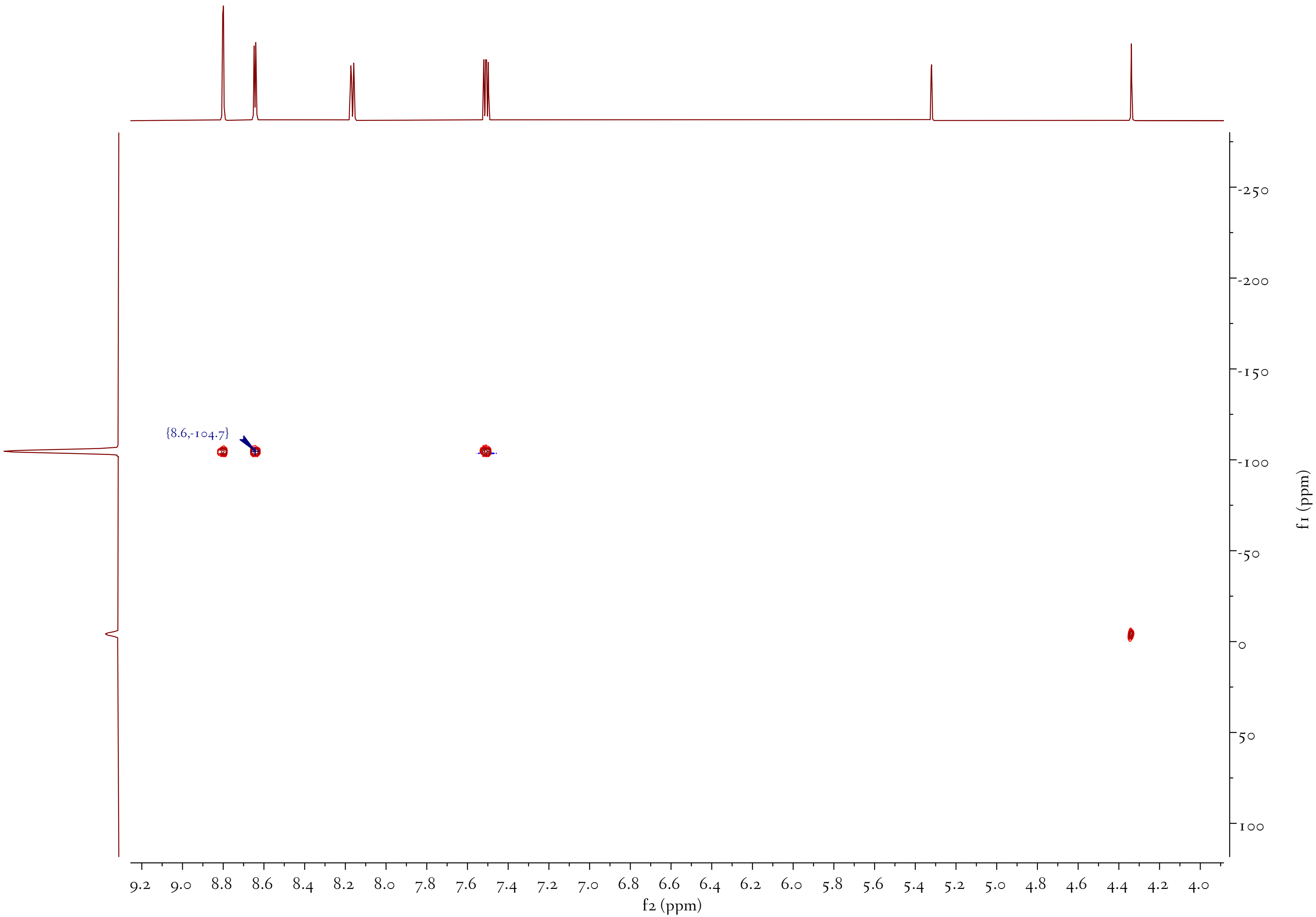
{8.6,-104.7}
-250
-200
-150
-100
-50
0
50
100
f1 (ppm)
9.2 9.0 8.8 8.6 8.4 8.2 8.0 7.8 7.6 7.4 7.2 7.0 6.8 6.6 6.4 6.2 6.0 5.8 5.6 5.4 5.2 5.0 4.8 4.6 4.4 4.2 4.0
f2 (ppm)

Figure S40. The ^{1}H–^{15}N HMBC spectrum of [**4**H]$^+$ in CD_2Cl_2.

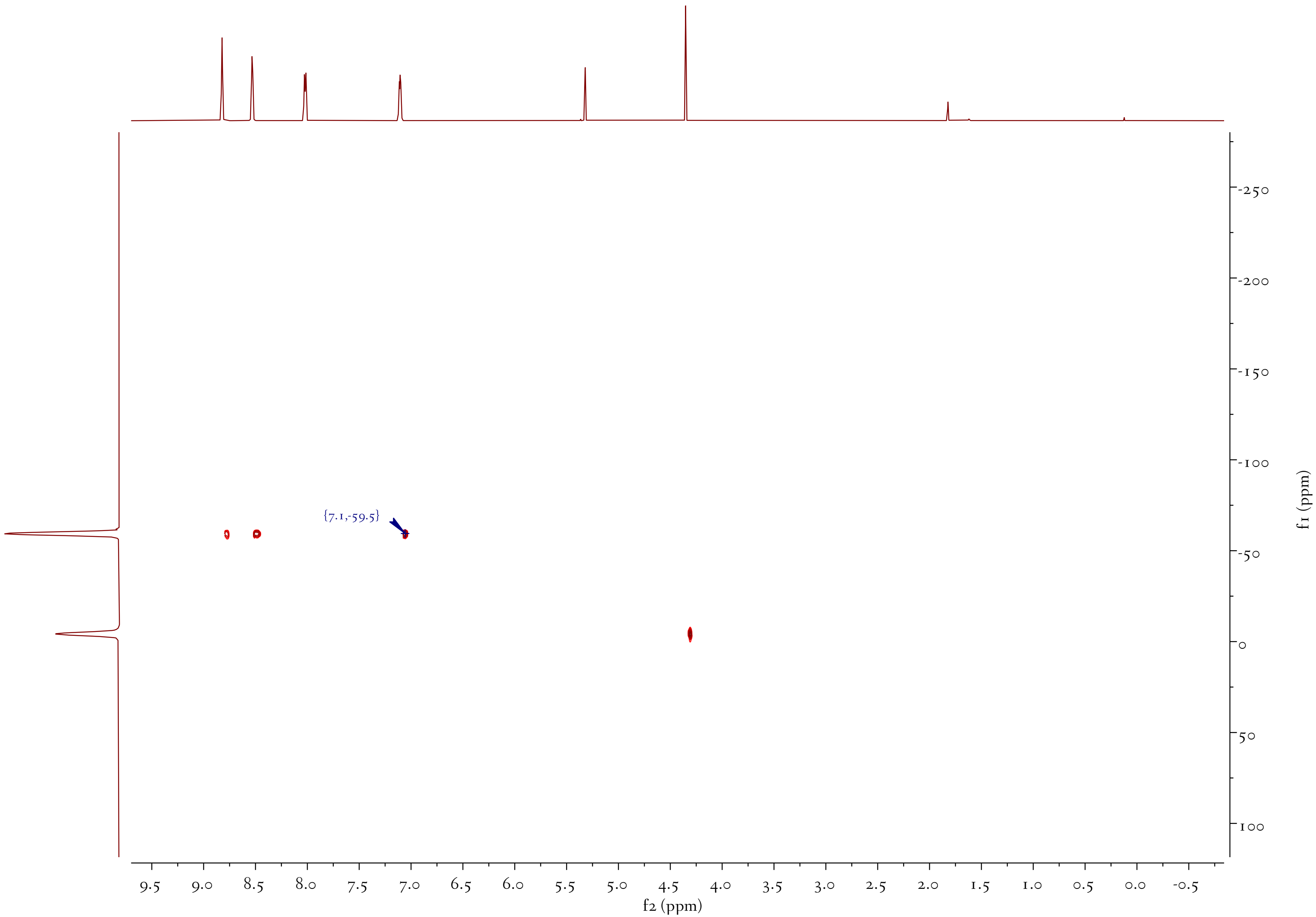

{7.1,-59.5}
-250
-200
-150
-100
-50
0
50
100
f1 (ppm)
9.5 9.0 8.5 8.0 7.5 7.0 6.5 6.0 5.5 5.0 4.5 4.0 3.5 3.0 2.5 2.0 1.5 1.0 0.5 0.0 -0.5
f2 (ppm)

Figure S41. The ^1H–^{15}N HMBC spectrum of **5** in CD_2Cl_2.

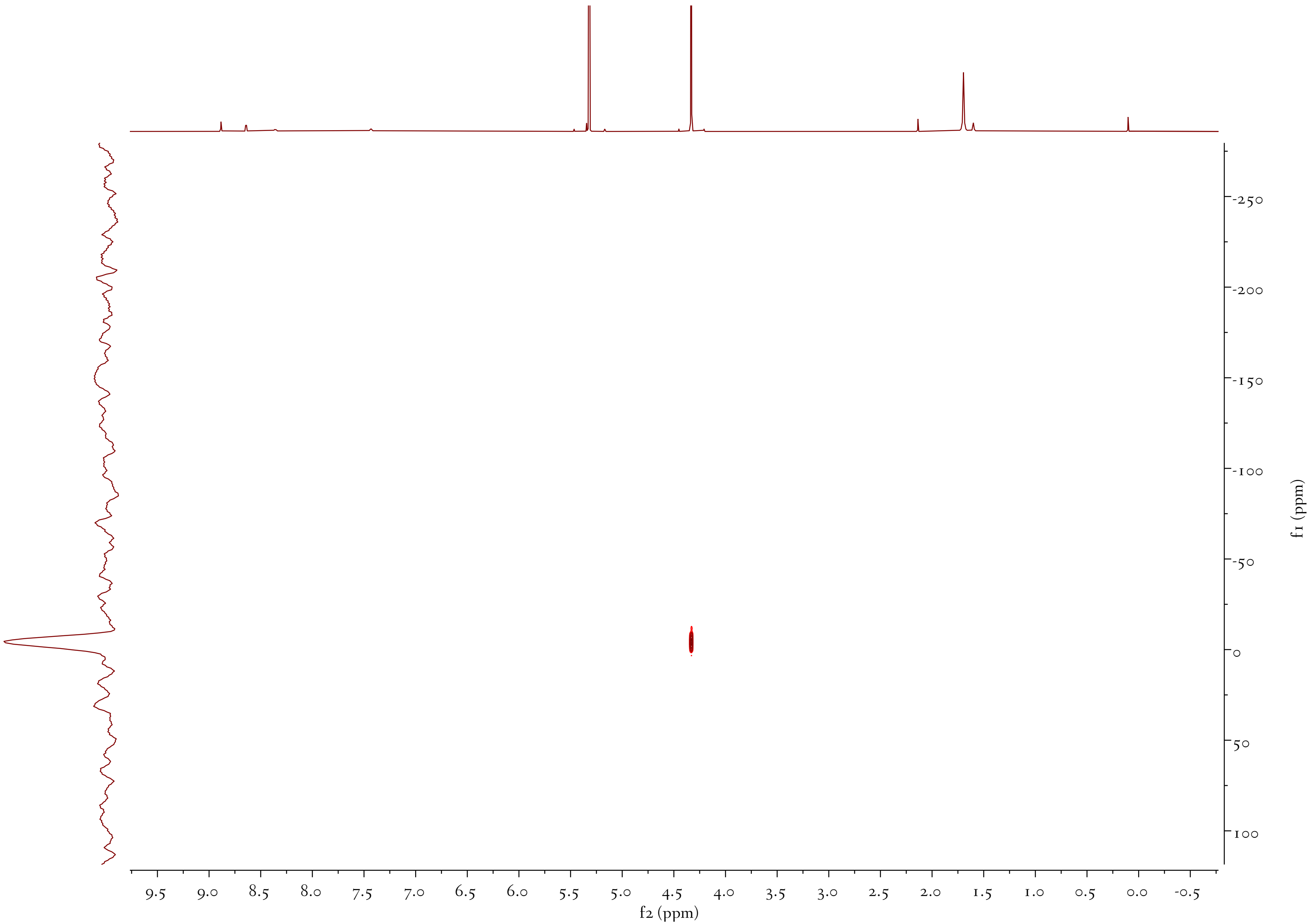
f2 (ppm)
f1 (ppm)

Figure S42. The ^1H–^{15}N HMBC spectrum of [**5**–Ag–**5**]$^+$ in CD_2Cl_2. No signals from the complex were detected at 298 K.

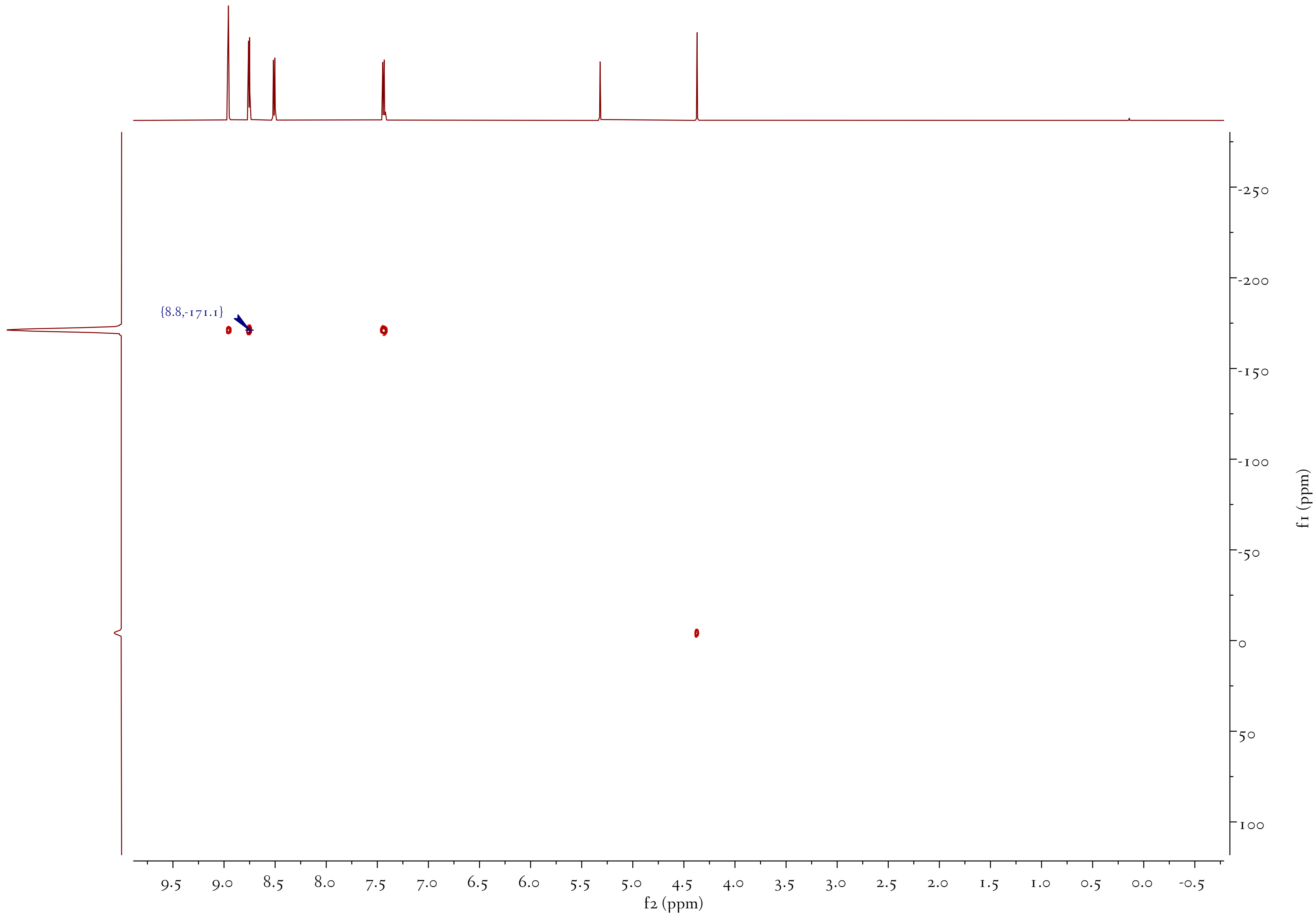

{8.8,-171.1}
9.5 9.0 8.5 8.0 7.5 7.0 6.5 6.0 5.5 5.0 4.5 4.0 3.5 3.0 2.5 2.0 1.5 1.0 0.5 0.0 -0.5
f2 (ppm)
-250 -200 -150 -100 -50 0 50 100
f1 (ppm)

Figure S43. The ^{1}H–^{15}N HMBC spectrum of [**5**–I–**5**]$^{+}$ in CD_2Cl_2.

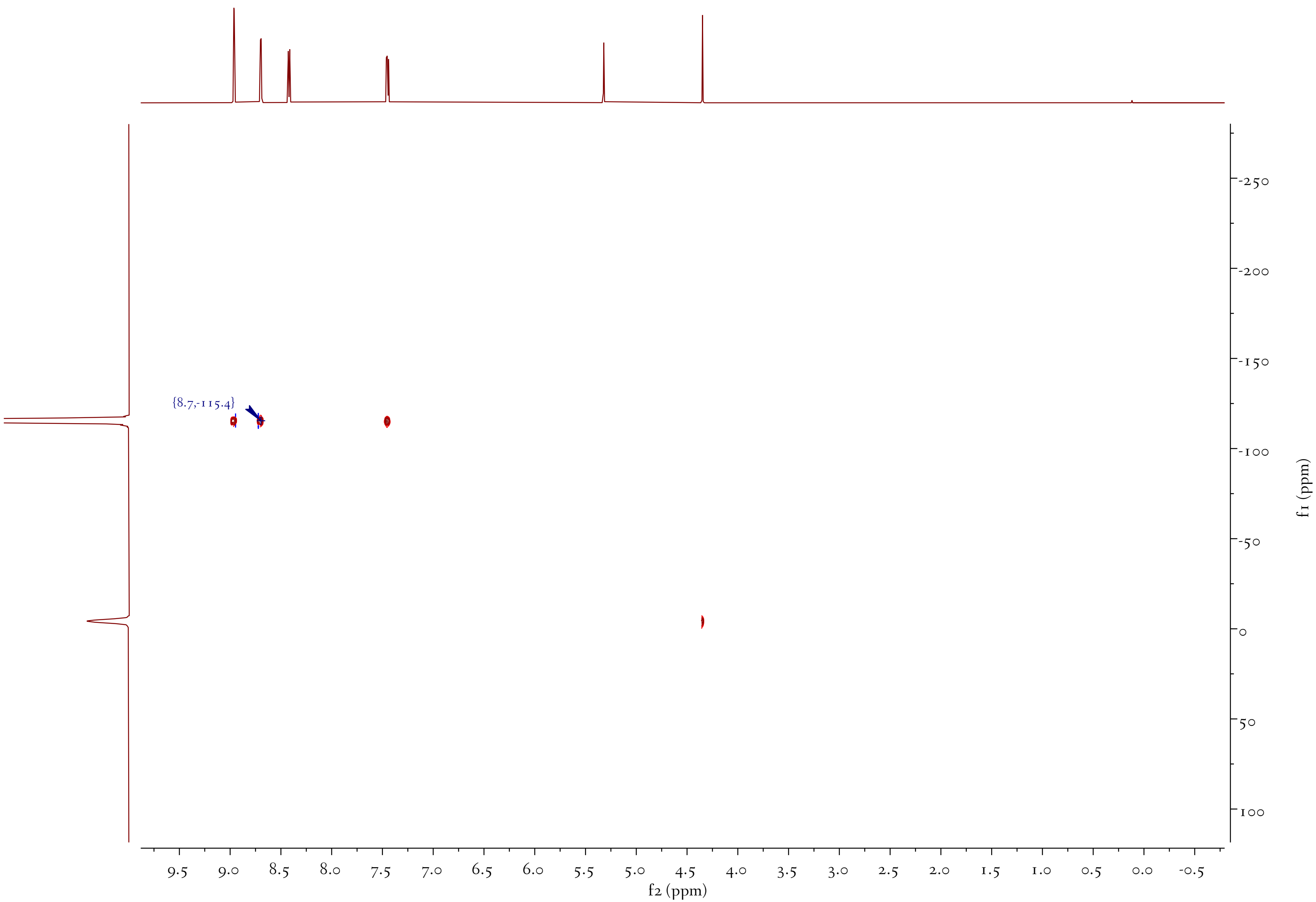
{8.7,-115.4}
9.5
9.0
8.5
8.0
7.5
7.0
6.5
6.0
5.5
5.0
4.5
4.0
3.5
3.0
2.5
2.0
1.5
1.0
0.5
0.0
-0.5
f2 (ppm)
-250
-200
-150
-100
-50
0
50
100
f1 (ppm)

Figure S44. The ^{1}H–^{15}N HMBC spectrum of [**5**H]$^{+}$ in CD_2Cl_2.

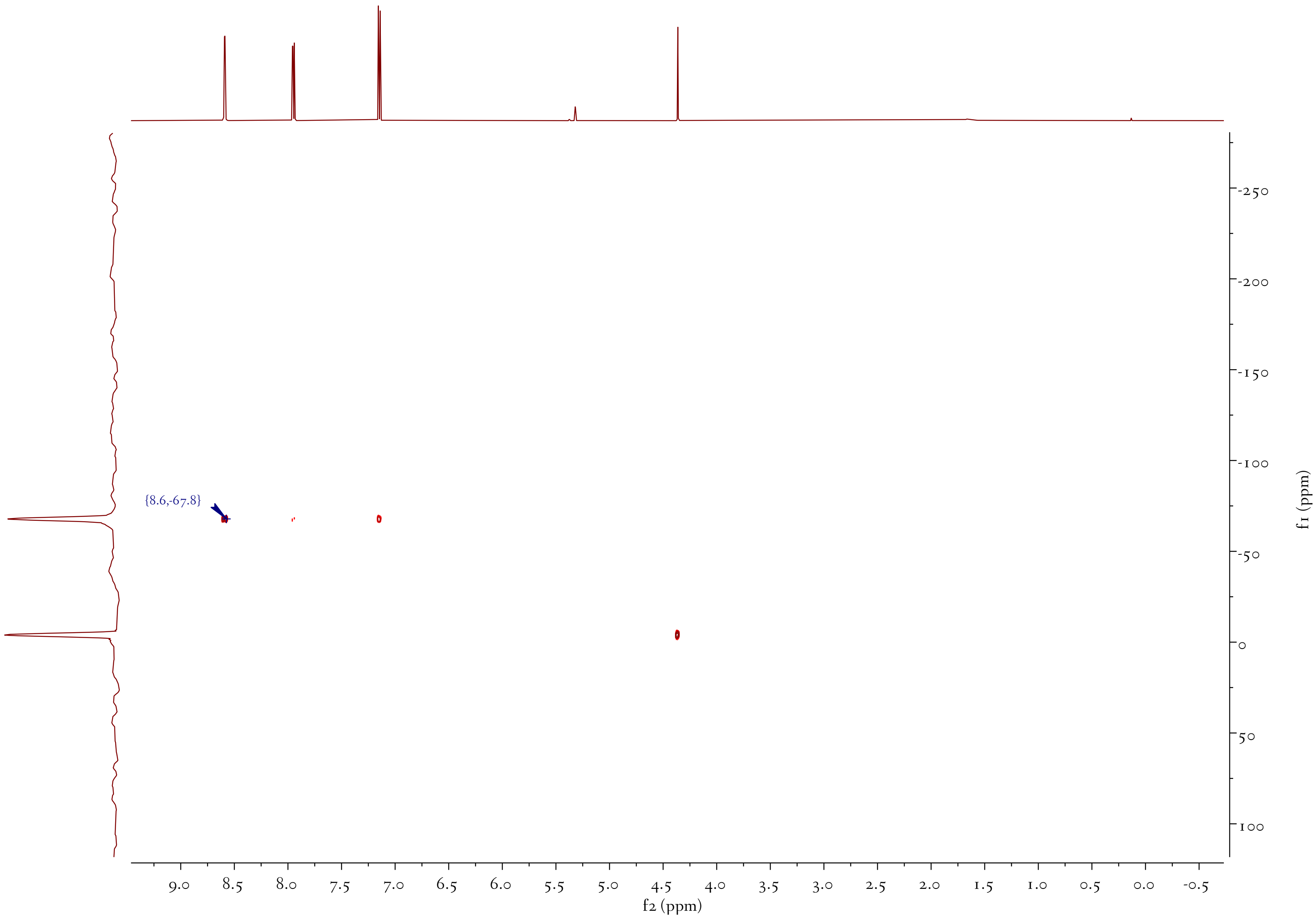
{8.6,-67.8}
-250
-200
-150
-100
-50
0
50
100
f1 (ppm)
9.0
8.5
8.0
7.5
7.0
6.5
6.0
5.5
5.0
4.5
4.0
3.5
3.0
2.5
2.0
1.5
1.0
0.5
0.0
-0.5
f2 (ppm)

Figure S45. The ^{1}H–^{15}N HMBC spectrum of **6** in CD_2Cl_2.

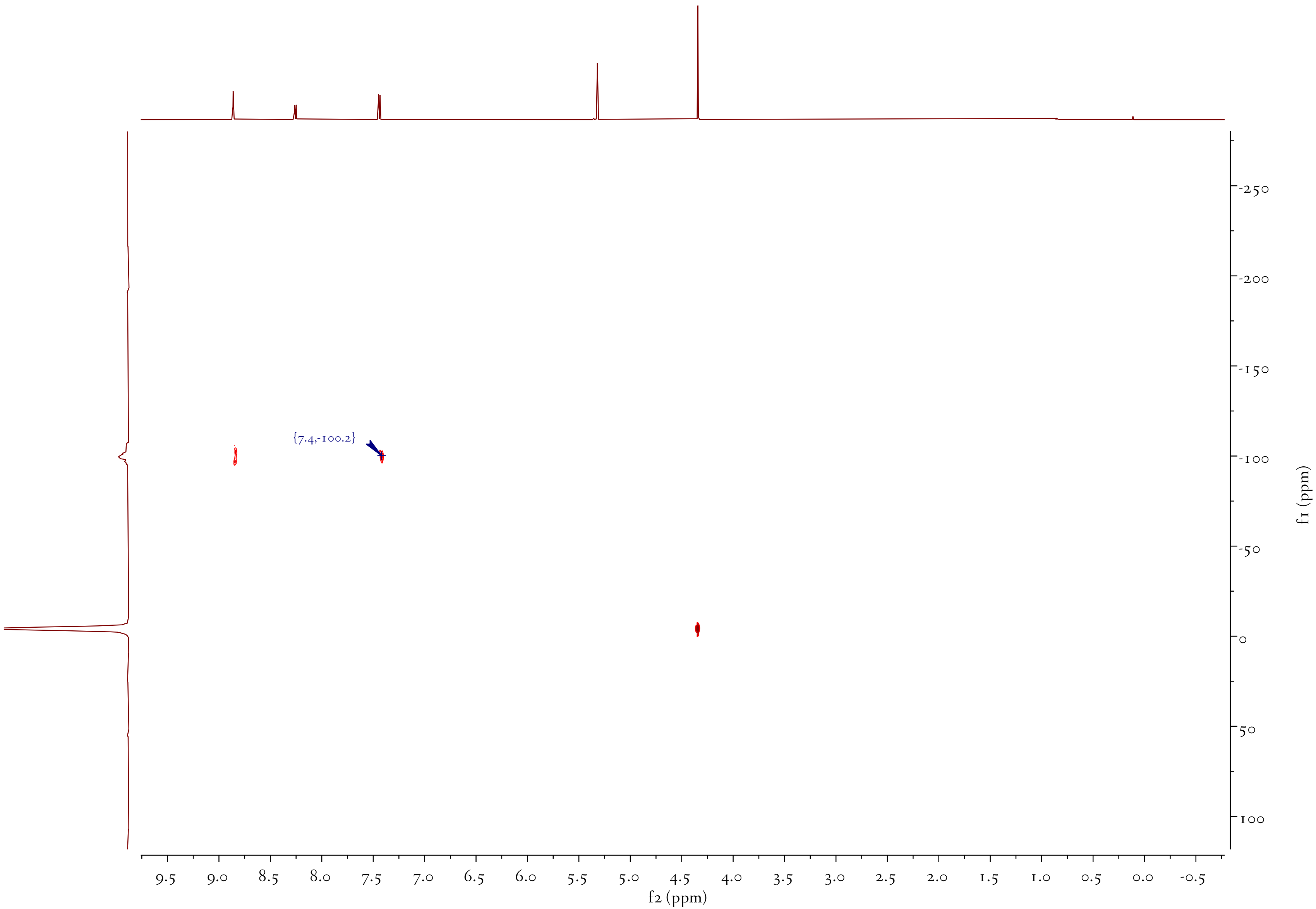
{7.4,-100.2}
-250
-200
-150
-100
-50
0
50
100
f1 (ppm)
9.5 9.0 8.5 8.0 7.5 7.0 6.5 6.0 5.5 5.0 4.5 4.0 3.5 3.0 2.5 2.0 1.5 1.0 0.5 0.0 -0.5
f2 (ppm)

Figure S46. The ^{1}H–^{15}N HMBC spectrum of [**6**–Ag–**6**]$^{+}$ in CD_2Cl_2

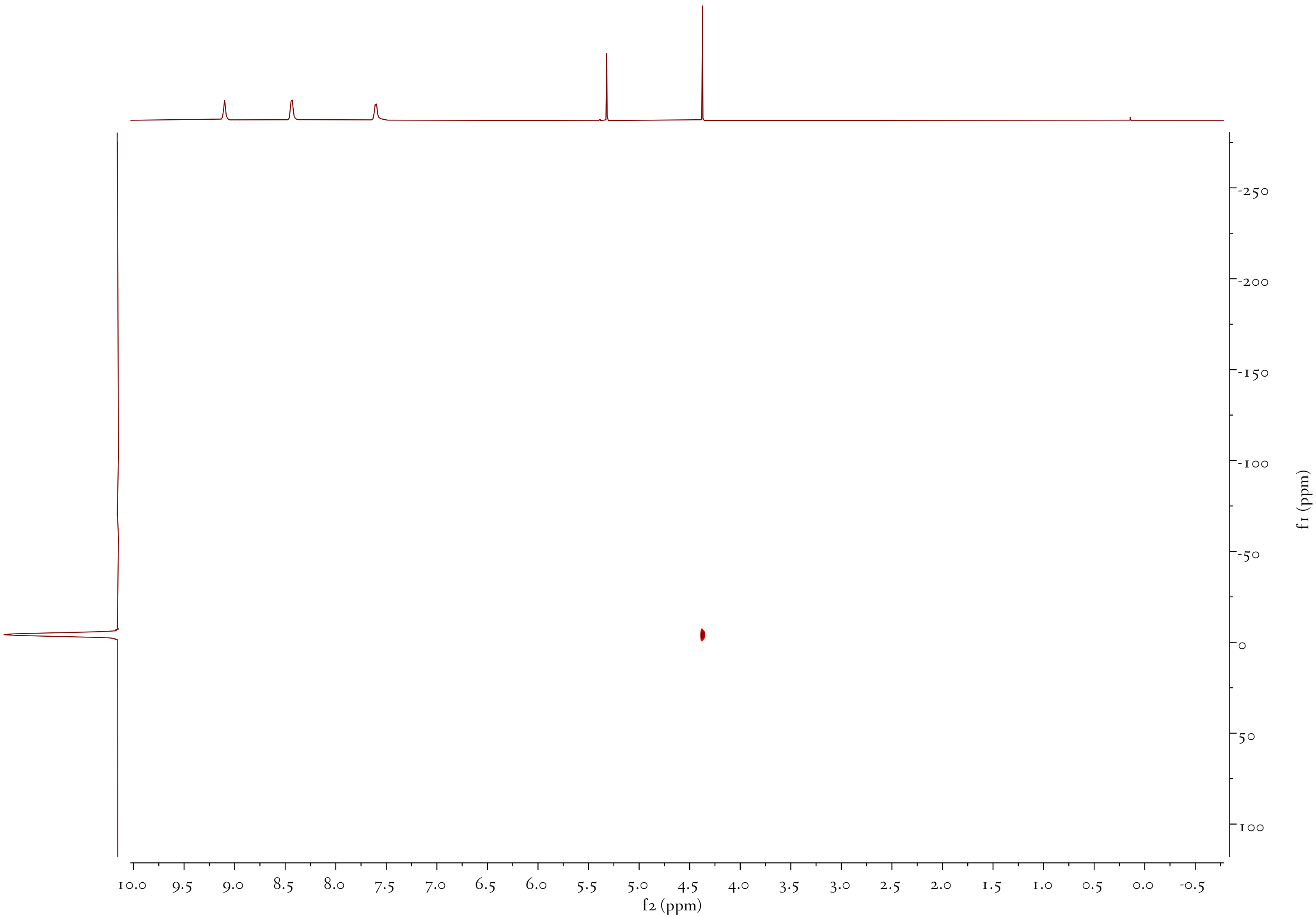

-250
-200
-150
-100
-50
0
50
100
f1 (ppm)
10.0
9.5
9.0
8.5
8.0
7.5
7.0
6.5
6.0
5.5
5.0
4.5
4.0
3.5
3.0
2.5
2.0
1.5
1.0
0.5
0.0
-0.5
f2 (ppm)

Figure S47. The ^{1}H–^{15}N HMBC spectrum of [**6**–I–**6**]$^+$ in CD_2Cl_2. No signals from the complex were detected at 298 K.

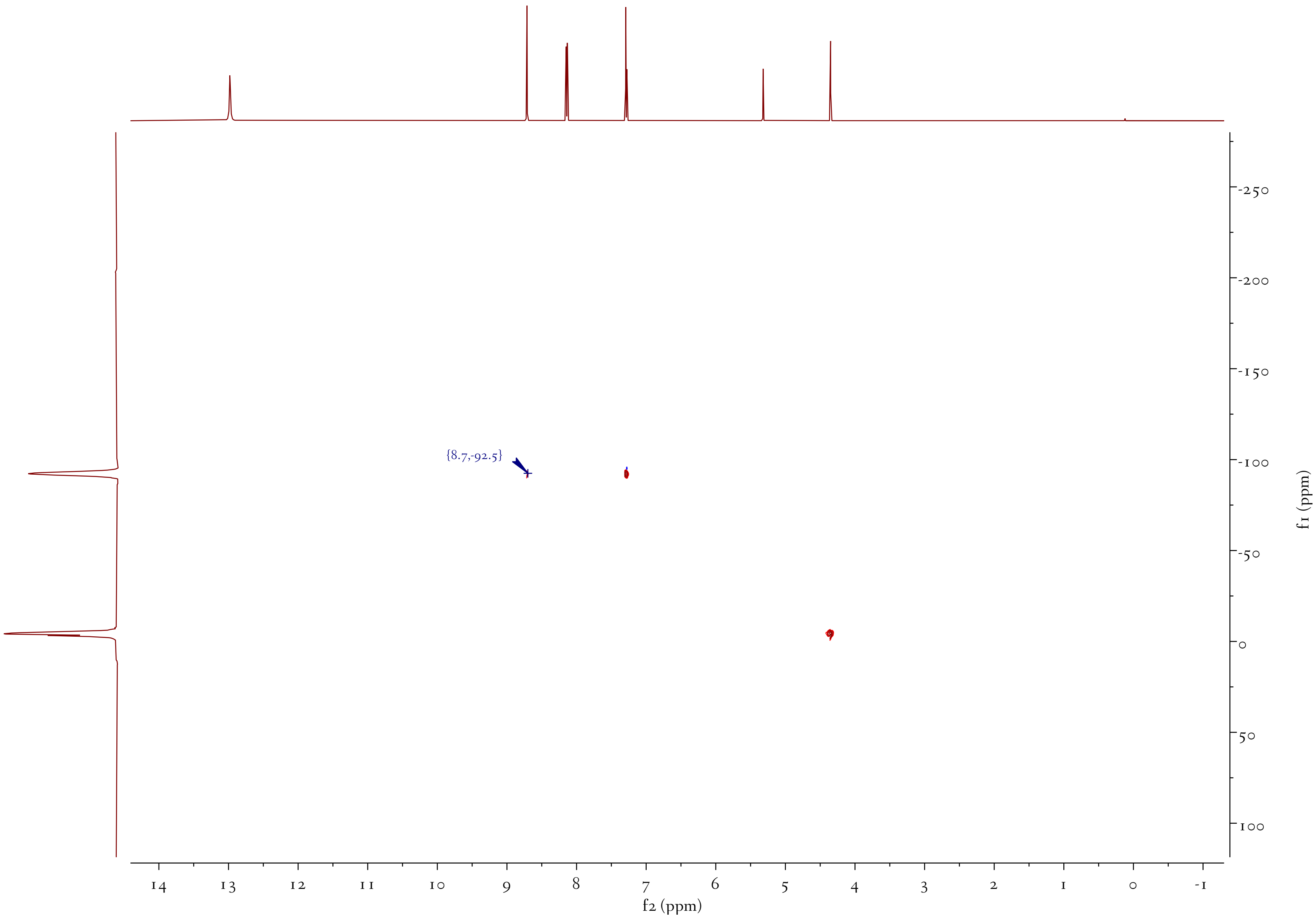

{8.7,-92.5}
-250
-200
-150
-100
-50
0
50
100
f1 (ppm)
14
13
12
11
10
9
8
7
6
5
4
3
2
1
0
-1
f2 (ppm)

Figure S48. The ^{1}H–^{15}N HMBC spectrum of [**6**H]$^{+}$ in CD_2Cl_2.

2.3 19F spectra

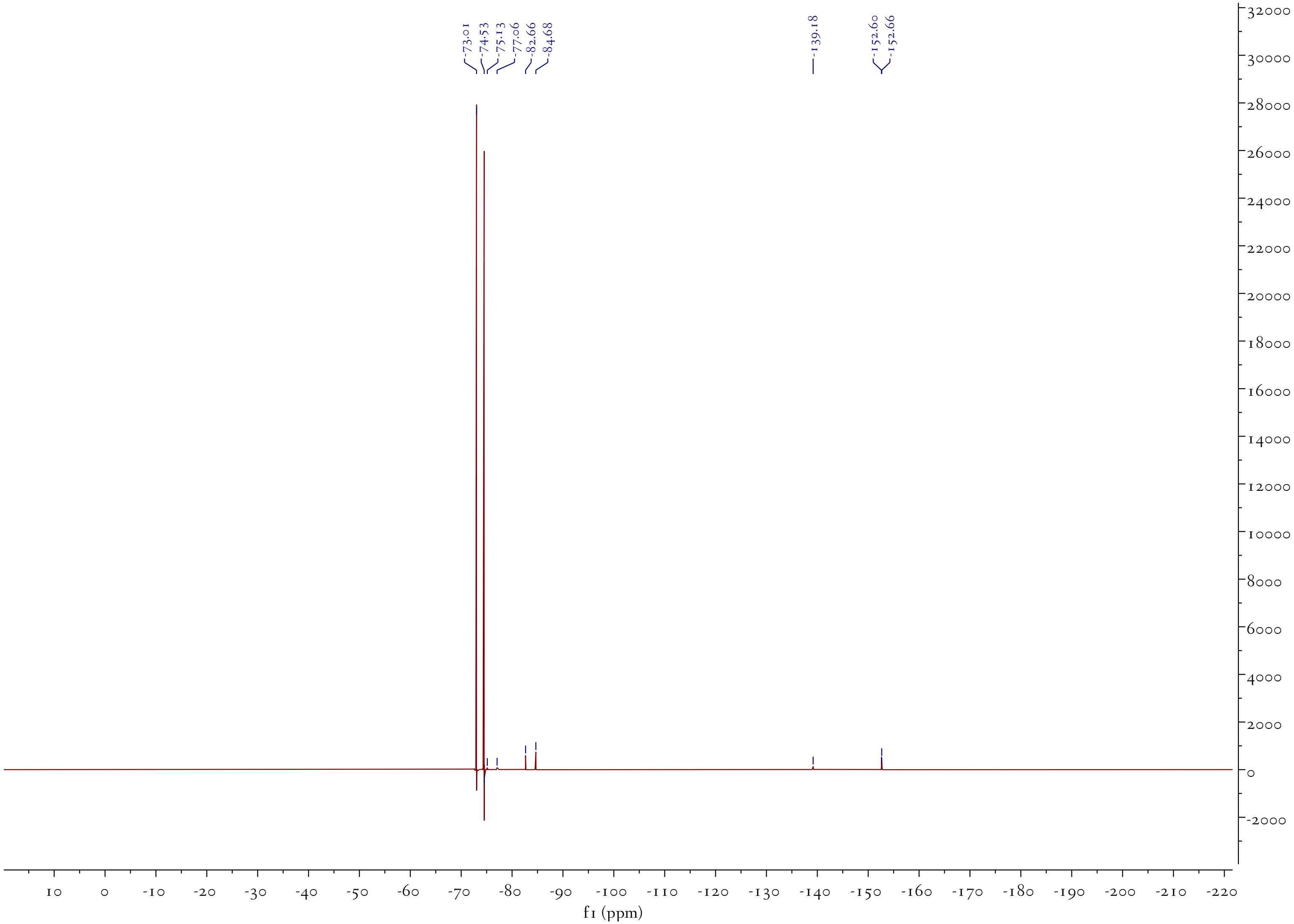

Figure S49. The ^{19}F spectrum of non-commercial $AgPF_6$ in CD_3CN.

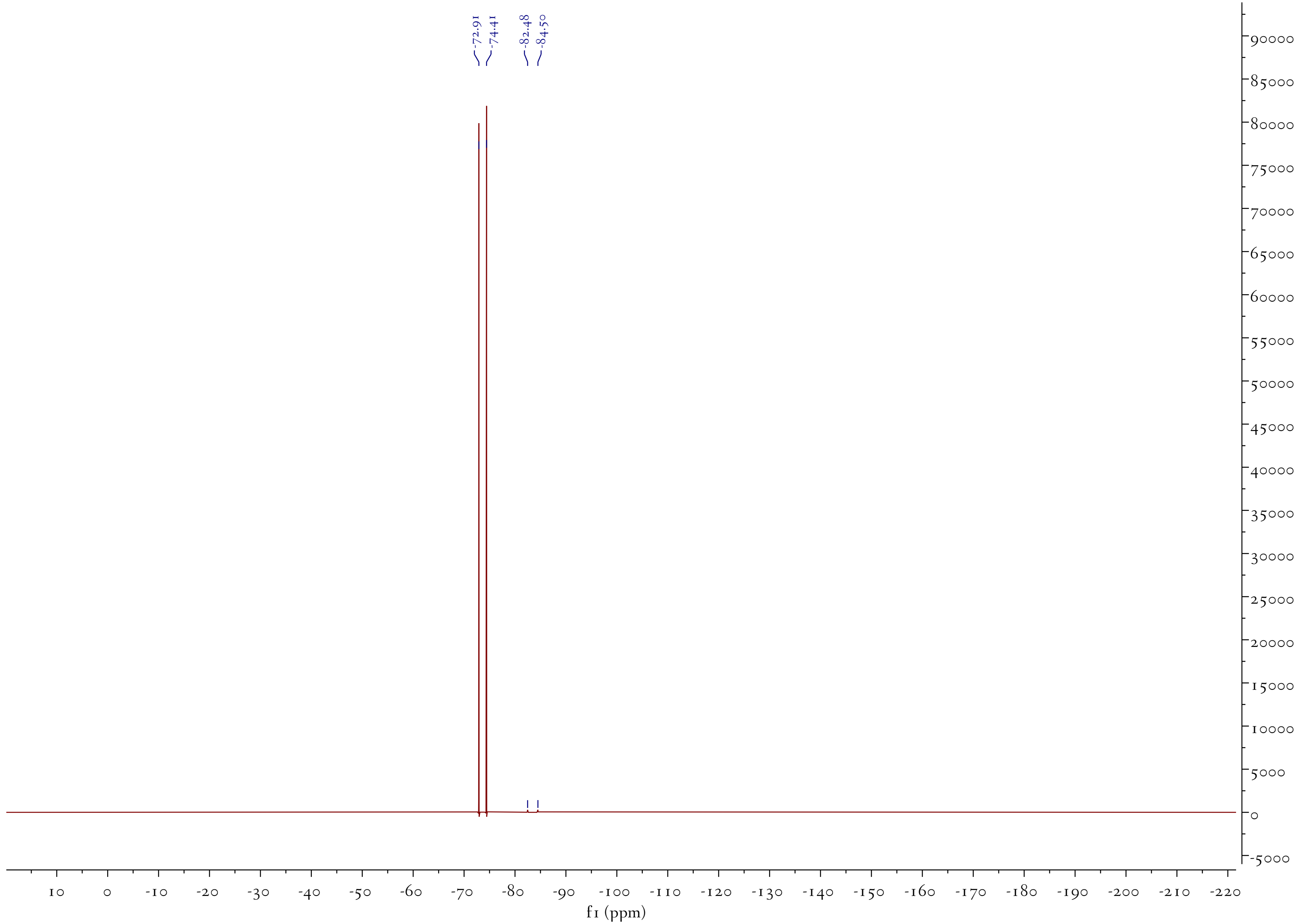
-72.91
-74.41
-82.48
-84.50
90000
85000
80000
75000
70000
65000
60000
55000
50000
45000
40000
35000
30000
25000
20000
15000
10000
5000
0
-5000
10 0 -10 -20 -30 -40 -50 -60 -70 -80 -90 -100 -110 -120 -130 -140 -150 -160 -170 -180 -190 -200 -210 -220
f1 (ppm)

Figure S50. The ^{19}F spectrum of commercial $AgPF_6$ in CD_3CN.

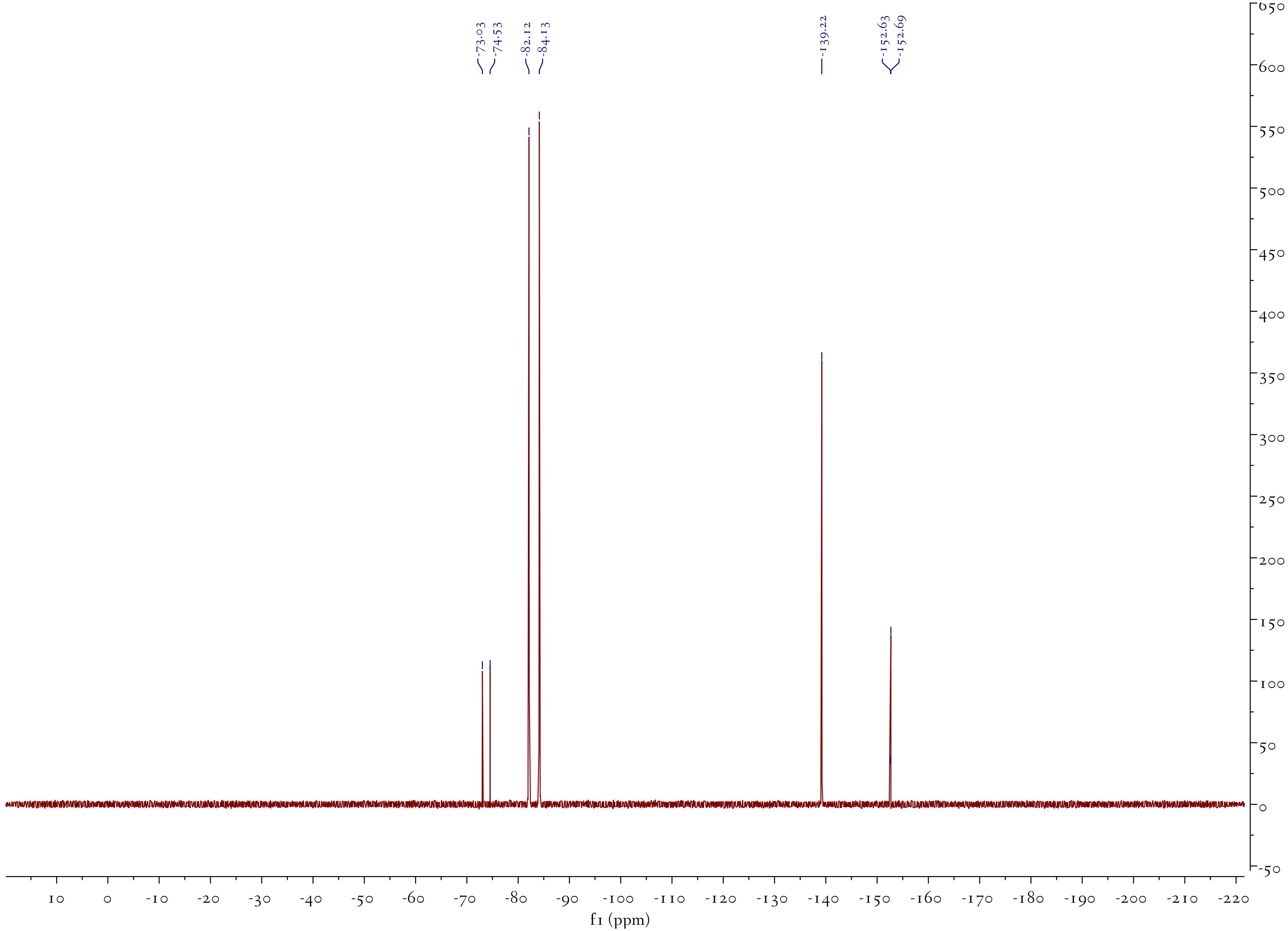
-73.03
-74.53
-82.12
-84.13
-139.22
-152.63
-152.69
650
600
550
500
450
400
350
300
250
200
150
100
50
0
-50
10 0 -10 -20 -30 -40 -50 -60 -70 -80 -90 -100 -110 -120 -130 -140 -150 -160 -170 -180 -190 -200 -210 -220
f1 (ppm)

Figure S51. The ^{19}F spectrum of [**6**–Ag–**6**]PF_2O_2 in CD_3CN.

2.4 ^{31}P spectra

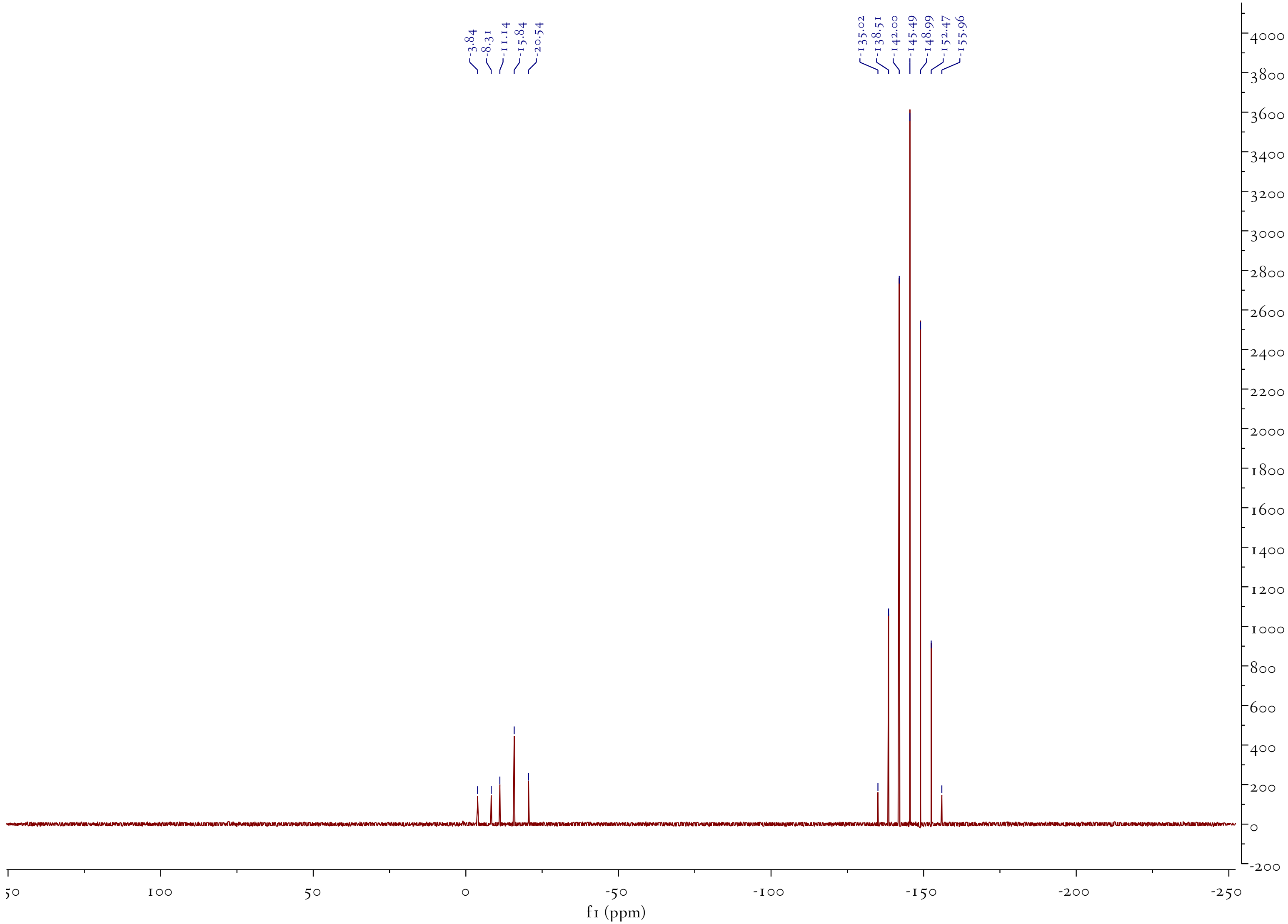

Figure S52. The ^{31}P spectrum of non-commercial $AgPF_6$ in CD_3CN.

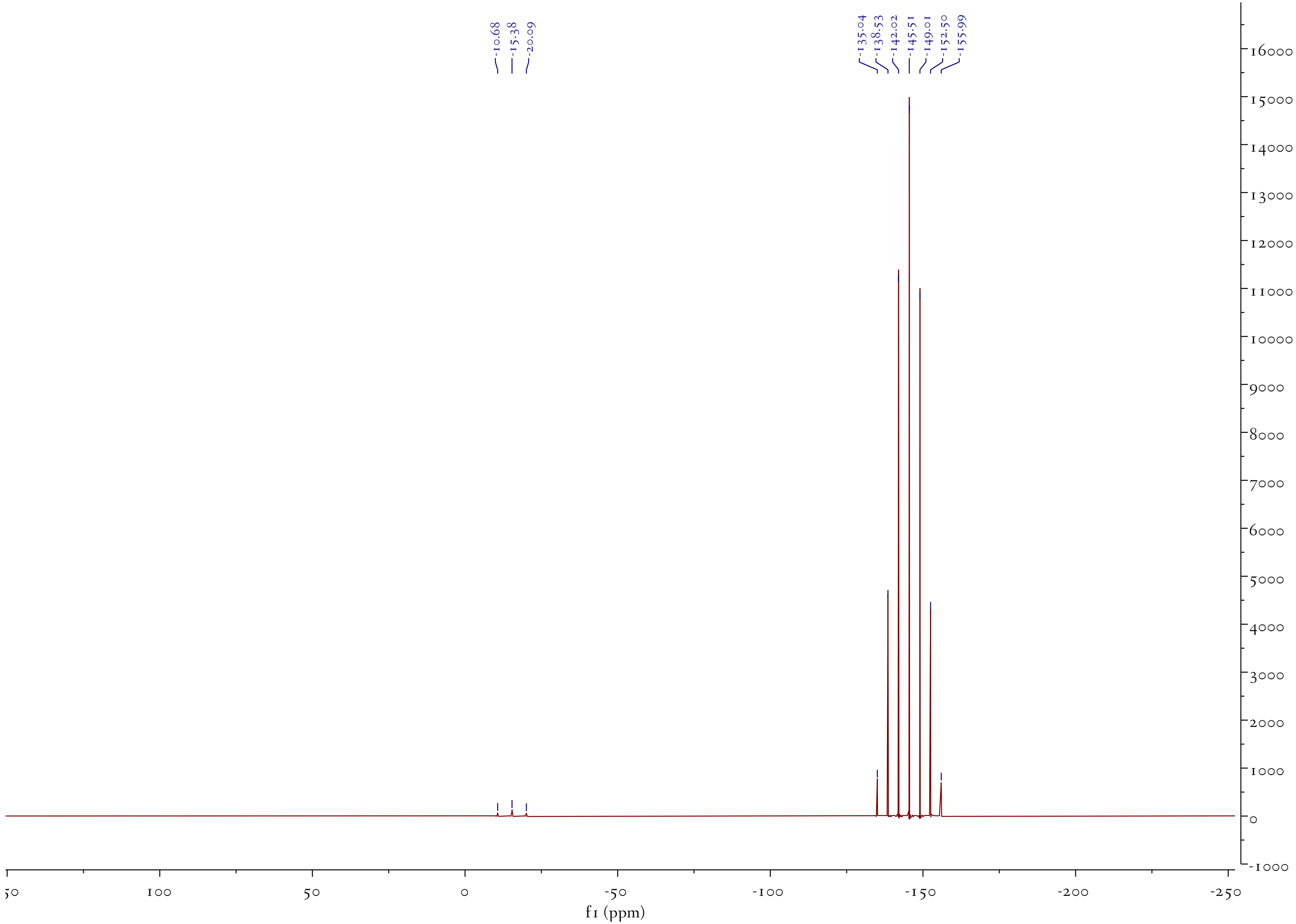

-10.68
-15.38
-20.09
-135.04
-138.53
-142.02
-145.51
-149.01
-152.50
-155.99
16000
15000
14000
13000
12000
11000
10000
9000
8000
7000
6000
5000
4000
3000
2000
1000
0
-1000
150
100
50
0
-50
-100
-150
-200
-250
f1 (ppm)

Figure S53. The ^{31}P spectrum of commercial $AgPF_6$ in CD_3CN.

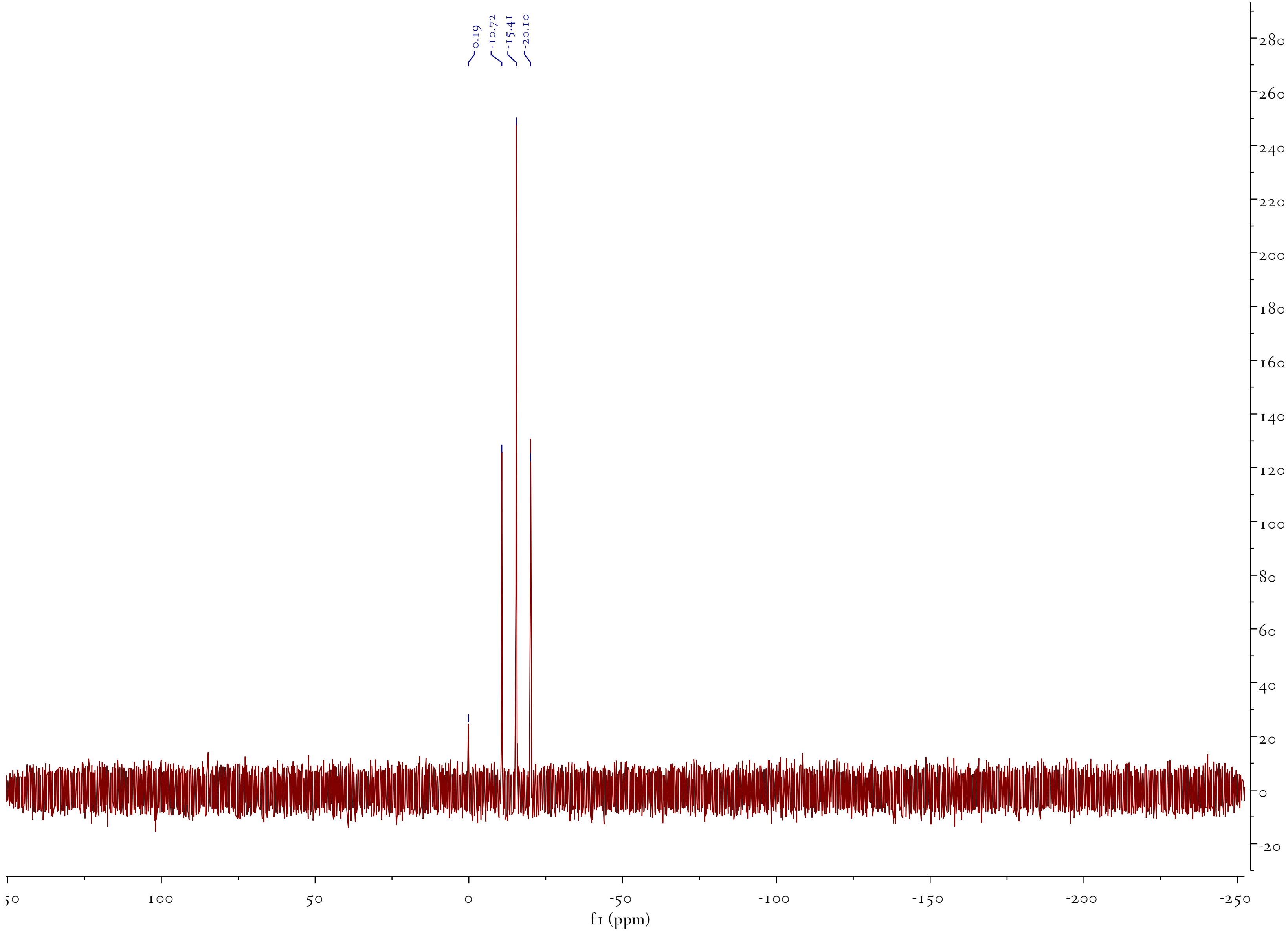
0.19
-10.72
-15.41
-20.10
280
260
240
220
200
180
160
140
120
100
80
60
40
20
0
-20
150
100
50
0
-50
-100
-150
-200
-250
f1 (ppm)

Figure S54. The ^{31}P spectrum of [**6**–Ag–**6**]PF_2O_2 in CD_3CN.

3. X-ray crystallography

3.1 Silver(I) complexes

Complexes, counterions and solvent molecules partially within the asymmetric unit have been grown. Thermal ellipsoids are drawn at the 50% probability level in all structures presented.

[Ag(3-MePy)$_2$]PF$_6$ ([1–Ag–1]PF$_6$):

Crystals suitable for X-ray diffraction were obtained through vapor diffusion with pentane. Crystal Data for CCDC-2467910, $C_{12}H_{14}AgF_6N_2P$ (M=439.09 g/mol): monoclinic, space group P2$_1$/n (no. 14), a = 8.2694(2) Å, b = 17.7293(3) Å, c = 10.9625(2) Å, β = 103.6310(10)°, V = 1561.95(5) Å^3, Z = 4, T = 200(1) K, μ(Mo Kα) = 1.451 mm^{-1}, D_{calc} = 1.867 g/cm^3, 30028 reflections measured (5.566° ≤ 2Θ ≤ 55.038°), 3592 unique (R_{int} = 0.0457, R_{sigma} = 0.0287) which were used in all calculations. The final R_1 was 0.0446 (I > 2σ(I)) and wR_2 was 0.0762 (all data).

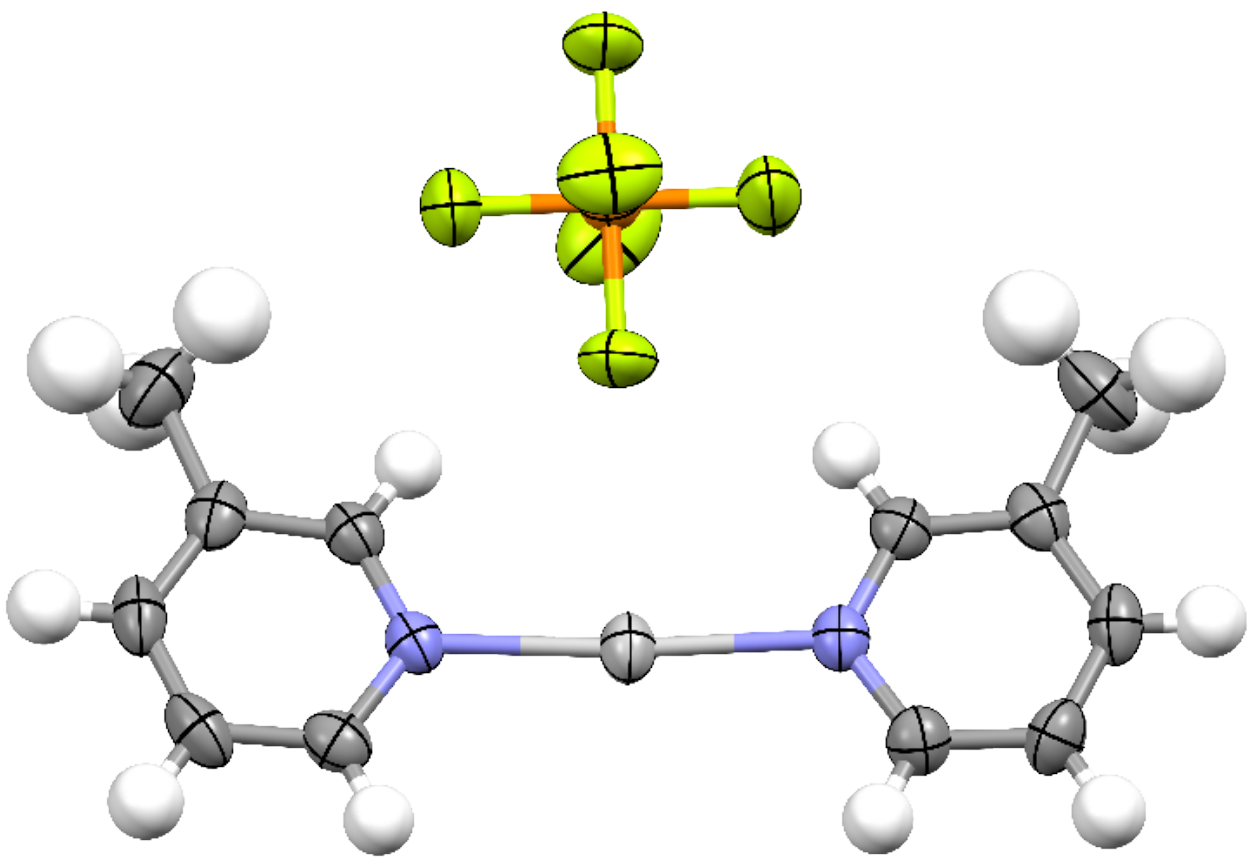

Figure S55. The crystal structure of [1–Ag–1]PF$_6$. Colors: light gray = silver, dark gray = carbon, white = hydrogen, blue = nitrogen, orange = phosphorus, neon green = fluorine.

[Ag(3-FPy)$_2$]PF$_6$ ([2–Ag–2]PF$_6$):

Crystals suitable for X-ray diffraction were obtained through vapor diffusion with pentane. Crystal Data for CCDC-2467907, $C_{10.5}H_9AgClF_8N_2P$ (M=489.49 g/mol): monoclinic, space group C2/m (no. 12), a = 13.9337(5) Å, b = 16.3478(5) Å, c = 7.9912(2) Å, β = 121.808(2)°, V = 1546.91(9) Å^3, Z = 4, T = 170(1) K, μ(Mo Kα) = 1.662 mm^{-1}, D_{calc} = 2.102 g/cm^3, 8513 reflections measured (5.682° ≤ 2Θ ≤ 55.044°), 1829 unique (R_{int} = 0.0264, R_{sigma} = 0.0251) which were used in all calculations. The final R_1 was 0.0359 (I > 2σ(I)) and wR_2 was 0.0757 (all data).

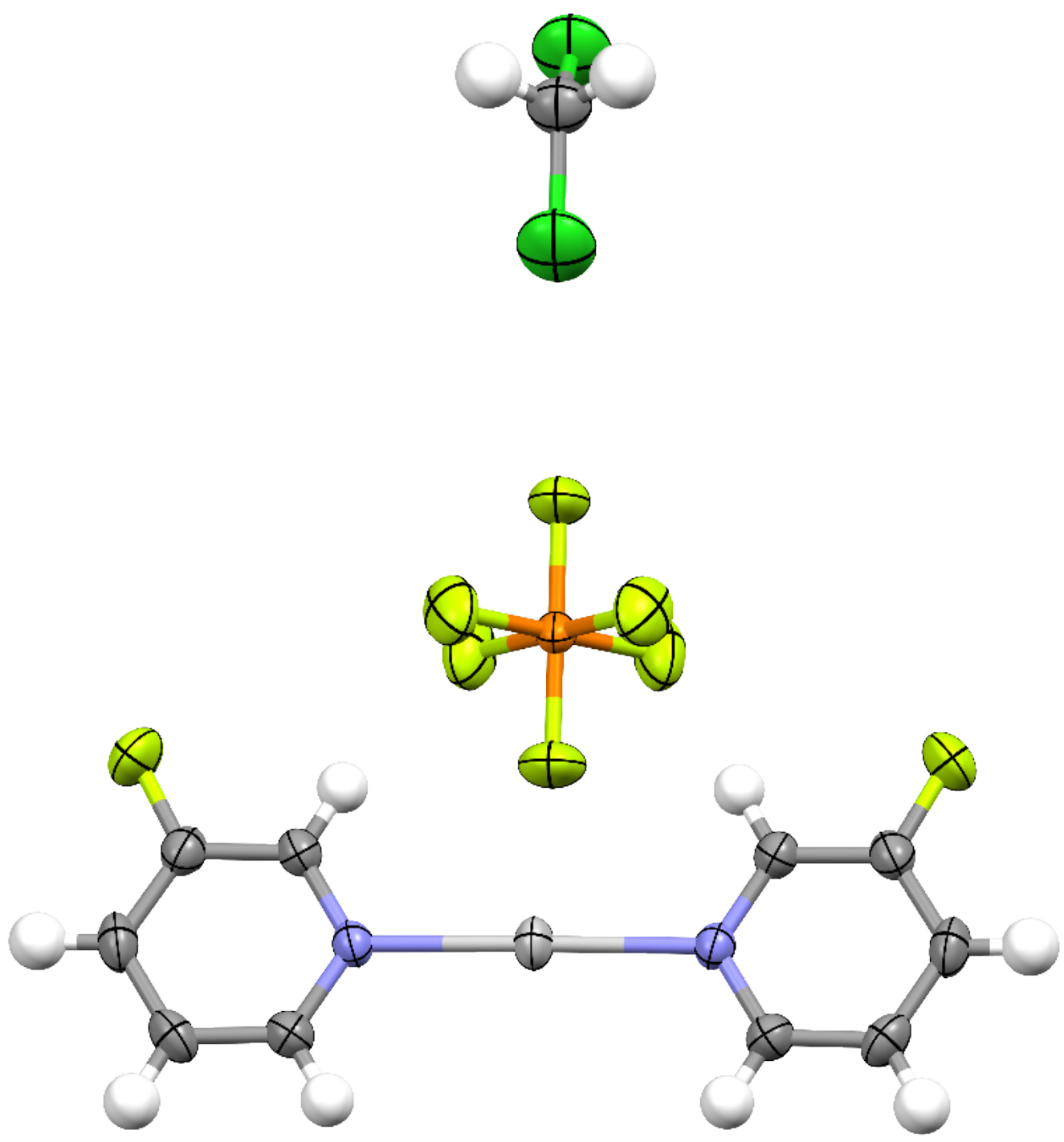

Figure S56. The crystal structure of [**2**–Ag–**2**]PF_6. Colors: light gray = silver, dark gray = carbon, white = hydrogen, blue = nitrogen, orange = phosphorus, neon green = fluorine, green = chlorine.

$[Ag(3\text{-}ClPy)_2]PF_6$ ($[3–Ag–3]PF_6$):

Crystals suitable for X-ray diffraction were obtained through vapor diffusion with pentane. Crystal Data for CCDC-2467904, $C_{10.333333}H_{8.666667}AgCl_{2.666667}F_6N_2P$ (M=508.23 g/mol): monoclinic, space group C2/c (no. 15), a= 10.7468(2) Å, b= 28.3299(4) Å, c= 16.4019(2) Å, β= 105.3710(10)°, V= 4815.03(13) Å^3, Z= 12, T= 170(1) K, μ(Mo Kα) = 1.857 mm^{-1}, D_{calc}= 2.103 g/cm^3, 22016 reflections measured (4.186° ≤ 2Θ ≤ 54.986°), 5503 unique (R_{int} = 0.0324, R_{sigma} = 0.0314) which were used in all calculations. The final R_1 was 0.0409 (I > 2σ(I)) and wR_2 was 0.0734 (all data).

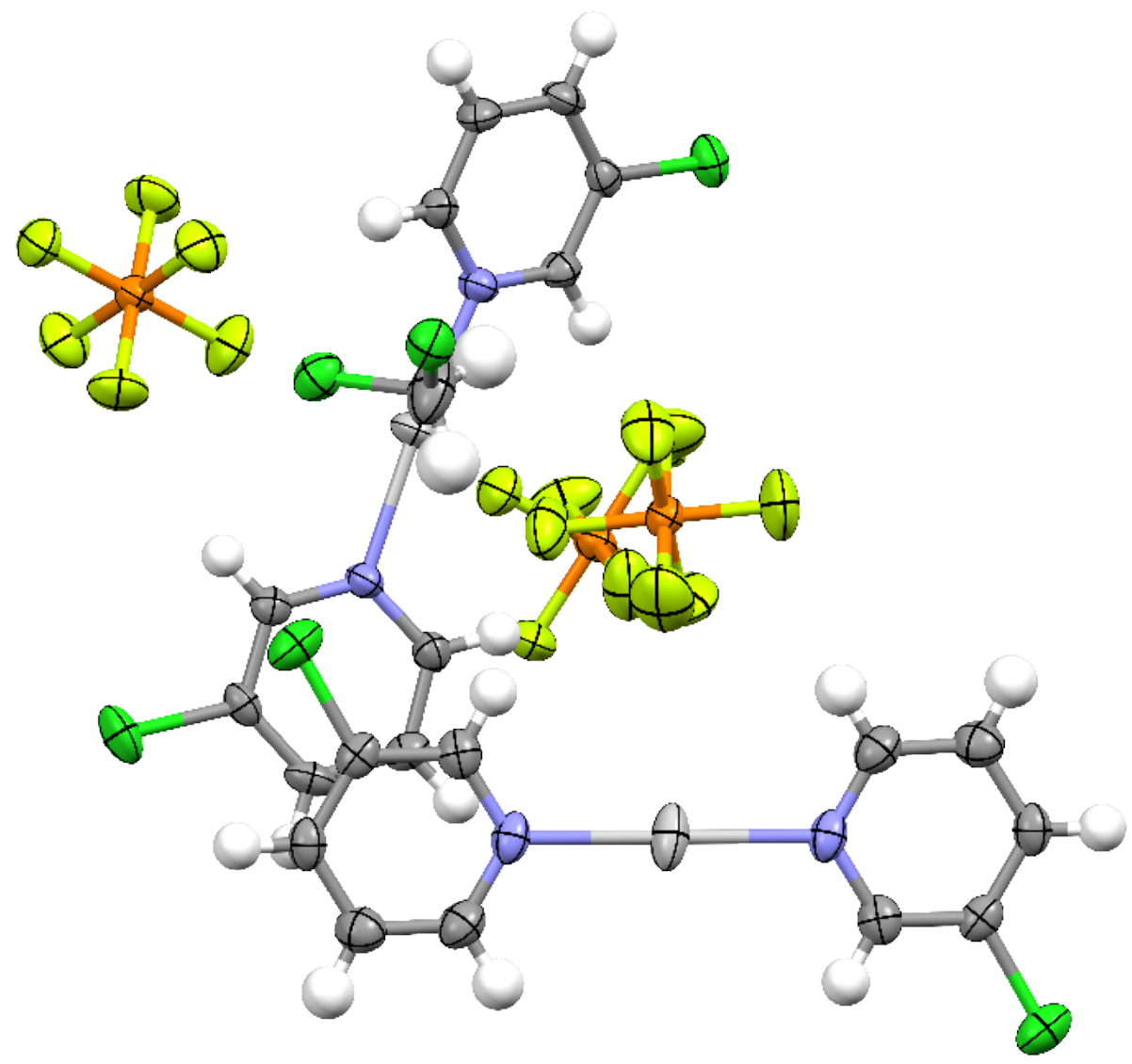

Figure S57. The crystal structure of $[3–Ag–3]PF_6$. Colors: light gray = silver, dark gray = carbon, white = hydrogen, blue = nitrogen, orange = phosphorus, neon green = fluorine, green = chlorine.

$[Ag(3\text{-}BrPy)_2]PF_6$ ([4–Ag–4]PF_6):

Crystals suitable for X-ray diffraction were obtained through vapor diffusion with diethyl ether. Crystal Data for CCDC-2467906, $C_{10}H_8AgBr_2F_6N_2P$ (M=568.84 g/mol): tetragonal, space group $P4_32_12$ (no. 96), a = 21.3063(4) Å, c = 13.9878(4) Å, V = 6349.9(3) Å^3, Z = 16, T = 170(1) K, μ(Mo Kα) = 6.460 mm^{-1}, D_{calc} = 2.380 g/cm^3, 32818 reflections measured (5.172° $\leq 2\theta \leq$ 52.05°), 6203 unique (R_{int} = 0.0902, R_{sigma} = 0.0735) which were used in all calculations. The final R_1 was 0.0765 (I > 2σ(I)) and wR_2 was 0.1977 (all data).

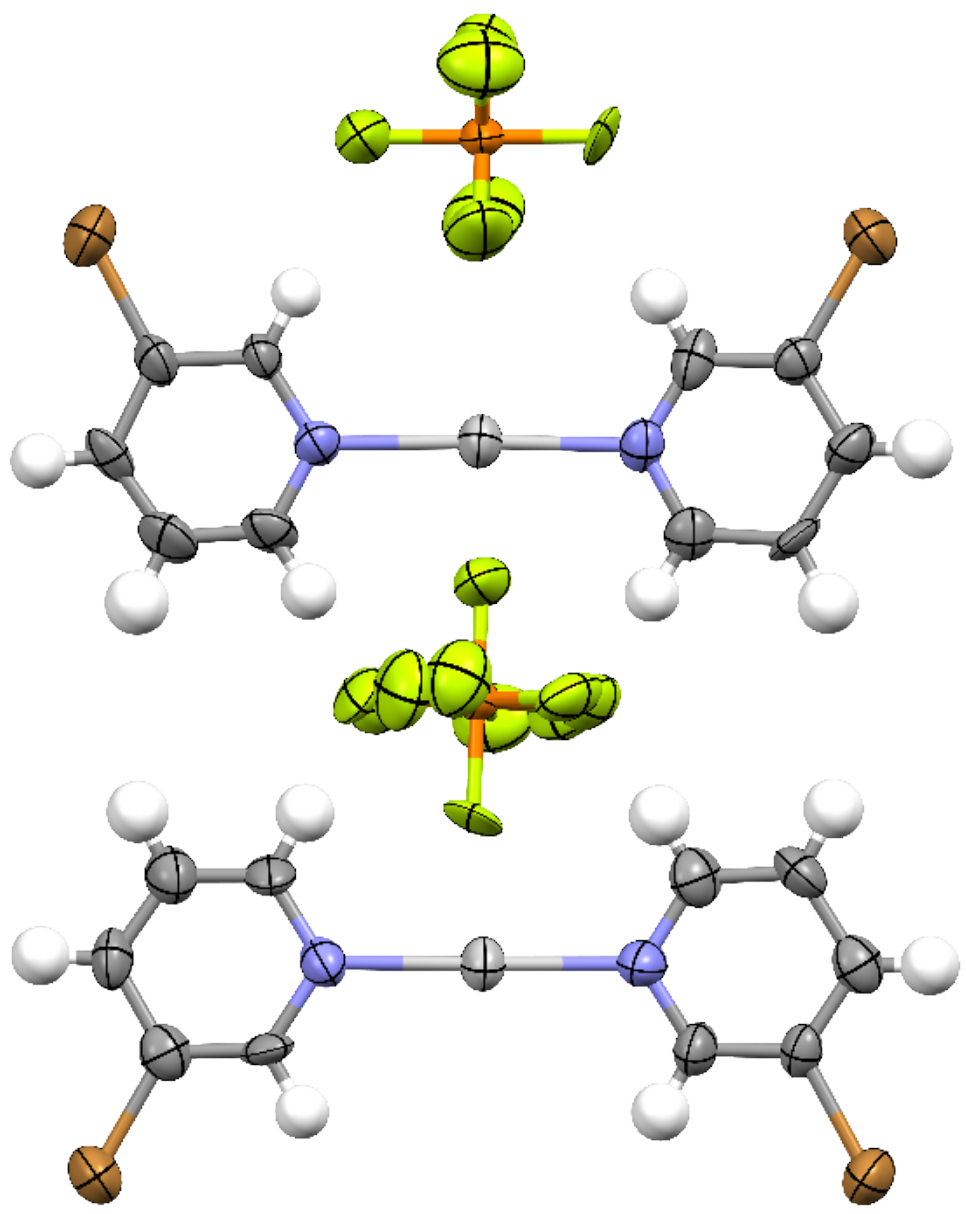

Figure S58. The crystal structure of [**4**–Ag–**4**]PF_6. Colors: light gray = silver, dark gray = carbon, white = hydrogen, blue = nitrogen, orange = phosphorus, neon green = fluorine, brown = bromine. One PF_6^- counterion is disordered over two positions.

$[Ag(3\text{-}IPy)_2]PF_6$ ([**5**–Ag–**5**]PF_6):

Attempts to grow high-quality crystals were unsuccessful. Poor-quality crystals were obtained through vapor diffusion with diethyl ether from a CH_2Cl_2 solution to which MeCN was added dropwise until the complex dissolved. Crystal Data for $C_{10}AgF_6I_2N_2P$ (M=654.76 g/mol): tetragonal, space group $P4_12_12$ (no. 92), a = 21.5406(2) Å, c = 13.95370(10) Å, V = 6474.48(13) Å^3, Z = 16, T = 170(1) K, μ(Mo Kα) = 5.213 mm^{-1}, D_{calc} = 2.687 g/cm^3, 73347 reflections measured (3.478° ≤ 2Θ ≤ 57.194°), 8235 unique (R_{int} = 0.0656, R_{sigma} = 0.0401) which were used in all calculations. The final R_1 was 0.1416 (I > 2σ(I)) and wR_2 was 0.4030 (all data).

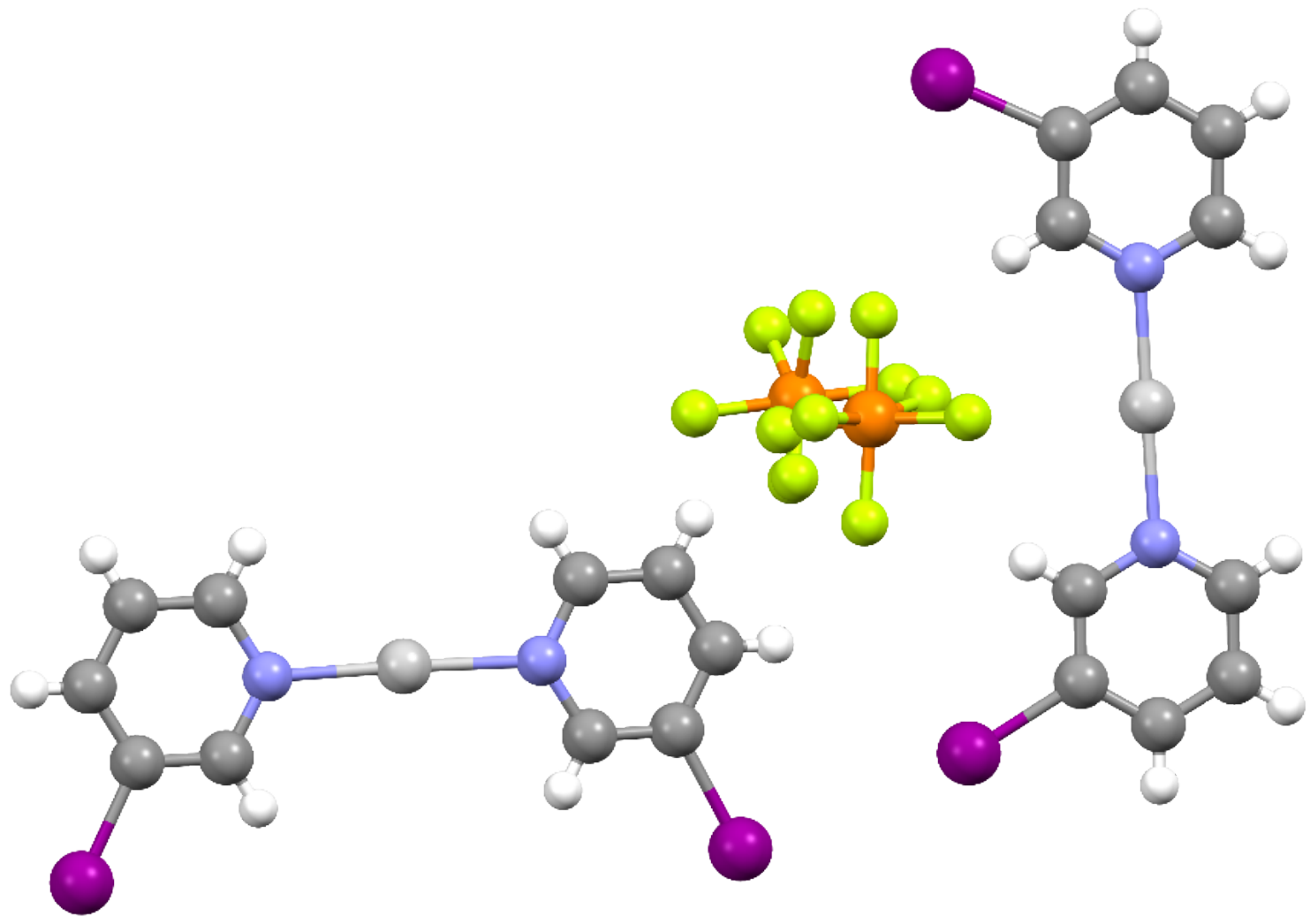

Figure S59. The preliminary crystal structure of [**5**–Ag–**5**]PF_6. Colors: light gray = silver, dark gray = carbon, white = hydrogen, blue = nitrogen, orange = phosphorus, neon green = fluorine, purple = iodine. Atoms drawn as cubes are NPD.

[Ag(2-Cl-5-IPy)$_2$]PF$_6$ ([6–Ag–6]PF$_6$), [Ag(2-Cl-5-IPy)$_2$]BF$_4$ ([6–Ag–6]BF$_4$), and [Ag(2-Cl-5-IPy)(PO₂F₂)]$_n$ (6-Ag-PO$_2$F$_2$):

Crystals suitable for X-ray diffraction were obtained through vapor diffusion with pentane in a plastic microcentrifuge tube. Crystal Data for CCDC-2467912, $C_{10}H_6N_2Cl_2I_2AgPF_6$ (M=731.71 g/mol): triclinic, space group P-1 (no. 2), a = 7.7679(2) Å, b = 7.9821(2) Å, c = 8.5646(2) Å, α = 78.703(2)°, β = 65.161(2)°, γ = 66.999(2)°, V = 443.28(2) Å^3, Z = 1, T = 170(1) K, μ(Mo Kα) = 5.065 mm^{-1}, D_{calc} = 2.741 g/cm^3, 5672 reflections measured (5.244° ≤ 2Θ ≤ 54.986°), 2021 unique (R_{int} = 0.0285, R_{sigma} = 0.0350) which were used in all calculations. The final R_1 was 0.0301 (I > 2σ(I)) and wR_2 was 0.0578 (all data).

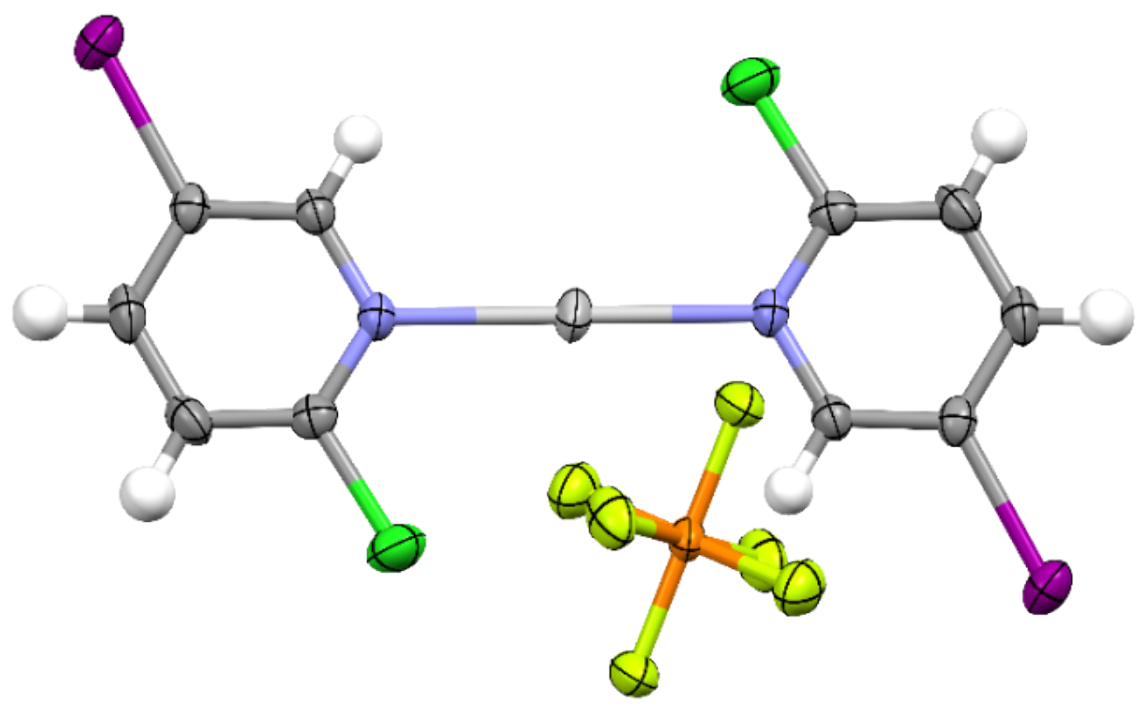

Figure S60. The crystal structure of [6–Ag–6]PF$_6$. Colors: light gray = silver, dark gray = carbon, white = hydrogen, blue = nitrogen, orange = phosphorus, neon green = fluorine, green = chlorine, purple = iodine.

Crystals suitable for X-ray diffraction were obtained through vapor diffusion with pentane in a glass vial. Crystal Data for CCDC-2467908, $C_{10}H_6AgBCl_2F_4I_2N_2$ (M=673.55 g/mol): triclinic, space group P-1 (no. 2), a = 7.27770(10) Å, b = 8.2872(2) Å, c = 14.3961(3) Å, α = 73.9460(10)°, β = 84.8700(10)°, γ = 82.5010(10)°, V = 825.96(3) Å^3, Z = 2, T = 170(1) K, μ(Mo Kα) = 5.314 mm^{-1}, D_{calc} = 2.708 g/cm^3, 12468 reflections measured (5.146° ≤ 2Θ ≤ 55.064°), 3789 unique (R_{int} = 0.0295, R_{sigma} = 0.0349) which were used in all calculations. The final R_1 was 0.0329 (I > 2σ(I)) and wR_2 was 0.0591 (all data).

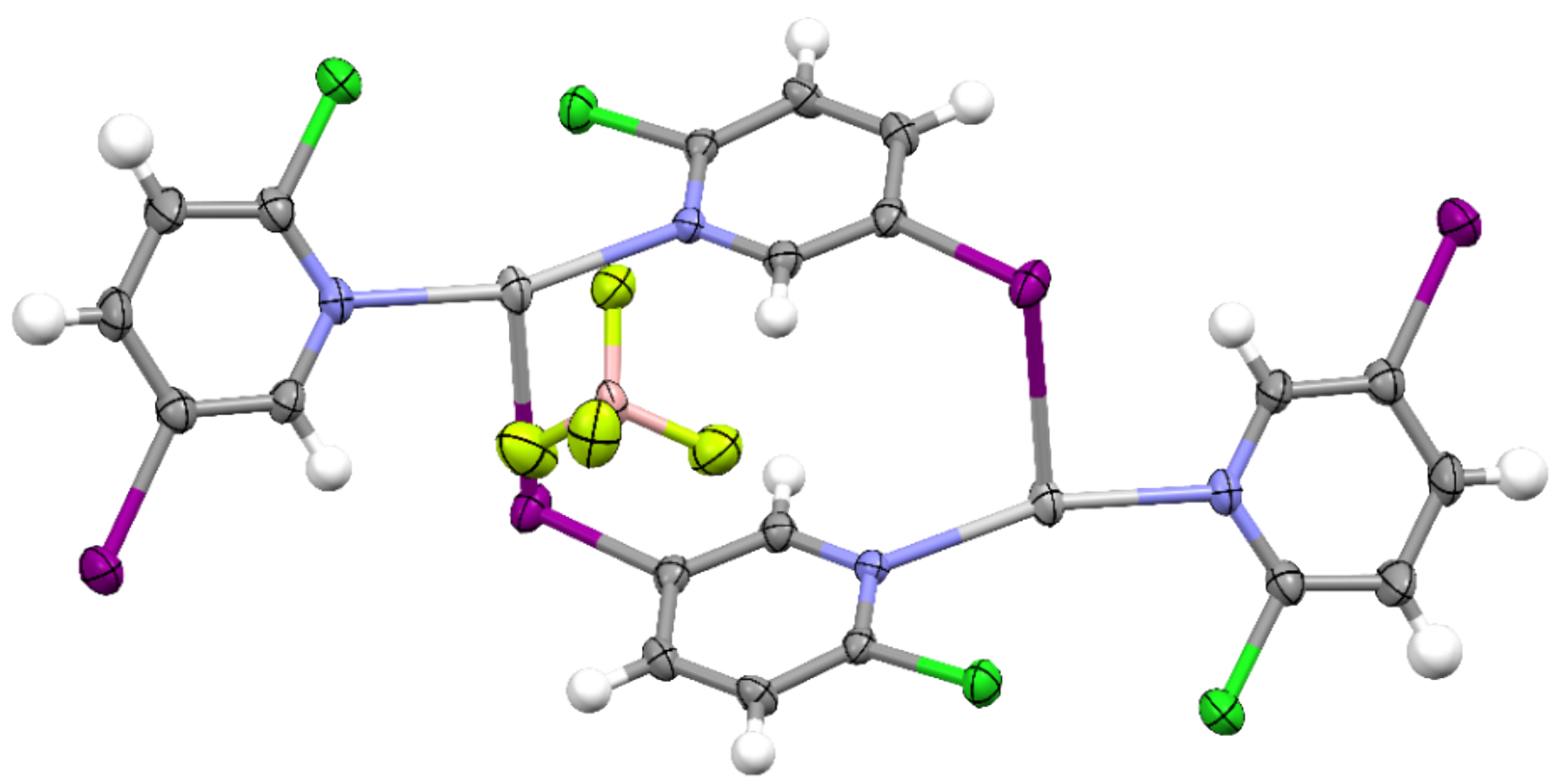

Figure S61. The crystal structure of [6–Ag–6]BF$_4$. Colors: light gray = silver, dark gray = carbon, white = hydrogen, blue = nitrogen, light pink = boron, neon green = fluorine, green = chlorine, purple = iodine.

Crystals suitable for X-ray diffraction were obtained through vapor diffusion with pentane in a glass vial. Crystal Data for CCDC-2593238, $C_5H_3AgClF_2INO_2P$ (M=448.27 g/mol): triclinic, space group P-1 (no. 2), a = 5.08620(10) Å, b = 10.0050(2) Å, c = 10.7362(3) Å, α = 102.1000(10)°, β = 99.0110(10)°, γ = 98.5990(10)°, V = 518.12(2) Å^3, Z = 2, T = 200(1) K, μ(MoKα) = 5.337 mm^{-1}, D_{calc} = 2.873 g/cm^3, 8563 reflections measured (5.062° ≤ 2Θ ≤ 57.334°), 2203 unique (R_{int} = 0.0403, R_{sigma} = 0.0383) which were used in all calculations. The final R_1 was 0.0292 (I > 2σ(I)) and wR_2 was 0.0572 (all data).

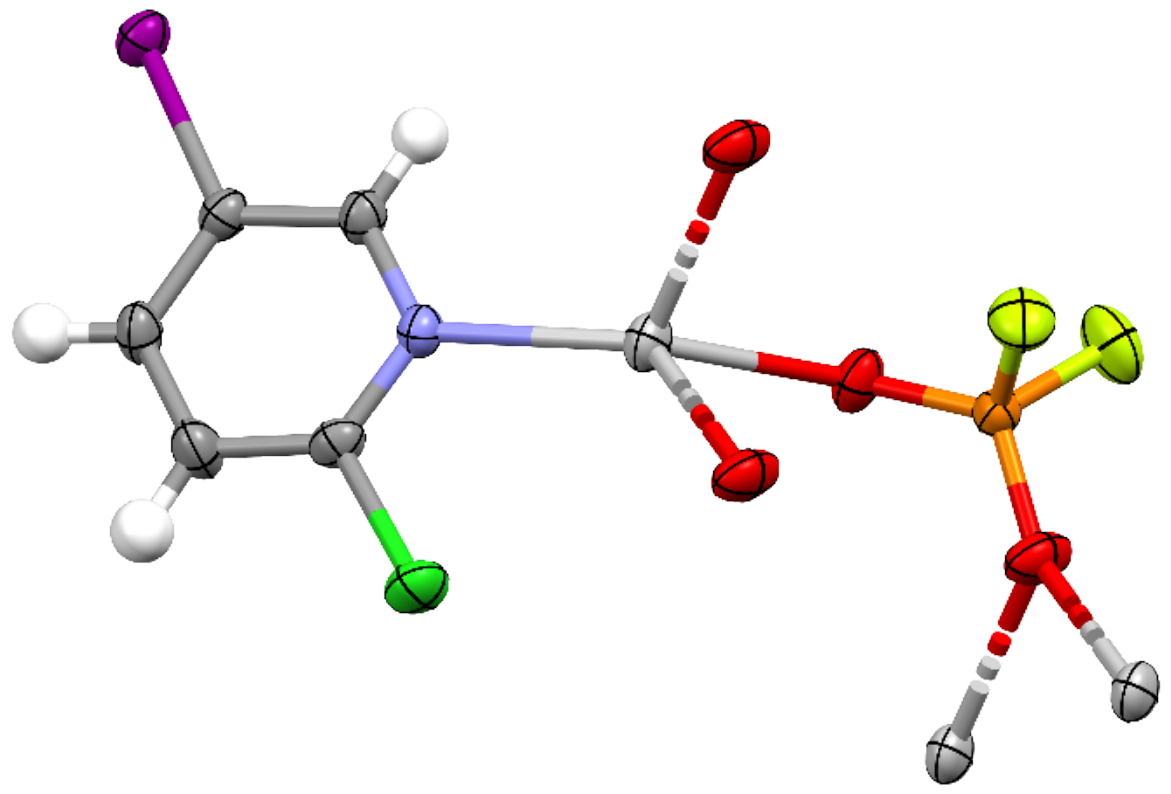

Figure S62. The crystal structure of **6**-Ag-PO_2F_2. Colors: light gray = silver, dark gray = carbon, white = hydrogen, blue = nitrogen, orange = phosphorus, neon green = fluorine, green = chlorine, purple = iodine, red = oxygen.

3.2 Iodine(I) complexes

Thermal ellipsoids are drawn at the 50% probability level in all structures presented. Complexes and counterions partially within the asymmetric unit have been grown.

$[I(3\text{-}MePy)_2]PF_6$ ($[1\text{–}I\text{–}1]PF_6$):

Crystals suitable for X-ray diffraction were obtained through vapor diffusion with pentane. Crystal Data for CCDC-2467903, $C_{12}H_{14}F_6IN_2P$ (M=458.12 g/mol): triclinic, space group P-1 (no. 2), a = 7.0041(4) Å, b = 7.8666(4) Å, c = 8.5083(5) Å, α= 104.859(3)°, β= 95.412(3)°, γ= 114.912(3)°, V= 399.86(4) Å^3, Z = 1, T = 170(1) K, μ(Mo Kα) = 2.159 mm^{-1}, D_{calc} = 1.902 g/cm^3, 1366 reflections measured (5.09° ≤ 2Θ ≤ 50.196°), 1366 unique (R_{sigma} = 0.0674) which were used in all calculations. The final R_1 was 0.0701 (I > 2σ(I)) and wR_2 was 0.1870 (all data).

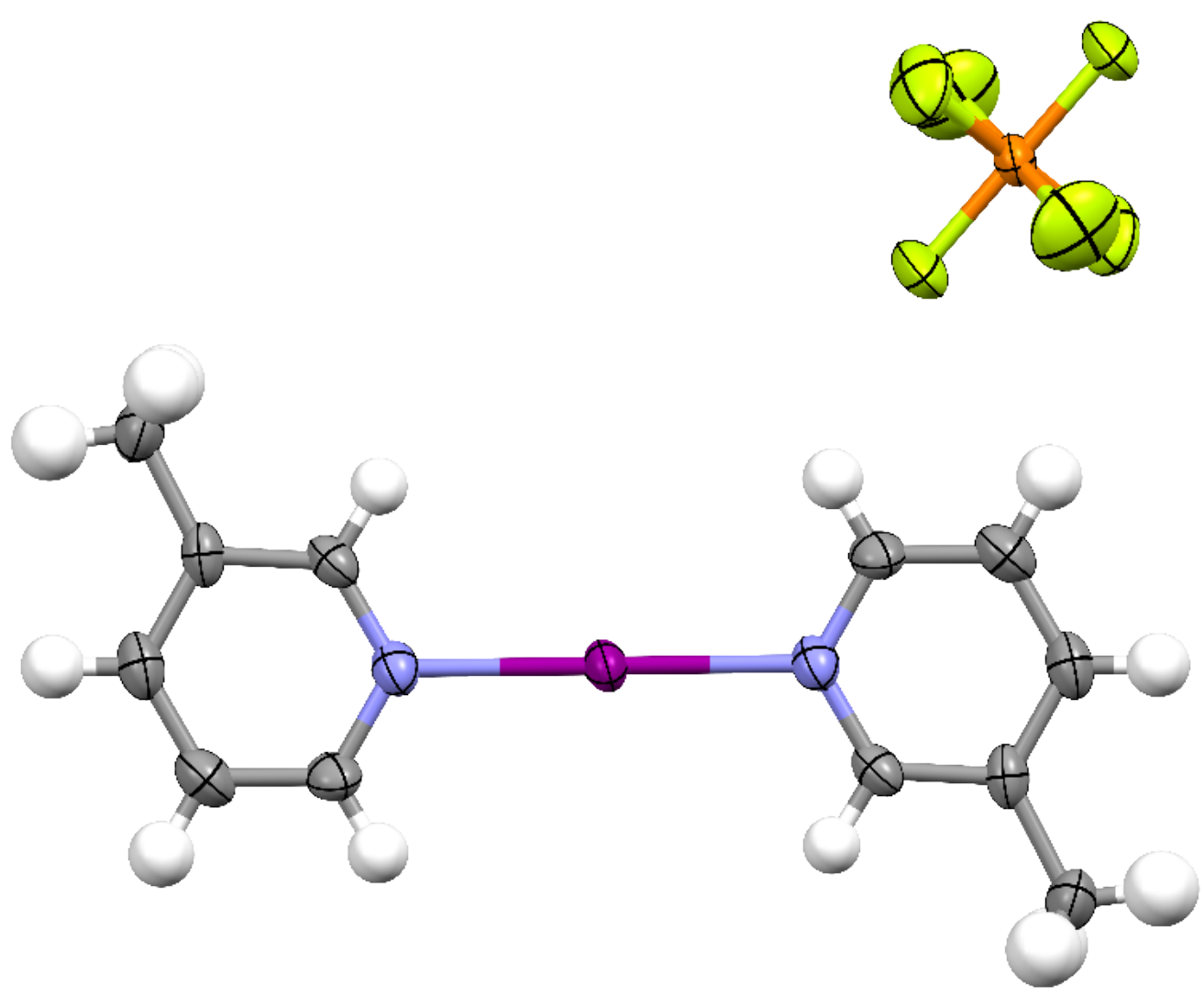

Figure S63. The crystal structure of [1–I–1]PF_6. Colors: purple = iodine, dark gray = carbon, white = hydrogen, blue = nitrogen, orange = phosphorus, neon green = fluorine.

[I(3-FPy)$_2$]PF$_6$ ([2–I–2]PF$_6$):

Crystals suitable for X-ray diffraction were obtained through vapor diffusion with pentane. Crystal Data for CCDC-2467905, $C_{10}H_8F_8IN_2P$ (M=466.05 g/mol): monoclinic, space group C2/c (no. 15), a = 11.8378(2) Å, b = 8.64570(10) Å, c = 14.7323(2) Å, β = 102.8030(10)°, V = 1470.31(4) Å^3, Z = 4, T = 170(1) K, μ(Mo Kα) = 2.370 mm^{-1}, D_{calc} = 2.105 g/cm^3, 13328 reflections measured (5.672° $\leq 2\Theta \leq$ 55.024°), 1695 unique (R_{int} = 0.0311, R_{sigma} = 0.0184) which were used in all calculations. The final R_1 was 0.0195 (I > 2σ(I)) and wR_2 was 0.0379 (all data).

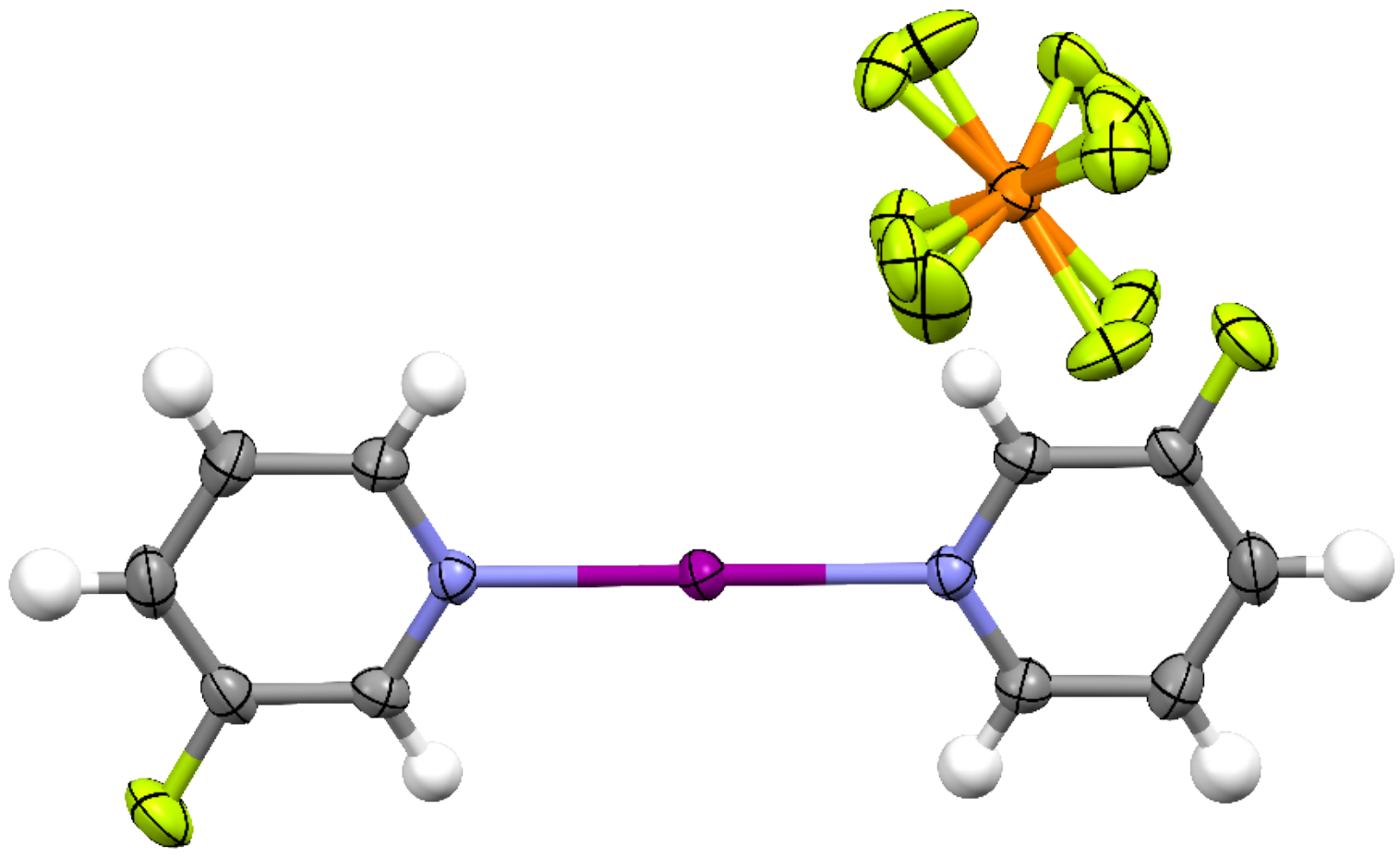

Figure S64. The crystal structure of [**2**–I–**2**]PF$_6$. Colors: purple = iodine, dark gray = carbon, white = hydrogen, blue = nitrogen, orange = phosphorus, neon green = fluorine, green = chlorine. The PF$_6^-$ counterion is disordered over two positions.

[I(3-ClPy)$_2$]PF$_6$ ([3–I–3]PF$_6$):

Crystals suitable for X-ray diffraction were obtained through vapor diffusion with pentane. Crystal Data for CCDC-2467911, $C_{10}H_8Cl_2F_6IN_2P$ (M=498.95 g/mol): monoclinic, space group C2/c (no. 15), a = 11.6914(2) Å, b = 10.6389(2) Å, c = 13.1149(3) Å, β = 109.0260(10)°, V = 1542.16(5) Å^3, Z = 4, T = 170(1) K, μ(Mo Kα) = 2.584 mm^{-1}, D_{calc} = 2.149 g/cm^3, 8160 reflections measured (5.314° ≤ 2Θ ≤ 54.964°), 1763 unique (R_{int} = 0.0285, R_{sigma} = 0.0248) which were used in all calculations. The final R_1 was 0.0212 (I > 2σ(I)) and wR_2 was 0.0439 (all data).

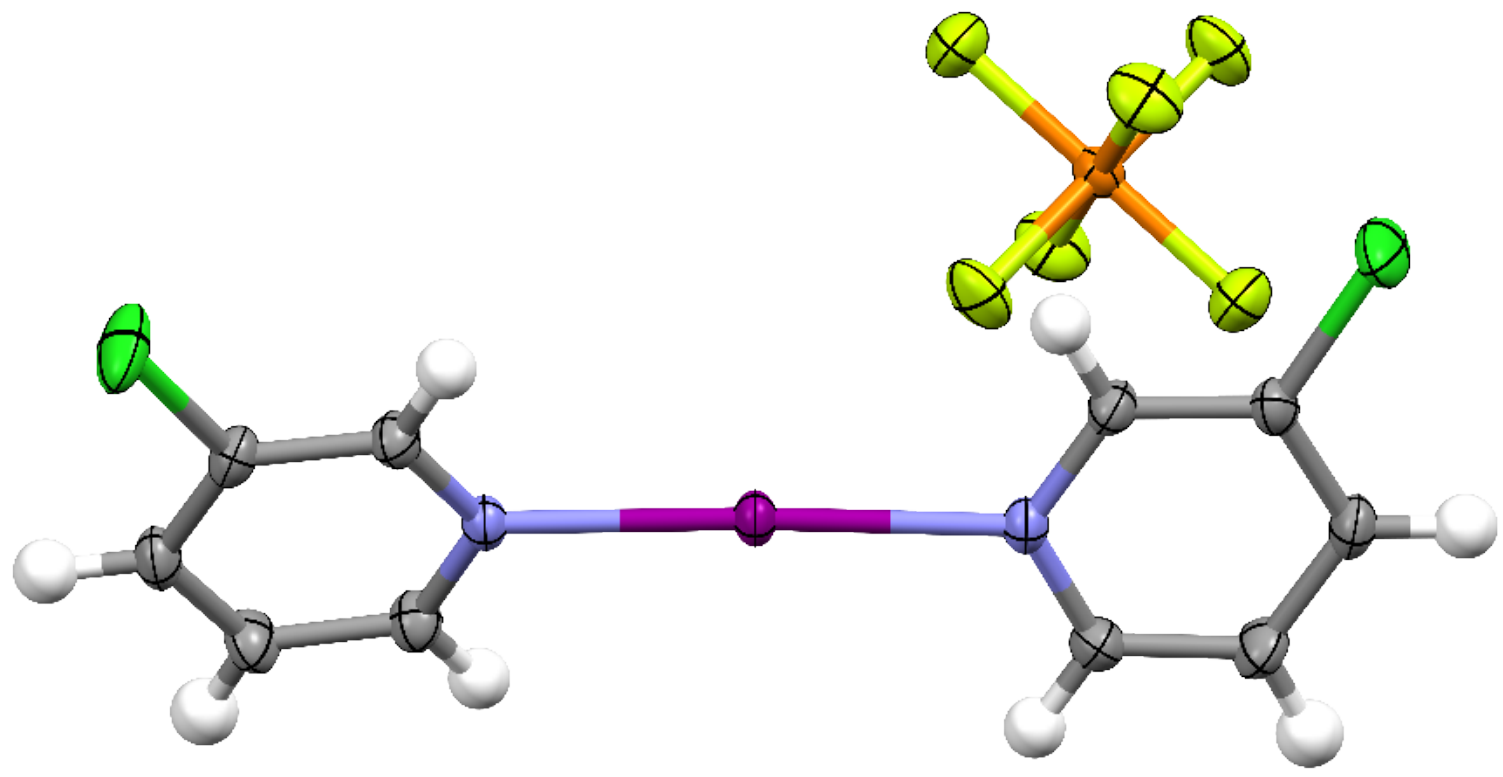

Figure S65. The crystal structure of [**3**–I–**3**]PF$_6$. Colors: purple = iodine, dark gray = carbon, white = hydrogen, blue = nitrogen, orange = phosphorus, neon green = fluorine, green = chlorine.

$[I(3\text{-}BrPy)_2]PF_6$ ($[4\text{–}I\text{–}4]PF_6$):

Crystals suitable for X-ray diffraction were obtained through vapor diffusion with pentane. Crystal Data for CCDC-2481496, $C_{10}H_8Br_2F_6IN_2P$ (M=587.87 g/mol): orthorhombic, space group Ibca (no. 73), a = 15.9398(2) Å, b = 16.0917(2) Å, c = 25.4136(4) Å, V = 6518.55(15) Å^3, Z = 16, T = 170(1) K, μ(Mo Kα) = 7.022 mm^{-1}, D_{calc} = 2.396 g/cm^3, 53352 reflections measured (3.596° ≤ 2Θ ≤ 53.998°), 3570 unique (R_{int} = 0.0806, R_{sigma} = 0.0363) which were used in all calculations. The final R_1 was 0.0339 (I > 2σ(I)) and wR_2 was 0.0617 (all data).

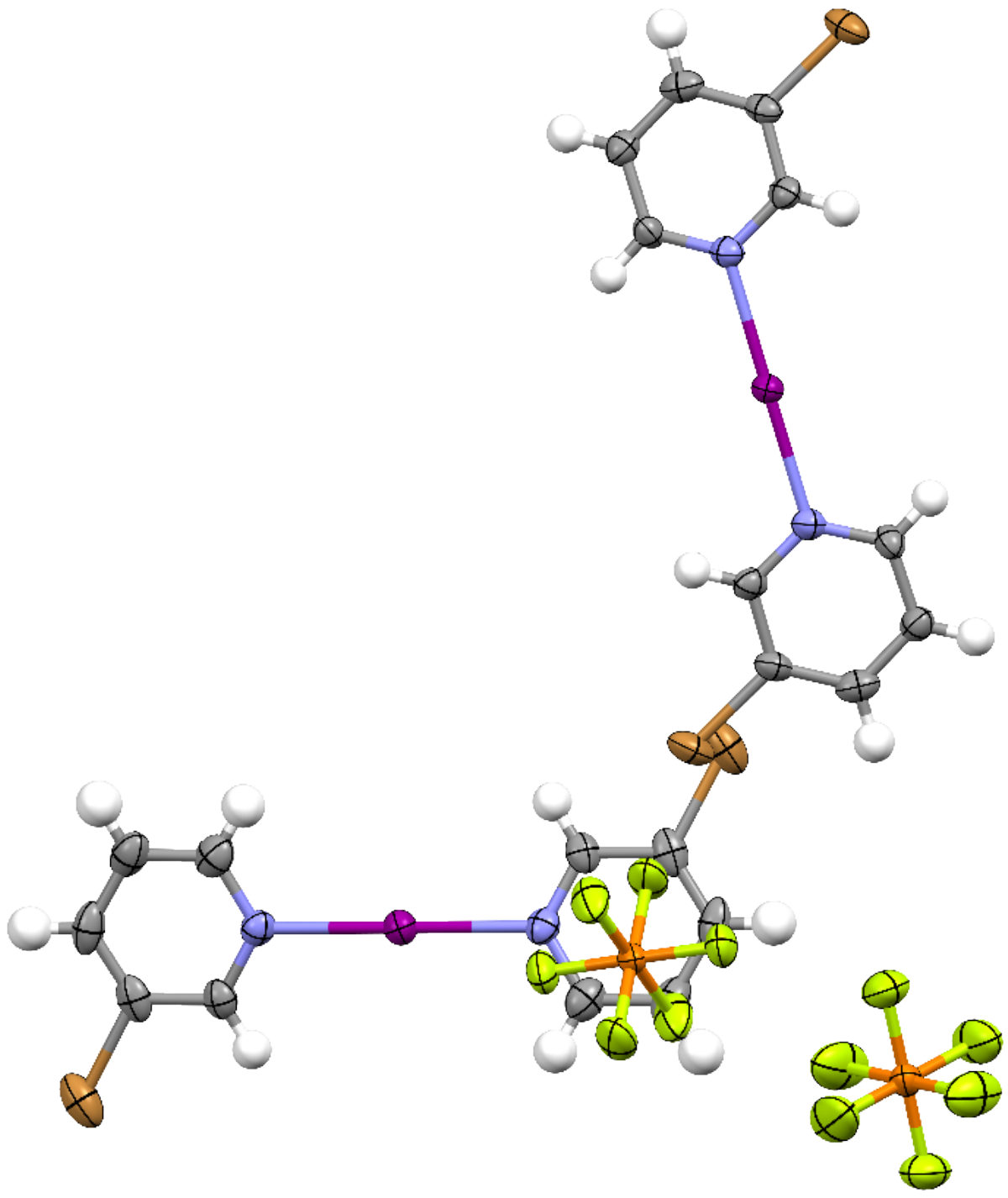

Figure S66. The crystal structure of [**4**–I–**4**]PF_6. Colors: purple = iodine, dark gray = carbon, white = hydrogen, blue = nitrogen, orange = phosphorus, neon green = fluorine, brown = bromine.

[I(3-IPy)$_2$]PF$_6$ ([5–I–5]PF$_6$):

Crystals suitable for X-ray diffraction were obtained through vapor diffusion with pentane. Crystal Data for CCDC-2467909, $C_{10}H_8F_6I_3N_2P$ (M=681.85 g/mol): monoclinic, space group P2$_1$/c (no. 14), a = 8.34210(10) Å, b = 15.2705(3) Å, c = 13.5415(2) Å, β = 101.5861(9)°, V = 1689.88(5) Å^3, Z = 4, T = 170(1) K, μ(Mo Kα) = 5.697 mm^{-1}, D_{calc} = 2.680 g/cm^3, 30595 reflections measured (4.068° ≤ 2Θ ≤ 54.984°), 3877 unique (R_{int} = 0.0488, R_{sigma} = 0.0306) which were used in all calculations. The final R_1 was 0.0449 (I > 2σ(I)) and wR_2 was 0.0830 (all data).

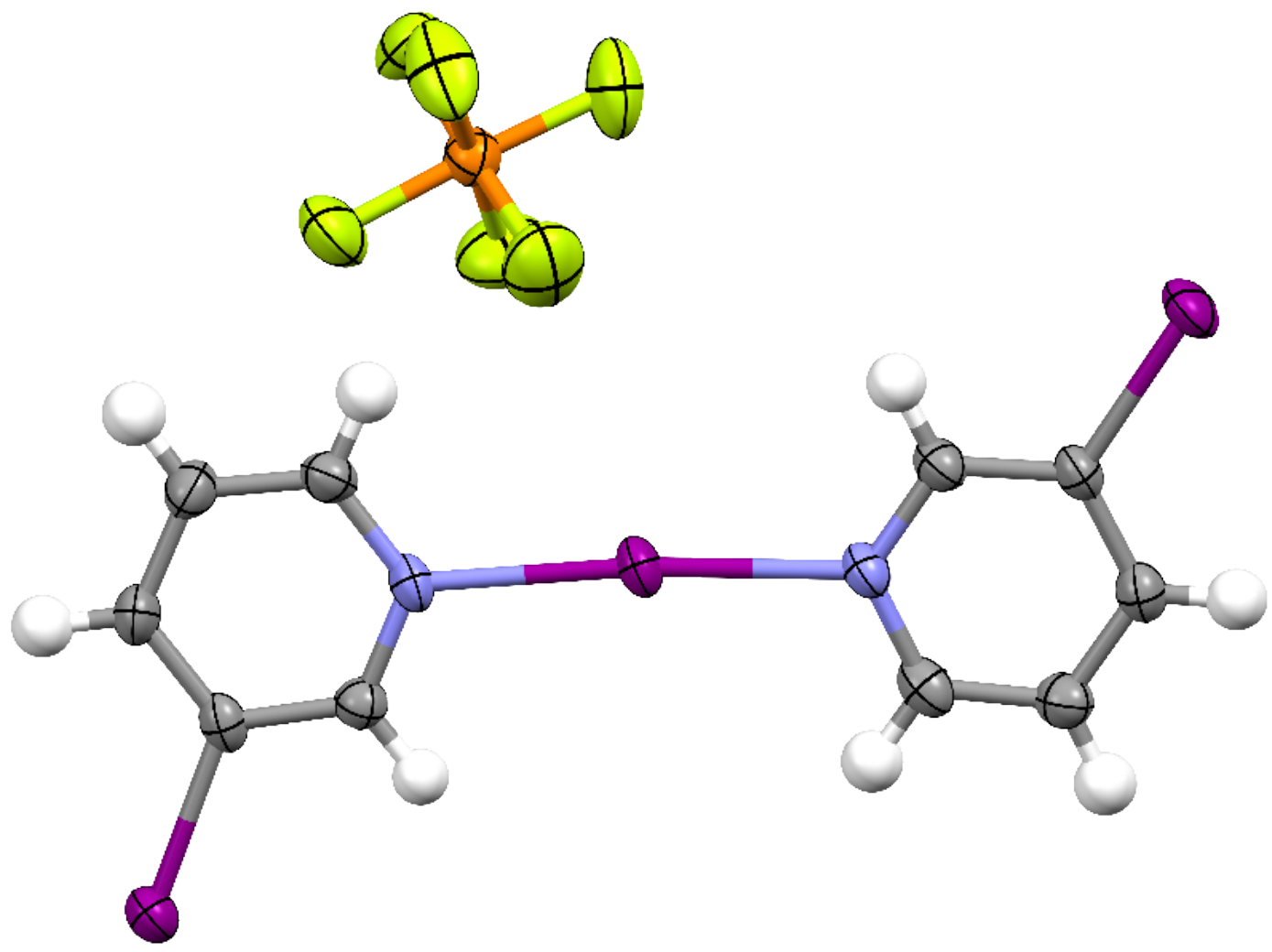

Figure S67. The crystal structure of [**5**–I–**5**]PF$_6$. Colors: purple = iodine, dark gray = carbon, white = hydrogen, blue = nitrogen, orange = phosphorus, neon green = fluorine.

[I(2-Cl-5-IPy)$_2$]PF$_6$ ([6–I–6]PF$_6$):

Attempts to grow crystals suitable for X-ray diffraction were unsuccessful as only crystals of the pure ligand **6** were obtained.

4. Hirshfeld surface analysis

To illustrate and highlight the differences in packing interactions between the silver(I) complexes [**L**–Ag–**L**]$^+$ and iodine(I) complexes [**L**–I–**L**]$^+$, Hirshfeld surfaces of the respective complexes were generated using CrystalExplorer[5] and analyzed in detail (**Figure S68**). The Hirshfeld surfaces mapped with the normalized contact distance d_{norm}[6] clearly show that the central iodine(I) cations in [**L**–I–**L**]$^+$ are surrounded by two blue bumps, indicating contact distances above the sum of vdW radii. This observation is consistent with the electron-rich surface of iodine(I) perpendicular to the N–I$^+$–N bond, which suppresses iodine(I)⋯PF_6^- interactions. The surfaces around the cation are otherwise white or light blue as the closest contacts to PF_6^- counterions tend to be close to or slightly above the sum of vdW radii, with [**5**–I–**5**]$^+$ being the only exception. In contrast, the silver(I) cations are surrounded by red hollows or spots, caused by Ag⋯Ag interactions and contacts to PF_6^- and BF_4^- counterions at distances shorter than the sum of vdW radii.

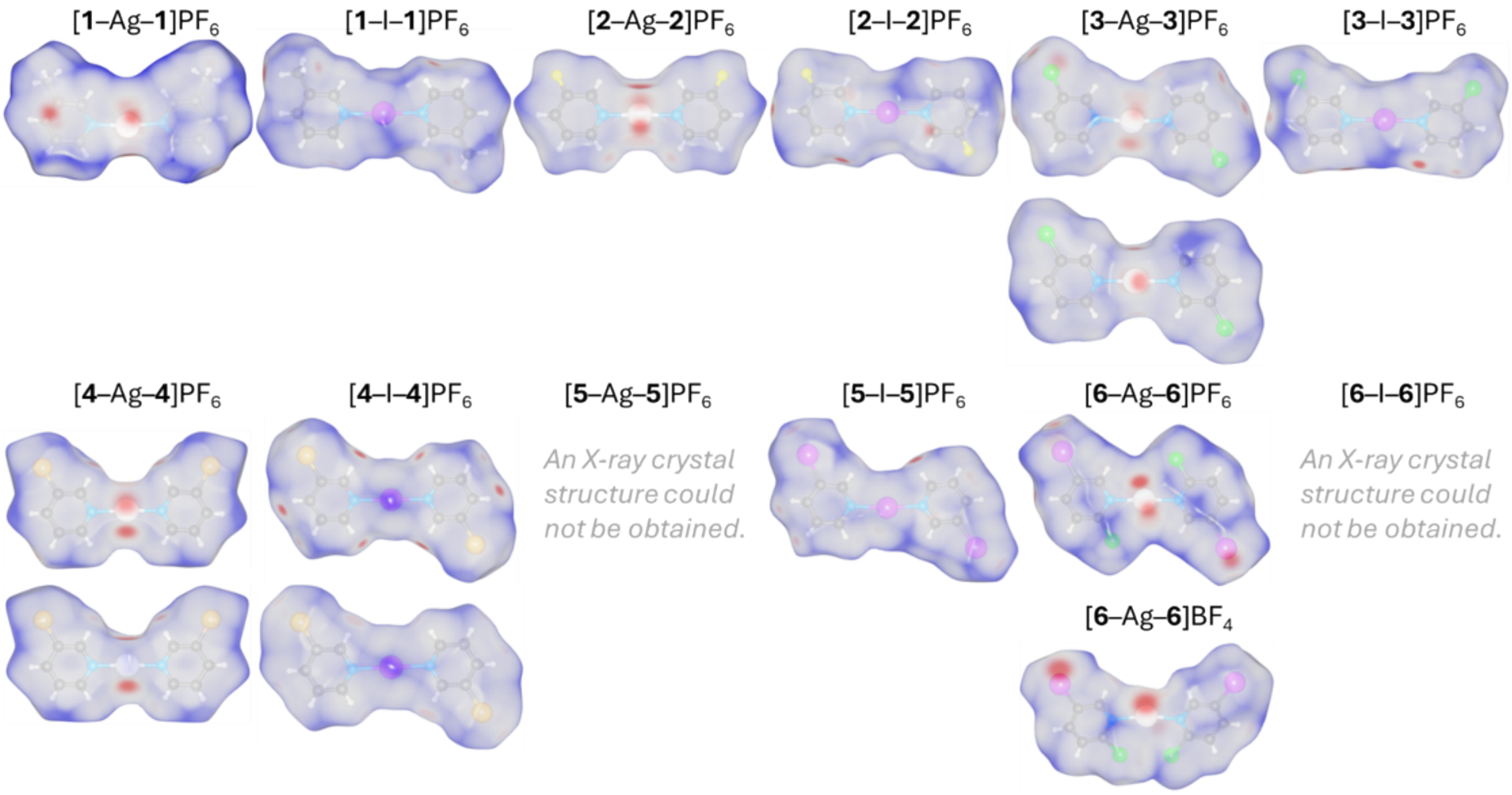

Figure S68. Hirshfeld surfaces (d_{norm}) of silver(I) complexes [**L**–Ag–**L**]$^+$ and iodine(I) complexes [**L**–I–**L**]$^+$. For [**3**–Ag–**3**]$^+$, [**4**–Ag–**4**]$^+$ and [**4**–I–**4**]$^+$, surfaces were generated for both crystallographically independent molecules.

To obtain further insights into the intermolecular interactions present, two-dimensional fingerprint plots were generated. The plots of the silver(I) and iodine(I) complexes of **2**–**5** (**Figure S69**) show that contacts involving the halogen substituents account for an increasing proportion of the total surface area with increasing atomic radius. These contacts also become more specific with the X⋯X contacts residing on a narrow line on the diagonal, indicating increased capacity for halogen bonding especially for **4** and **5**. Additionally, the fingerprint plots of [**4**–Ag–**4**]PF_6 and [**5**–I–**5**]PF_6 show narrow lines for Br⋯F and I⋯F contacts, respectively, again demonstrating the ability of **4** and **5** to form intermolecular halogen

bonds with the PF_6^- counterions. Interestingly, the Br⋯Br line is practically absent in [**4**–Ag–**4**]PF_6 while Br⋯F contacts are present, although at distances above the sum of vdW radii.

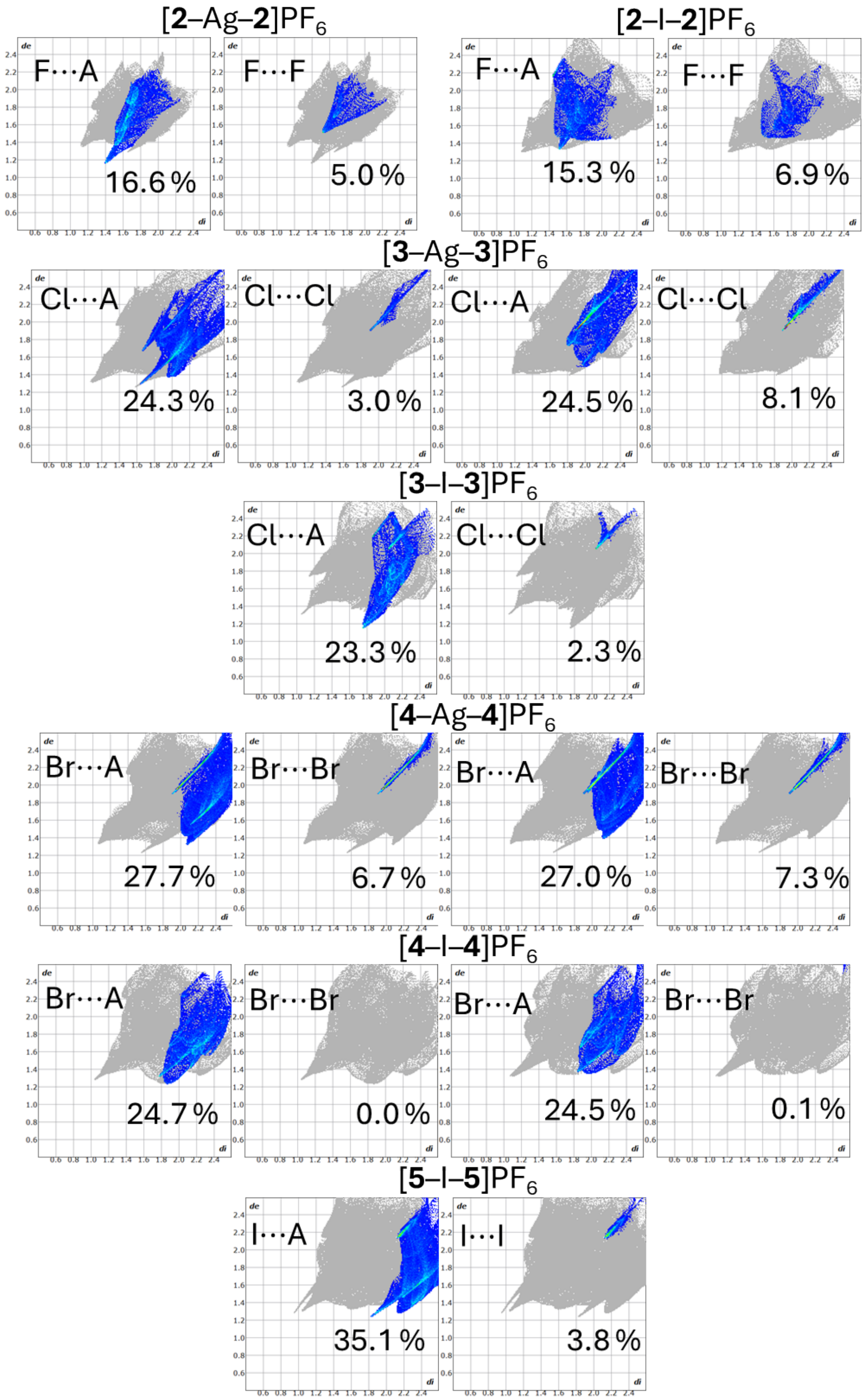

Figure S69. Fingerprint plots of the silver(I) and iodine(I) complexes of **2**–**5**. The percentages show the contribution of each contact to the total Hirshfeld surface area. Plots were generated for all crystallographically independent molecules.

The greater variety in intermolecular interactions for the silver(I) complexes is reflected in the fingerprint plots of [**1**–Ag–**1**]PF_6 and [**1**–I–**1**]PF_6 (**Figure S70**). With only weak interactions between the central cation and counterion and no argentophilic interactions to facilitate contacts to a neighboring [**1**–I–**1**]$^+$ unit, the less specific C⋯H/H⋯C, N⋯H/H⋯N and H⋯H contacts exhibit a wide range of d_i and d_e, resulting in wings in the fingerprint plot. The difference in the percentage of H⋯H contacts (40.4 % for [**1**–Ag–**1**]PF_6 *vs.* 32.3 % for [**1**–I–**1**]PF_6) illustrates the difference in the location of PF_6^- ions: in silver(I) complexes these anions are located near the metal centers, while the pyridine rings in the iodine(I) complex are surrounded by the counterions, leading to an increase in H⋯F contacts instead. The offset π-stacking in [**1**–I–**1**]PF_6 also contributes to this, with H⋯I/I⋯H contacts (7.9 %) being more prevalent than H⋯Ag/Ag⋯H contacts are in [**1**–Ag–**1**]PF_6 (1.1 %).

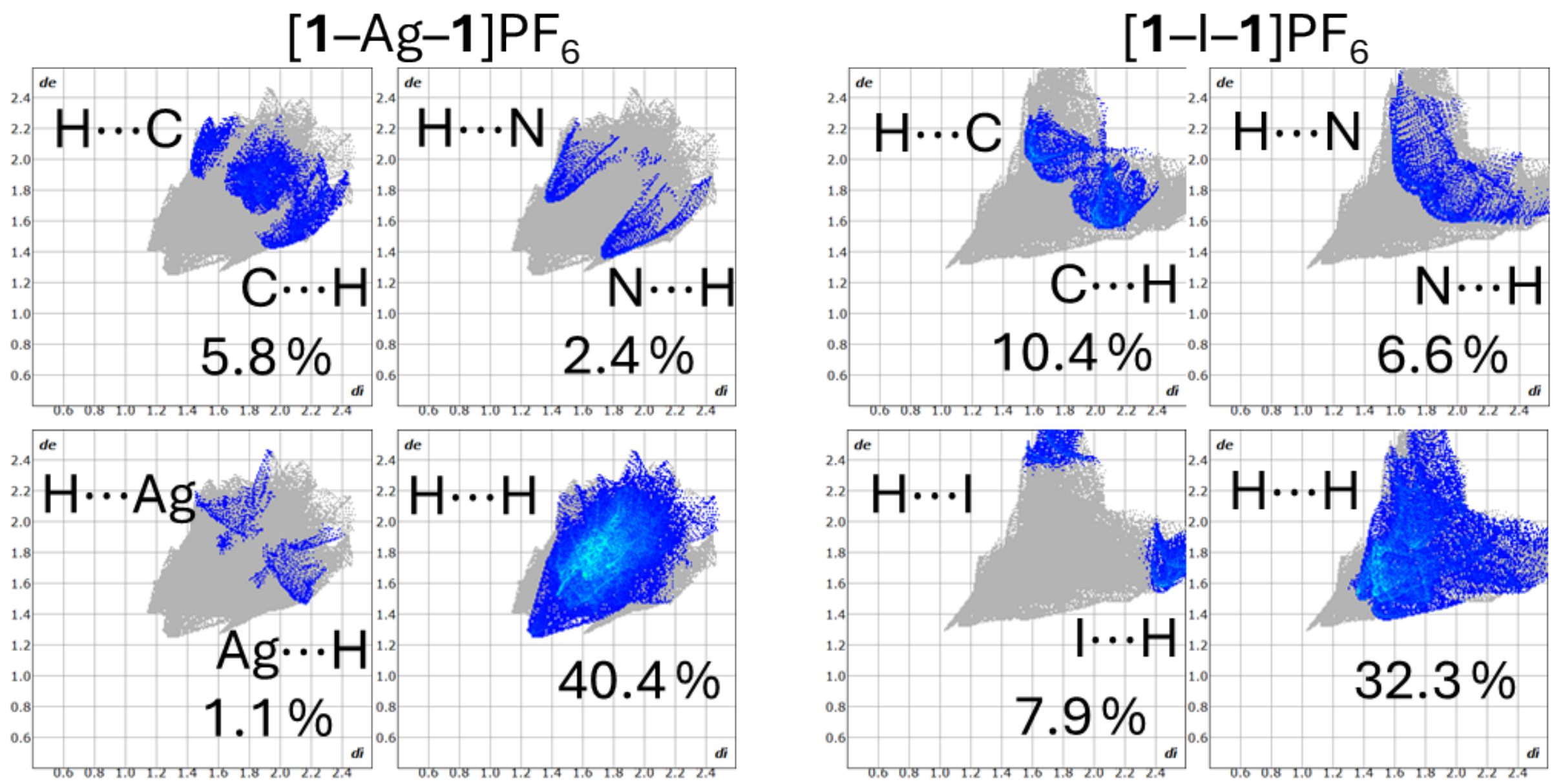

Figure S70. Fingerprint plots of the silver(I) and iodine(I) complexes of **1**. The percentages show the contribution of each contact to the total Hirshfeld surface area.

The fingerprint plots of [**6**–Ag–**6**]PF_6 and [**6**–Ag–**6**]BF_4 appear strikingly different (**Figure S71**). As these are structures of the same complex, most contacts are present in both, with differences stemming from the counterion, geometry of the complex as well as the interactions between [**6**–Ag–**6**]$^+$ units. The BF_4^- counterion resides close to the silver(I) center in [**6**–Ag–**6**]BF_4 and thus the *ortho*-hydrogens in the pyridine ring, resulting in the wing in the H⋯F plot reaching lower d_i and d_e values than for [**6**–Ag–**6**]PF_6. The I⋯Cl/Cl⋯I contacts form a sharp line for [**6**–Ag–**6**]BF_4, whereas the same plot for [**6**–Ag–**6**]PF_6 is more disperse. This is because the bent, pseudo-*syn* geometry of [**6**–Ag–**6**]$^+$ in the BF_4^- salt allows the two chloro-substituents of a [**6**–Ag–**6**]$^+$ complex to easily interact with the iodo-substituent of another, something which is not possible in the planar *anti* conformation found in the PF_6^- salt. A further consequence of this difference in conformation and interactions between the [**6**–Ag–**6**]$^+$ units is that I⋯I contacts are present in [**6**–Ag–**6**]BF_4 but absent in [**6**–Ag–**6**]PF_6.

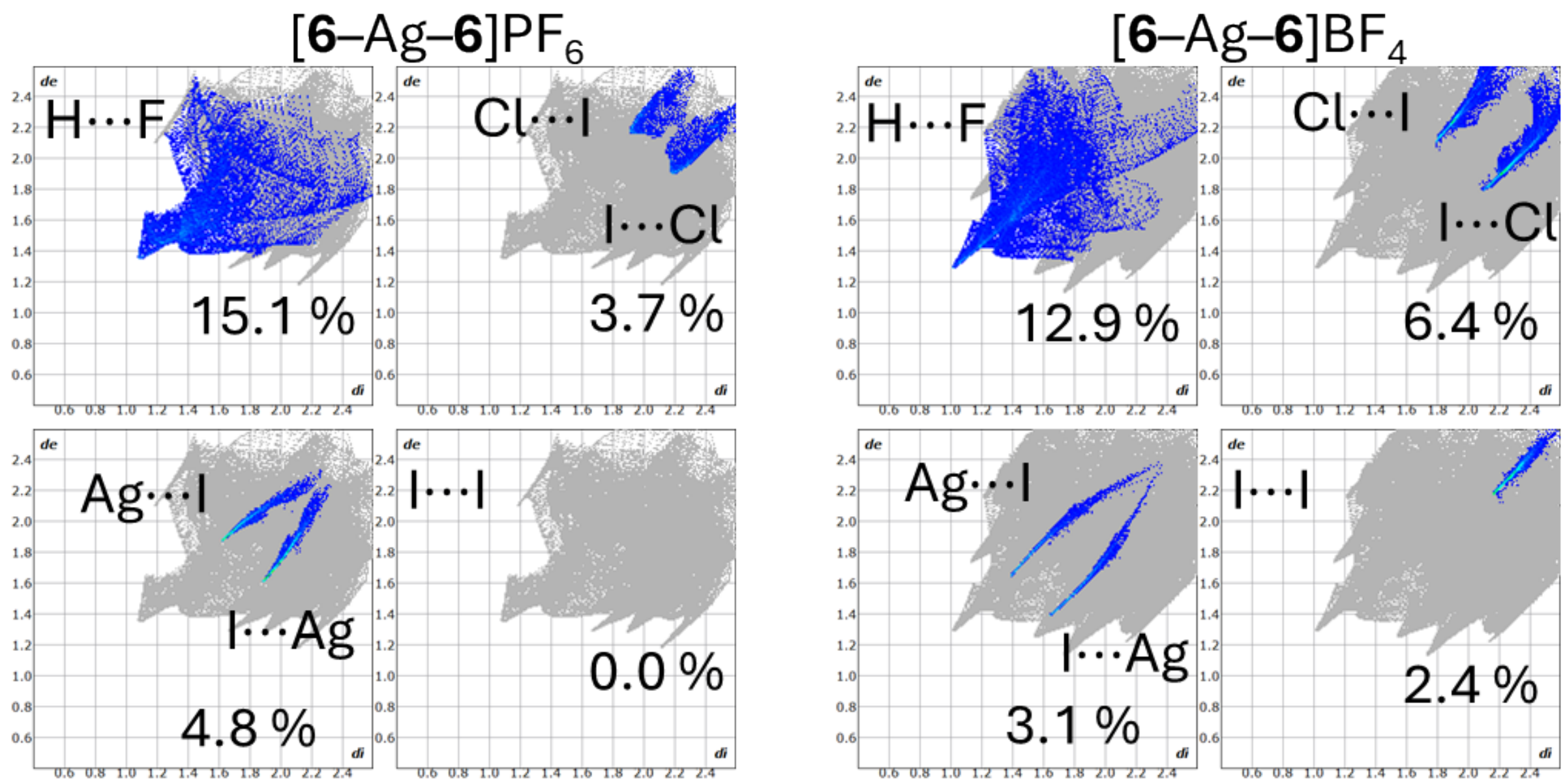

Figure S71. Fingerprint plots of the two structures of the silver(I) complex of **6**. The percentages show the contribution of each contact to the total Hirshfeld surface area.

For the fingerprint plots, the counterions in [**2**–I–**2**]PF_6 and [**4**–Ag–**4**]PF_6 were not modeled as disordered to maintain consistency with other structures, and the DCM molecules present in [**3**–Ag–**3**]PF_6 were masked to avoid conflating contacts involving the chloro-substituent of the pyridine ligands and those of the DCM chlorine atoms.

5. Powder diffraction and Pawley fitting

Powder diffraction samples were obtained by evaporating the solutions prepared as described above under nitrogen flow. Experimental PXRD patterns and patterns simulated from the respective crystal structures using Mercury (Build 454209)[7] are presented for all complexes, except [**5**–Ag–**5**]PF_6 for which only the experimental pattern is available, as a crystal structure could not be obtained, and [**6**–I–**6**]PF_6 for which neither is available. Lattice parameters from the Pawley fits are presented in Table S2.

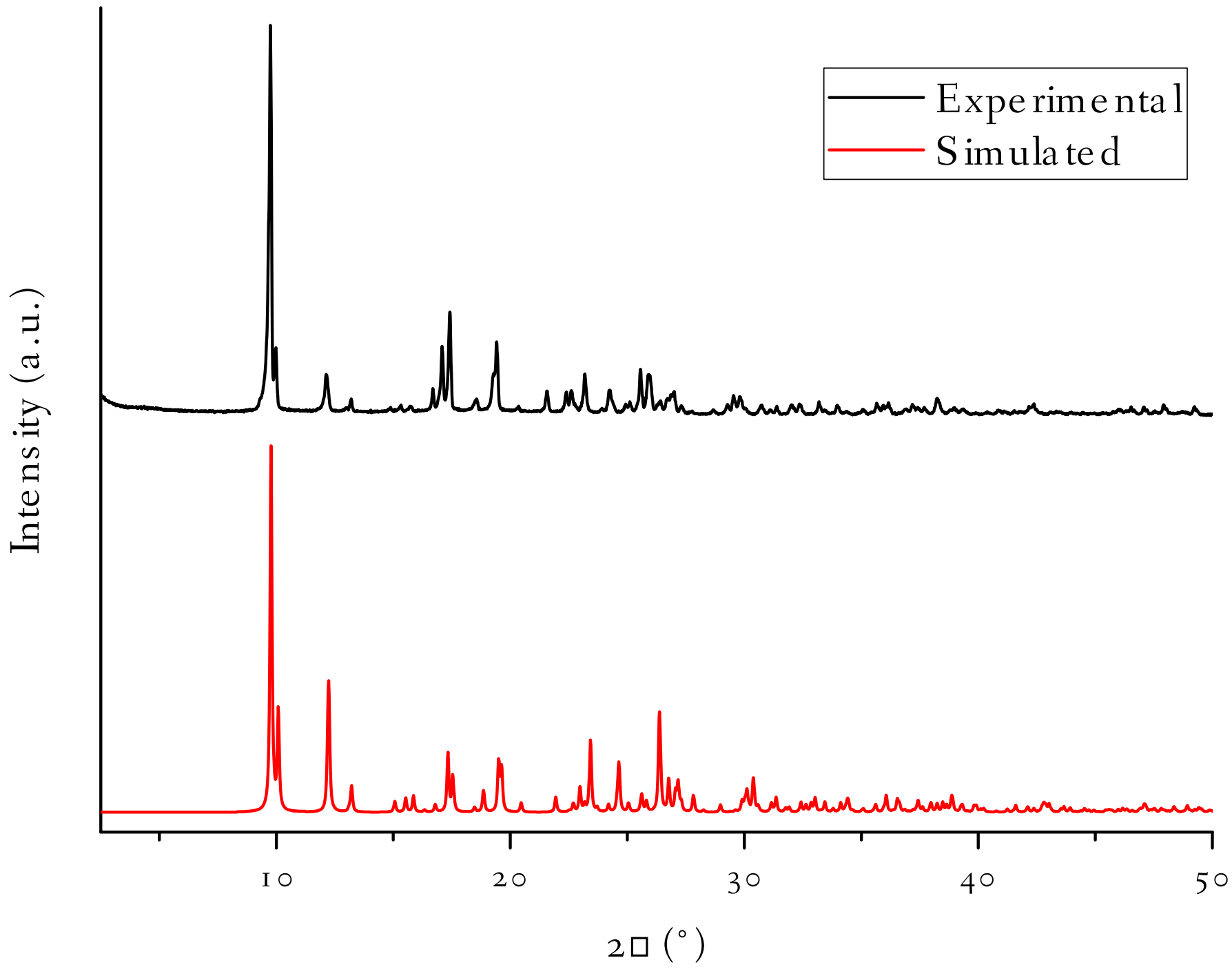

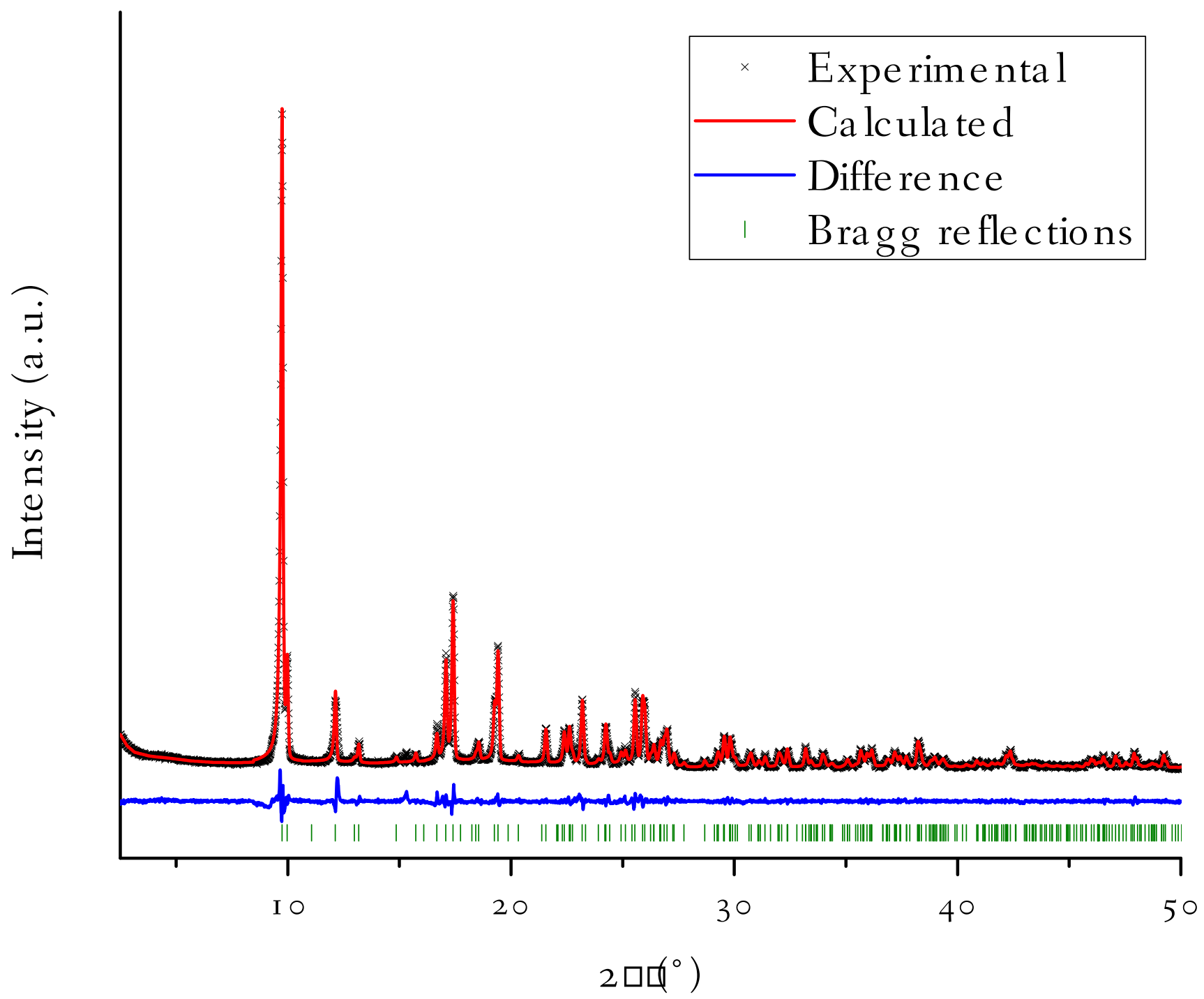

Figure S72. Simulated and experimental PXRD patterns (top) and Pawley fit (bottom) of [1–Ag–1]PF_6.

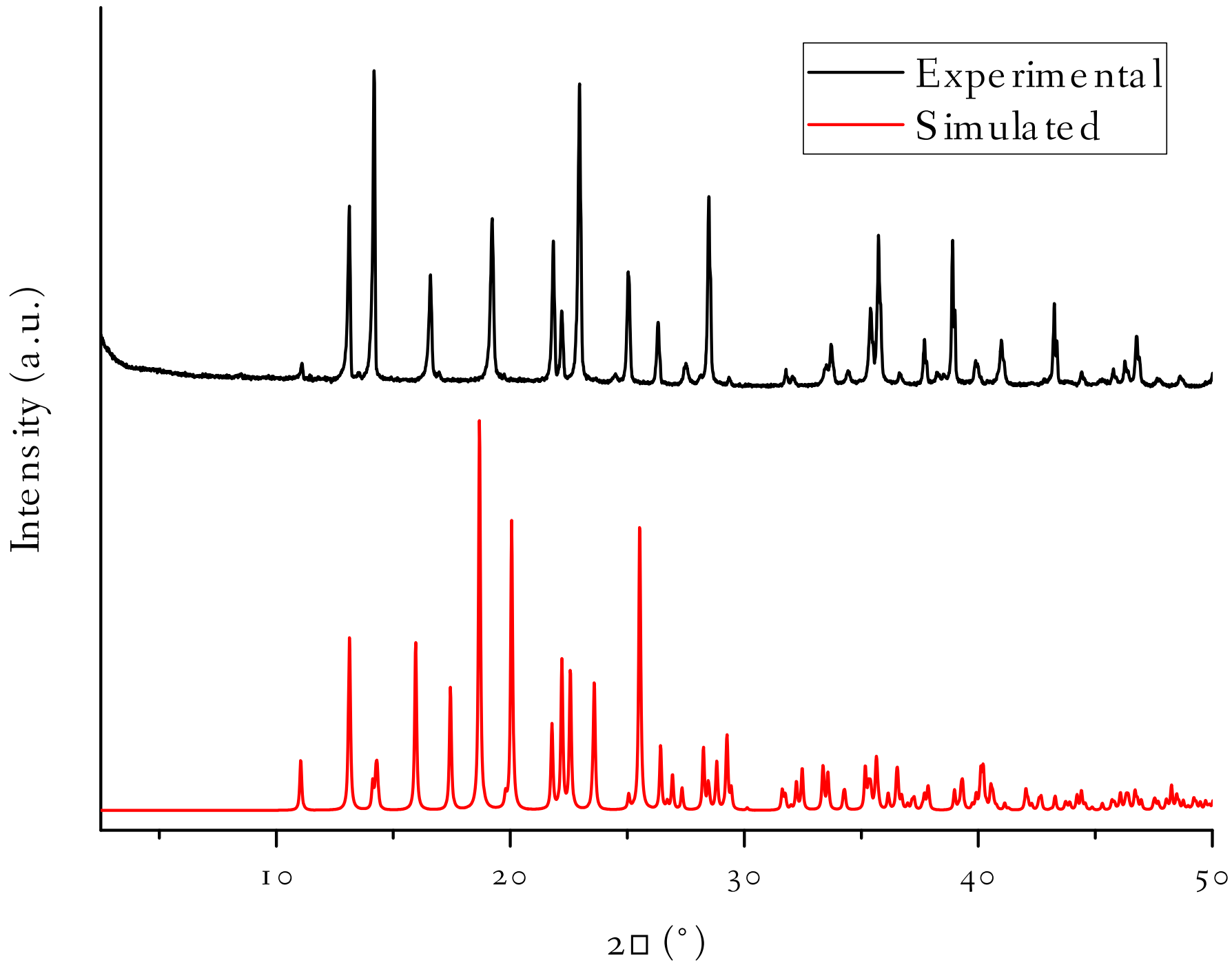

Figure S73. Simulated and experimental PXRD patterns of [1–I–1]PF_6.

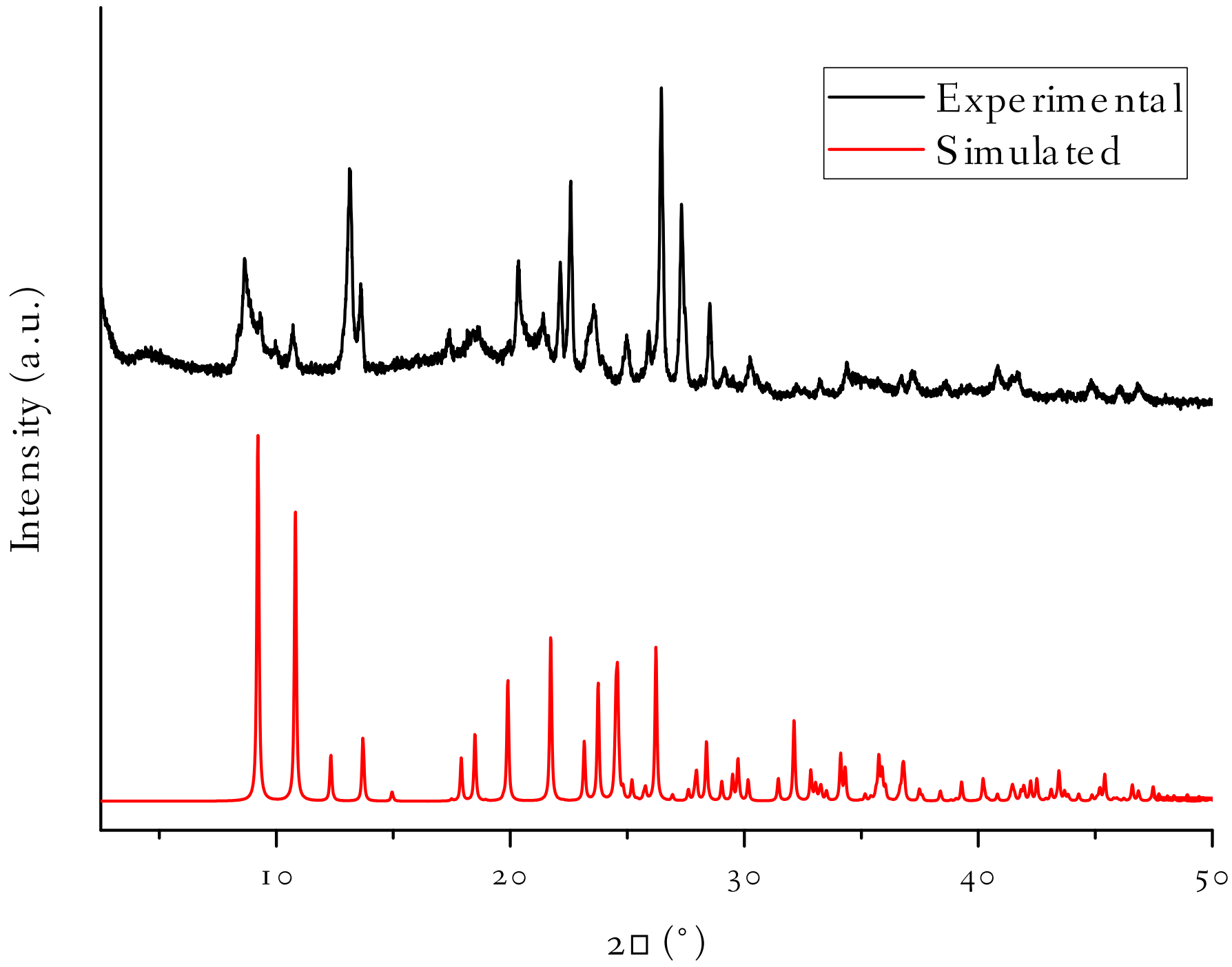

Figure S74. Simulated and experimental PXRD patterns of [**2**–Ag–**2**]PF_6.

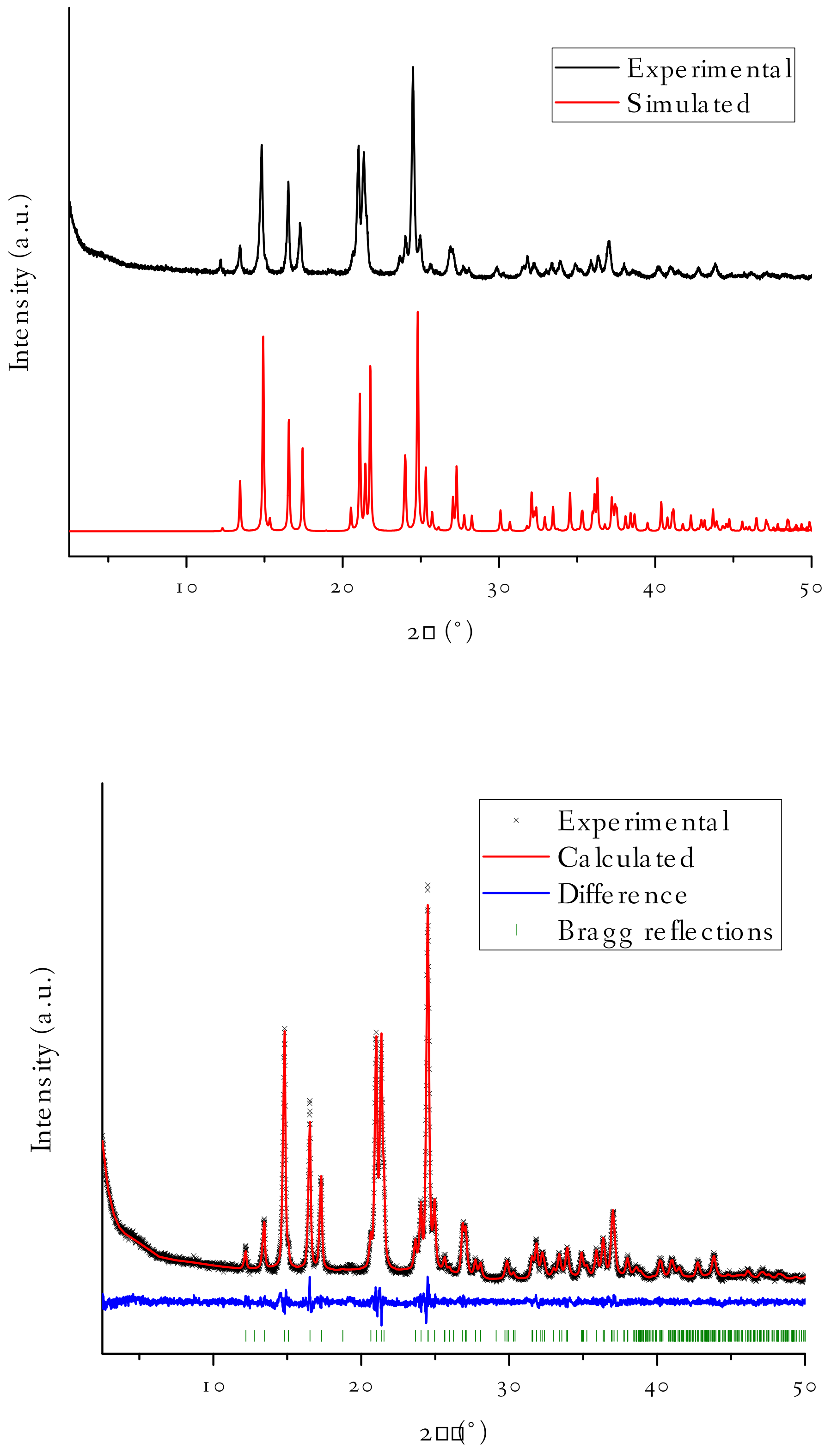

Figure S75. Simulated and experimental PXRD patterns (top) and Pawley fit (bottom) of [**2**–I–**2**]PF_6.

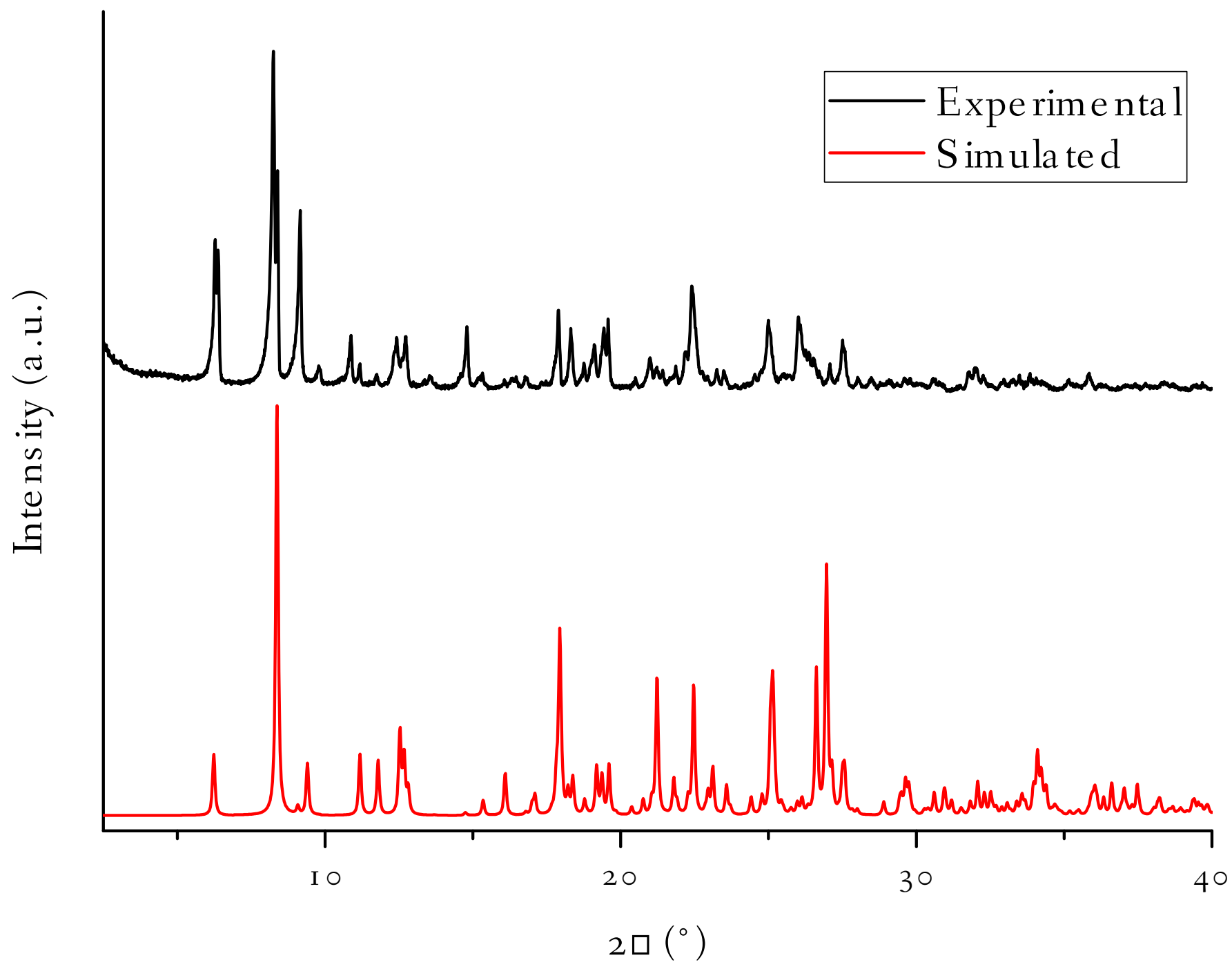

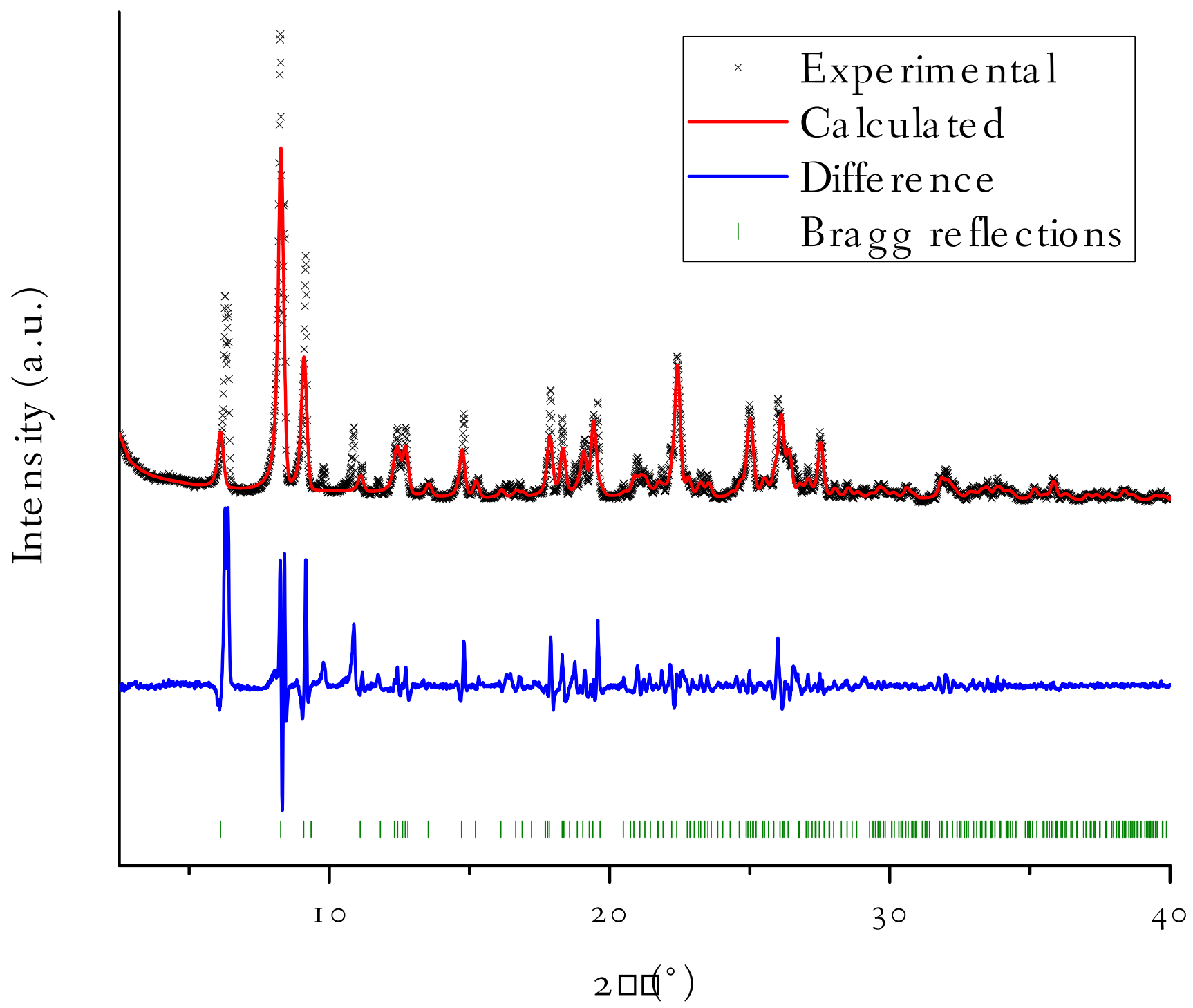

Figure S76. Simulated and experimental PXRD patterns (top) and Pawley fit (bottom) of [**3**–Ag–**3**]PF_6.

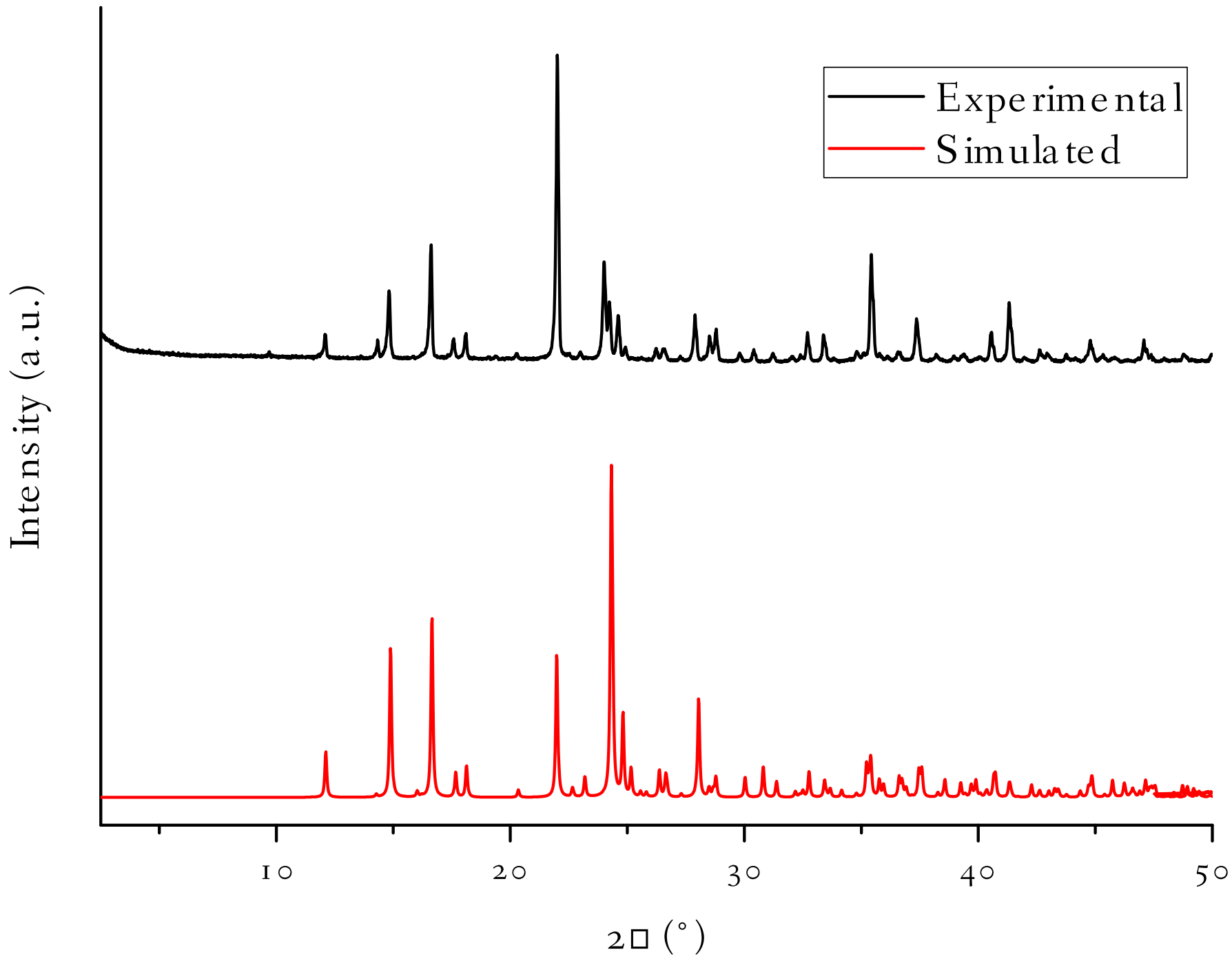

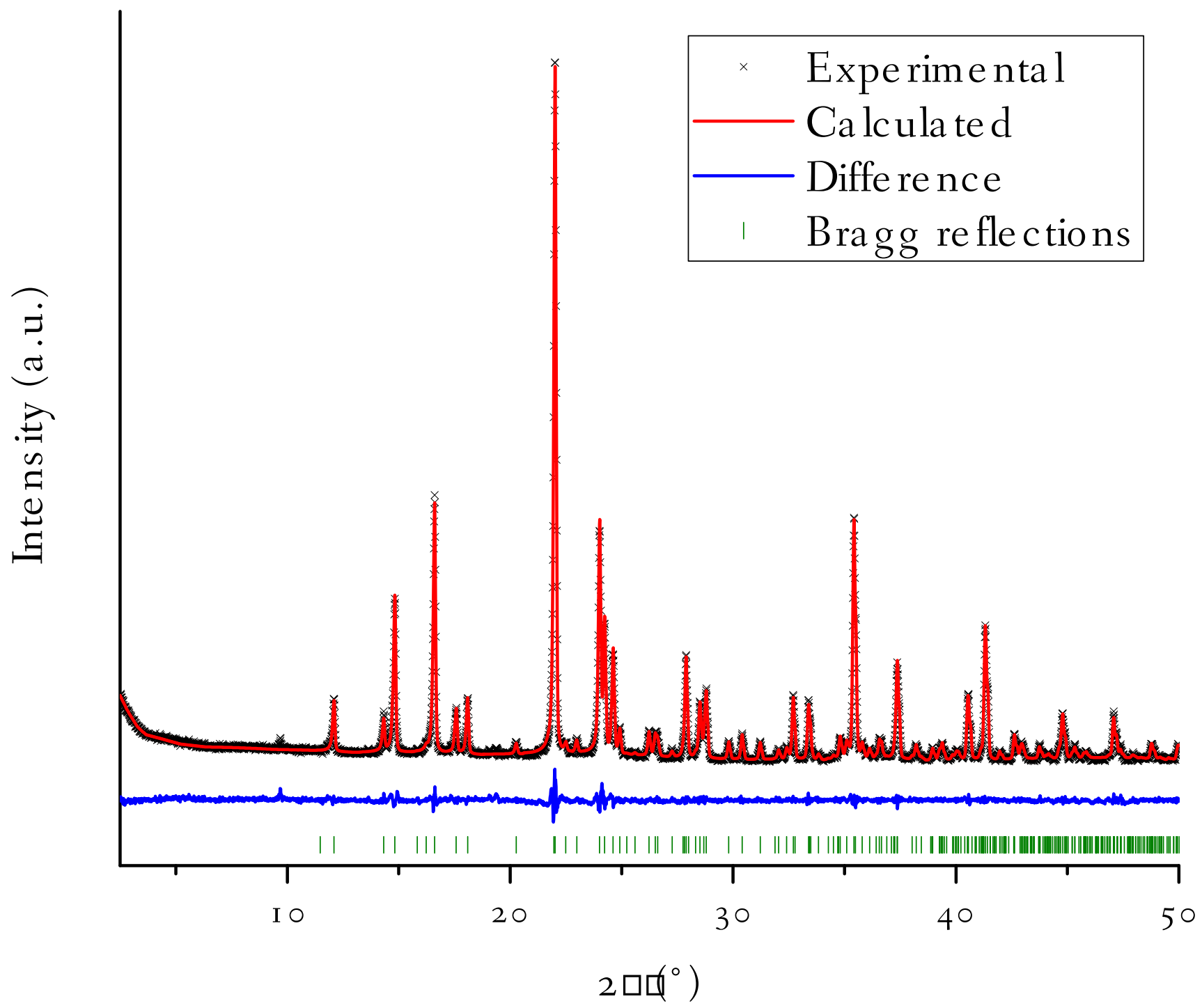

Figure S77. Simulated and experimental PXRD patterns (top) and Pawley fit (bottom) of [**3**–I–**3**]PF_6.

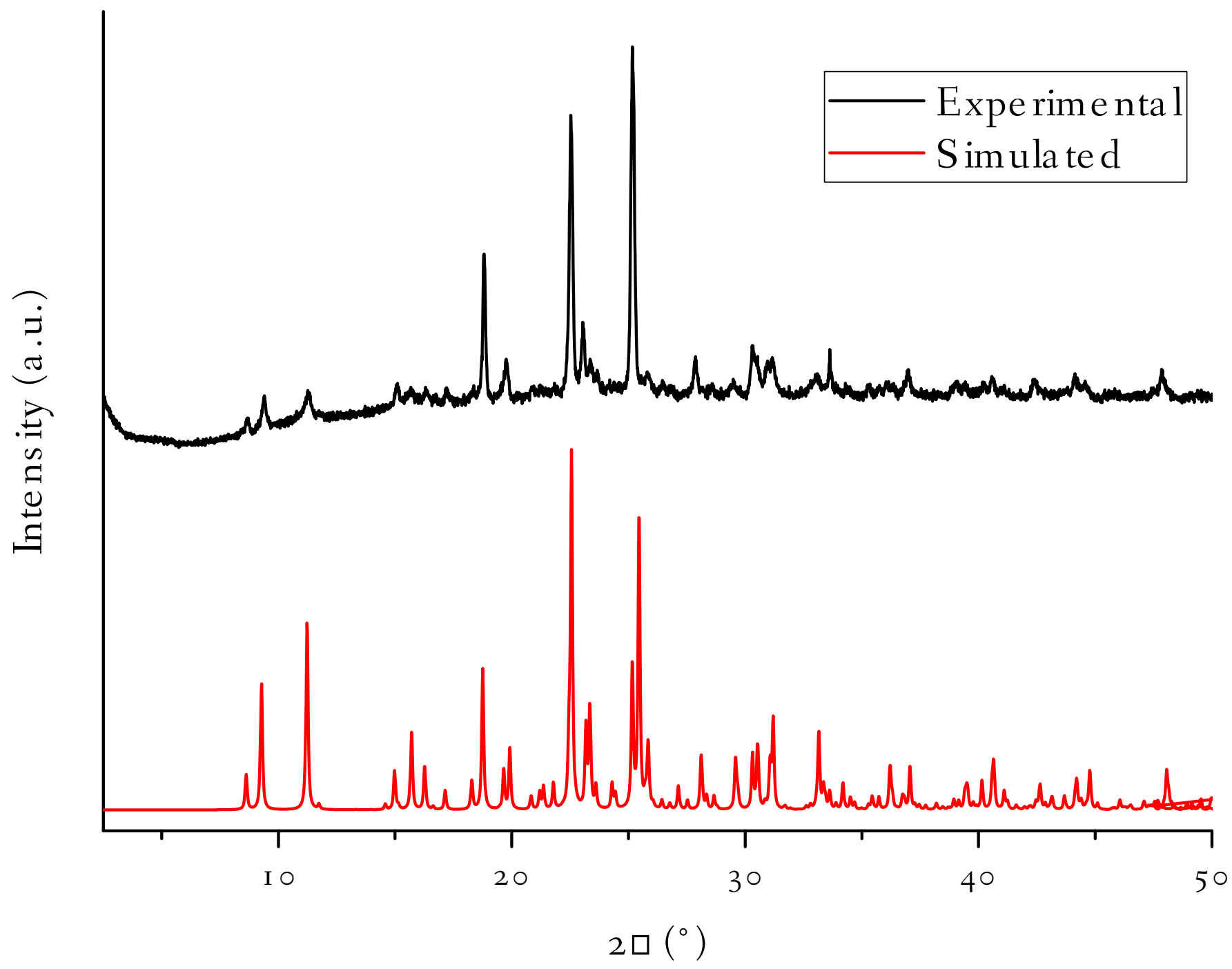

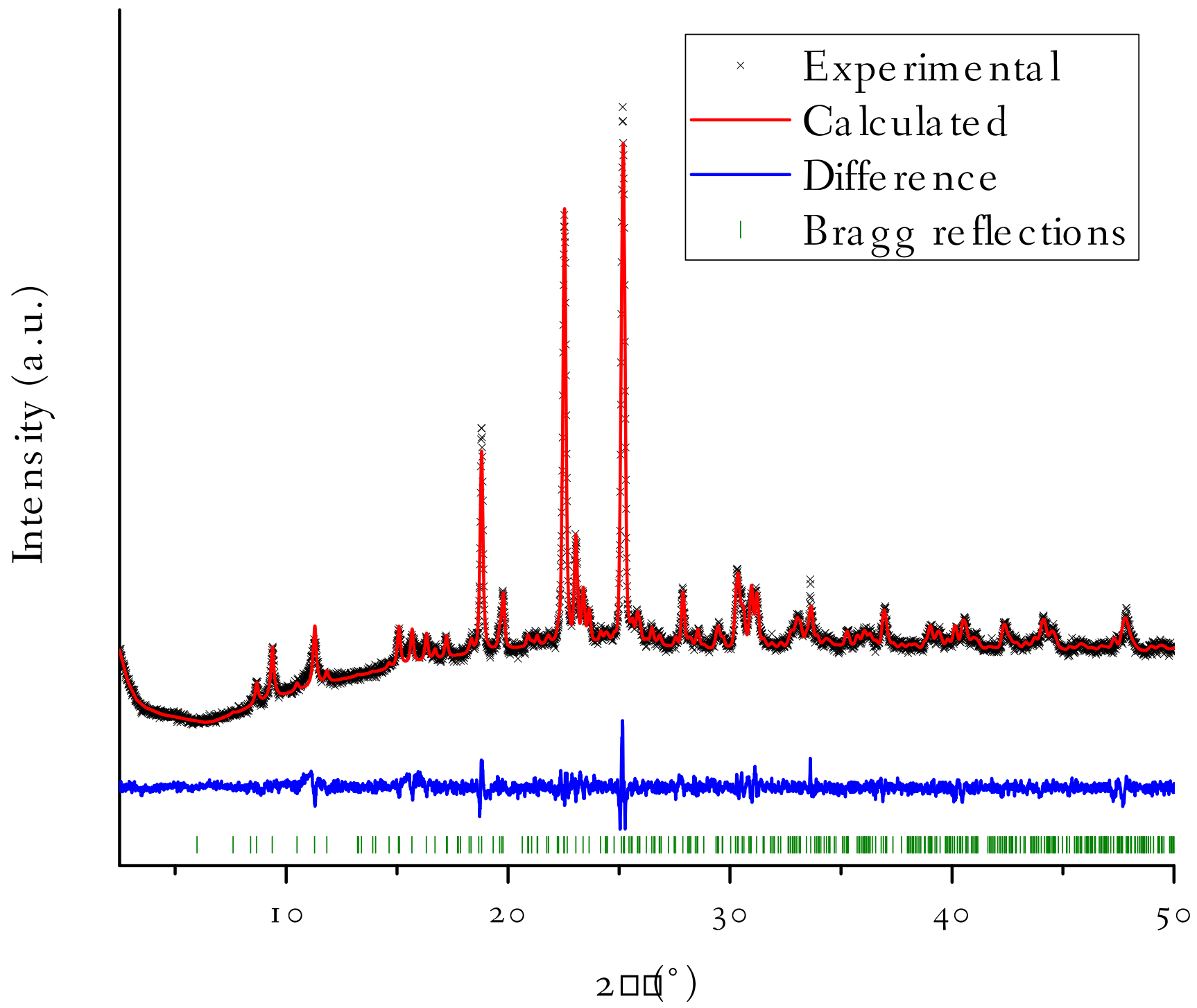

Figure S78. Simulated and experimental PXRD patterns (top) and Pawley fit (bottom) of [**4**–Ag–**4**]PF_6.

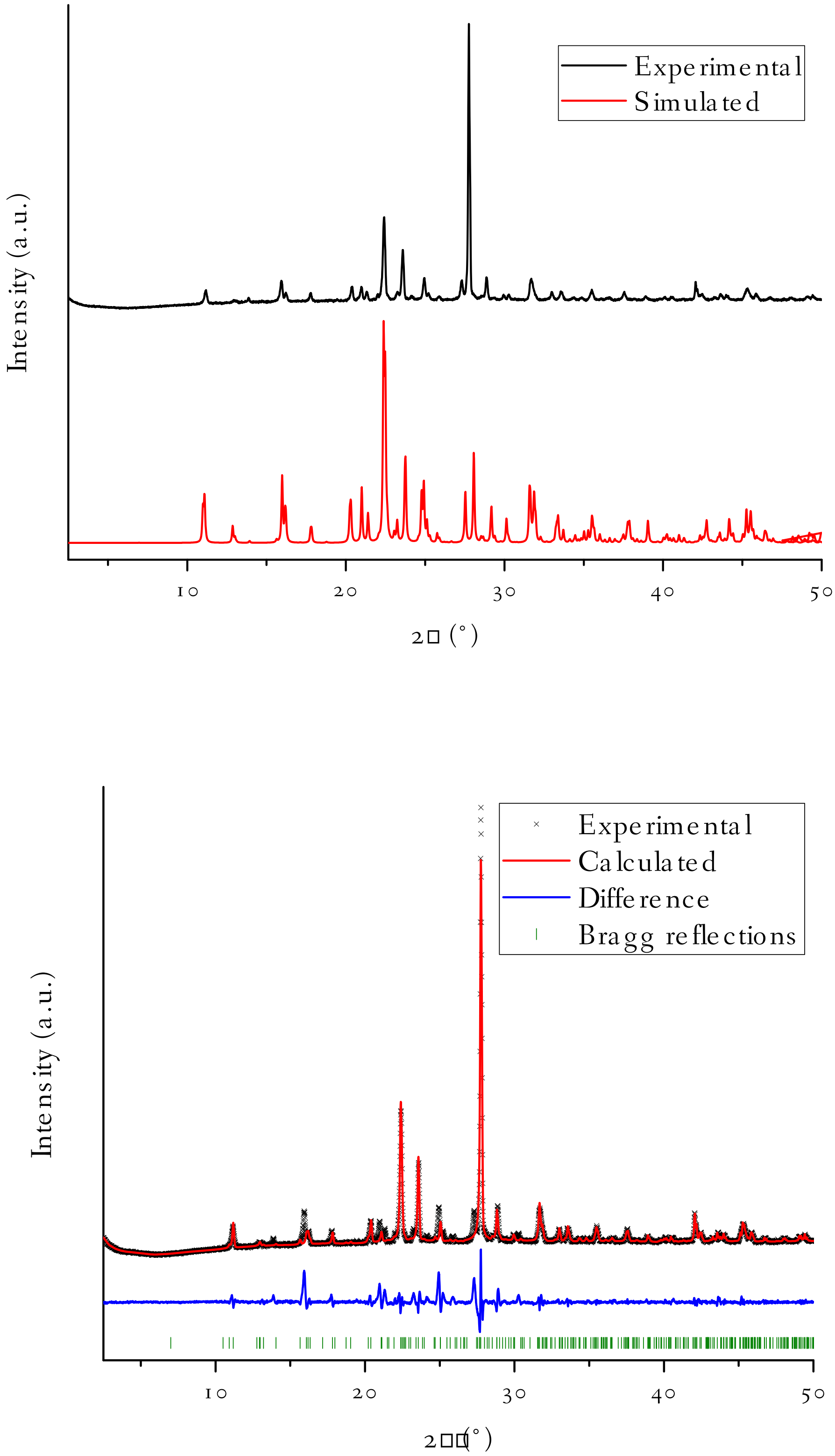

Figure S79. Simulated and experimental PXRD patterns (top) and Pawley fit (bottom) of [**4**–I–**4**]PF_6.

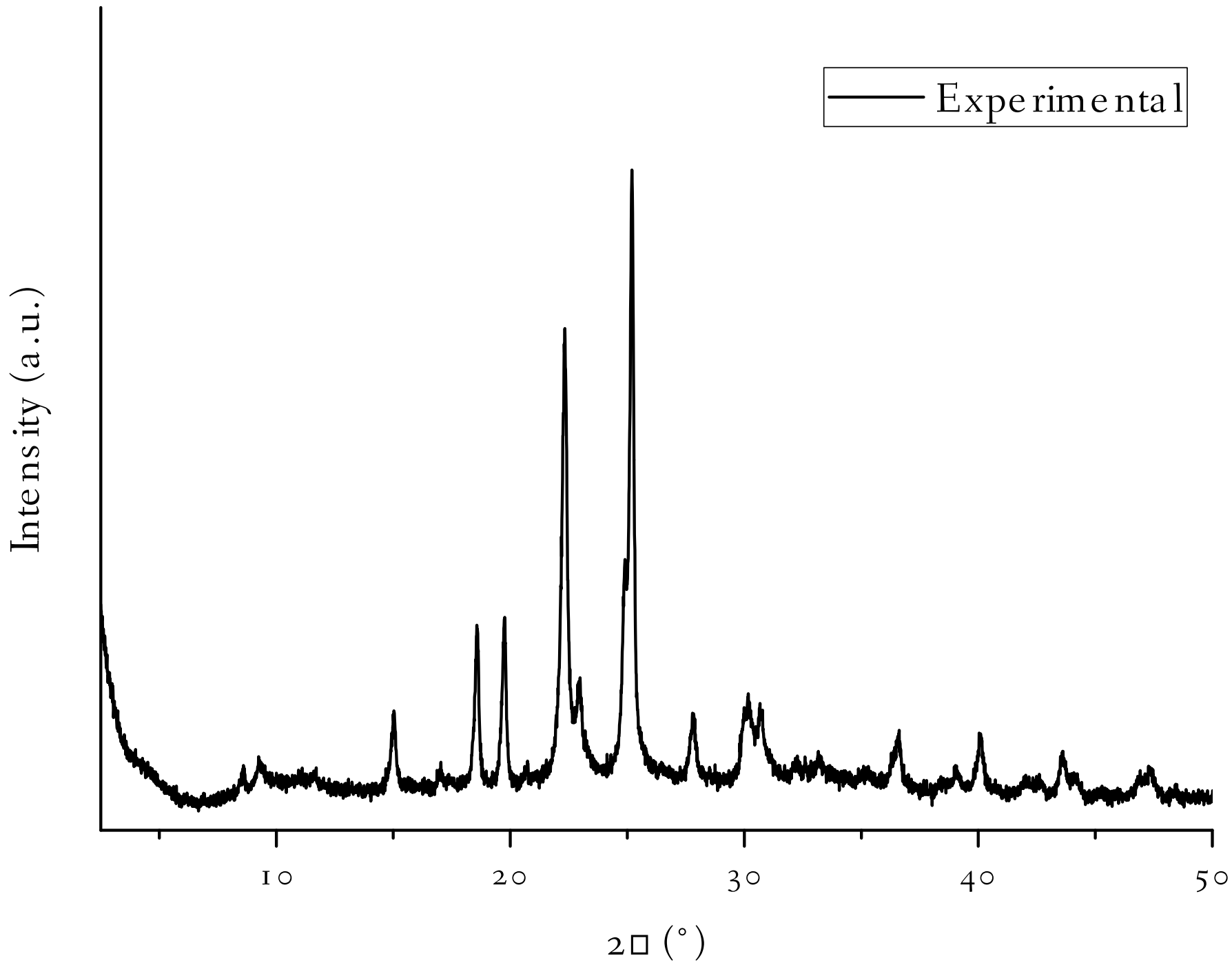

Figure S80. Experimental PXRD pattern of [**5**–Ag–**5**]PF_6.

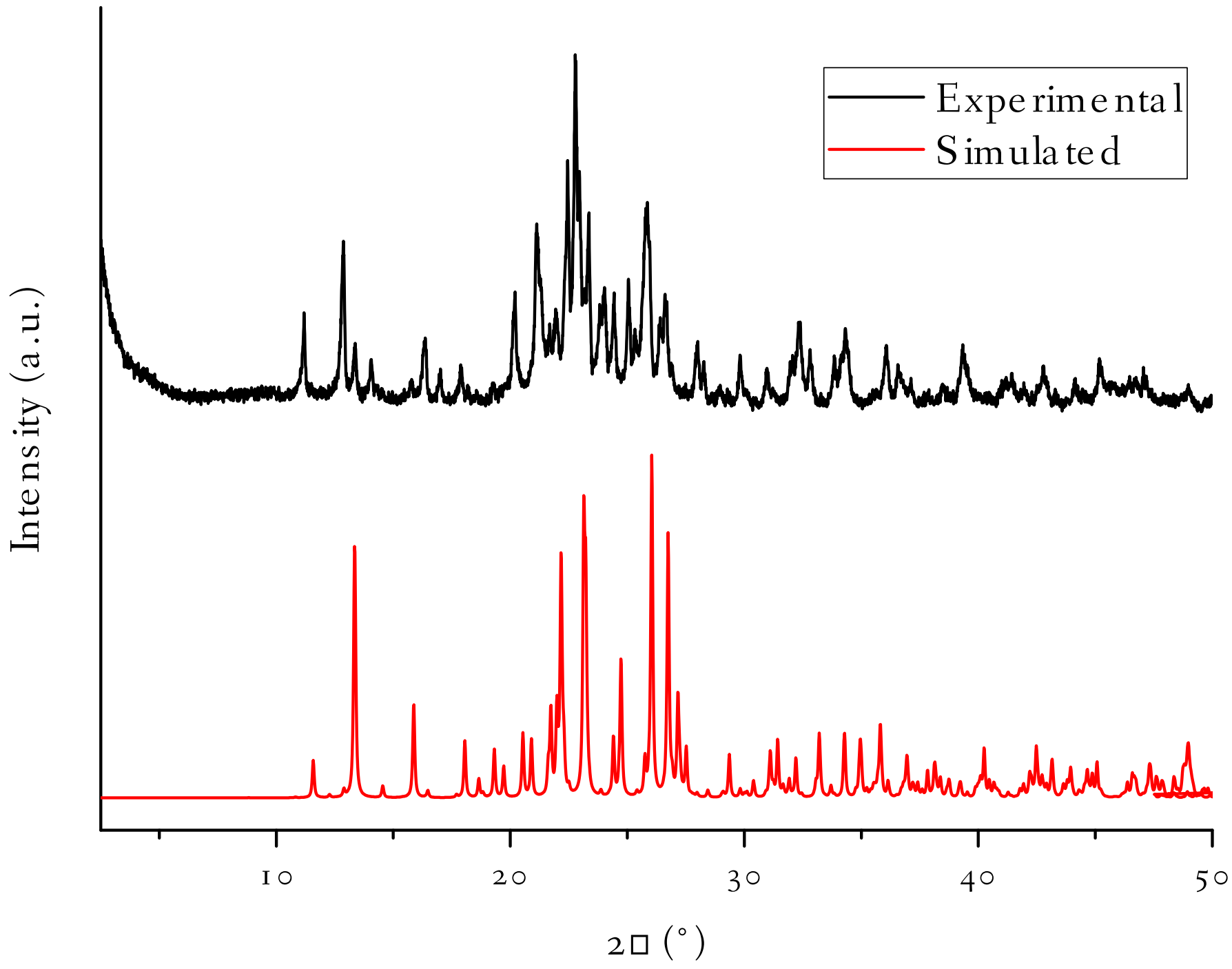

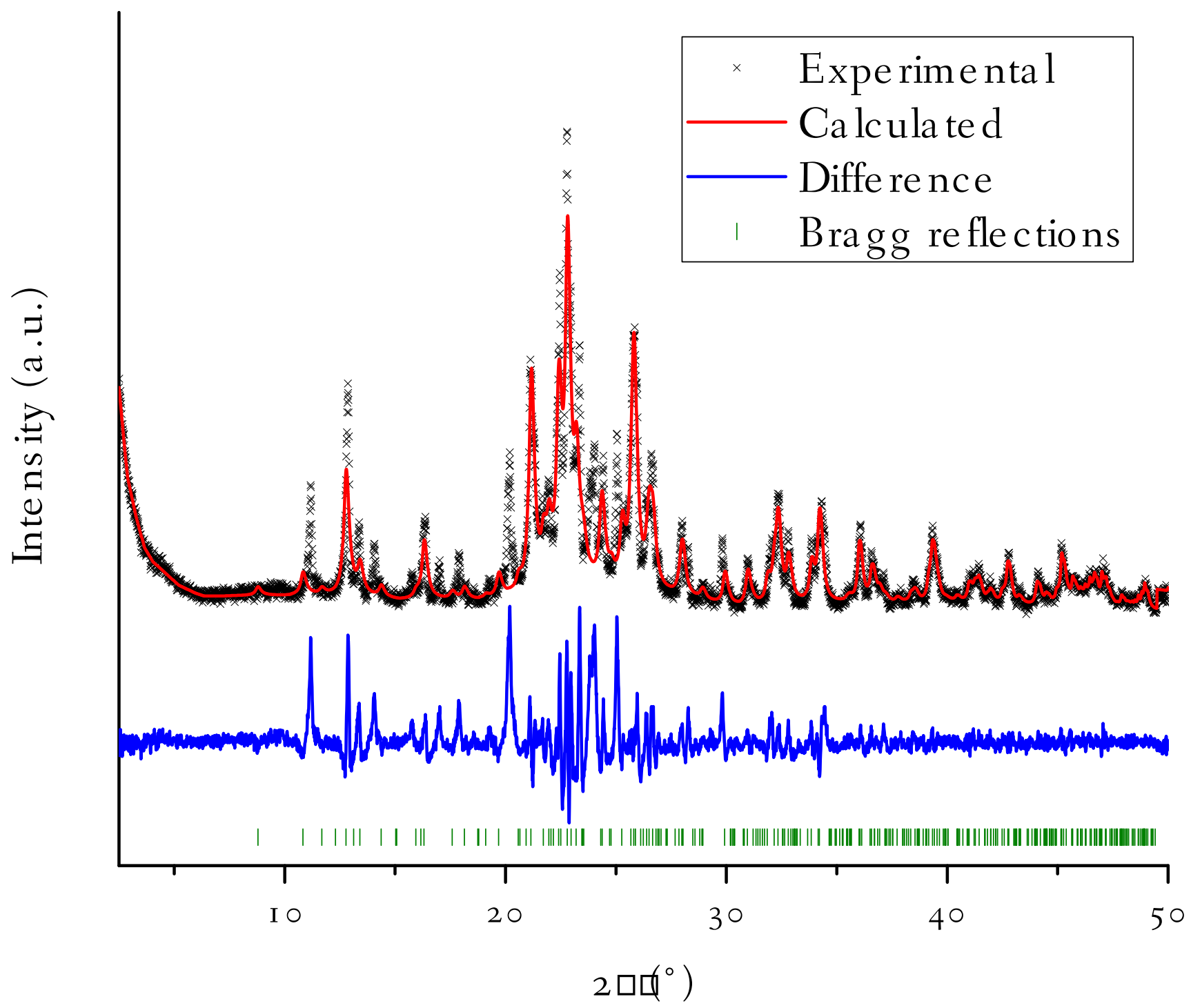

Figure S81. Simulated and experimental PXRD patterns (top) and Pawley fit (bottom) of [**5**–I–**5**]PF_6.

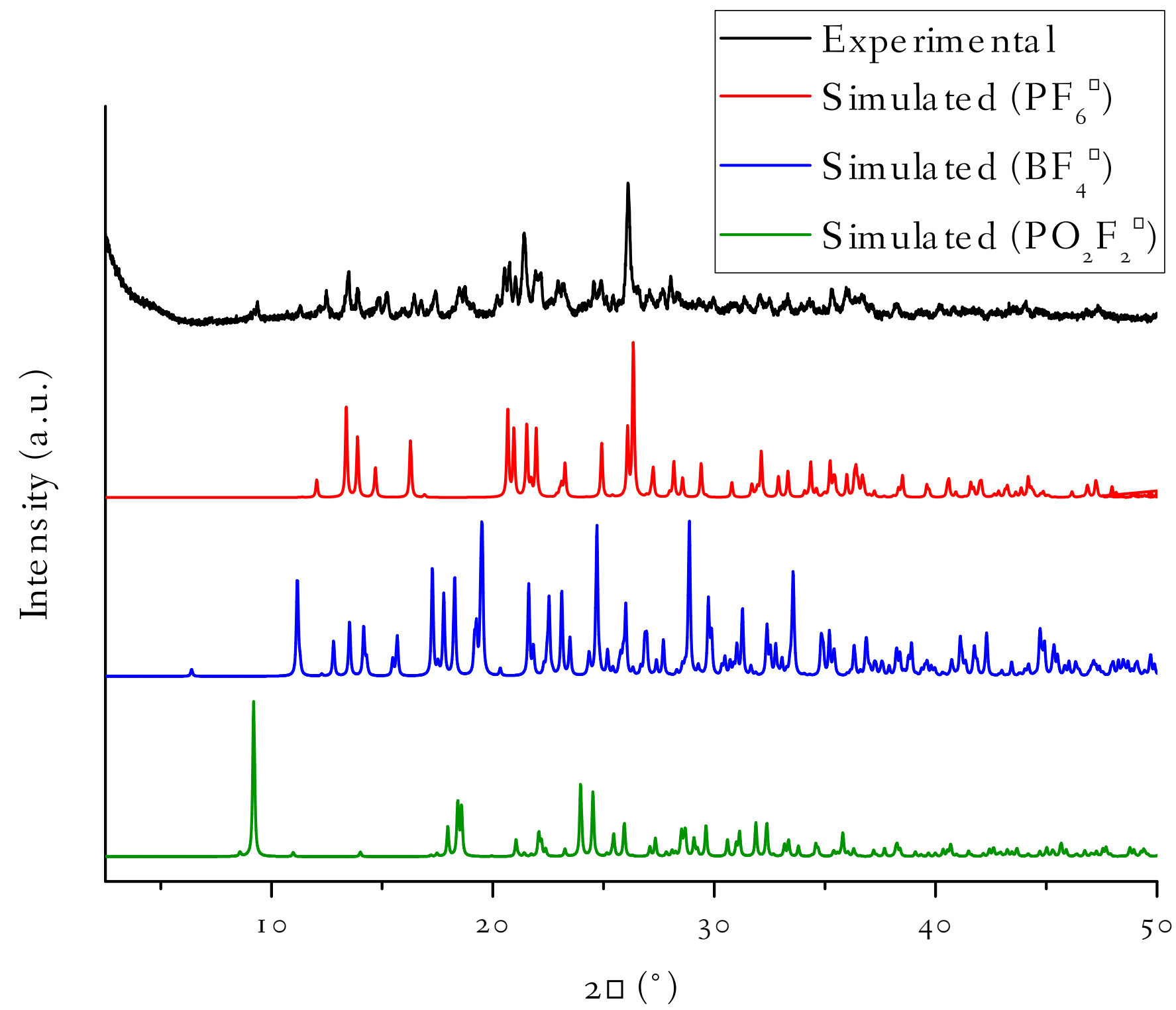

Figure S82. Simulated and experimental PXRD patterns of [**6**–Ag–**6**]PF_6 and [**6**–Ag–**6**]BF_4.

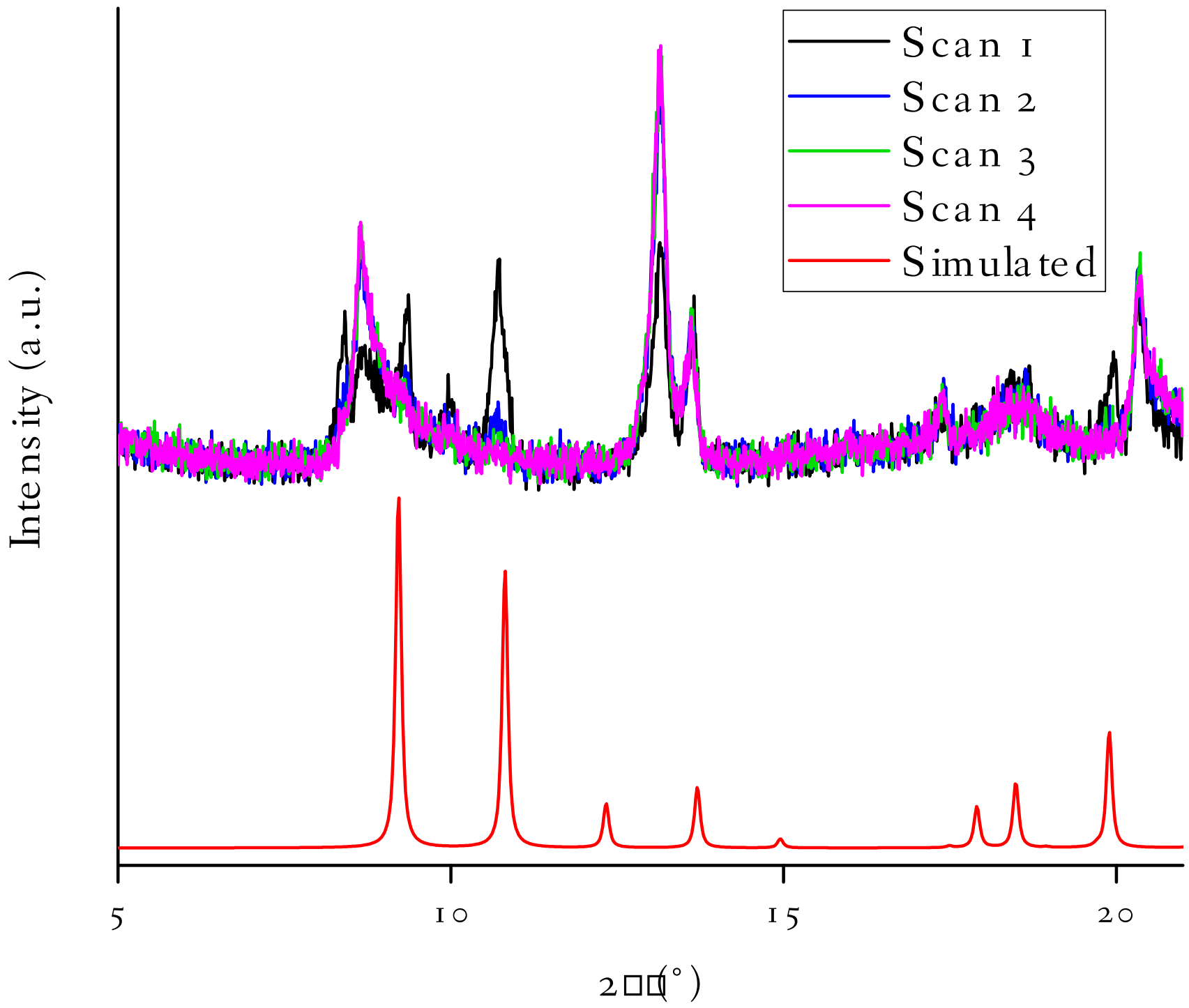

Figure S83. Comparison of the simulated pattern and the four individual scans of a measurement of [**2**–Ag–**2**]PF_6.

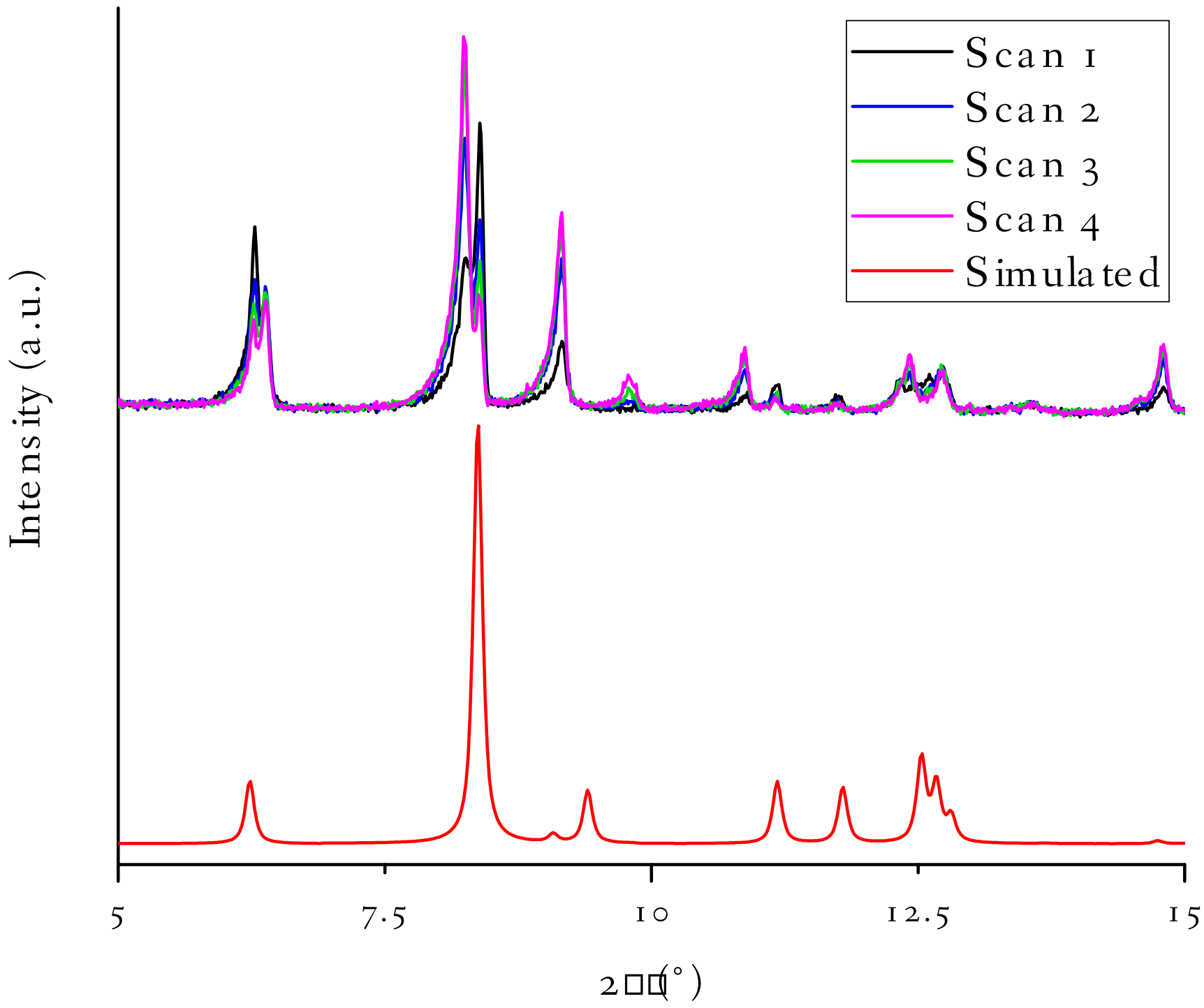

Figure S84. Comparison of the simulated pattern and the four individual scans of a measurement of [**3**–Ag–**3**]PF_6.

Table S2. Lattice parameters for the complexes of ligands **1**–**6** obtained from Pawley fits.

	[**1**–Ag–**1**]PF_6	[**1**–I–**1**]PF_6	[**2**–Ag–**2**]PF_6	[**2**–I–**2**]PF_6	[**3**–Ag–**3**]PF_6	[**3**–I–**3**]PF_6
a (Å)	8.3156(4)	–	–	12.040(1)	10.651(1)	11.8893(5)
b (Å)	17.9815(8)	–	–	8.614(1)	28.523(3)	10.7202(5)
c (Å)	11.0133(5)	–	–	14.881(1)	16.436(2)	13.1379(4)
▱(°)	90	–	–	90	90	90
▱(°)	103.4373(7)	–	–	102.1792(9)	105.810(3)	108.9147(5)
▱(°)	90	–	–	90	90	90
V(Å^3)	1601.7(2)	–	–	1508.6(3)	4804(2)	1584.1(2)
R_{wp} (%)	6.07801	–	–	5.11952	18.96785	5.39484

	[**4**–Ag–**4**]PF_6	[**4**–I–**4**]PF_6	[**5**–Ag–**5**]PF_6	[**5**–I–**5**]PF_6	[6–Ag–6]PF_6	[6–Ag–6]BF_4	[6–I–6]PF_6
a (Å)	21.384(5)	15.814(1)	–	8.397(2)	–	–	–
b (Å)	21.384(5)	16.192(1)	–	15.180(4)	–	–	–
c (Å)	14.201(4)	25.211(2)	–	13.856(4)	–	–	–
▱(°)	90	90	–	90	–	–	–
▱(°)	90	90	–	102.698(2)	–	–	–
▱(°)	90	90	–	90	–	–	–

V(Å^3)	6494(3)	6455.7(9)	–	1723.0(8)	–	–	–
R_{wp}(%)	3.60203	10.59855	–	12.78950	–	–	–

6. DFT studies

Each of the Ag(I) and I(I) complex structures of 3-substituted pyridines (**1**–**5**) were optimized at the M06-2X/def2-TZVP[8–10] level using the Polarizable Continuum Model (PCM) with CH_2Cl_2 as the solvent using Gaussian 16, Revision C.02.[11] Pseudopotentials were used for Br, I and Ag. Frequency calculations were carried out to the optimized structures to confirm that their geometries represent the local energy minima. The energies are reported in Table S3 and the *xyz*-coordinates in Table S4.

Table S3. List of energies (in hartrees) obtained by optimization of each of the complex cations at the M06X/def2-TZVP level. The energy difference between the *anti* and *syn* conformers is reported both in hartrees and in kJ mol^{-1}.

	**1**-*anti*	**1**-*syn*	$E_{\text{anti-syn}}$	$E_{\text{anti-syn}}$ (kJ mol^{-1})
Ag	-721.996298	-721.997014	0.000716	1.88
I	-872.636512	-872.636509	-0.000003	-0.01
	**2**-*anti*	**2**-*syn*	$E_{\text{anti-syn}}$	$E_{\text{anti-syn}}$ (kJ mol^{-1})
Ag	-841.870052	-841.870029	-0.000023	-0.06
I	-992.505174	-992.505157	-0.000017	-0.05
	**3**-*anti*	**3**-*syn*	$E_{\text{anti-syn}}$	$E_{\text{anti-syn}}$ (kJ mol^{-1})
Ag	-1562.571693	-1562.571685	-0.000009	-0.02
I	-1713.206683	-1713.206671	-0.000012	-0.03
	**4**-*anti*	**4**-*syn*	$E_{\text{anti-syn}}$	$E_{\text{anti-syn}}$ (kJ mol^{-1})
Ag	-5790.558429	-5790.558437	0.000008	0.02
I	-5941.193391	-5941.193379	-0.000012	-0.03
	**5**-*anti*	**5**-*syn*	$E_{\text{anti-syn}}$	$E_{\text{anti-syn}}$ (kJ mol^{-1})
Ag	-1237.429318	-1237.429324	0.000006	0.02
I	-1388.064845	-1388.064834	-0.000010	-0.03

Table S4. List of *xyz*-coordinates of the optimized Ag(I) and I(I) complexes of **1**–**5**.

[**1**–Ag–**1**]$^+$ *anti*

C	-5.908815	-7.447037	-0.586081
C	-4.821629	-8.305926	-0.642958
C	-3.531125	-7.794554	-0.591799
C	-3.403154	-6.414110	-0.478826
N	-4.443750	-5.580332	-0.423618
C	-5.680847	-6.088600	-0.477549
C	-2.313816	-8.668715	-0.665503
Ag	-4.144356	-3.394740	-0.267901
N	-3.848749	-1.206779	-0.112248
C	-4.896478	-0.379498	-0.084269
C	-4.779644	1.001732	0.030068
C	-3.493904	1.521665	0.112060
C	-2.400273	0.670019	0.082311
C	-2.617239	-0.690062	-0.029507
C	-6.003703	1.868386	0.073820
H	-5.875511	-0.837599	-0.158247
H	-3.349659	2.591869	0.197329
H	-1.793783	-1.390972	-0.055400
H	-1.390793	1.050564	0.143519
H	-2.420037	-5.960568	-0.429339
H	-4.974651	-9.375030	-0.725683
H	-6.500569	-5.384752	-0.429721
H	-6.921848	-7.821310	-0.623254
H	-5.739597	2.918360	-0.033342
H	-6.699569	1.603143	-0.721772
H	-6.526255	1.745441	1.023680
H	-2.582653	-9.717669	-0.559300
H	-1.809588	-8.543350	-1.624847
H	-1.600394	-8.412715	0.117540

[**1**–Ag–**1**]$^+$ *syn*

C	-4.867616	-0.470701	0.645870
N	-3.890396	-1.233918	0.151830
C	-2.707223	-0.670794	-0.119792
C	-2.435557	0.673676	0.086919
C	-3.465809	1.455592	0.602333
C	-4.692595	0.881926	0.884350
Ag	-4.204409	-3.397631	-0.180242
N	-4.455894	-5.564180	-0.537760
C	-3.475786	-6.419441	-0.223441
C	-3.556662	-7.787022	-0.441642
C	-4.728812	-8.267916	-1.018925
C	-5.746518	-7.390657	-1.347534
C	-5.572868	-6.041182	-1.091608
C	-2.428309	-8.702051	-0.069637
C	-1.088759	1.252387	-0.229630
H	-1.942943	-1.325717	-0.520141
H	-3.301822	2.511671	0.780930
H	-5.809867	-0.959661	0.853769
H	-5.508175	1.466921	1.284763
H	-2.587116	-5.989545	0.222632
H	-4.838721	-9.328926	-1.209282
H	-6.343054	-5.321255	-1.334205
H	-6.665666	-7.738245	-1.797073
H	-2.075996	-9.249903	-0.944121
H	-1.591433	-8.146223	0.348877
H	-2.757855	-9.435511	0.667110
H	-1.180879	2.064725	-0.951070
H	-0.628210	1.662193	0.669985
H	-0.422416	0.497887	-0.643495

[**2**–Ag–**2**]$^+$ *anti*

C	-4.918849	-0.379861	-0.074864
C	-4.752715	0.986413	0.037627
C	-3.495240	1.549398	0.094565
C	-2.411794	0.689115	0.033571
C	-2.634319	-0.672998	-0.079000
N	-3.863645	-1.194586	-0.131751
H	-5.909530	-0.812290	-0.117681
H	-3.379385	2.620986	0.183344
F	-5.836703	1.760289	0.091811
H	-1.808758	-1.369438	-0.127244
H	-1.400839	1.067711	0.073195
Ag	-4.154295	-3.394655	-0.273016
C	-3.389631	-6.409334	-0.472509
H	-2.398968	-5.976895	-0.429410
C	-3.555717	-7.775561	-0.585631
N	-4.444862	-5.594665	-0.415345

[**2**–Ag–**2**]$^+$ *syn*

C	-5.898806	-7.471284	-0.621011
C	-4.817795	-8.335333	-0.671219
C	-3.559925	-7.779474	-0.570721
C	-3.391188	-6.416612	-0.425378
N	-4.443917	-5.598062	-0.378289
C	-5.673571	-6.112909	-0.474080
F	-2.478069	-8.557082	-0.612439
Ag	-4.146143	-3.407739	-0.124608
N	-3.854695	-1.208882	0.033615
C	-2.627026	-0.694041	0.123363
C	-2.436577	0.670699	0.211663
C	-3.505993	1.541251	0.209762
C	-4.772743	0.989680	0.115691
C	-4.907770	-0.385885	0.029451
F	-1.191416	1.138143	0.299609
H	-1.781703	-1.369074	0.125762

C	-4.813171	-8.338556	-0.642931
C	-5.674168	-6.116263	-0.468425
C	-5.896646	-7.478330	-0.581632
H	-4.928989	-9.410107	-0.732207
H	-6.499756	-5.419872	-0.419928
F	-2.471702	-8.549383	-0.640070
H	-6.907587	-7.856937	-0.621509

[**3**–Ag–**3**]$^+$ *anti*

C	-4.894531	-0.373786	-0.091862
C	-4.738067	0.997850	0.021965
C	-3.466529	1.541227	0.094000
C	-2.385129	0.677998	0.048163
C	-2.612813	-0.682137	-0.065854
N	-3.844906	-1.195772	-0.134190
H	-5.881067	-0.813934	-0.148777
H	-3.331745	2.610509	0.183412
Cl	-6.134991	2.003652	0.072781
H	-1.791025	-1.383949	-0.102855
H	-1.373454	1.053278	0.100683
Ag	-4.154276	-3.394604	-0.273743
C	-3.413901	-6.415404	-0.456330
H	-2.427345	-5.975247	-0.399796
C	-3.570389	-7.787037	-0.570172
N	-4.463516	-5.593434	-0.413532
C	-4.841948	-8.330428	-0.641727
C	-5.695631	-6.107082	-0.481416
C	-5.923340	-7.467214	-0.595417
H	-4.976753	-9.399707	-0.731146
H	-6.517419	-5.405288	-0.444064
Cl	-2.173466	-8.792806	-0.621601
H	-6.935030	-7.842506	-0.647563

[**4**–Ag–**4**]$^+$ *anti*

C	-4.897193	-0.360545	-0.041487
C	-4.718946	1.006073	0.094660
C	-3.439294	1.534805	0.111197
C	-2.371108	0.662150	-0.012114
C	-2.620421	-0.692210	-0.144969
N	-3.860003	-1.191961	-0.158986
H	-5.888322	-0.793507	-0.055757
H	-3.283188	2.599417	0.217719
Br	-6.228220	2.121964	0.256679
H	-1.809743	-1.401320	-0.242846
H	-1.353950	1.026215	-0.005044
Ag	-4.187106	-3.386758	-0.323626
C	-3.428068	-6.406465	-0.429859
H	-2.448925	-5.954474	-0.347144
C	-3.574010	-7.780931	-0.516819
N	-4.483310	-5.589571	-0.444899
C	-4.839443	-8.332918	-0.622535

H	-3.342553	2.608043	0.279491
H	-5.883242	-0.846222	-0.045283
H	-5.651463	1.617932	0.109014
H	-2.400141	-5.989853	-0.347539
H	-4.935736	-9.404445	-0.784040
H	-6.497285	-5.413976	-0.432349
H	-6.910025	-7.844096	-0.694319

[**3**–Ag–**3**]$^+$ *syn*

C	-5.924100	-7.481502	-0.618410
C	-4.833249	-8.334061	-0.608628
C	-3.569654	-7.775380	-0.517290
C	-3.430113	-6.399422	-0.438838
N	-4.488661	-5.587956	-0.449140
C	-5.713271	-6.116335	-0.537274
Cl	-2.162095	-8.767498	-0.499304
Ag	-4.206003	-3.385978	-0.297243
N	-3.900800	-1.195833	-0.036060
C	-2.667661	-0.691159	0.026601
C	-2.445567	0.667257	0.181514
C	-3.521783	1.533238	0.275565
C	-4.796866	0.997883	0.209910
C	-4.947162	-0.368896	0.053636
Cl	-0.827885	1.253429	0.253098
H	-1.837145	-1.380091	-0.047119
H	-3.361333	2.595846	0.396318
H	-5.928571	-0.819751	-0.002129
H	-5.668641	1.632149	0.278307
H	-2.450114	-5.947272	-0.366822
H	-4.954943	-9.406862	-0.670043
H	-6.542129	-5.421814	-0.542661
H	-6.930035	-7.869215	-0.687844

[**4**–Ag–**4**]$^+$ *syn*

C	-5.943417	-7.461868	-0.595399
C	-4.862778	-8.323003	-0.687550
C	-3.590115	-7.783423	-0.608826
C	-3.430926	-6.417809	-0.441837
N	-4.479758	-5.597329	-0.354693
C	-5.712908	-6.107813	-0.429841
Br	-2.064788	-8.882620	-0.727570
Ag	-4.174313	-3.406577	-0.093293
N	-3.892460	-1.203160	0.047337
C	-2.668204	-0.682881	0.151734
C	-2.465351	0.685001	0.226464
C	-3.550797	1.543935	0.192807
C	-4.816533	0.992172	0.084500
C	-4.947831	-0.383421	0.014226
Br	-0.708677	1.349613	0.371873
H	-1.834404	-1.371426	0.175716
H	-3.409744	2.614471	0.249528

C	-5.709492	-6.111609	-0.545631
C	-5.926619	-7.475019	-0.636224
H	-4.970448	-9.403984	-0.692161
H	-6.535063	-5.413178	-0.554099
Br	-2.040604	-8.874433	-0.488668
H	-6.933796	-7.857561	-0.716426

H	-5.920690	-0.847965	-0.071211
H	-5.694611	1.620859	0.054628
H	-2.445561	-5.976426	-0.377408
H	-5.004286	-9.387025	-0.817639
H	-6.533766	-5.407814	-0.356113
H	-6.955864	-7.834712	-0.651288

[**5**–Ag–**5**]$^+$ *anti*

C	-4.877895	-0.369246	-0.089499
C	-4.719205	1.003271	0.021783
C	-3.441081	1.535457	0.095011
C	-2.362492	0.666905	0.053363
C	-2.597392	-0.691669	-0.058689
N	-3.832018	-1.198603	-0.128571
H	-5.861444	-0.815991	-0.148519
H	-3.287974	2.602544	0.182258
I	-6.400727	2.227911	0.075006
H	-1.779357	-1.398186	-0.093282
H	-1.348928	1.037380	0.107176
Ag	-4.154335	-3.394564	-0.274333
C	-3.430575	-6.419850	-0.458883
H	-2.447033	-5.973029	-0.400343
C	-3.589217	-7.792427	-0.569471
N	-4.476485	-5.590533	-0.419881
C	-4.867330	-8.324716	-0.642143
C	-5.711097	-6.097566	-0.489248
C	-5.945954	-7.456200	-0.600645
H	-5.020403	-9.391851	-0.728849
H	-6.529164	-5.391084	-0.454771
I	-1.907651	-9.017023	-0.622457
H	-6.959512	-7.826750	-0.654055

[**5**–Ag–**5**]$^+$ *syn*

C	-5.957774	-7.467104	-0.566550
C	-4.872254	-8.326727	-0.612913
C	-3.597899	-7.782112	-0.571830
C	-3.449796	-6.406609	-0.485387
N	-4.502317	-5.585983	-0.441084
C	-5.733373	-6.104873	-0.481145
I	-1.906189	-8.992217	-0.634758
Ag	-4.200507	-3.387780	-0.283752
N	-3.908146	-1.196073	-0.032067
C	-2.682089	-0.681243	0.091041
C	-2.472340	0.680627	0.240974
C	-3.562323	1.536986	0.263942
C	-4.830206	0.993760	0.135838
C	-4.962957	-0.375371	-0.010243
I	-0.531946	1.407809	0.428780
H	-1.853294	-1.376111	0.069479
H	-3.427055	2.603670	0.378943
H	-5.937070	-0.834109	-0.113121
H	-5.707977	1.623606	0.148650
H	-2.469422	-5.950504	-0.450250
H	-5.017214	-9.396441	-0.679406
H	-6.556972	-5.404916	-0.443330
H	-6.968662	-7.847508	-0.595665

[**1**–I–**1**]$^+$ *anti*

C	-5.900196	-7.468289	-0.714745
C	-4.807051	-8.316686	-0.727597
C	-3.516331	-7.803709	-0.615295
C	-3.392899	-6.429445	-0.492211
N	-4.453981	-5.617093	-0.480313
C	-5.688566	-6.107936	-0.588271
C	-2.309447	-8.692015	-0.627261
I	-4.153129	-3.394512	-0.274179
N	-3.853398	-1.171830	-0.067596
C	-4.914956	-0.360104	-0.055469
C	-4.792328	1.014206	0.067867
C	-3.501905	1.527924	0.180212
C	-2.408261	0.680172	0.167102
C	-2.619093	-0.680282	0.040354
C	-5.999746	1.901782	0.079795
H	-5.885103	-0.833291	-0.147248
H	-3.358978	2.597394	0.278083
H	-1.805191	-1.392854	0.023396
H	-1.400237	1.059261	0.253085
H	-2.422489	-5.956823	-0.400396
H	-4.950603	-9.386094	-0.825207
H	-6.502050	-5.394884	-0.571499
H	-6.908440	-7.846800	-0.800684
H	-5.946884	2.626013	-0.733662

[**1**–I–**1**]$^+$ *syn*

C	-4.940473	-0.375523	0.118216
N	-3.878976	-1.177273	0.031487
C	-2.640460	-0.676194	0.069272
C	-2.396590	0.681093	0.198237
C	-3.506328	1.518841	0.289193
C	-4.785147	0.991885	0.249507
I	-4.172568	-3.399658	-0.184402
N	-4.467957	-5.621931	-0.398788
C	-3.402815	-6.427109	-0.458941
C	-3.521859	-7.801034	-0.587091
C	-4.812478	-8.322367	-0.651469
C	-5.909790	-7.481742	-0.588731
C	-5.702555	-6.120884	-0.461033
C	-2.310386	-8.680895	-0.653074
C	-0.997578	1.217058	0.239446
H	-1.829690	-1.390312	-0.006649
H	-3.362315	2.587664	0.390397
H	-5.913604	-0.846413	0.080587
H	-5.658012	1.624548	0.317829
H	-2.433065	-5.948169	-0.402070
H	-4.952498	-9.391896	-0.750848
H	-6.519353	-5.413675	-0.407178
H	-6.917932	-7.866716	-0.636775
H	-1.394712	-8.098691	-0.574223

H	-6.915463	1.324335	-0.030055
H	-6.051819	2.459292	1.015623
H	-2.364579	-9.418786	0.183758
H	-2.255672	-9.246590	-1.564754
H	-1.393596	-8.115502	-0.513672
H	-2.327452	-9.411549	0.155869
H	-2.291637	-9.230452	-1.594585
H	-0.850461	1.955410	-0.548931
H	-0.808847	1.710231	1.193418
H	-0.265825	0.422254	0.111101

[**2**–I–**2**]+ *anti*

C	-4.926607	-0.365807	-0.087236
C	-4.755453	0.999341	0.010553
C	-3.494363	1.555977	0.086574
C	-2.408873	0.696756	0.061906
C	-2.624416	-0.665777	-0.036951
N	-3.859763	-1.165335	-0.109030
H	-5.910433	-0.811775	-0.146712
H	-3.377968	2.628399	0.162388
F	-5.833061	1.775702	0.030711
H	-1.809554	-1.376072	-0.059451
H	-1.399229	1.075747	0.118302
I	-4.154322	-3.394601	-0.273940
C	-3.381898	-6.423287	-0.460579
H	-2.398105	-5.977220	-0.401150
C	-3.552946	-7.788451	-0.558329
N	-4.448813	-5.623854	-0.438767
C	-4.813993	-8.345194	-0.634283
C	-5.684124	-6.123514	-0.510759
C	-5.899556	-7.486067	-0.609575
H	-4.930300	-9.417627	-0.710067
H	-6.499046	-5.413290	-0.488222
F	-2.475277	-8.564727	-0.578517
H	-6.909171	-7.865145	-0.665906

[**2**–I–**2**]+ *syn*

C	-5.900249	-7.485943	-0.596029
C	-4.814819	-8.344205	-0.643956
C	-3.553405	-7.788548	-0.567588
C	-3.381713	-6.425322	-0.447350
N	-4.448511	-5.626631	-0.403821
C	-5.684234	-6.125345	-0.474969
F	-2.475758	-8.564174	-0.609154
I	-4.153709	-3.400340	-0.203271
N	-3.859750	-1.174033	-0.002718
C	-2.621558	-0.686182	0.078862
C	-2.430064	0.674144	0.201479
C	-3.501153	1.544308	0.241373
C	-4.772873	1.003936	0.154087
C	-4.920999	-0.365704	0.032221
F	-1.187350	1.136548	0.281093
H	-1.789242	-1.376398	0.047077
H	-3.333148	2.608133	0.337676
H	-5.892827	-0.834075	-0.038189
H	-5.648184	1.635751	0.179893
H	-2.397695	-5.980003	-0.387671
H	-4.931491	-9.415109	-0.737610
H	-6.498914	-5.415807	-0.433906
H	-6.910135	-7.864221	-0.651385

[**3**–I–**3**]+ *anti*

C	-4.903562	-0.357398	-0.110870
C	-4.740769	1.012555	-0.010789
C	-3.465479	1.546489	0.091675
C	-2.383764	0.682167	0.090586
C	-2.606249	-0.677784	-0.012062
N	-3.844559	-1.166770	-0.109663
H	-5.883059	-0.809342	-0.191579
H	-3.328383	2.616192	0.170185
Cl	-6.128123	2.024745	-0.015474
H	-1.797016	-1.395073	-0.017464
H	-1.373530	1.055938	0.168280
I	-4.155229	-3.394480	-0.273648
C	-3.405335	-6.431001	-0.436903
H	-2.426117	-5.978427	-0.356383
C	-3.567188	-7.801013	-0.537438
N	-4.464884	-5.622356	-0.437612
C	-4.842122	-8.335825	-0.639738
C	-5.702884	-6.112190	-0.535010
C	-5.924452	-7.472272	-0.638055
H	-4.978439	-9.405607	-0.718607
H	-6.512591	-5.395473	-0.529155
Cl	-2.179086	-8.812185	-0.533664
H	-6.934450	-7.846709	-0.715651

[**3**–I–**3**]+ *syn*

C	-5.928617	-7.480554	-0.625388
C	-4.839463	-8.335088	-0.651885
C	-3.568056	-7.791997	-0.549329
C	-3.416371	-6.422806	-0.424059
N	-4.482352	-5.622937	-0.401480
C	-5.717121	-6.120897	-0.498423
Cl	-2.172026	-8.791753	-0.575586
I	-4.188784	-3.396110	-0.198738
N	-3.893689	-1.169331	0.002096
C	-2.655724	-0.681935	0.082282
C	-2.443046	0.679576	0.202311
C	-3.527420	1.542715	0.239984
C	-4.802019	1.008758	0.154938
C	-4.954429	-0.359513	0.035802
Cl	-0.834156	1.271823	0.301203
H	-1.834491	-1.385351	0.049965
H	-3.371754	2.608676	0.333516
H	-5.927242	-0.826624	-0.033536
H	-5.674342	1.644962	0.180195
H	-2.440174	-5.963782	-0.342755
H	-4.967906	-9.404265	-0.749929
H	-6.532463	-5.410996	-0.472898
H	-6.936136	-7.861791	-0.702141

[**4**–I–**4**]$^+$ *anti*

C	-4.900017	-0.356942	-0.056541
C	-4.731837	1.011551	0.049612
C	-3.452529	1.544602	0.087332
C	-2.371911	0.680962	0.018376
C	-2.600196	-0.677688	-0.085264
N	-3.842039	-1.166313	-0.121134
H	-5.880135	-0.813384	-0.089425
H	-3.306216	2.612645	0.169690
Br	-6.246677	2.119752	0.143176
H	-1.792397	-1.394646	-0.140322
H	-1.358977	1.054815	0.044733
I	-4.157544	-3.393567	-0.287932
C	-3.420009	-6.437832	-0.346338
H	-2.449467	-5.986142	-0.191300
C	-3.585301	-7.807764	-0.436382
N	-4.469351	-5.621113	-0.448542
C	-4.852377	-8.334622	-0.634563
C	-5.699265	-6.103538	-0.639927
C	-5.924048	-7.463270	-0.737583
H	-4.996183	-9.403852	-0.706121
H	-6.500016	-5.380525	-0.713540
Br	-2.083278	-8.927184	-0.285213
H	-6.927502	-7.831765	-0.891831

[**5**–I–**5**]$^+$ *anti*

C	-4.888191	-0.354432	-0.072370
C	-4.724047	1.016246	0.025899
C	-3.440631	1.541454	0.088017
C	-2.359866	0.674903	0.051178
C	-2.589327	-0.683648	-0.046981
N	-3.831619	-1.168648	-0.106814
H	-5.865492	-0.815583	-0.124127
H	-3.285159	2.608967	0.163810
I	-6.398647	2.245088	0.075375
H	-1.782740	-1.403476	-0.079048
H	-1.346695	1.046466	0.097352
I	-4.154647	-3.394602	-0.273445
C	-3.420461	-6.434485	-0.474633
H	-2.443308	-5.973070	-0.422212
C	-3.584211	-7.805174	-0.573369
N	-4.477264	-5.620565	-0.440365
C	-4.867466	-8.330690	-0.636217
C	-5.719412	-6.105858	-0.500882
C	-5.948479	-7.464443	-0.599523
H	-5.022610	-9.398227	-0.712356
H	-6.526203	-5.386253	-0.468951
I	-1.909261	-9.033528	-0.623175
H	-6.961532	-7.836262	-0.646203

[**4**–I–**4**]$^+$ *syn*

C	-5.942517	-7.477977	-0.594156
C	-4.854160	-8.332655	-0.653254
C	-3.580057	-7.791170	-0.579220
C	-3.424537	-6.423250	-0.448968
N	-4.489733	-5.622628	-0.395249
C	-5.726805	-6.119525	-0.464577
Br	-2.054909	-8.886307	-0.656844
I	-4.193888	-3.395910	-0.192063
N	-3.902525	-1.168258	0.005182
C	-2.666021	-0.679419	0.106952
C	-2.456537	0.682549	0.223004
C	-3.541903	1.545138	0.233681
C	-4.815032	1.010065	0.127099
C	-4.964064	-0.358806	0.013431
Br	-0.698896	1.332748	0.362364
H	-1.847701	-1.386768	0.094239
H	-3.393343	2.612351	0.323218
H	-5.935134	-0.827039	-0.072150
H	-5.687915	1.646101	0.131541
H	-2.448811	-5.960295	-0.388609
H	-4.990687	-9.400263	-0.755493
H	-6.540843	-5.409222	-0.415669
H	-6.951763	-7.858684	-0.648316

[**5**–I–**5**]$^+$ *syn*

C	-5.959810	-7.472476	-0.591661
C	-4.872272	-8.329795	-0.642157
C	-3.592993	-7.795332	-0.573401
C	-3.439600	-6.424905	-0.455910
N	-4.502620	-5.619720	-0.408633
C	-5.741038	-6.113770	-0.473823
I	-1.908816	-9.010142	-0.641339
I	-4.200612	-3.393641	-0.209817
N	-3.909976	-1.166352	-0.009237
C	-2.675715	-0.672968	0.109326
C	-2.466817	0.689131	0.237406
C	-3.560183	1.544381	0.240349
C	-4.831310	1.007490	0.115392
C	-4.974418	-0.360740	-0.007953
I	-0.530557	1.418001	0.425259
H	-1.860160	-1.383834	0.100569
H	-3.423223	2.612427	0.338467
H	-5.942861	-0.832068	-0.106523
H	-5.706080	1.641094	0.114058
H	-2.466056	-5.956440	-0.398717
H	-5.019382	-9.397322	-0.733132
H	-6.553233	-5.400865	-0.430170
H	-6.970048	-7.851376	-0.642208

References

1 P. Kumar, J. M. Rautiainen, J. Novotný, J. S. Ward, R. Marek, K. Rissanen and R. Puttreddy, *Chem. Eur. J.*, 2024, **30**, e202303643.

2 K. Rissanen and J. S. Ward, *ACS Omega*, 2023, **8**, 24064–24071.

3 R. K. Harris, E. D. Becker, S. M. Cabral De Menezes, R. Goodfellow and P. Granger, *Pure Appl. Chem.*, 2001, **73**, 1795–1818.

4 J. C. Cobas and F. J. Sardina, *Concepts Magn. Reson. Part A*, 2003, **19**, 80–96.

5 P. R. Spackman, M. J. Turner, J. J. McKinnon, S. K. Wolff, D. J. Grimwood, D. Jayatilaka and M. A. Spackman, *J. Appl. Crystallogr.*, 2021, **54**, 1006–1011.

6 M. A. Spackman and D. Jayatilaka, *CrystEngComm*, 2009, **11**, 19–32.

7 C. F. Macrae, I. Sovago, S. J. Cottrell, P. T. A. Galek, P. McCabe, E. Pidcock, M. Platings, G. P. Shields, J. S. Stevens, M. Towler and P. A. Wood, *J. Appl. Cryst.*, 2020, **53**, 226–235.

8 Y. Zhao and D. G. Truhlar, *Theor. Chem. Acc.*, 2008, **120**, 215–241.

9 F. Weigend and R. Ahlrichs, *Phys. Chem. Chem. Phys.*, 2005, **7**, 3297–3305.

10 F. Weigend, *Phys. Chem. Chem. Phys.*, 2006, **8**, 1057–1065.

11 M. J. Frisch, G. W. Trucks, H. B. Schlegel, G. E. Scuseria, M. A. Robb, J. R. Cheeseman, G. Scalmani, V. Barone, G. A. Petersson, H. Nakatsuji, X. Li, M. Caricato, A. V. Marenich, J. Bloino, B. G. Janesko, R. Gomperts, B. Mennucci, H. P. Hratchian, J. V. Ortiz, A. F. Izmaylov, J. L. Sonnenberg, D. Williams-Young, F. Ding, F. Lipparini, F. Egidi, J. Goings, B. Peng, A. Petrone, T. Henderson, D. Ranasinghe, V. G. Zakrzewski, J. Gao, N. Rega, G. Zheng, W. Liang, M. Hada, M. Ehara, K. Toyota, R. Fukuda, J. Hasegawa, M. Ishida, T. Nakajima, Y. Honda, O. Kitao, H. Nakai, T. Vreven, K. Throssell, J. A. Montgomery Jr., J. E. Peralta, F. Ogliaro, M. J. Bearpark, J. J. Heyd, E. N. Brothers, K. N. Kudin, V. N. Staroverov, T. A. Keith, R. Kobayashi, J. Normand, K. Raghavachari, A. P. Rendell, J. C. Burant, S. S. Iyengar, J. Tomasi, M. Cossi, J. M. Millam, M. Klene, C. Adamo, R. Cammi, J. W. Ochterski, R. L. Martin, K. Morokuma, O. Farkas, J. B. Foresman and D. J. Fox, *Gaussian 16, Revision C.02*, Gaussian Inc., Wallingford, CT, 2019.
